# Supplementary material for: Pathological variants in TOP3A cause distinct disorders of mitochondrial and nuclear genome stability
Source: EMBO Mol Med. 2023 Apr 4;15(5):e16775. doi: 10.15252/emmm.202216775 (PMC10165364; doi:10.15252/emmm.202216775)
Supplement: Supplementary file 1 — Appendix [file EMMM-15-e16775-s009.pdf]

Appendix for:

"Pathological variants in *TOP3A* cause distinct disorders of mitochondrial and nuclear genome stability"

Direnis Erdinc, Alejandro Rodriguez-Luis *et al.*

Contents: **Page**

Appendix Supplementary Text: Clinical Summaries for Families I-IX	2
Appendix Table S1: Oligonucleotides used	8

Appendix Supplementary Text - Clinical Summaries

Patient (Pa1)

Patient Pa1 is a 72 year-old female who was reported to have bilateral ptosis and complex ophthalmoplegia at the age of 43; gait ataxia and falls were noted from the age of 50. There was a history of bipolar disease and sensorineural hearing loss. Examination revealed bilateral ptosis, complex ophthalmoplegia, cerebellar ataxia, and retained reflexes. ECG showed sinus rhythm with ventricular bigeminy and left axis deviation (consistent with a left anterior hemi block). Echocardiogram was normal at the age of 50. Progression of disease was noted in all areas with the requirement for permanent pacemaker, and also the emergence of the significant proximal myopathy. An axonal sensory motor neuropathy was identified on nerve conduction studies (NCS), causing predominantly sensory symptoms. Left ventricular dysfunction was noted on echocardiogram and it improved with treatment. Subsequent to our previously published studies (Nicholls et al., 2018), a pathogenic *DSP* variant was identified which was felt to be consistent with the cardiac phenotype. Brain MRI revealed bilateral symmetrical changes within the thalami, red nuclei and long tracts within the midbrain, in addition to cerebellar atrophy. There was no other relevant family history.

Patient (Pa2)

Patient Pa2 is a 39 year-old female who developed problems with coordination and gait in her early twenties. She was found to have both cerebellar and sensory ataxia, with distal muscle weakness, foot drop and wasting. She has a bilateral ptosis but no ophthalmoplegia, hearing loss, nasal speech and dysphagia. She also showed dysautonomia with postural hypotension. NCS showed an axonal sensory motor neuropathy. Brain MRI showed cerebellar and spinal cord atrophy, with a lactate peak on brain MRS. Serum lactate 1.1mmol/l. CSF lactate 1.4mmol/l. Left bundle branch block developed on ECG at age 30, progressing to trifascicular block by age 32 requiring pacemaker insertion. Echocardiogram was normal apart from mitral valve prolapse. There was no other relevant family history, both parents and a sibling were clinically unaffected.

Patient (Pa3-1)

Patient Pa3-1 is a 64 year-old male who developed ptosis in his early 50's, and was subsequently found to have a complex ophthalmoplegia. There was a past history of deafness and an isolated seizure at age 56. His younger sister (Patient 3-2; see below) was similarly affected. Examination revealed bilateral ptosis and advanced complex ophthalmoplegia. Mild bi-facial weakness was evident. There was evidence of a mild proximal myopathy and also features of a peripheral neuropathy with distal weakness, sensory loss and a positive Romberg's test. The patient had an ataxic gait and was unable to heel toe walk. MRI brain scan revealed bilateral symmetric high signal within the brain stem, and the globus pallidus and antero-medial thalami. Calcification was noted in the dentate, caudate, lentiform and thalamic nuclei. Non-specific high signal lesions were noted within the white matter. Cerebellar atrophy was present. EMG/nerve conduction studies revealed an axonal sensory motor neuropathy and myopathic changes. An EEG showed marked photosensitivity. His mother was reported to have epilepsy and deafness, but no ophthalmoplegia, myopathy or neuropathy. No further details regarding the diagnosis of his mother's epilepsy were available.

Patient (Pa3-2)

Patient Pa3-2, a 59 year-old female, is the clinically-affected sibling of Patient 3-1. She was diagnosed with photosensitive epilepsy and myoclonus, both of which were well controlled. She was noted to have bilateral ptosis in her late 40's and subsequently found to have a complex ophthalmoplegia. Facial power was normal. She has dysarthria, dysphagia (requiring modified diet), mild right-sided hemiplegia (MRC 4/5) and cerebellar ataxia. She was unable to heel toe walk and had evidence of some proprioceptive issues. She was reported to have previously suffered a supraventricular tachycardia and left ventricular hypertrophy was detected on echocardiogram and cardiac MRI. She was documented to have had a premature menopause. Brain CT scan, following an RTA and orbital fracture, showed mild generalised atrophy consistent with age.

Patient (Pa4)

Patient Pa4 is a 27 year-old female, who first presented at the age of 16 with headache and encephalopathy. She has continued to suffer recurrent encephalopathic episodes, characterised by confusion and neurological deficits, some of which were associated

with pyrexia. After the first episode a progressive sensorineural hearing loss developed, unresponsive to steroids, and she currently uses hearing aids. No ptosis or ophthalmoplegia was noted and she had no clinical presentation of myopathy or ataxia. Although she returned to the usual cognitive baseline between episodes, she developed mild difficulties with executive functions. At the age of 24, she was hospitalised for a similar episode, and required rehabilitation for gait difficulties. During her first pregnancy (age 22) she suffered from preeclampsia. MRI brain scans revealed bilateral and symmetrical T2 hyperintensities in the globus pallidus, thalamus and brain stem (predominantly in the midbrain). Upon laboratory testing, CSF analysis and viral studies were unremarkable. Extended autoantibody screens were normal. Electroencephalogram (EEG) during these episodes demonstrated diffuse generalized slowing, and treatment with antiepileptic drugs was initiated. At the age of 25, she developed presyncope and complete atrioventricular block was observed. Echocardiogram revealed normal cardiac function. Cardiac MRI was not available. Cardiac biopsy was performed, but results were non-specific. Nerve conduction study (NCS) revealed an axonal sensory motor neuropathy without myopathic features on electromyography. There was no other relevant family history demonstrated amongst her unrelated parents or four siblings.

Patient (Pa5-1)

Patient Pa5-1 is a 72 year-old female who reported eyelid ptosis and the emergence of diplopia at the age of 59. She was found to have bilateral ptosis and a complex ophthalmoplegia. There was evidence of a subtle proximal myopathy, but no neuropathic features. She has a past medical history of asthma, cholecystectomy and hypertension. Her younger sister (Patient 5-2; see below) was similarly affected. Her mother and father were both clinically unaffected but passed away at the ages of 75 and 56 respectively. She does not have children. An ECG and echocardiogram at the age of 71 were both normal.

Patient (Pa5-2)

Patient Pa5-2, a 66 year-old female, is the clinically-affected sibling of Patient 5-1. She originally sought opinion for diplopia at the age of 59 and was diagnosed with bilateral ptosis and a complex ophthalmoplegia. There were no symptoms or signs of either myopathy or neuropathy. Cardiac investigations for unexplained collapses were

unremarkable (including echocardiogram and an implantable loop recorder). She had previously suffered pancreatitis and subsequent gall bladder surgery. There was no other relevant family history aside from her clinically-affected sister.

Patient (Pa6)

Patient Pa6 is a 52 year-old female who complained of weakness and wasting bilaterally in the hands. She was referred to the Neurology department where she was found to have bilateral ptosis and complex ophthalmoplegia, bilateral sensorineural hearing loss (reported to date back several decades), proximal weakness and evidence for a sensory motor peripheral neuropathy, with distal wasting, areflexia and reduced sensation to the mid shin. Nerve conduction studies confirmed an axonal sensory motor neuropathy. There was no relevant family history; her unrelated parents, her brother and two children are clinically normal. An ECG showed inverted T-waves in the lateral chest leads and some anterior Q-waves. An MRI brain scan showed extensive white matter changes. These were consistent with small vessel ischemic change. It was noted that there were limited vascular risk factors.

Patient (Pa7)

Patient Pa7 is a 59-years-old male with a 5-years history of progressive imbalance. His symptoms were more noticeable in environments without adequate illumination, and he subsequently developed dysarthria, changes in the volume of his voice and dysphagia. His father had developed balance problems in his early 80s, but none of his relatives had received a diagnosis of ataxia. Physical examination showed ophthalmoparesis in the up-gaze and lateral gaze, with preserved down-gaze, and slowness of saccades. Motor examination was normal, apart from areflexia in both Achilles tendons. Vibration and proprioception were impaired in both lower limbs distally, and pin-prick test revealed hypoesthesia in the feet. He showed mild dysmetria in the four limbs and broad-based gait. The Neuro-Ophthalmological examination showed normal visual acuity and colour vision, and ptosis with frontalis overaction. Fundoscopy revealed a pigmentary retinopathy, with preserved retinal nerve fibre layer.

Over the years, he experienced slow progression of his ataxia (aged 62, his SARA score was 13/40) and oculomotor signs (aged 71, he presented with total ophthalmoplegia with horizontal diplopia). Paraesthesia in both lower limbs, urinary

symptoms (nocturia, increased frequency and urgency) and restless leg syndrome (RLS) were also reported in his last follow-up. He has received treatment with Co-enzyme Q10 (200 mg, tds), with improvement in his fatigue levels, and for his RLS symptoms.

Brain MRI showed moderate cerebellar atrophy, with mild atrophy in the brainstem (midbrain and pons). EMG/NCS revealed a length-dependent axonal sensorimotor polyneuropathy. Neuropsychological assessment identified mild cognitive under-functioning, implicating anterior and bitemporal regions. Muscle biopsy showed SDH-positive/COX-negative fibres and mild changes secondary to denervation. Genetic testing was negative for FRDA, SCA1, SCA2, SCA3, SCA6, SCA7, POLG, PEO1, GFPT1, CHRNE. Sequencing of the whole mtDNA in muscle was normal, and no large scale mtDNA rearrangements were observed. There was no evidence of mitochondrial respiratory chain enzyme deficiency in the muscle biopsy.

Patient (Pa8)

Patient Pa8 is a 42 year old female with myopathy, dysphagia, dysarthria, sensorineural hearing loss, cardiac conduction defects, chronic progressive external ophthalmoplegia, and ptosis. She initially presented at age 21 with syncope and was found to have cardiac conduction defects requiring pacemaker placement. She was then diagnosed with sensorineural hearing loss about 10 years later, and shortly after that she began to notice muscle weakness and swallowing difficulties. EMG/NCV at that time revealed moderate sensory axonal polyneuropathy. Muscle biopsy revealed ragged-red fibres consistent with mitochondrial disease. She requires g-tube feedings due to silent aspiration identified on swallow study. Her muscle weakness has worsened over time, and she now has respiratory muscle involvement with decreased FVC (60%) as well as cardiomyopathy with ejection fraction (EF) of 30-35%.

Patient (Pa9)

Patient Pa9 is a 41-year-old female, born to consanguineous parents, who at the age of 15 developed focal onset seizures presenting with right arm and language abnormalities and no postictal state, accompanied by worsening of academic performance. At the age of 17, a posterior fossa arachnoid cyst was diagnosed. She required placement of a cardiac pacemaker at the age of 21, due to syncope and

arrhythmia and ever since she was seizure free. At the age of 26, she developed distal weakness, atrophy of the distal limbs' musculature and sensory abnormalities. She had early menopause at the age of 28. Posterior fossa arachnoid cyst was drained surgically at the age of 33, with no improvement in the neurological condition that continued to progress. At the age of 38, sensorineural hearing loss was identified. She was evaluated in our service at age 40, presenting a steppage gait with the necessity of unilateral support. In addition, she presented with mild dysmetria and dysarthria. Bilateral eyelid ptosis, ophthalmoparesis with alteration of the vestibulo-ocular reflex were also identified. Vibration, tactile and pain sensory modalities were compromised in distal lower limbs, and she presented global areflexia. Nerve conduction studies depicted an axonal sensory and motor polyneuropathy. Tonal audiometry revealed bilateral neurosensory hearing loss. Brain CT scan only showed the posterior fossa arachnoid cyst and brain MRI was not performed due to the cardiac pacemaker placement.

Appendix Table S1 *- Oligonucleotides used in this study*

'p' indicates 5' phosphorylation, regions in bold denote homology.

Name	Sequence (5'-3')
R1	p-GACTAGAGCAGATATAATACGACT**CACTATAGGG**ATACAATAGGCAGCTGGACGTGTACCAAGTTAGCAGTCATG
R2	p-ATGTCTGG*TTCGTCTCACGACTCATCACG***CCCTATAGTG***GA*TCAGCACACATCATATCACAGC
H1	GTACACGTCCAGCTGCCTATTGTAT CGTGATGAGTCGTGAGACGAA
H2	GTGCTGATCAGTCGTATTA
S1	CTAGTCCATGAC
S1	AGACATGCTGTG
R1 Probe	TCTGCTCTAGTCCATGACTGCTAAC
R2 Probe	CGAACCAGACATGCTGTGATATGAT
Probe A Forward	CTCACCCACTAGGATACCAAC
Probe A Reverse	GATACTGCGACATAGGGTGC
EMSA substrate (80 mer)	ACCACATTAACAACATAAAACCCTCATTCACACGAGAAAACACCCTCATGTTCATACACCTATCCCCCATTCTCCTCCTA
EMSA substrate (40 mer)	TGTTATTATTATGTCCTACAAGCATTAATTAATTAACACA
TOP3A siRNA s14310	CGGCUUGCCUAGUUCUCUAtt
