# Supplementary material for: Pathological variants in TOP3A cause distinct disorders of mitochondrial and nuclear genome stability
Source: EMBO Mol Med. 2023 Apr 4;15(5):e16775. doi: 10.15252/emmm.202216775 (PMC10165364; doi:10.15252/emmm.202216775)
Supplement: Supplementary file 2 — Expanded View Figures PDF [file EMMM-15-e16775-s006.pdf]

Expanded View Figures

Figure EV1. Establishing the phase of TOP3A variants using long-read sequencing.

A–E Creation of an assembled haplotype at the *TOP3A* locus in Pa5-1. (A) Three reads highlighted in brown, orange and blue span the interval between target variants c.298A>G and c.1723A>G. (B) Read-level data for the haplotype assembled reads (highlighted brown and orange), which are reference nucleotide supporting at position c.1723. (C) Read-level data used to create the assembled haplotype through variant-supporting nucleotides at positions c.1282–21 and c.1468–11. (D) Read-level data for the haplotype assembled read (highlighted blue), which is variant supporting at position c.298. (E) A schematic illustration of the assembled haplotype, for target variants c.298A>G and c.1723A>G, which are consistent with a *trans* configuration (arranged on different parental alleles). Nomenclature provided according to transcript NM_004618.5.

F–J Creation of an assembled haplotype at the *TOP3A* locus in Pa6. (F) Two reads highlighted in red and pink span the interval between target variants c.778C>T and c.1723A>G. (G) Read-level data for the haplotype assembled read (highlighted red), which is variant nucleotide supporting at position c.1723. (H) Read-level data used to create the assembled haplotype from reference supporting nucleotides at positions chr17:18195268 and chr17:18195287 (red and pink highlighted reads). (I) Read-level data for the haplotype assembled read (highlighted pink), which is reference supporting at position c.778. (J) A schematic illustration of the assembled haplotype for target variants c.778C>T and c.1723A>G, which are consistent with a *trans* configuration (arranged on different parental alleles). Nomenclature provided according to transcript NM_004618.5.

© 2023 The Authors

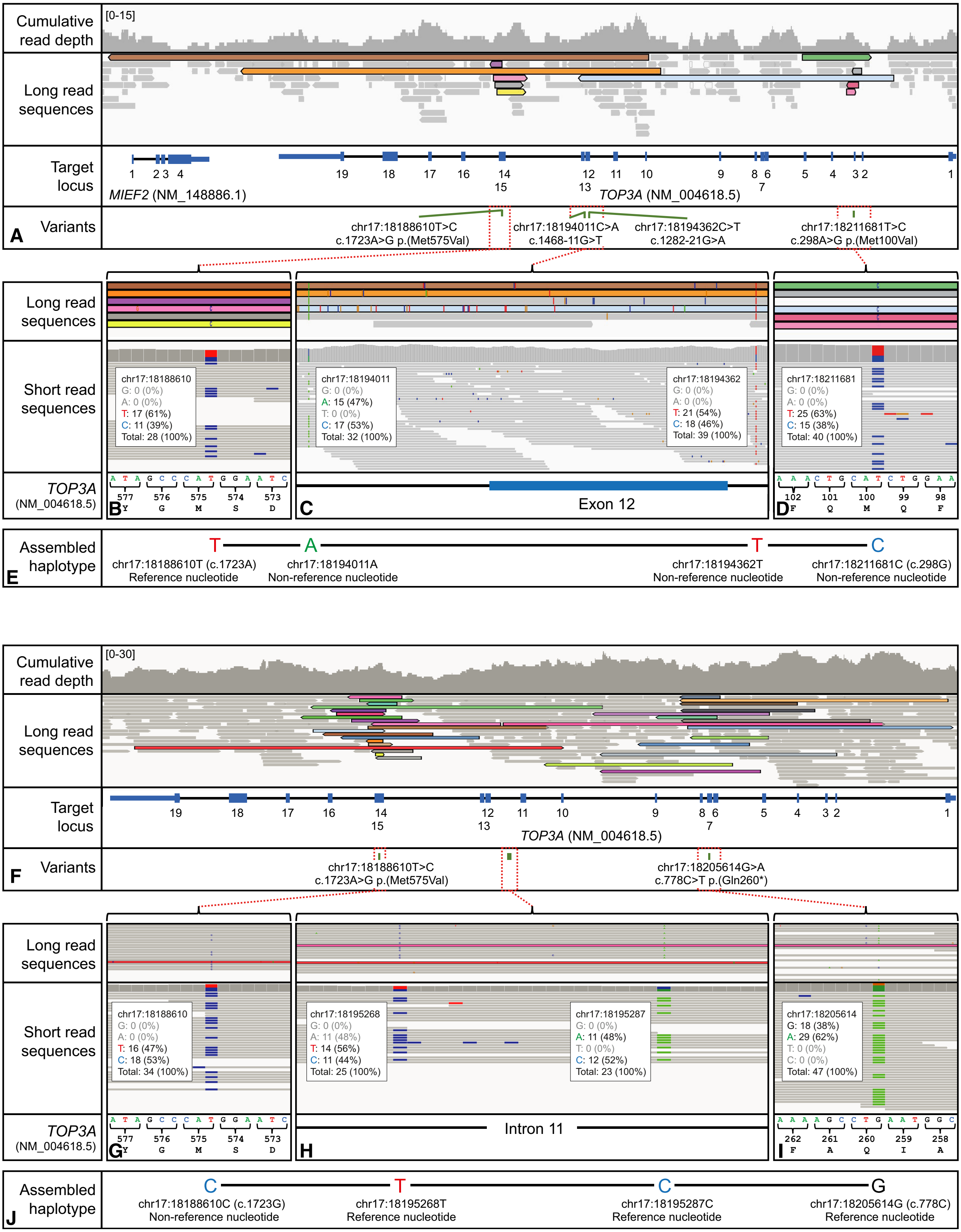

Cumulative read depth
[0-15]
Long read sequences
Target locus
MIEF2 (NM_148886.1)
TOP3A (NM_004618.5)
A
Variants
chr17:18188610T>C
c.1723A>G p.(Met575Val)
chr17:18194011C>A
c.1468-11G>T
chr17:18194362C>T
c.1282-21G>A
chr17:18211681T>C
c.298A>G p.(Met100Val)
Short read sequences
chr17:18188610
G: 0 (0%)
A: 0 (0%)
T: 17 (61%)
C: 11 (39%)
Total: 28 (100%)
chr17:18194011
G: 0 (0%)
A: 15 (47%)
T: 0 (0%)
C: 17 (53%)
Total: 32 (100%)
chr17:18194362
G: 0 (0%)
A: 0 (0%)
T: 21 (54%)
C: 18 (46%)
Total: 39 (100%)
chr17:18211681
G: 0 (0%)
A: 0 (0%)
T: 25 (63%)
C: 15 (38%)
Total: 40 (100%)
TOP3A (NM_004618.5)
B
C
Exon 12
D
E
Assembled haplotype
chr17:18188610T (c.1723A)
Reference nucleotide
chr17:18194011A
Non-reference nucleotide
chr17:18194362T
Non-reference nucleotide
chr17:18211681C (c.298G)
Non-reference nucleotide
Cumulative read depth
[0-30]
Long read sequences
Target locus
TOP3A (NM_004618.5)
F
Variants
chr17:18188610T>C
c.1723A>G p.(Met575Val)
chr17:18205614G>A
c.778C>T p.(Gln260*)
Long read sequences
Short read sequences
chr17:18188610
G: 0 (0%)
A: 0 (0%)
T: 16 (47%)
C: 18 (53%)
Total: 34 (100%)
chr17:18195268
G: 0 (0%)
A: 11 (48%)
T: 14 (56%)
C: 11 (44%)
Total: 25 (100%)
chr17:18195287
G: 0 (0%)
A: 11 (48%)
T: 0 (0%)
C: 12 (52%)
Total: 23 (100%)
chr17:18205614
G: 18 (38%)
A: 29 (62%)
T: 0 (0%)
C: 0 (0%)
Total: 47 (100%)
TOP3A (NM_004618.5)
G
H
Intron 11
I
J
Assembled haplotype
chr17:18188610C (c.1723G)
Non-reference nucleotide
chr17:18195268T
Reference nucleotide
chr17:18195287C
Reference nucleotide
chr17:18205614G (c.778C)
Reference nucleotide

Figure EV1.

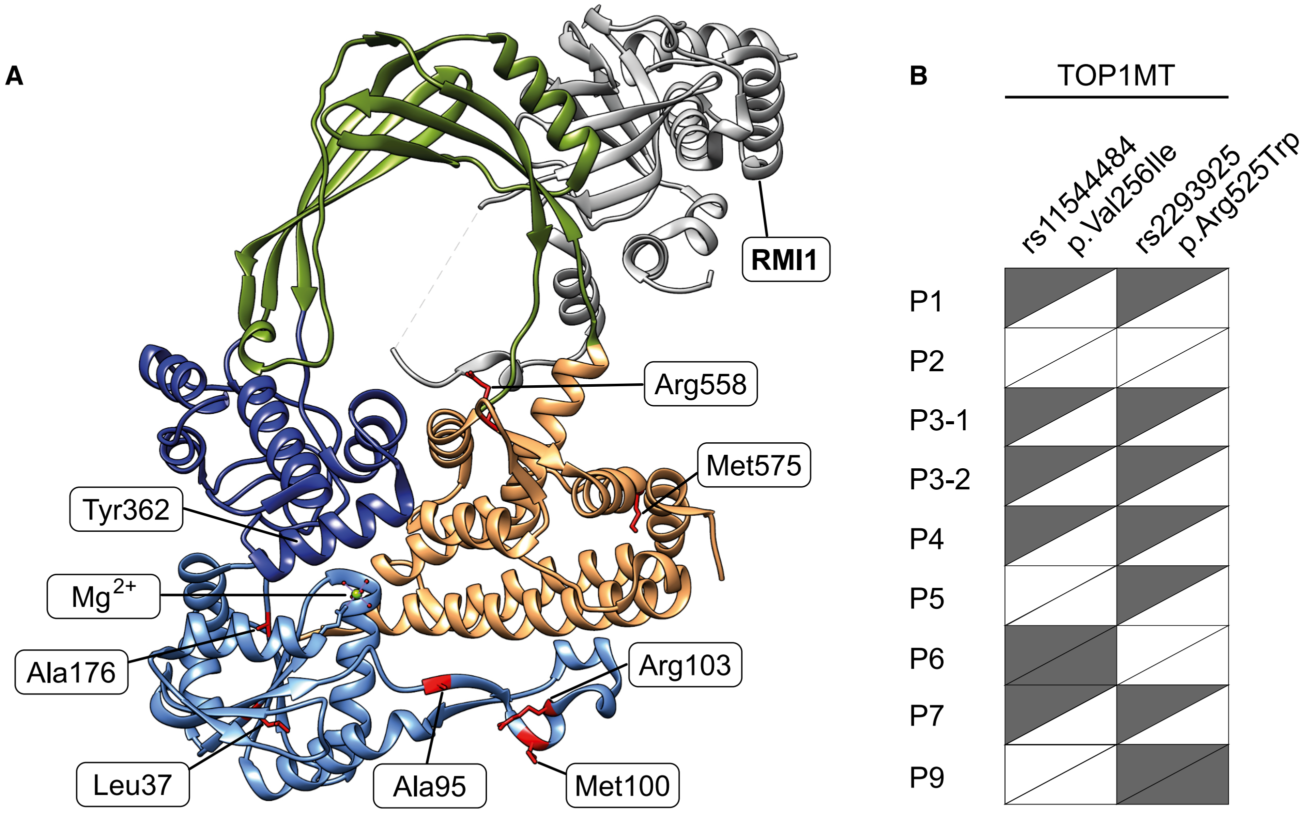

Figure EV2. Modelling of TOP3A variants and SNPs.

A The location of pathological variants within the crystal structure of TOP3A, including RMI1, a nuclear-binding partner of TOP3A (PDB: 4CGY). The affected residues are shown in red, and domains are coloured according to format shown in Fig 1B.

B The presence of previously identified SNPs in TOP1MT (rs11544484, p.Val256Ile; and rs2293925, p.Arg525Trp; Zhang *et al*, 2017) in TOP3A patients reported in this study for which the information is available. Half-filled rectangles indicate a heterozygous SNP, and filled rectangles indicate a homozygous SNP.

Source data are available online for this figure.

 © 2023 The Authors

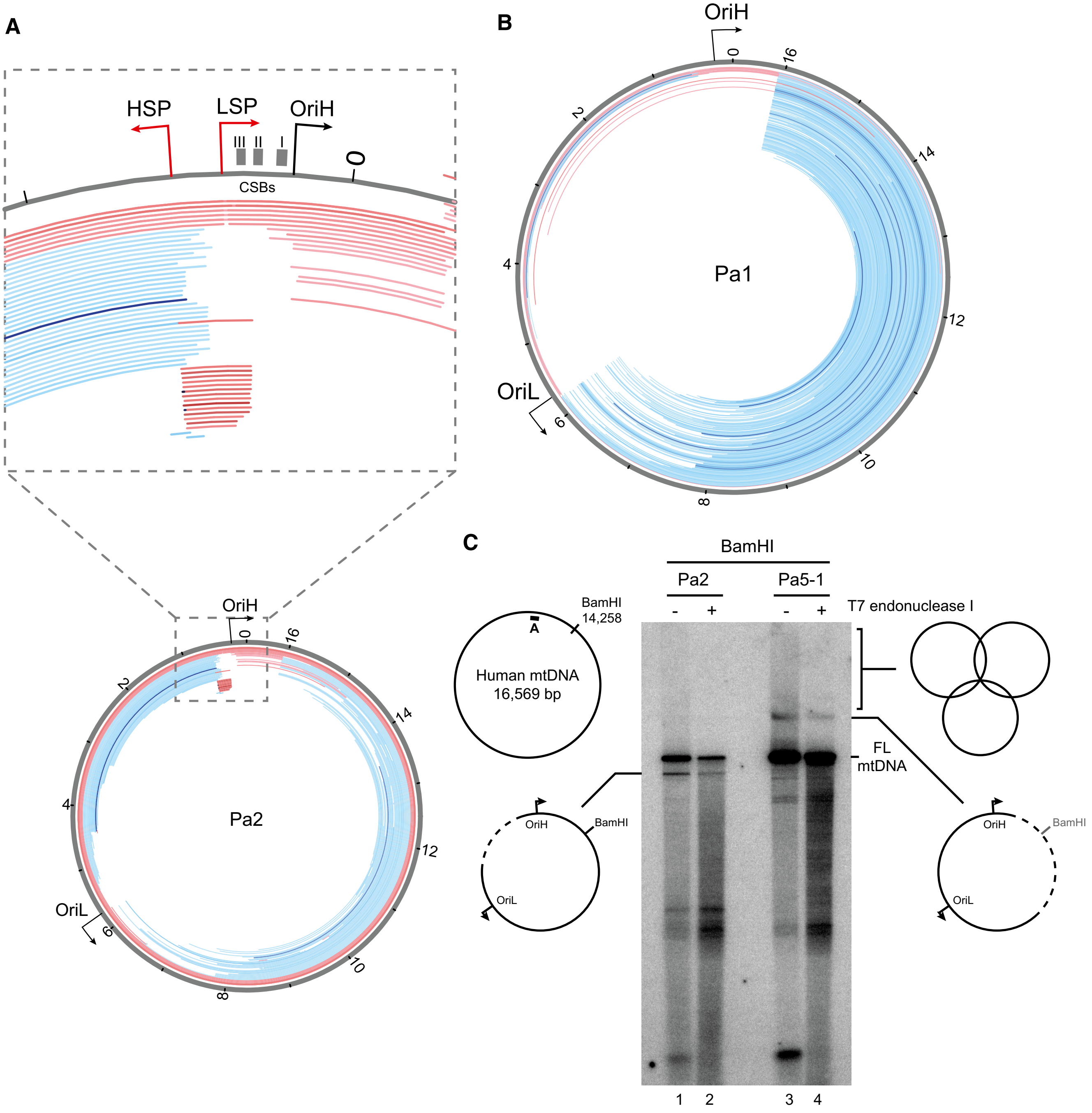

Figure EV3. mtDNA rearrangements in patient muscle.

A Enlargement of the NCR region in whole-genome sequence data of Pa2, indicating the locations of small mtDNA rearrangements. The locations of mtDNA *cis*-elements in this region are indicated (LSP, light-strand promoter; HSP, heavy-strand promoter; OriH, origin of heavy-strand replication; CSBs, conserved sequence blocks).

B Mitochondrial DNA rearrangements from Pa1 (Nicholls *et al*, 2018). Whole-genome sequencing data were analysed using the same version of the MitoSAlt pipeline as for newly reported individuals (Fig 2) to permit comparison of breakpoint mapping datasets. Deleted regions are shown as blue bars and predicted duplicated regions as red bars. The intensity of the colour corresponds to the abundance of the rearrangement.

C Analysis of mtDNA rearrangements in Pa2 and Pa5-1 using Southern blotting. Total muscle DNA (250 ng) was restricted with BamHI, and then left further untreated or incubated with 1 U of T7 endonuclease I, separated on agarose and Southern blotted using probe A (indicated with a black bar). Diagrams indicate the structures visible.

Source data are available online for this figure.

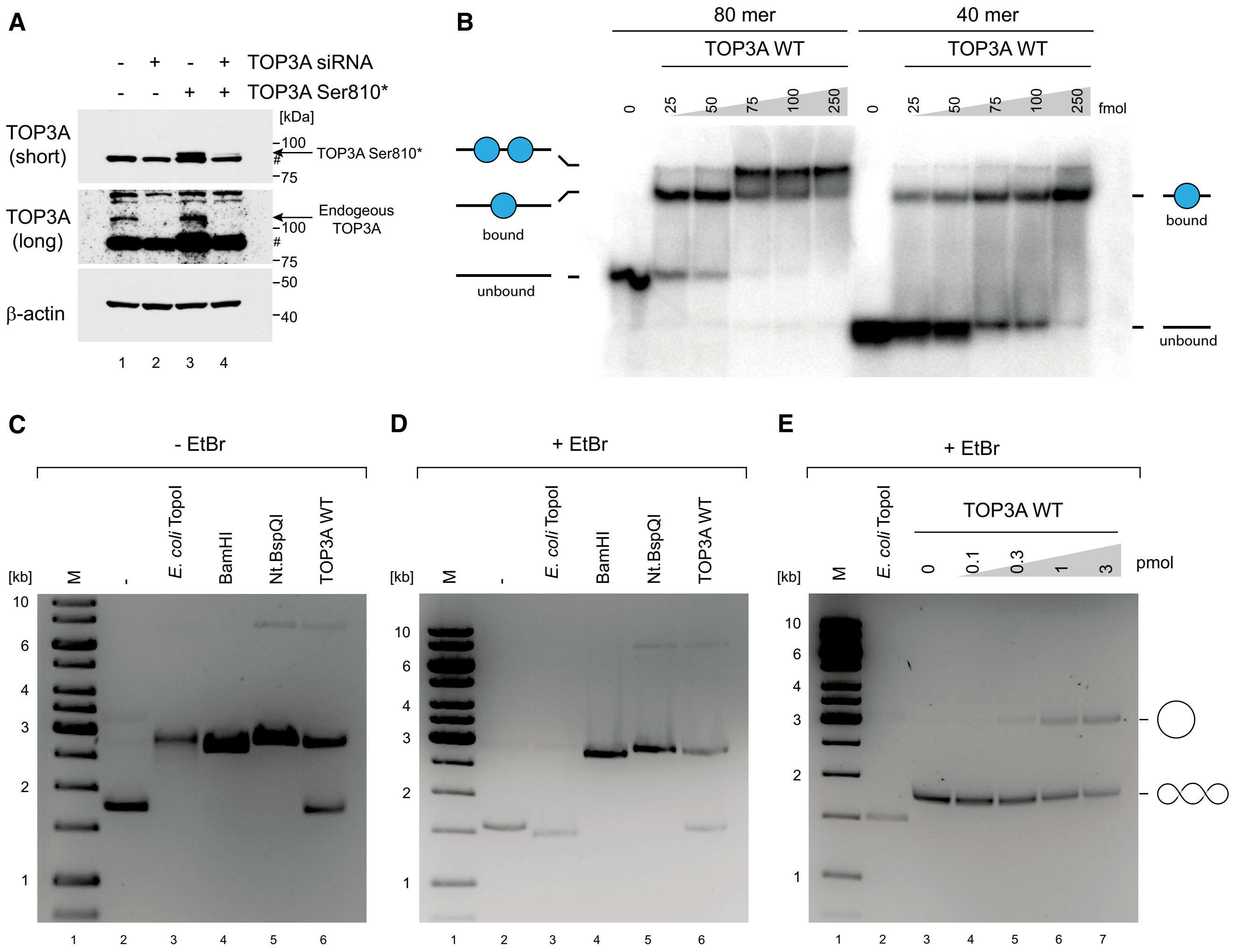

Figure EV4. Control reactions for EMSA and relaxation assays.

A Western blot of HeLa cells treated with TOP3A siRNA, with or without transient expression of TOP3A Ser810*. The band corresponding to TOP3A Ser810* migrates close to a non-specific band (indicated with #). β-actin is used as a loading control.

B Electrophoretic mobility shift assay (EMSA) using either 80-mer or 40-mer ssDNA substrates. Diagrams indicate singly and doubly shifted substrate.

C, D Controls for substrate migration in plasmid relaxation assays. Negatively supercoiled pUC19 plasmid DNA was either left untreated (lane 2) or treated with the indicated enzymes. *E. coli* TopoI-treated plasmid DNA (lane 3) shows the migration of covalently closed relaxed DNA, BamHI-treated DNA (lane 4) shows the migration of linearised plasmid and Nt.BspQI-treated DNA (lane 5) shows the migration of nicked circular plasmid. Reactions were separated either in the absence of ethidium bromide (EtBr) and post-stained for imaging (C) or run in the presence of EtBr (D). The migration of relaxed open, circular DNA and supercoiled DNA is indicated to the right of figure.

E Controls for TOP3A nicking activity. Negatively supercoiled pUC19 plasmid DNA was incubated with WT TOP3A protein as in Fig 5A, then separated on an agarose gel containing EtBr. The migration of nicked DNA and supercoiled DNA is indicated to the right of the figure. M" indicates marker.

Source data are available online for this figure.

© 2023 The Authors

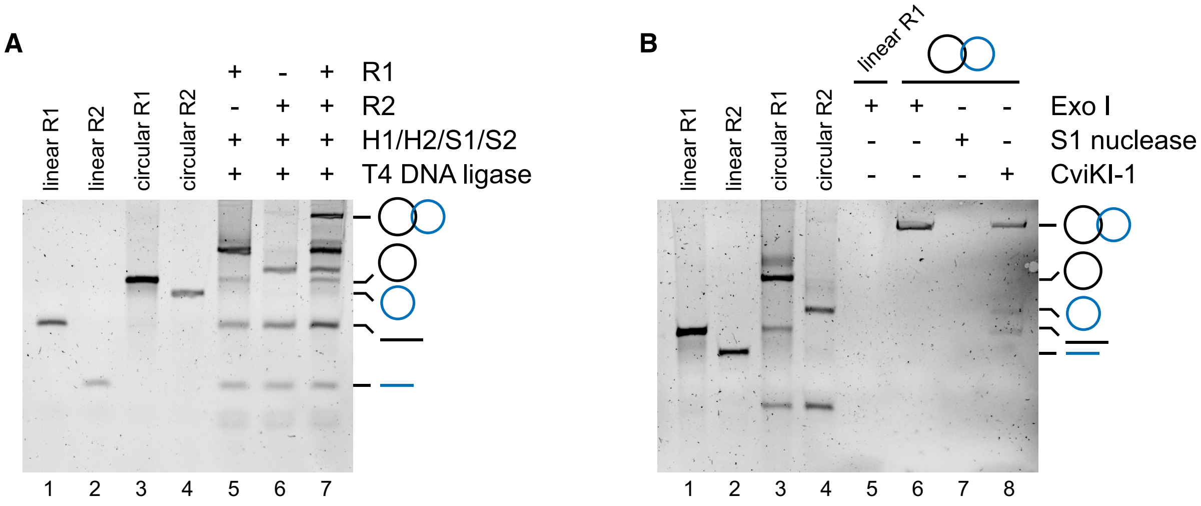

Figure EV5. ssDNA catenane substrate construction and validation.

A Synthesis of Lk1 ssDNA catenanes. Linear and circular R1 and R2 oligos are shown for size comparison (lanes 1–4). Products resulting from synthesis reactions omitting R2 (lane 5) or R1 (lane 6) are shown, as well as a complete synthesis reaction (lane 7). The migration of Lk1 ssDNA catenanes and circular and linear R1 and R2 oligos is indicated to the right of figure.

B Verification of Lk1 ssDNA catenanes. Linear and circular R1 and R2 oligos are shown for size comparison (lanes 1–4). The treatment of linear R1 oligo with ExoI (lane 5) is shown as a positive control for ExoI activity. Lk1 ssDNA catenanes were treated with ExoI (lane 6), S1 nuclease (lane 7) or CviKI-1 (lane 8). The migration of Lk1 ssDNA catenanes and circular and linear R1 and R2 oligos is indicated to the right of the figure.

Source data are available online for this figure.
