# Supplementary figures and images for: Pathological variants in TOP3A cause distinct disorders of mitochondrial and nuclear genome stability
Source: EMBO Mol Med. 2023 Apr 4;15(5):e16775. doi: 10.15252/emmm.202216775 (PMC10165364; doi:10.15252/emmm.202216775)

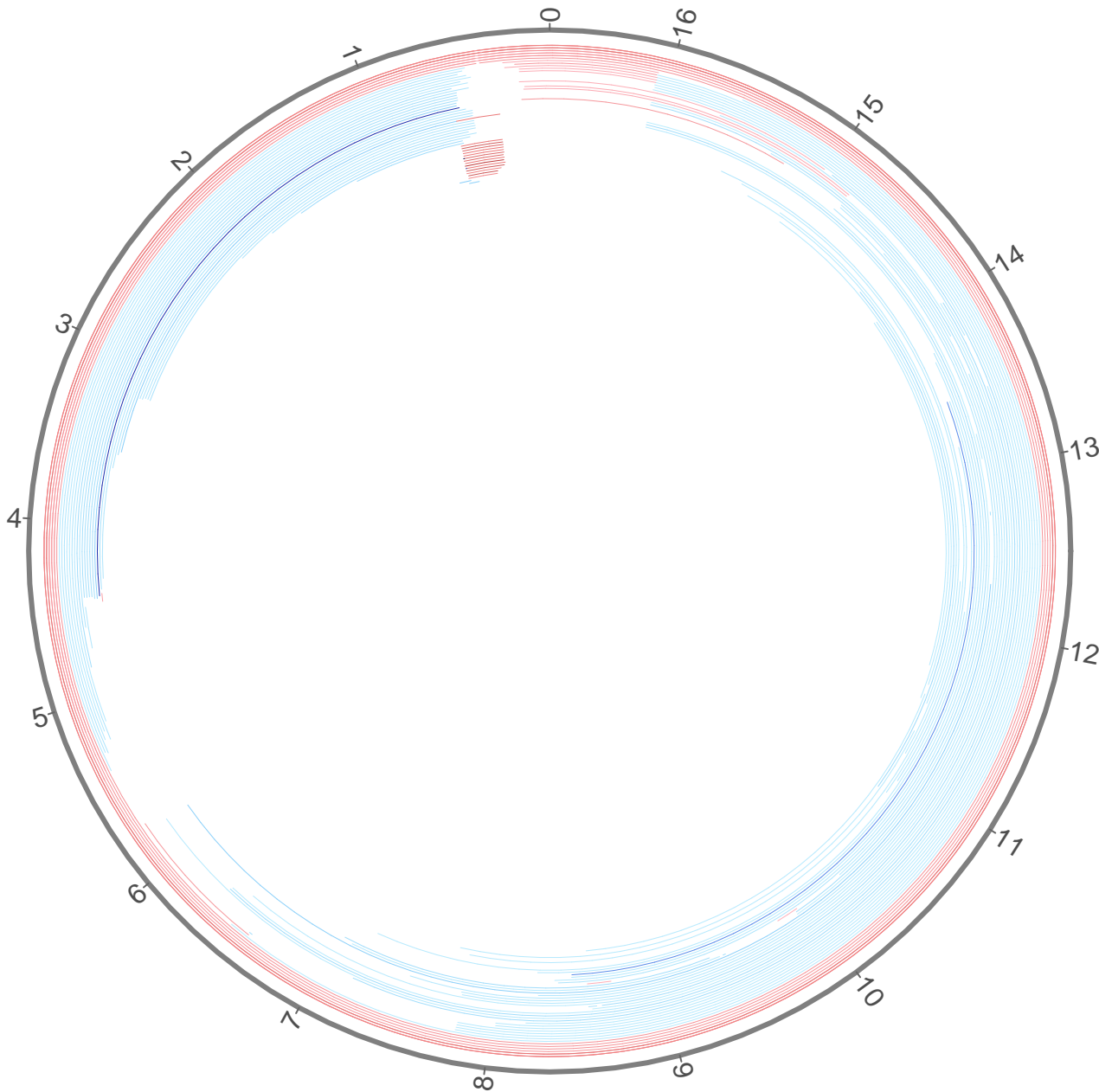

Supplement: Supplementary file 3 — Source Data for Expanded View [file EMMM-15-e16775-s005.zip › Figure_EV2/Figure_EV2A.pdf]

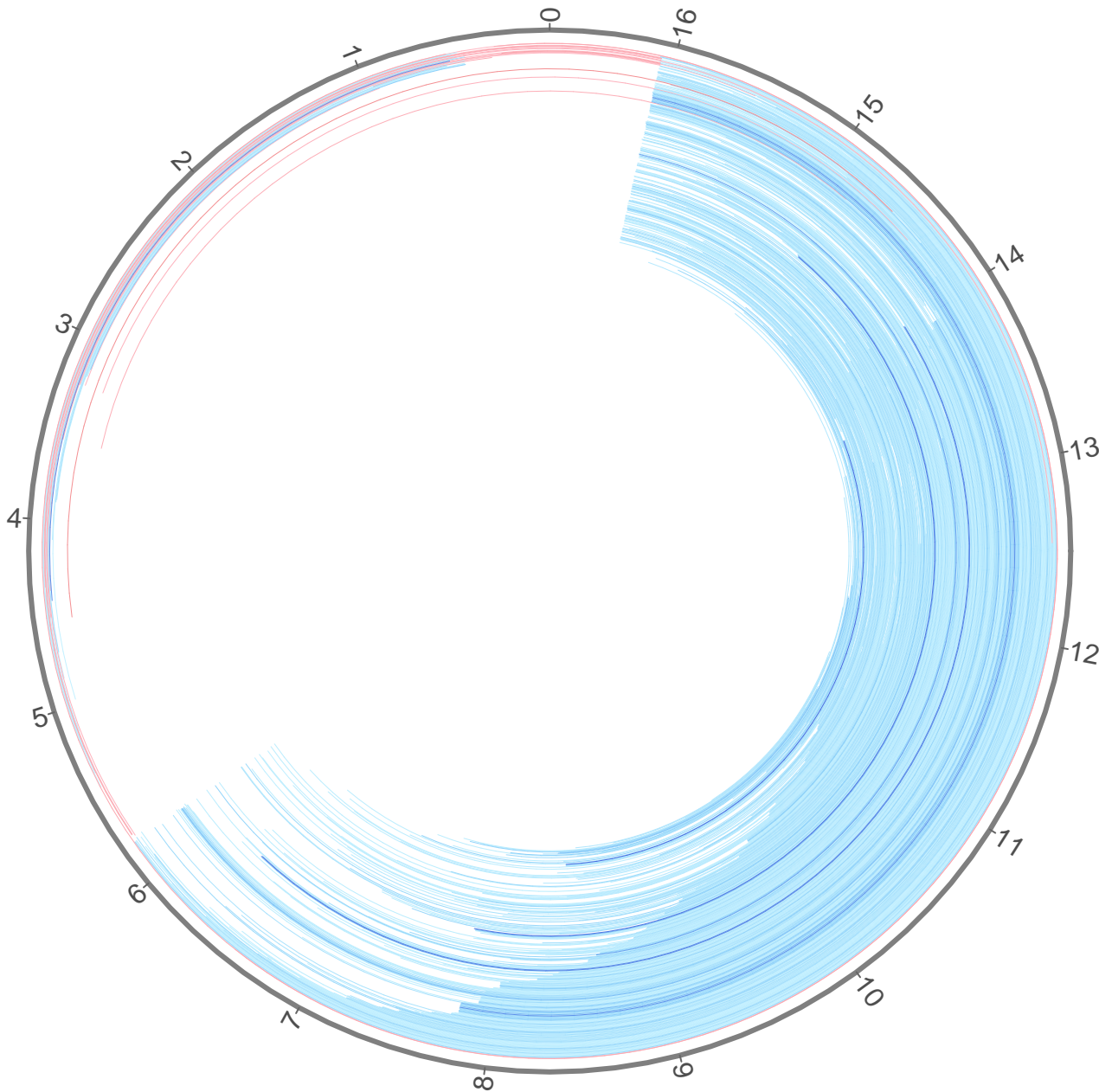

Supplement: Supplementary file 3 — Source Data for Expanded View [file EMMM-15-e16775-s005.zip › Figure_EV2/Figure_EV2B.pdf]

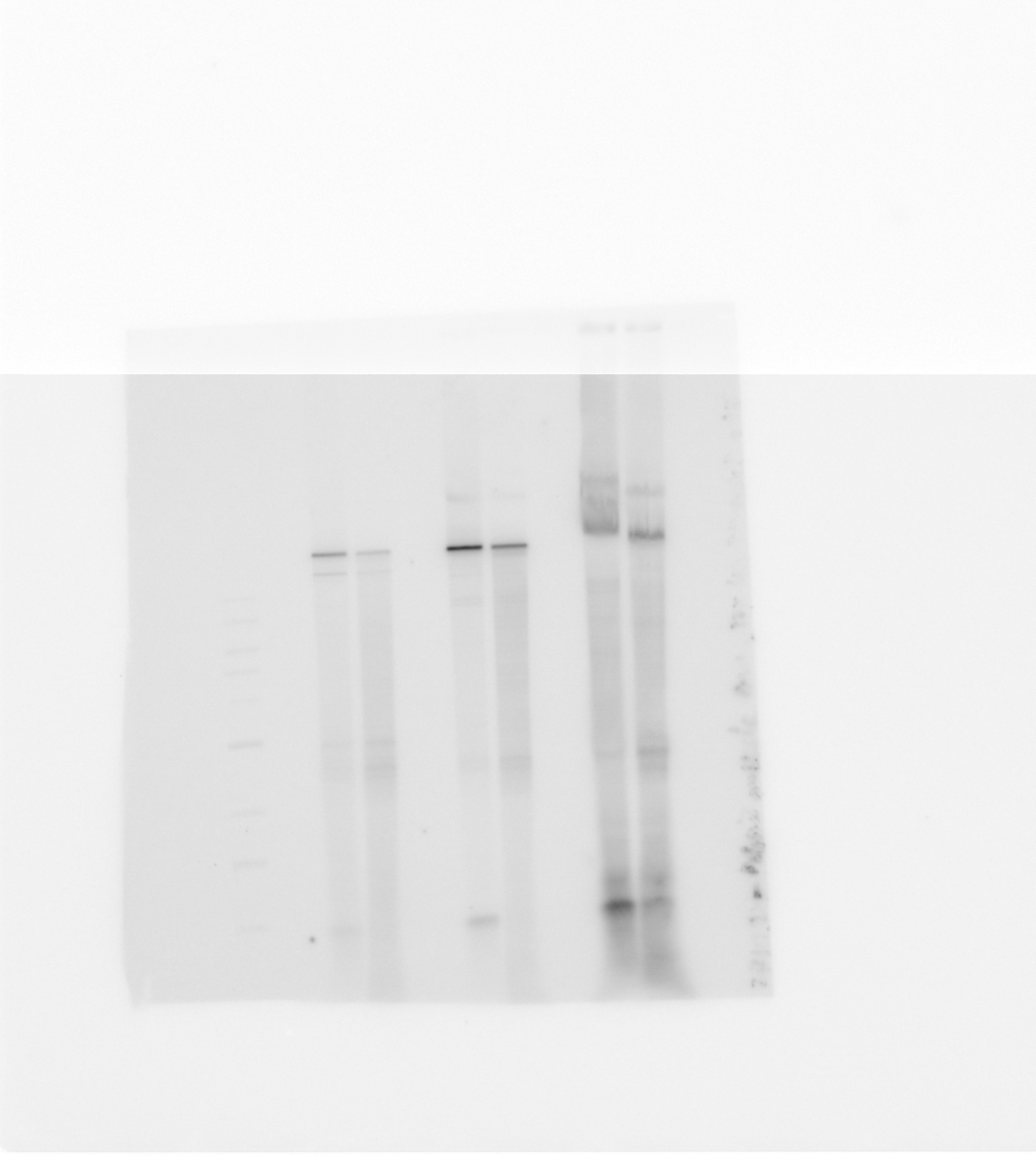

Supplement: Supplementary file 3 — Source Data for Expanded View [file EMMM-15-e16775-s005.zip › Figure_EV3/Figure_EV3C.tif]

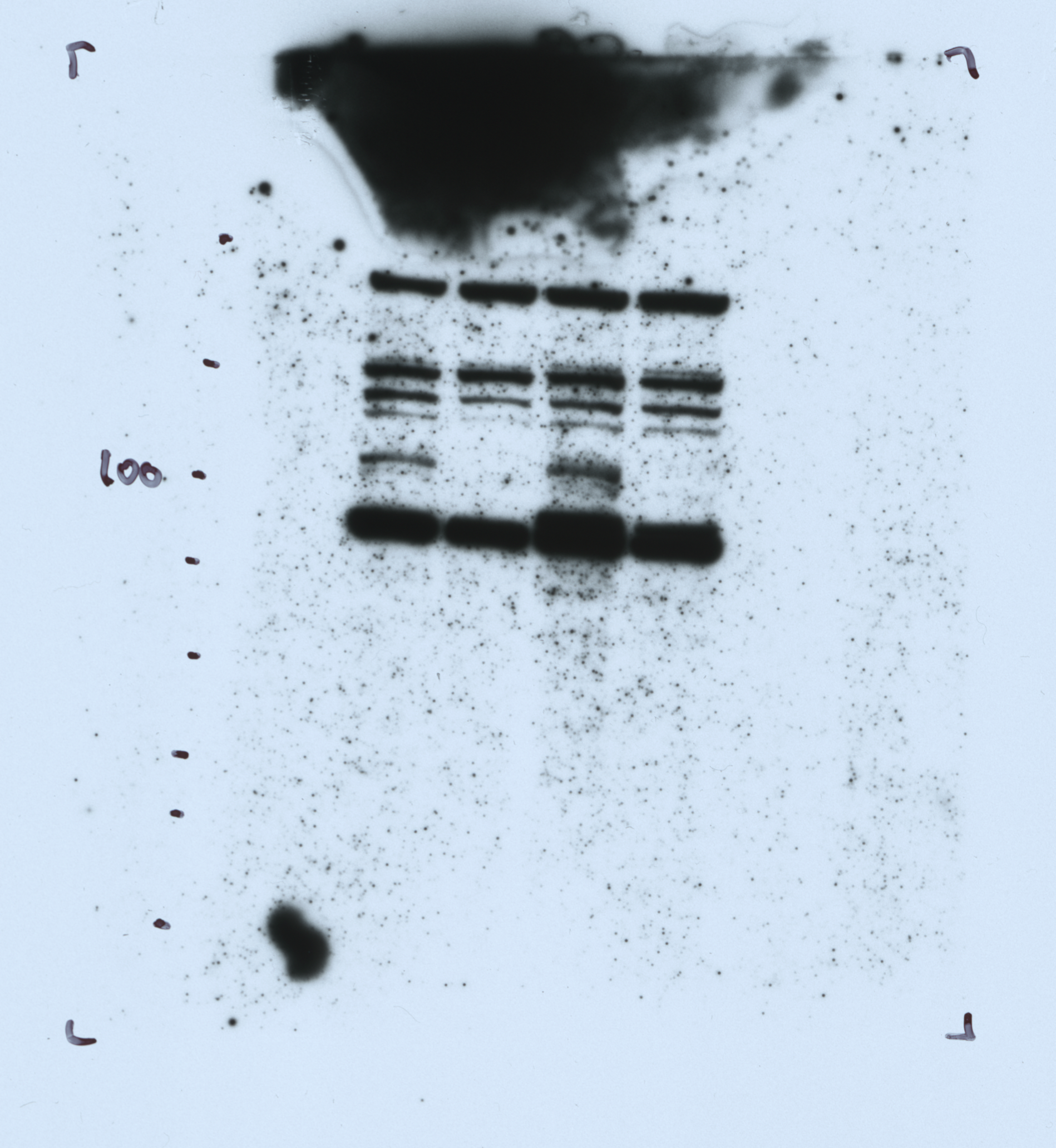

Supplement: Supplementary file 3 — Source Data for Expanded View [file EMMM-15-e16775-s005.zip › Figure_EV4/Figure_EV4A_TOP3A_long_exp.tif]

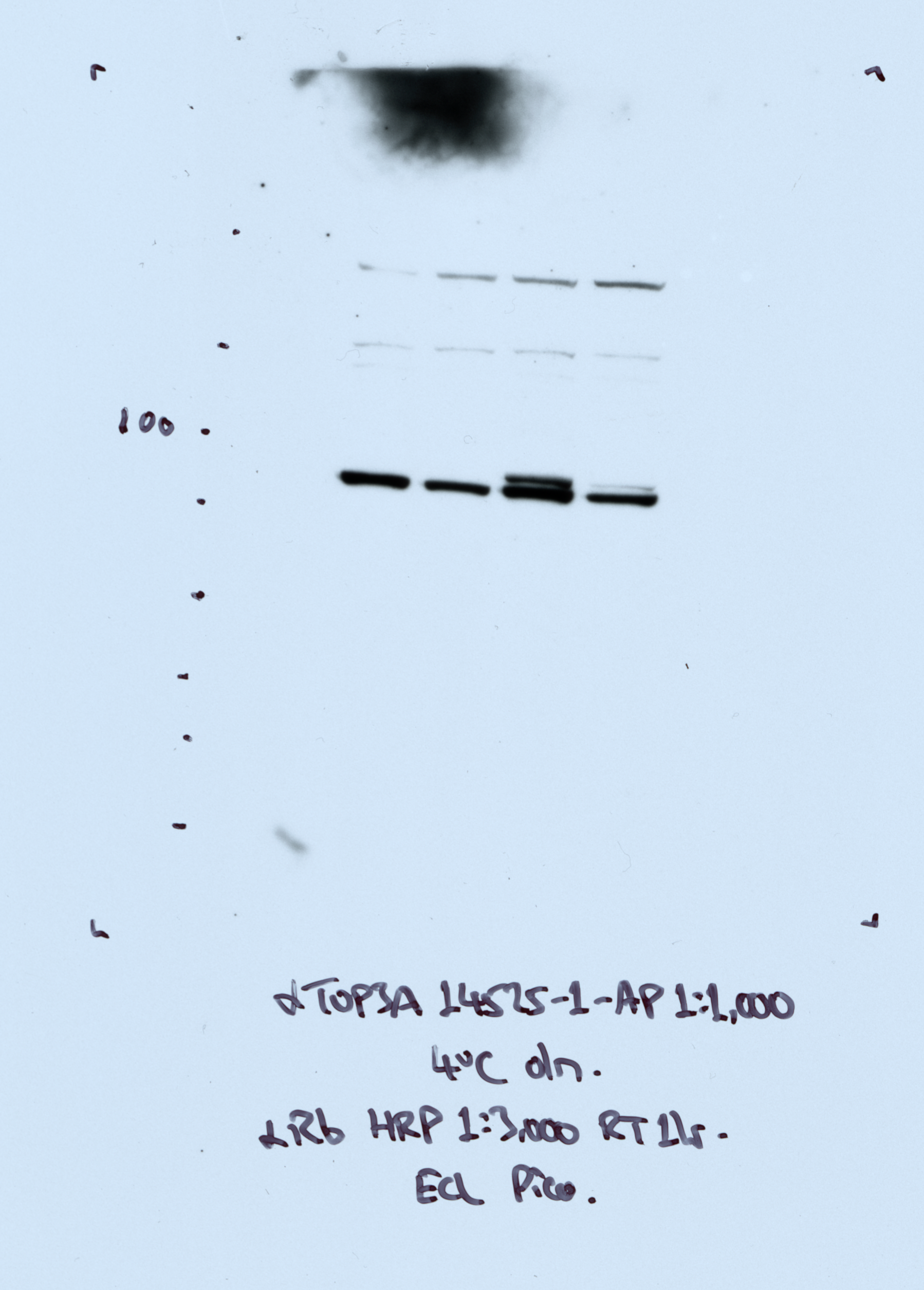

Supplement: Supplementary file 3 — Source Data for Expanded View [file EMMM-15-e16775-s005.zip › Figure_EV4/Figure_EV4A_TOP3A_short_exp.tif]

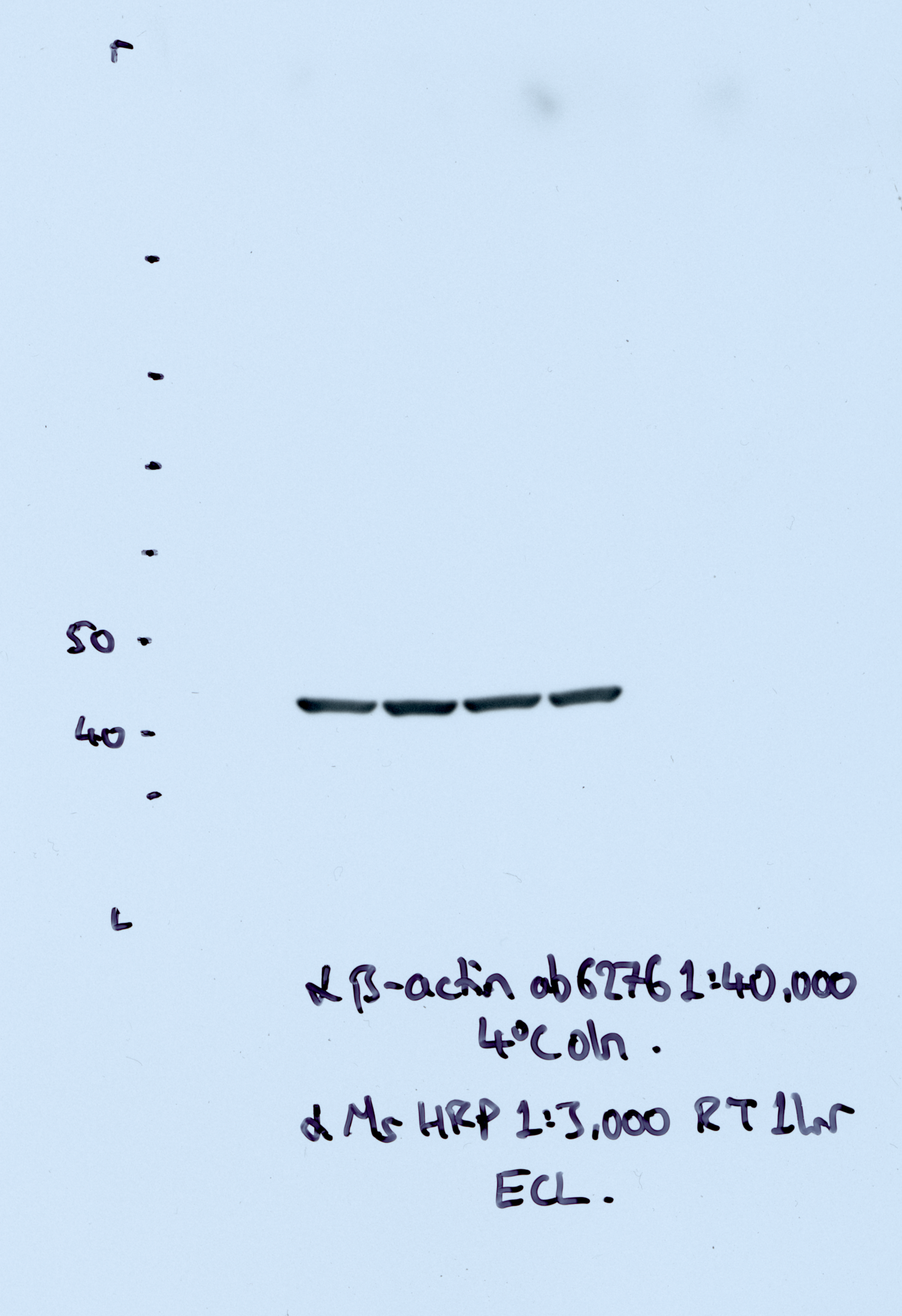

Supplement: Supplementary file 3 — Source Data for Expanded View [file EMMM-15-e16775-s005.zip › Figure_EV4/Figure_EV4A_actin.tif]

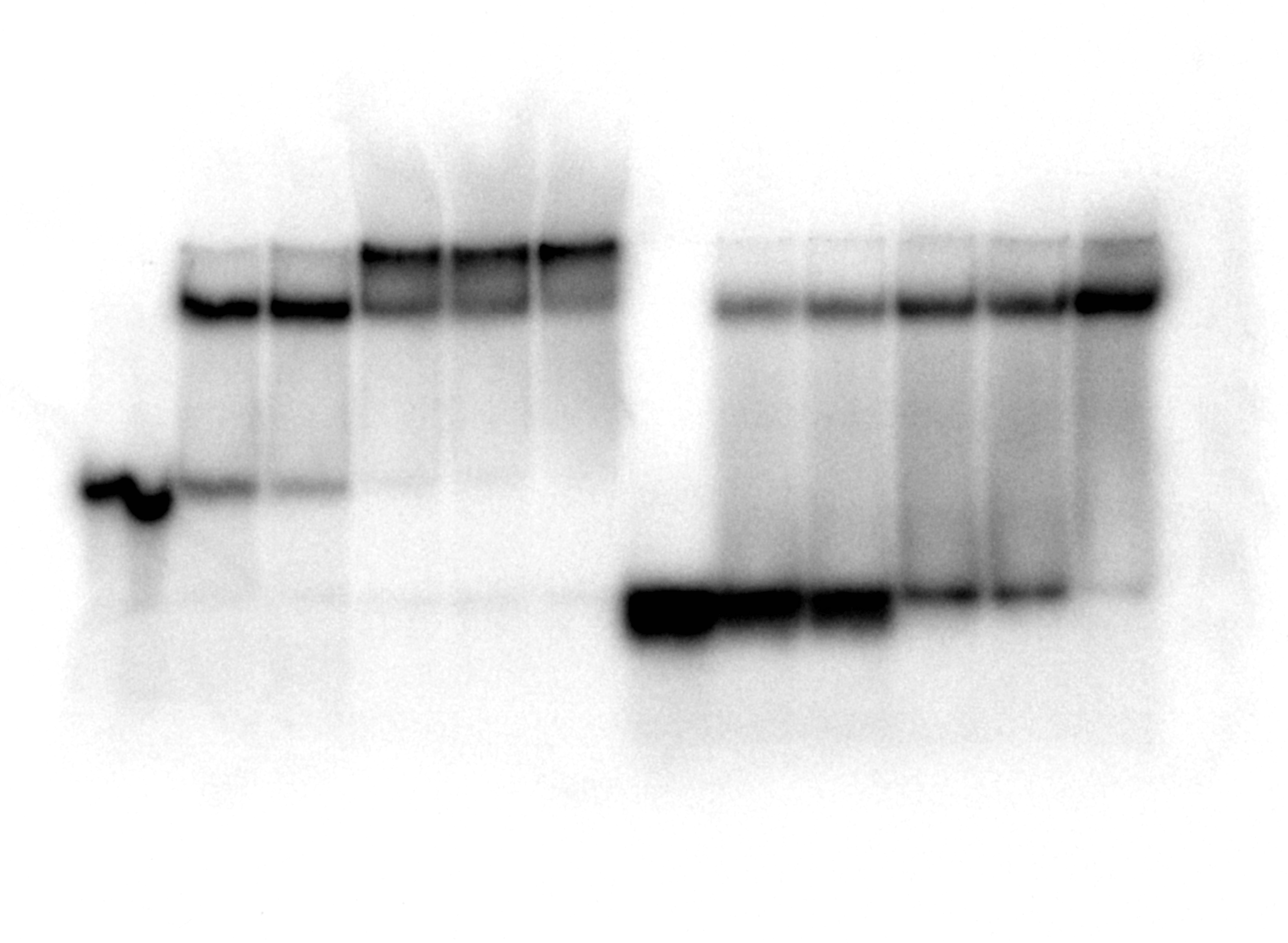

Supplement: Supplementary file 3 — Source Data for Expanded View [file EMMM-15-e16775-s005.zip › Figure_EV4/Figure_EV4B.tif]

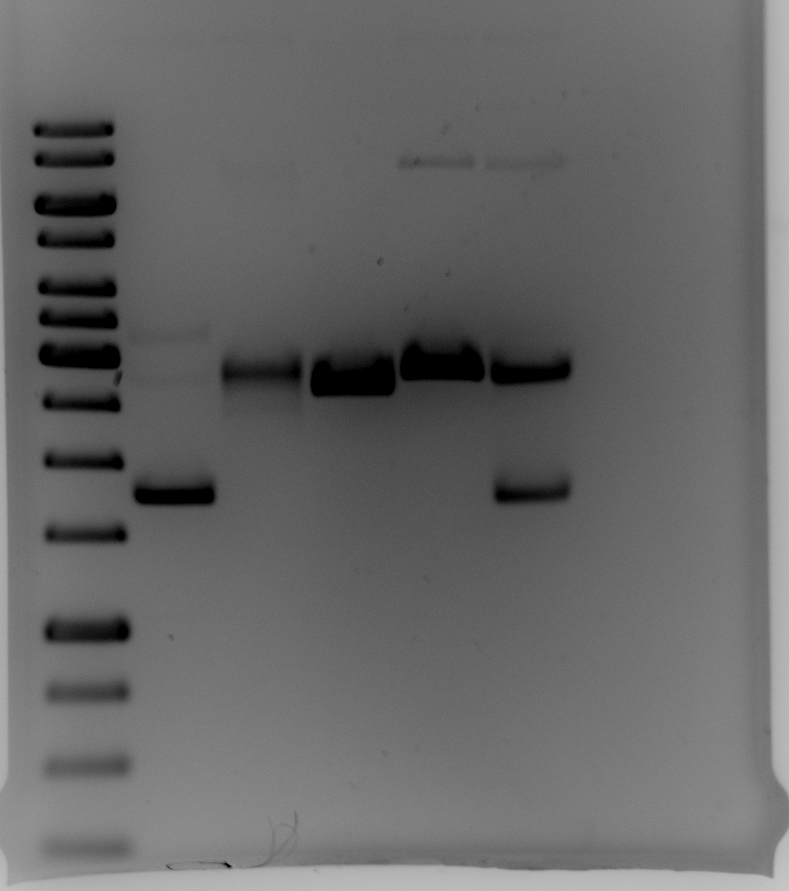

Supplement: Supplementary file 3 — Source Data for Expanded View [file EMMM-15-e16775-s005.zip › Figure_EV4/Figure_EV4C.tif]

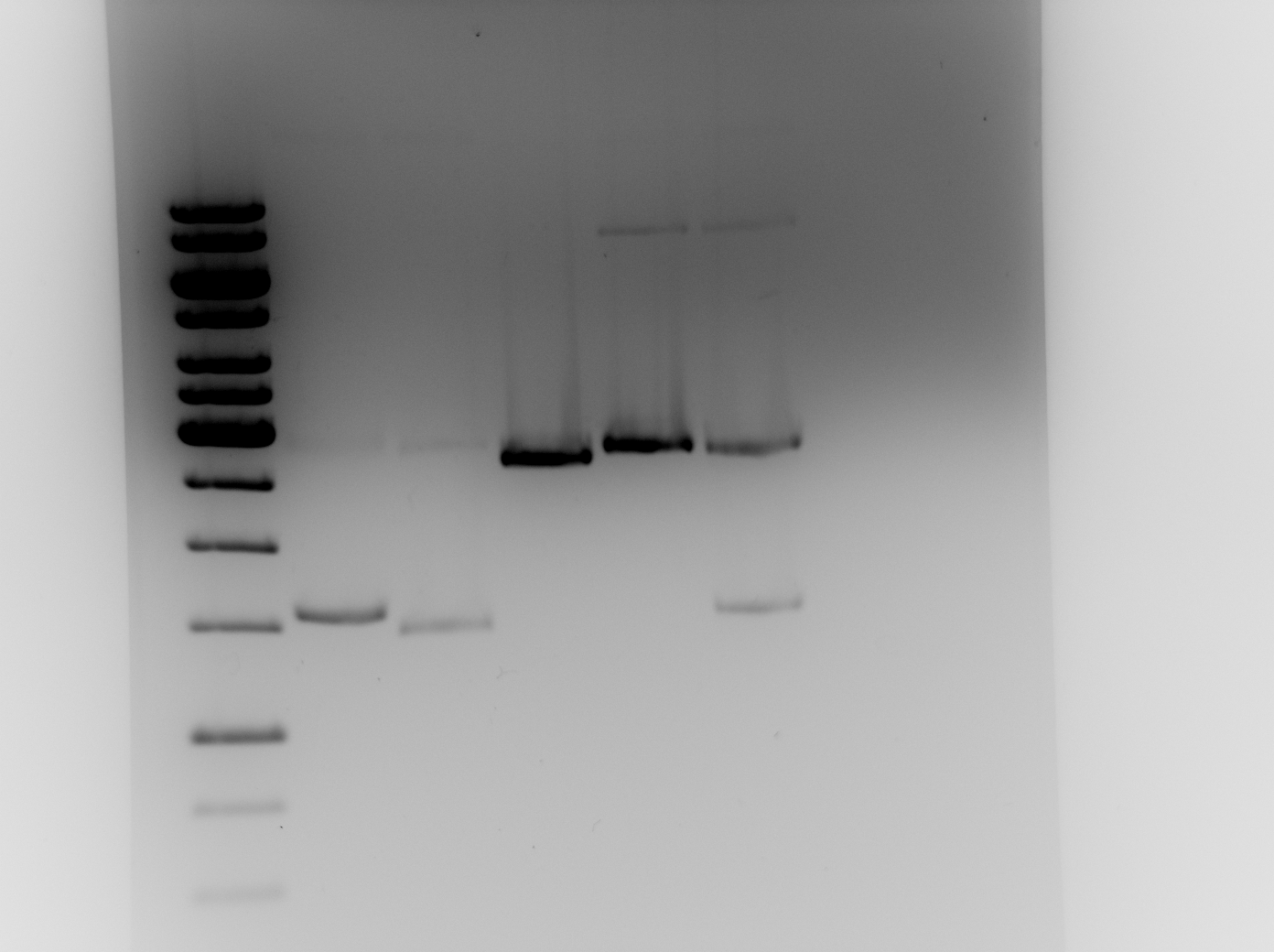

Supplement: Supplementary file 3 — Source Data for Expanded View [file EMMM-15-e16775-s005.zip › Figure_EV4/Figure_EV4D.tif]

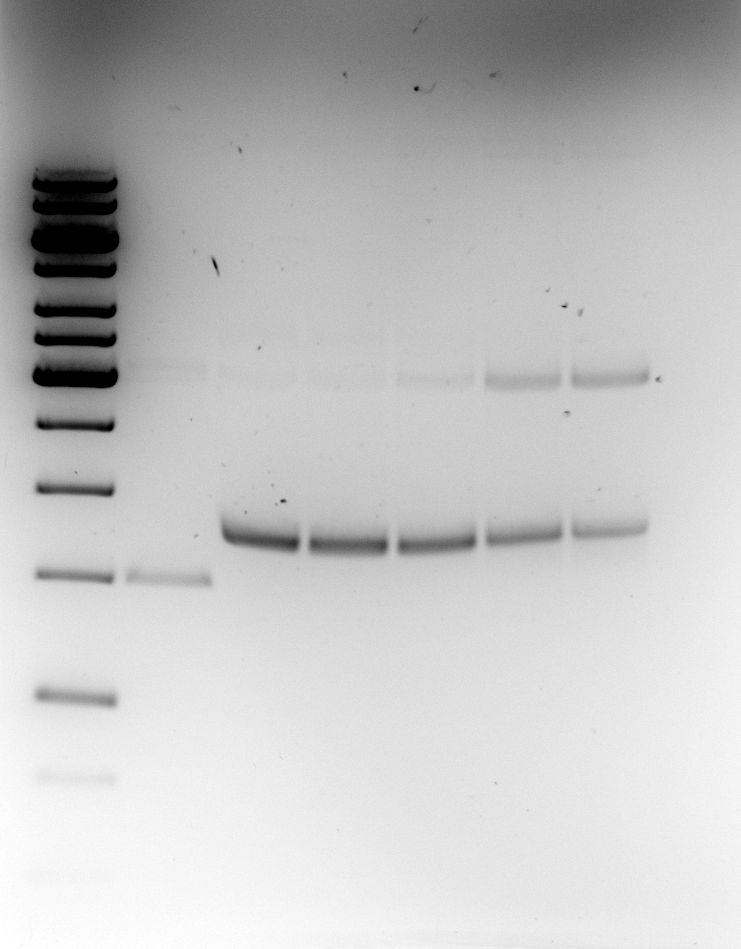

Supplement: Supplementary file 3 — Source Data for Expanded View [file EMMM-15-e16775-s005.zip › Figure_EV4/Figure_EV4E.tif]

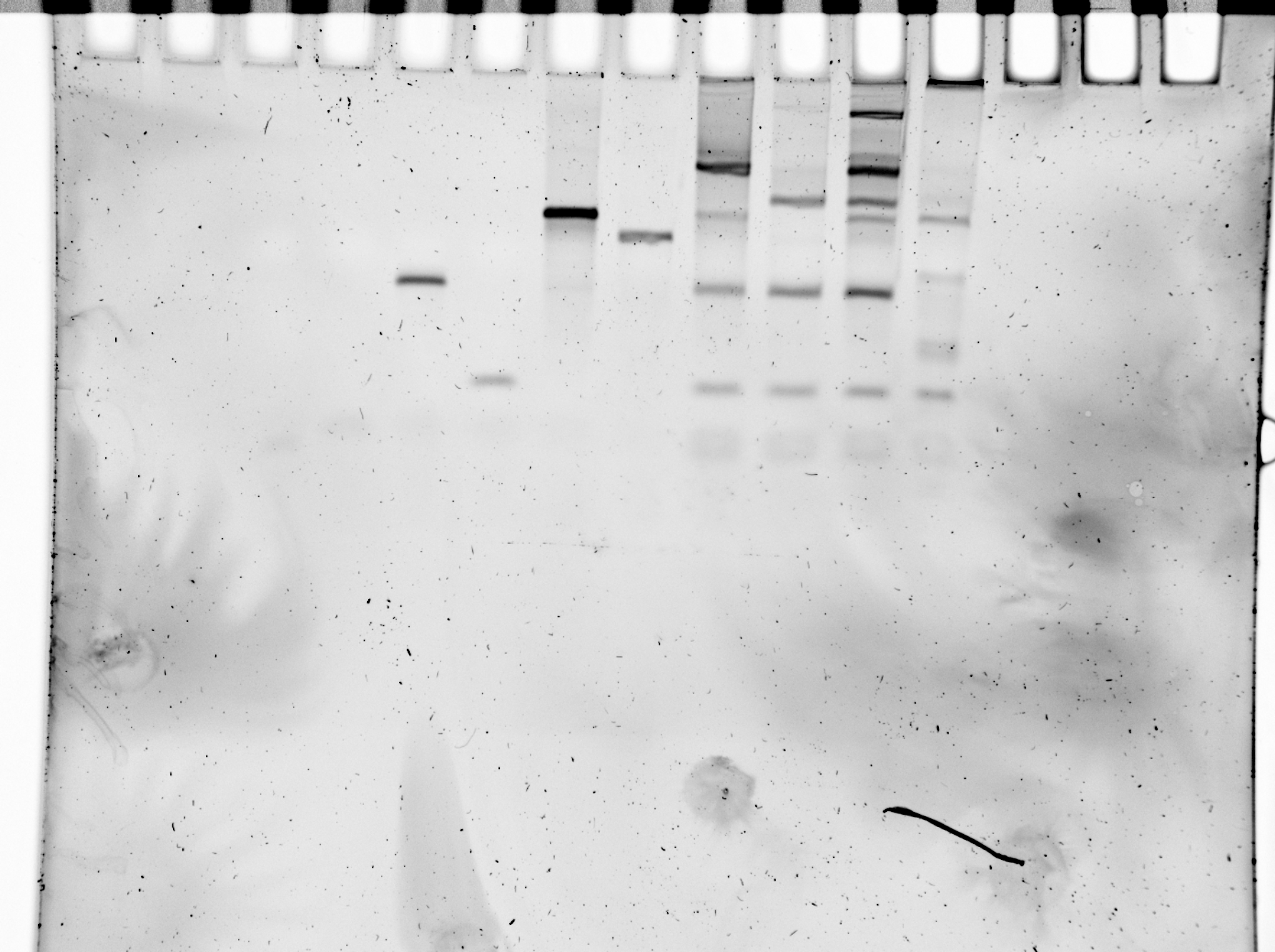

Supplement: Supplementary file 3 — Source Data for Expanded View [file EMMM-15-e16775-s005.zip › Figure_EV5/Figure_EV5A.tif]

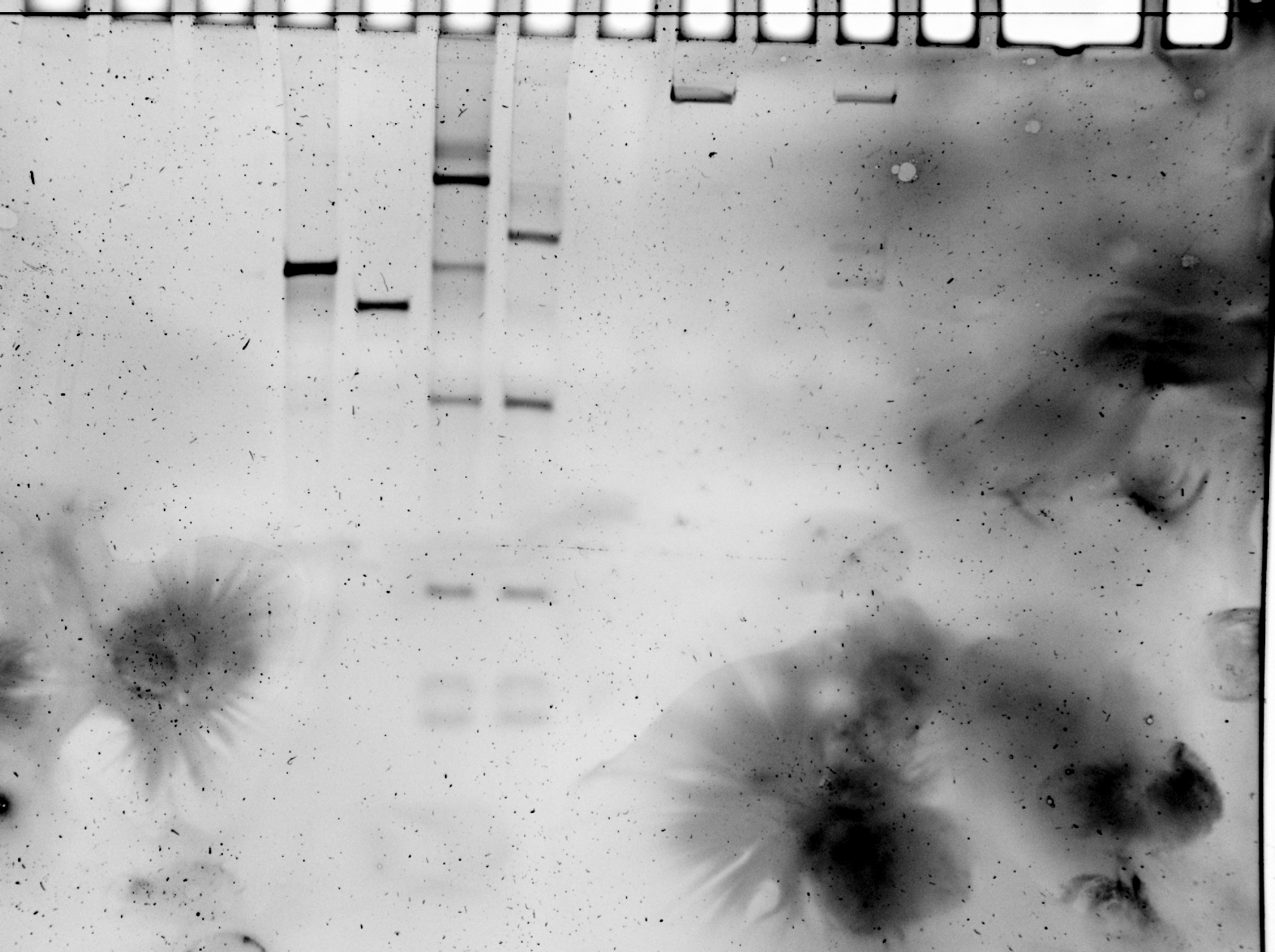

Supplement: Supplementary file 3 — Source Data for Expanded View [file EMMM-15-e16775-s005.zip › Figure_EV5/Figure_EV5B.tif]

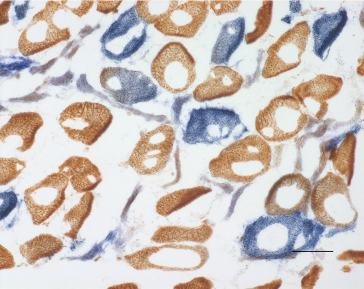

Supplement: Supplementary file 5 — Source Data for Figure 2 [file EMMM-15-e16775-s007.zip › Figure_2/Figure_2A_P2.png]

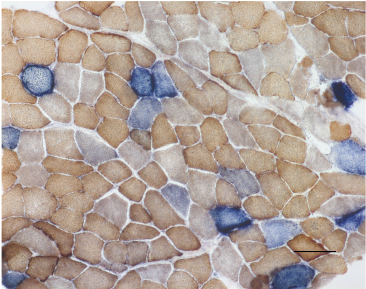

Supplement: Supplementary file 5 — Source Data for Figure 2 [file EMMM-15-e16775-s007.zip › Figure_2/Figure_2A_P6.png]

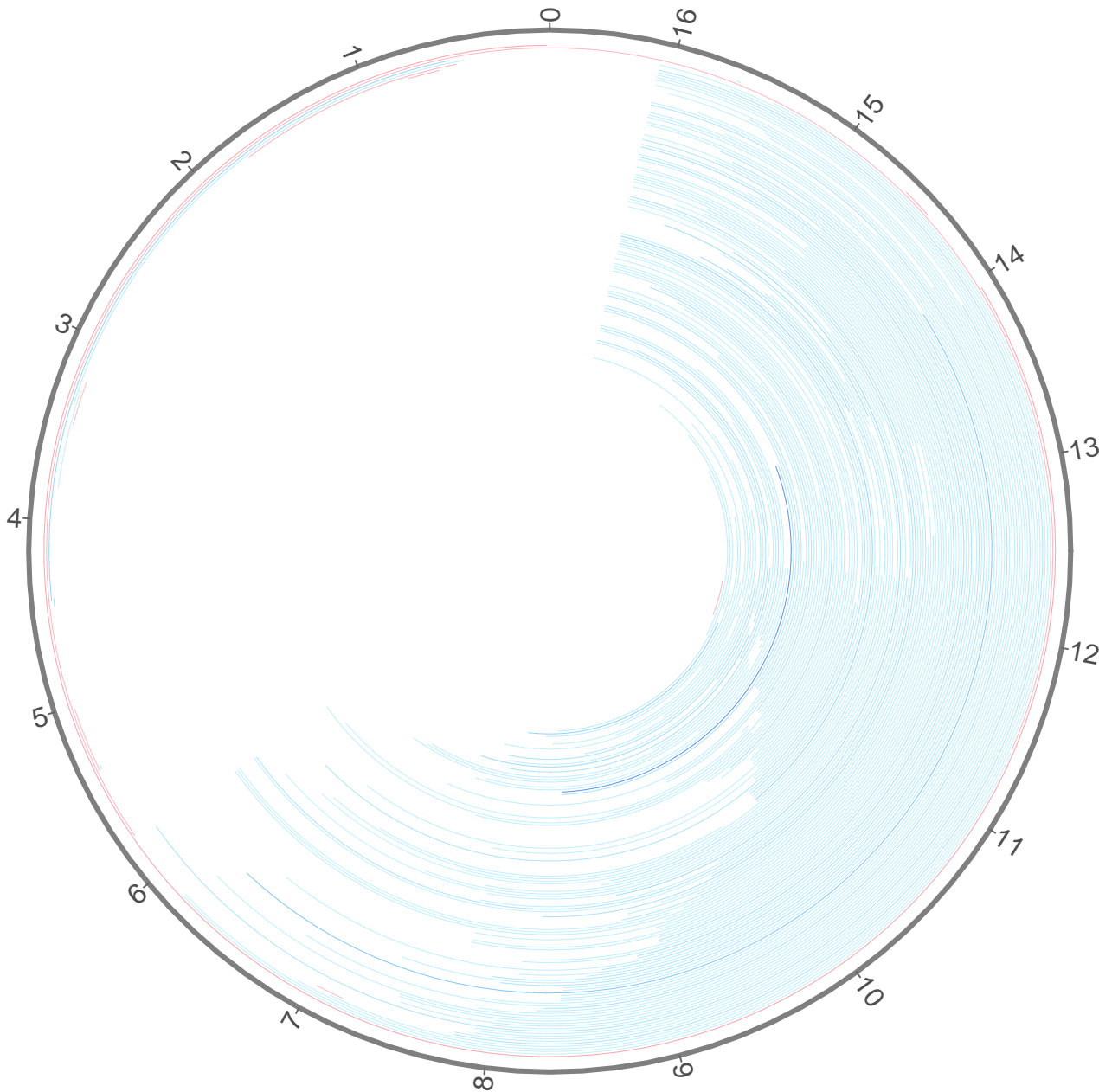

Supplement: Supplementary file 5 — Source Data for Figure 2 [file EMMM-15-e16775-s007.zip › Figure_2/Figure_2E.pdf]

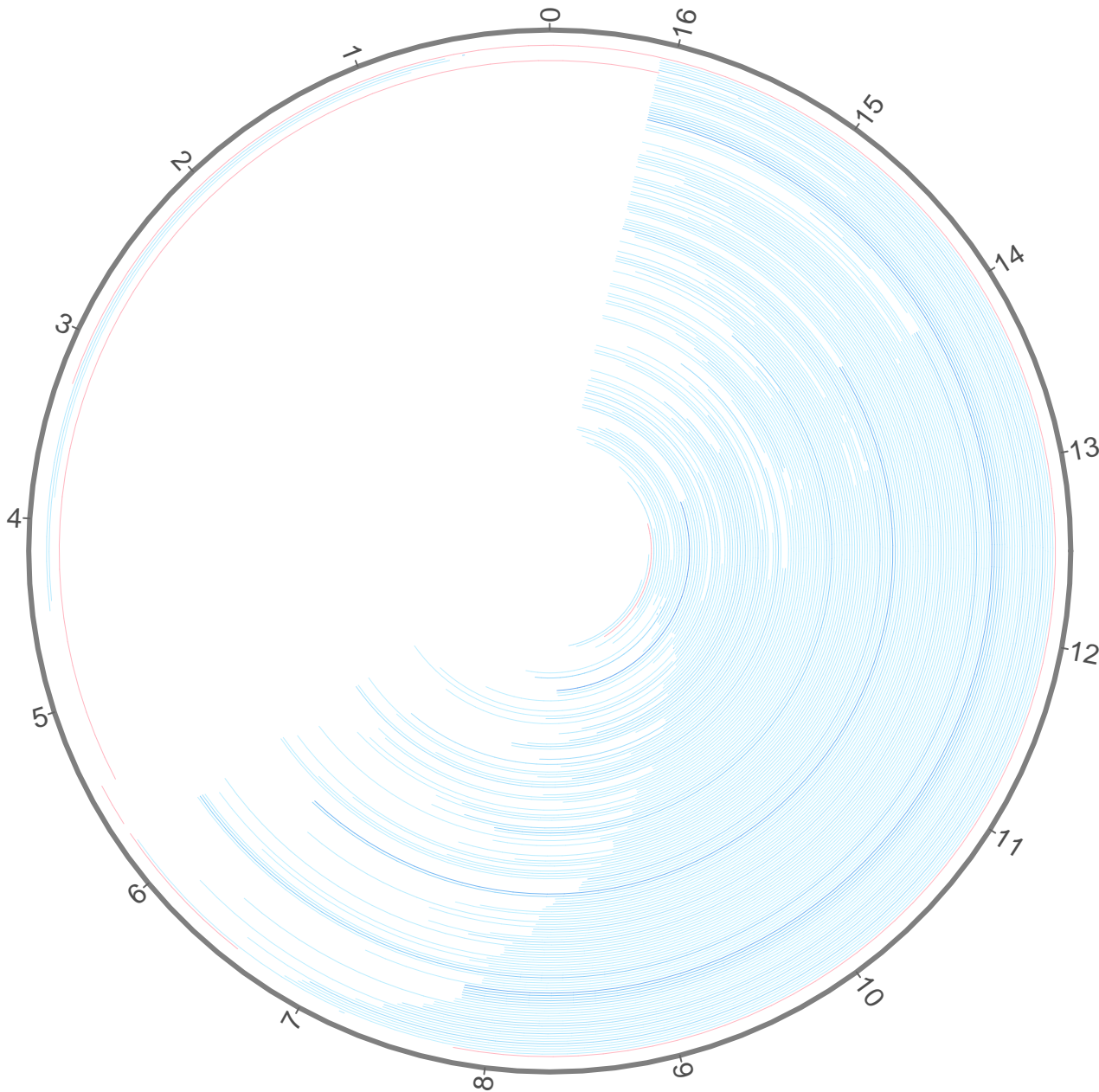

Supplement: Supplementary file 5 — Source Data for Figure 2 [file EMMM-15-e16775-s007.zip › Figure_2/Figure_2G.pdf]

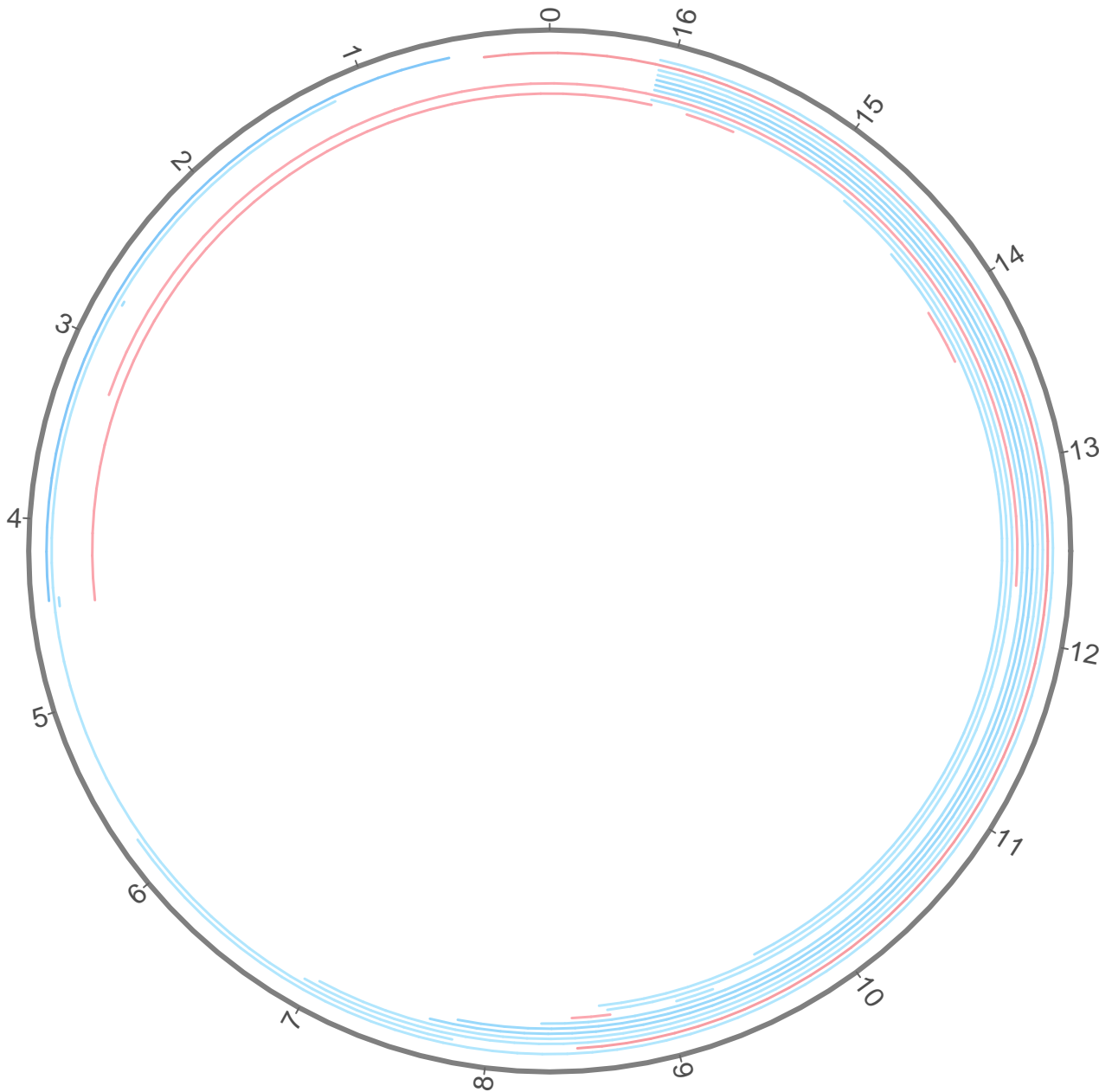

Supplement: Supplementary file 5 — Source Data for Figure 2 [file EMMM-15-e16775-s007.zip › Figure_2/Figure_2F.pdf]

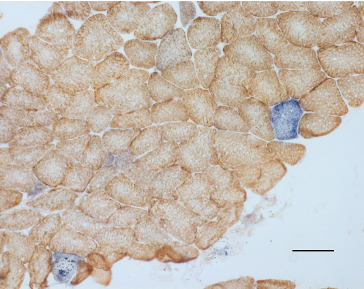

Supplement: Supplementary file 5 — Source Data for Figure 2 [file EMMM-15-e16775-s007.zip › Figure_2/Figure_2A_P4.png]

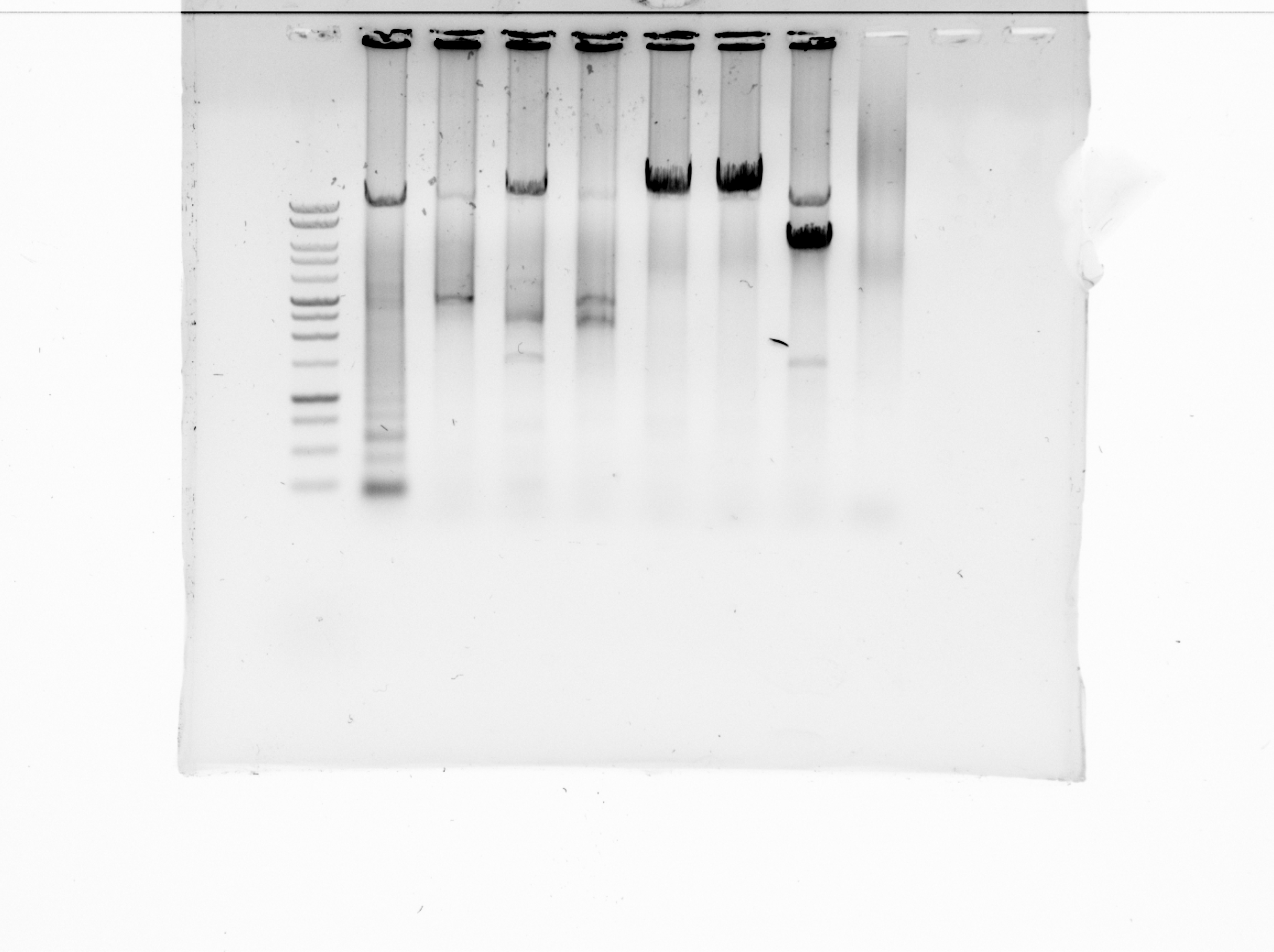

Supplement: Supplementary file 5 — Source Data for Figure 2 [file EMMM-15-e16775-s007.zip › Figure_2/Figure_2C.tif]

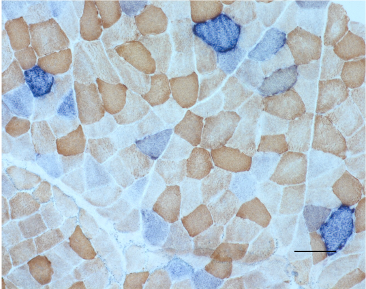

Supplement: Supplementary file 5 — Source Data for Figure 2 [file EMMM-15-e16775-s007.zip › Figure_2/Figure_2A_P3-1.png]

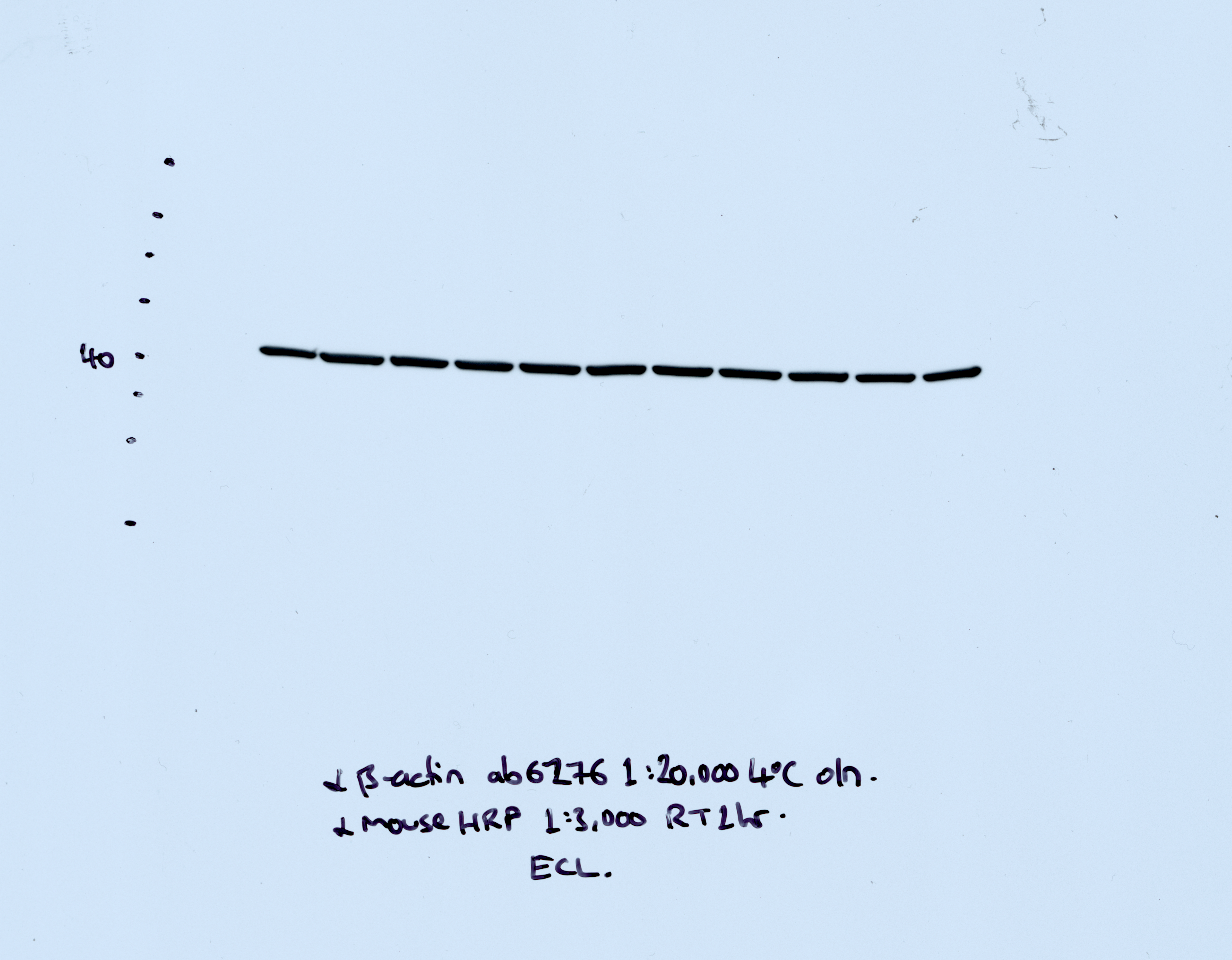

Supplement: Supplementary file 6 — Source Data for Figure 3 [file EMMM-15-e16775-s004.zip › Figure_3/Figure_3C_beta_actin.tif]

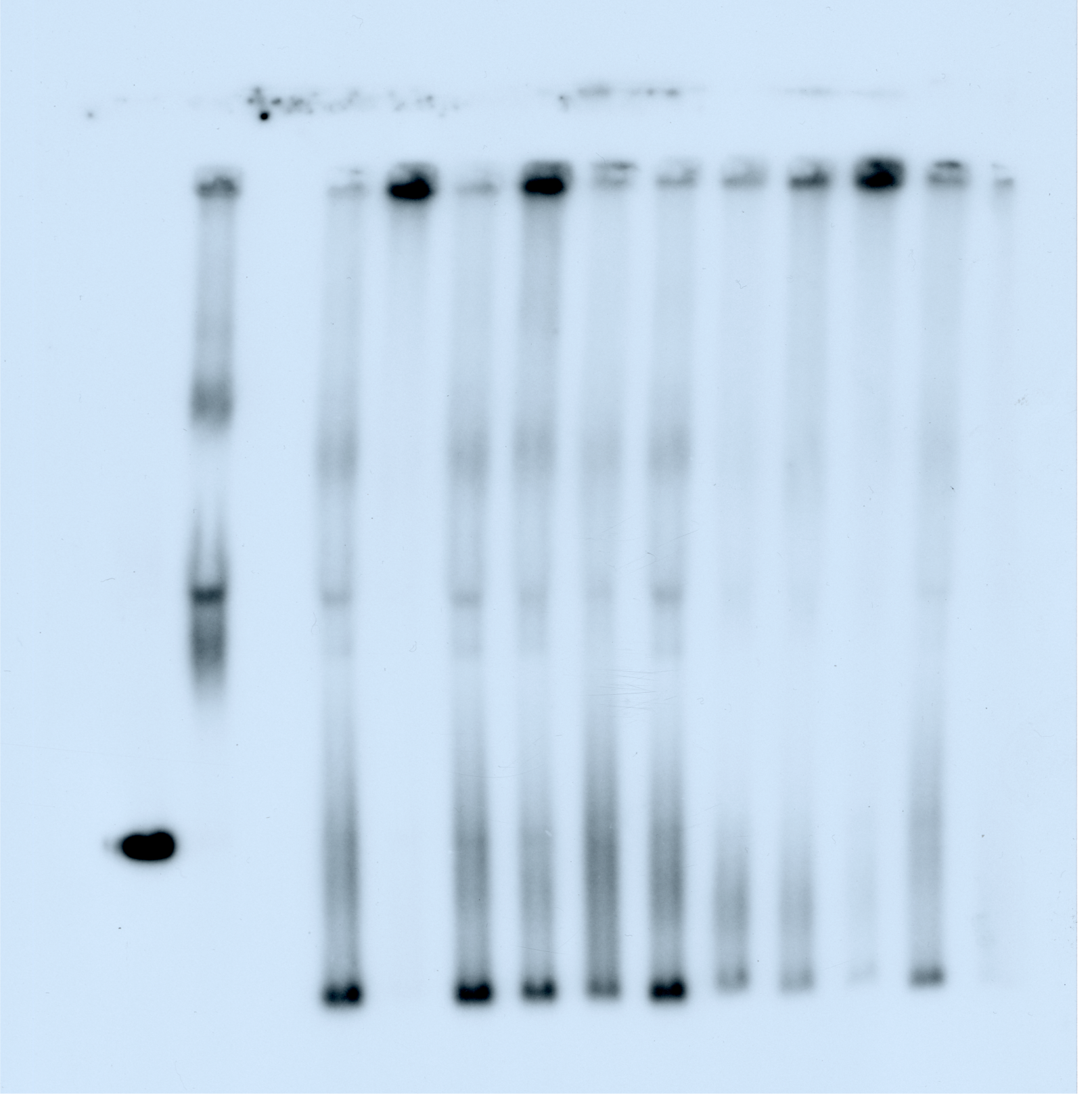

Supplement: Supplementary file 6 — Source Data for Figure 3 [file EMMM-15-e16775-s004.zip › Figure_3/Figure_3E.tif]

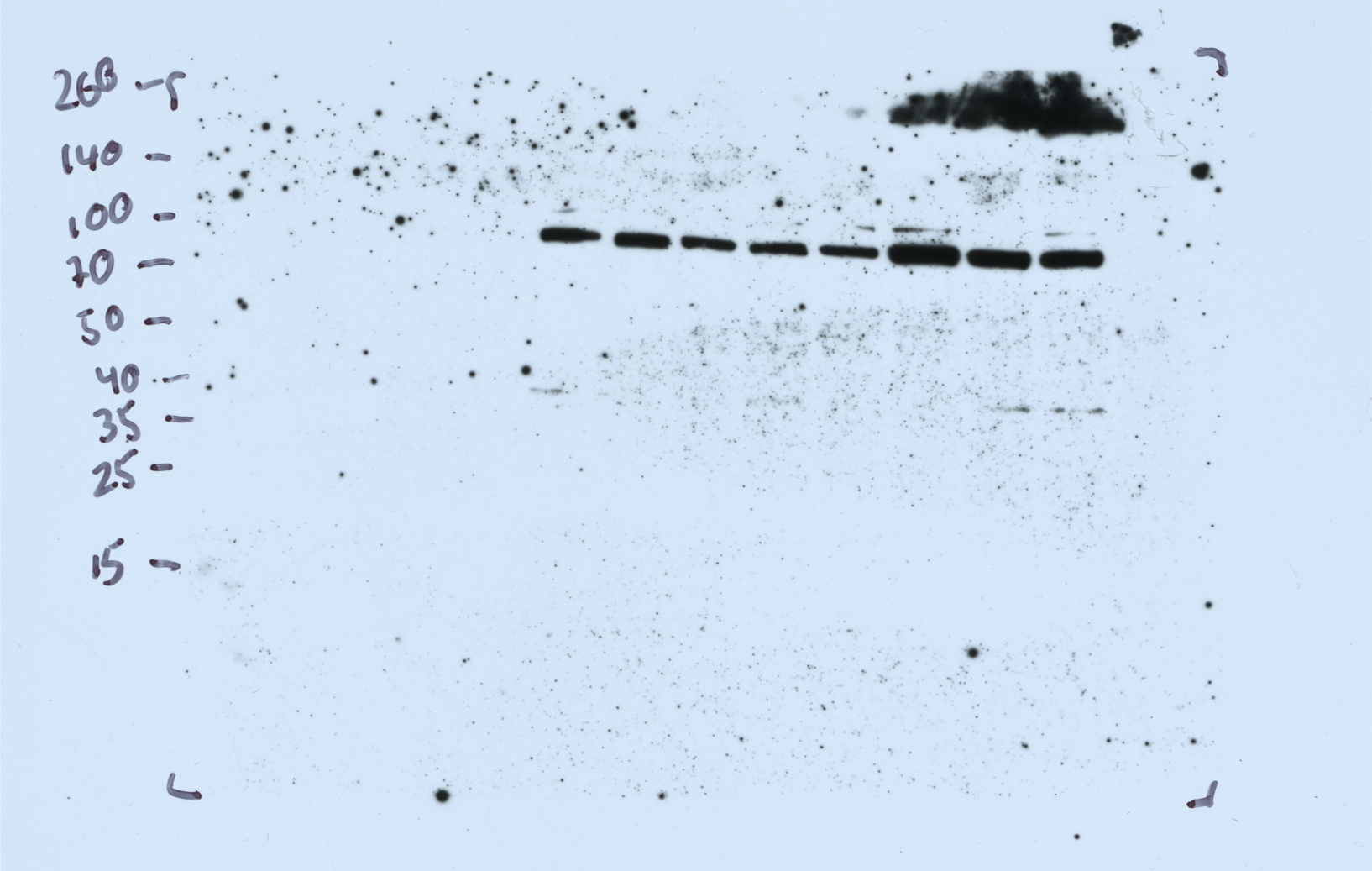

Supplement: Supplementary file 6 — Source Data for Figure 3 [file EMMM-15-e16775-s004.zip › Figure_3/Figure_3C_HA.tif]

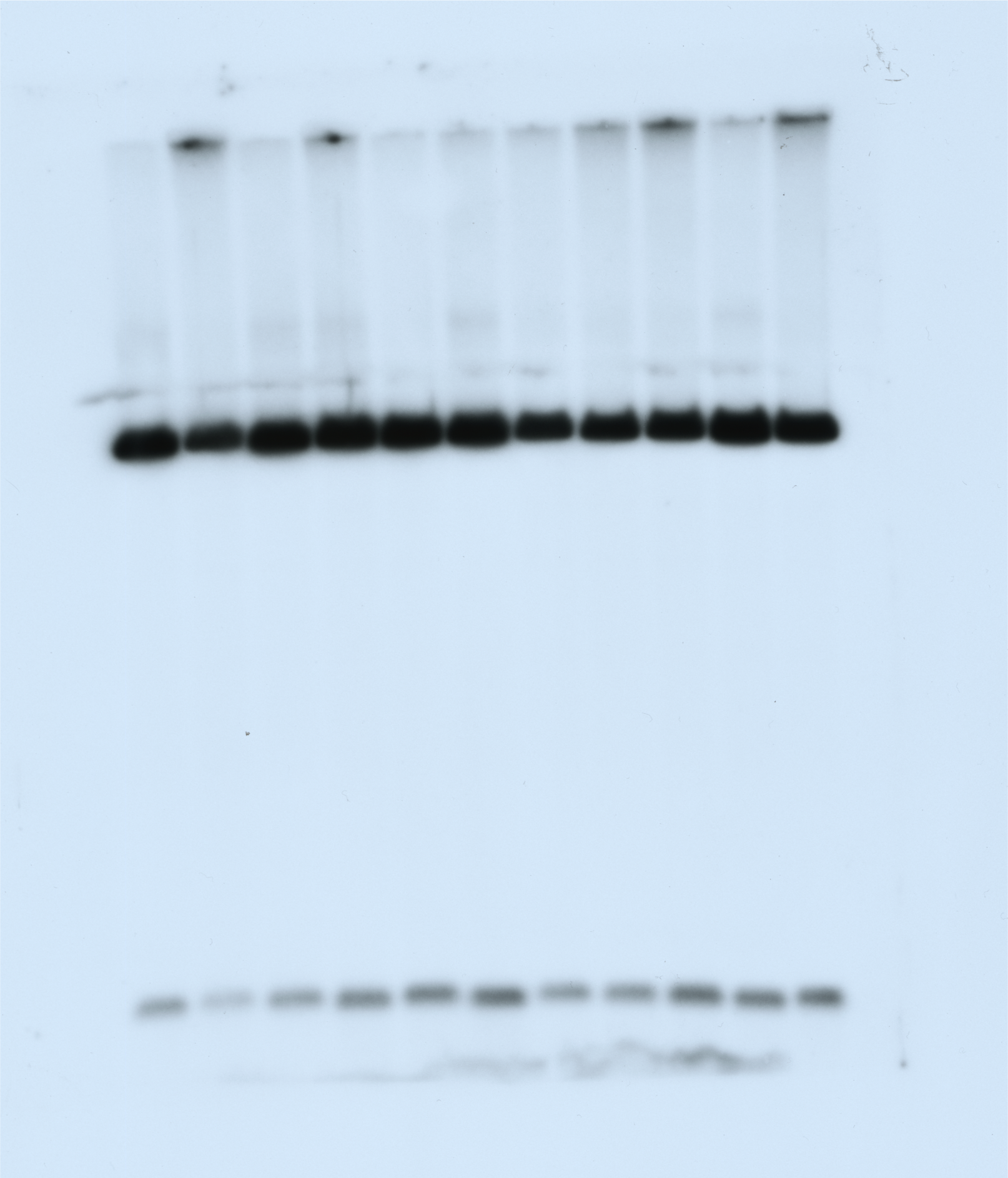

Supplement: Supplementary file 6 — Source Data for Figure 3 [file EMMM-15-e16775-s004.zip › Figure_3/Figure_3F.tif]

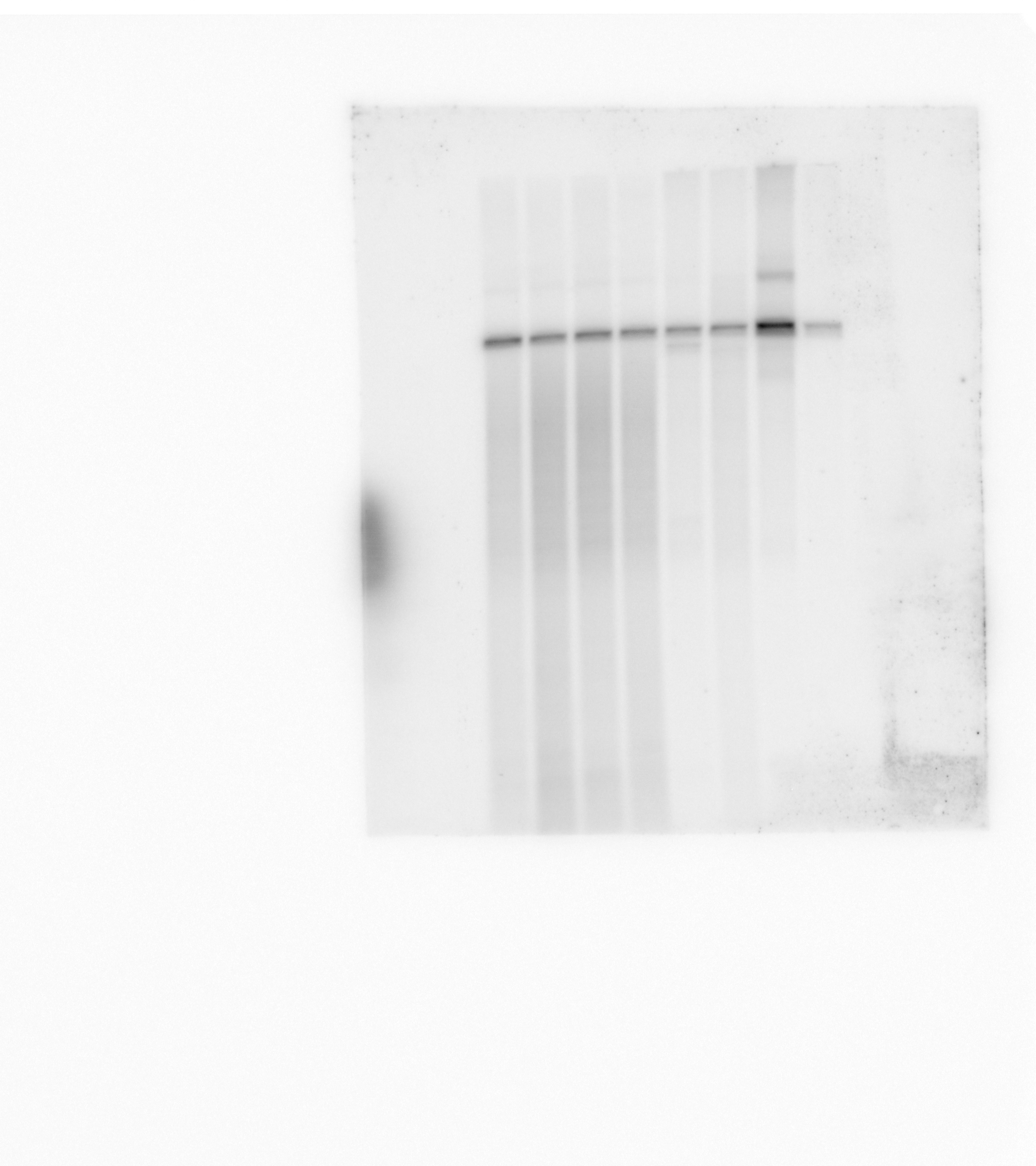

Supplement: Supplementary file 6 — Source Data for Figure 3 [file EMMM-15-e16775-s004.zip › Figure_3/Figure_3A.gel]

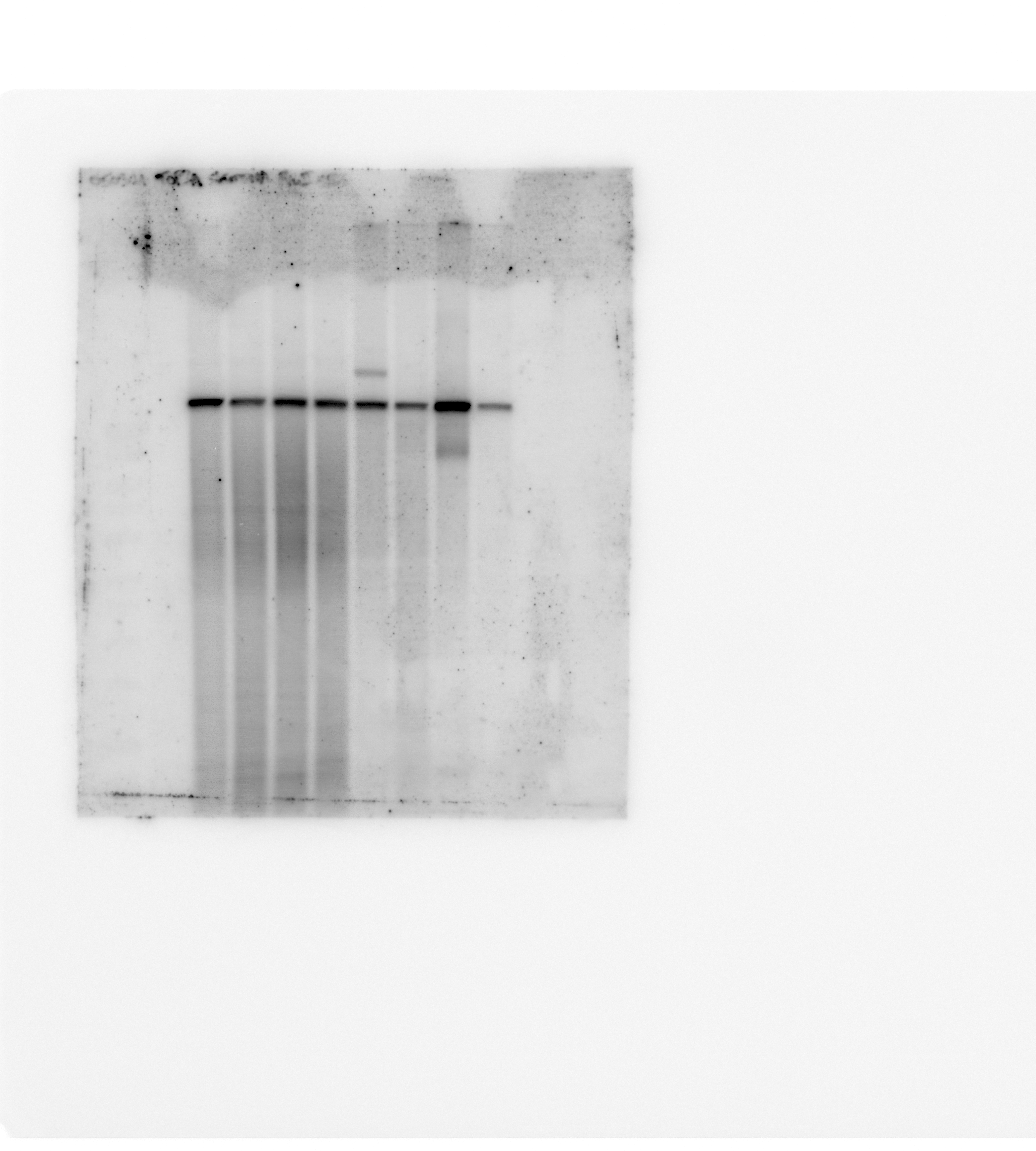

Supplement: Supplementary file 6 — Source Data for Figure 3 [file EMMM-15-e16775-s004.zip › Figure_3/Figure_3B.gel]

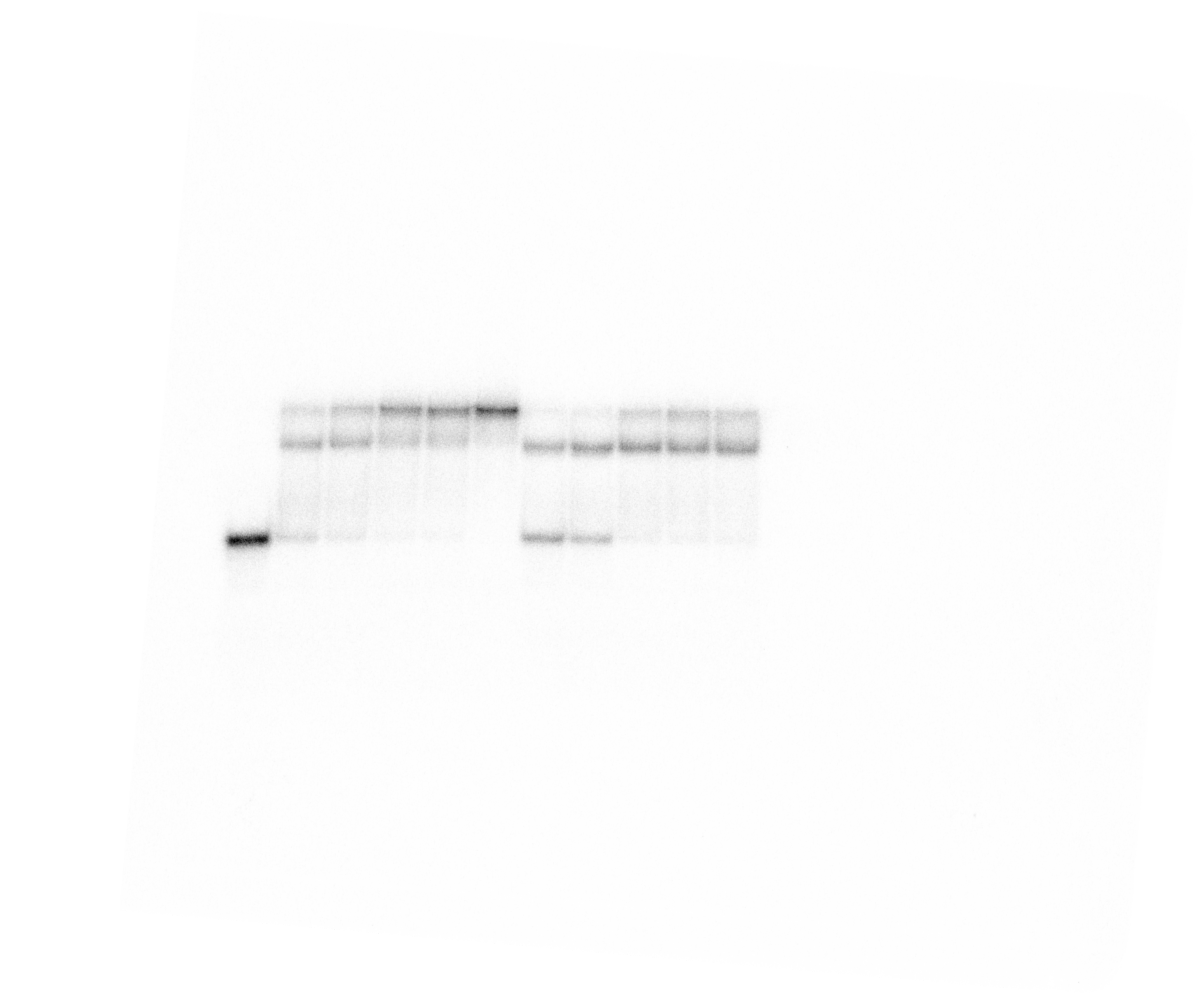

Supplement: Supplementary file 7 — Source Data for Figure 4 [file EMMM-15-e16775-s001.zip › Figure_4/Figure_4D.tif]

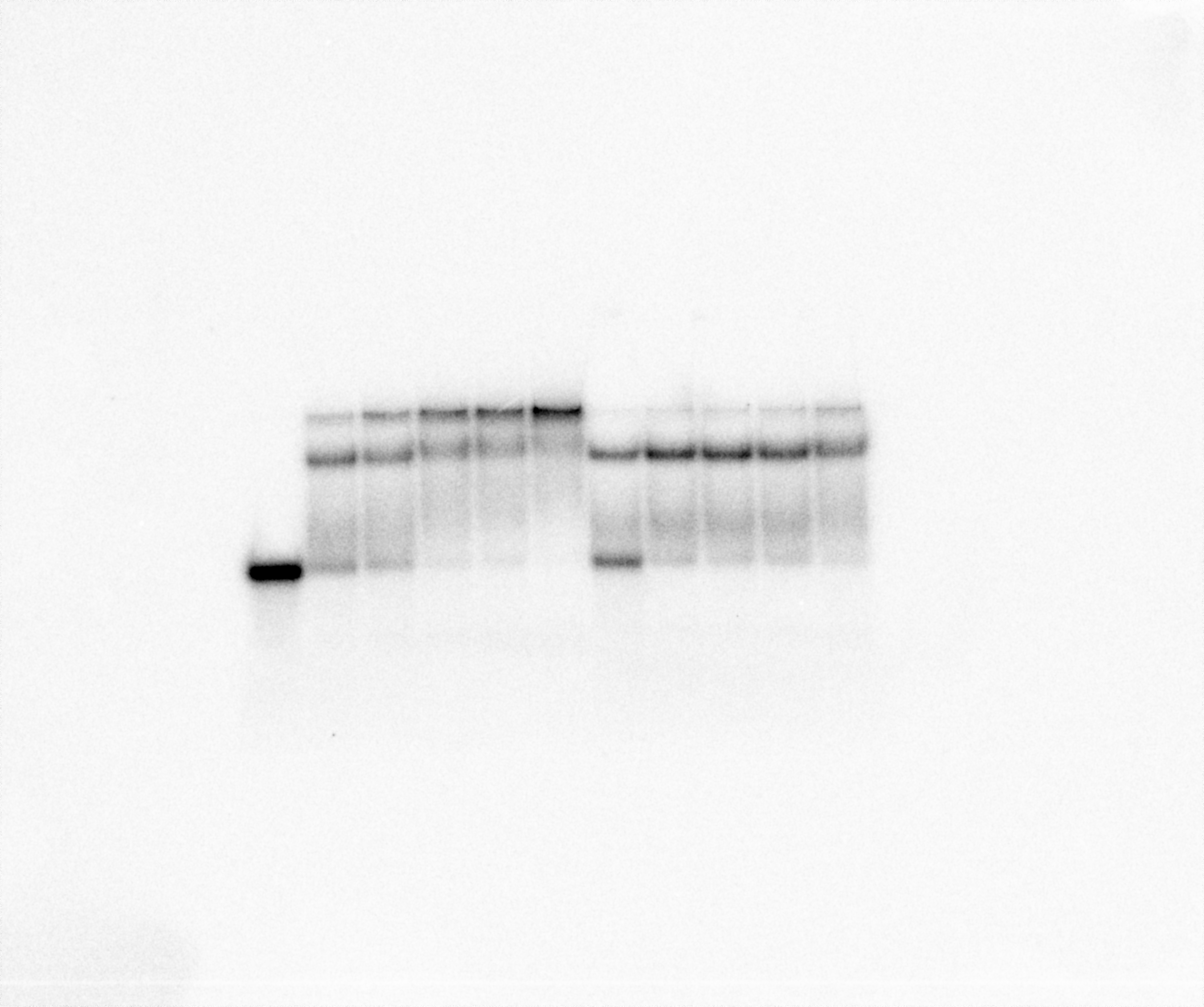

Supplement: Supplementary file 7 — Source Data for Figure 4 [file EMMM-15-e16775-s001.zip › Figure_4/Figure_4F.tif]

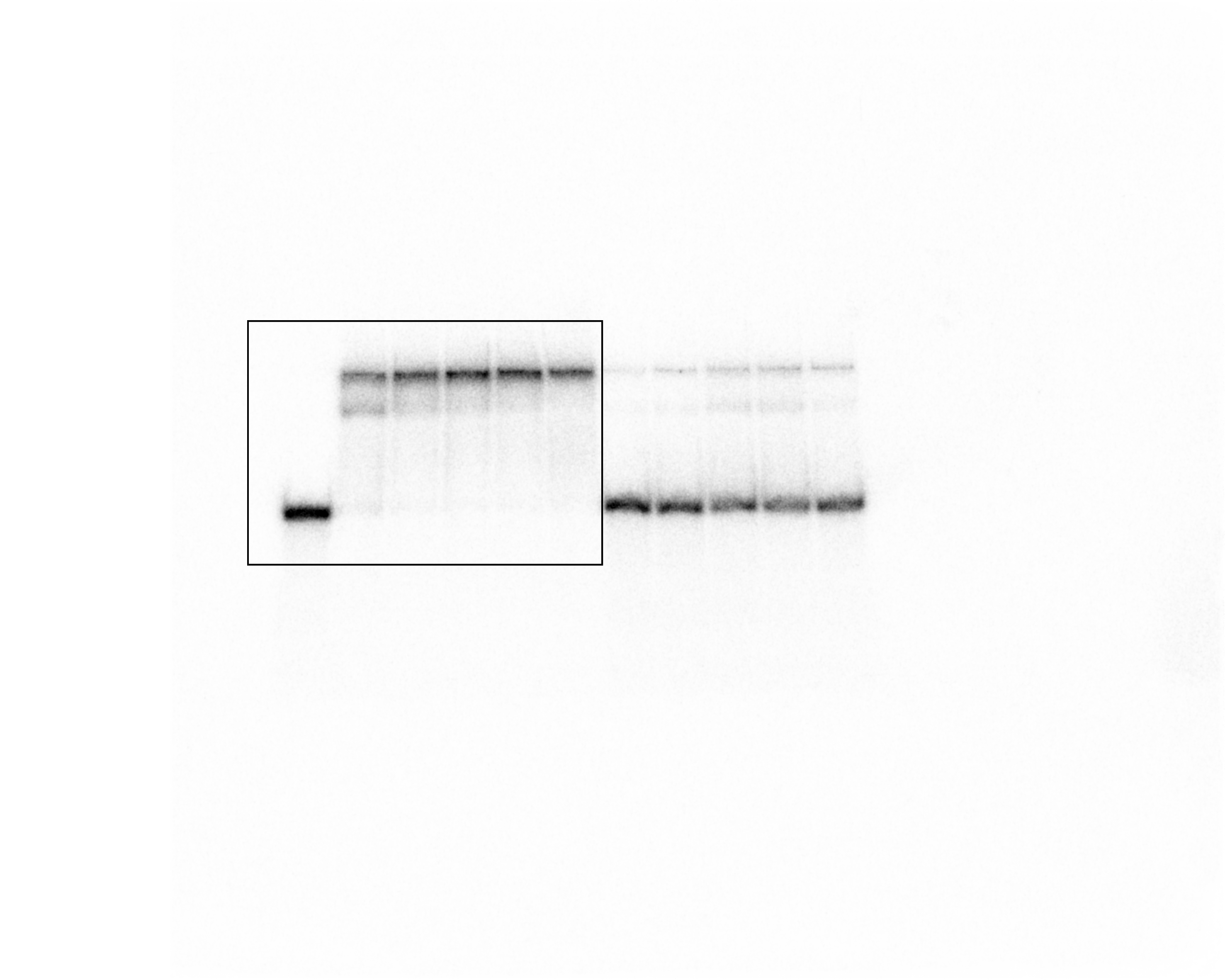

Supplement: Supplementary file 7 — Source Data for Figure 4 [file EMMM-15-e16775-s001.zip › Figure_4/Figure_4B.tif]

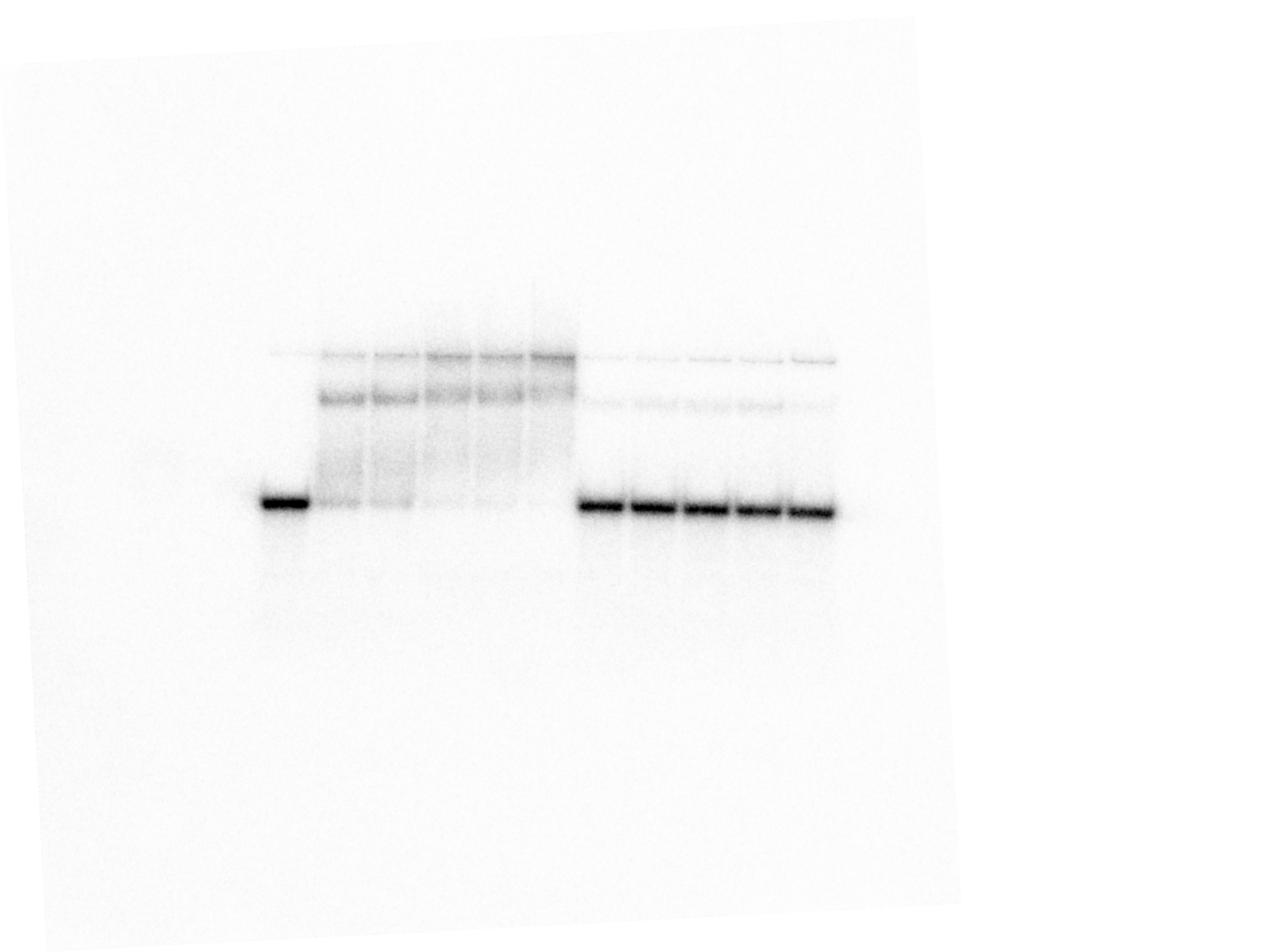

Supplement: Supplementary file 7 — Source Data for Figure 4 [file EMMM-15-e16775-s001.zip › Figure_4/Figure_4C.tif]

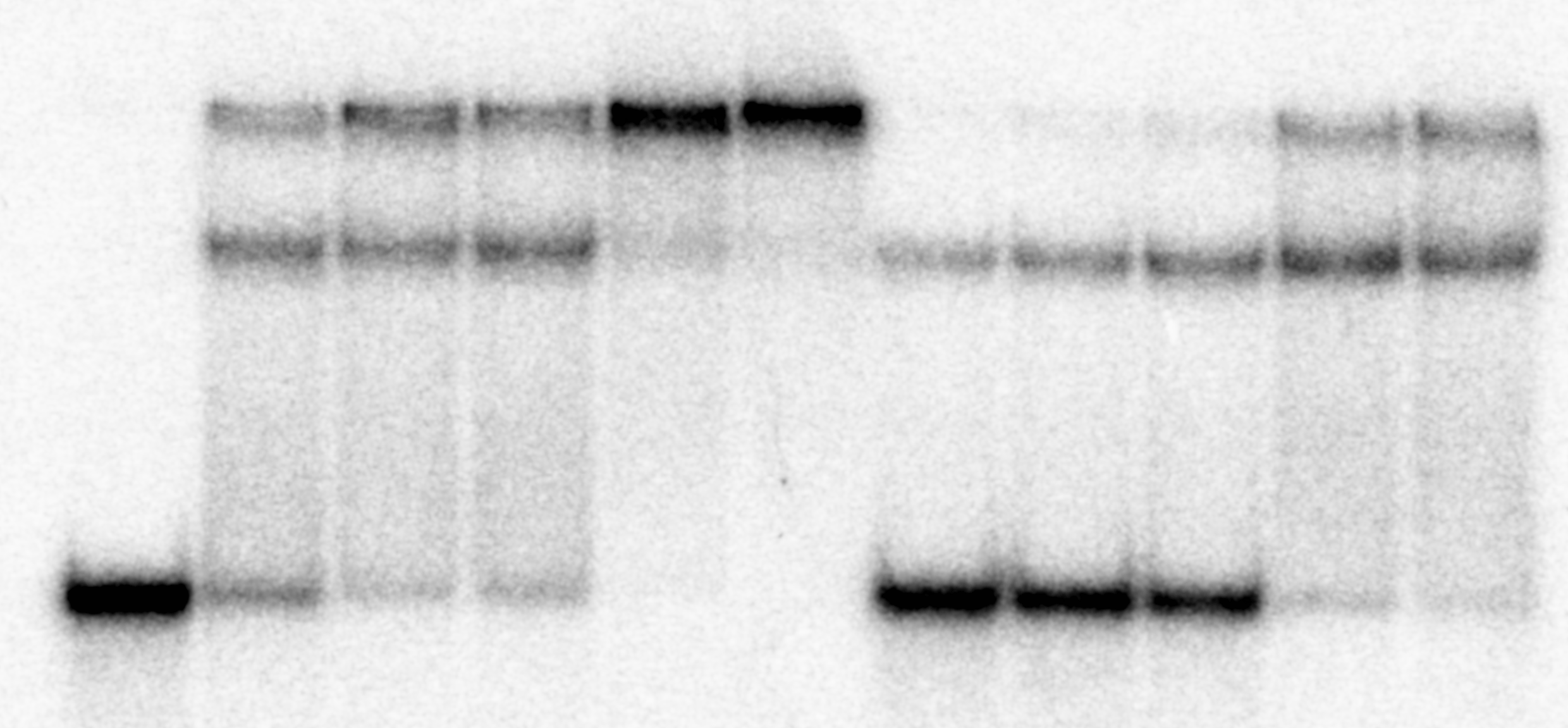

Supplement: Supplementary file 7 — Source Data for Figure 4 [file EMMM-15-e16775-s001.zip › Figure_4/Figure_4G.tiff]

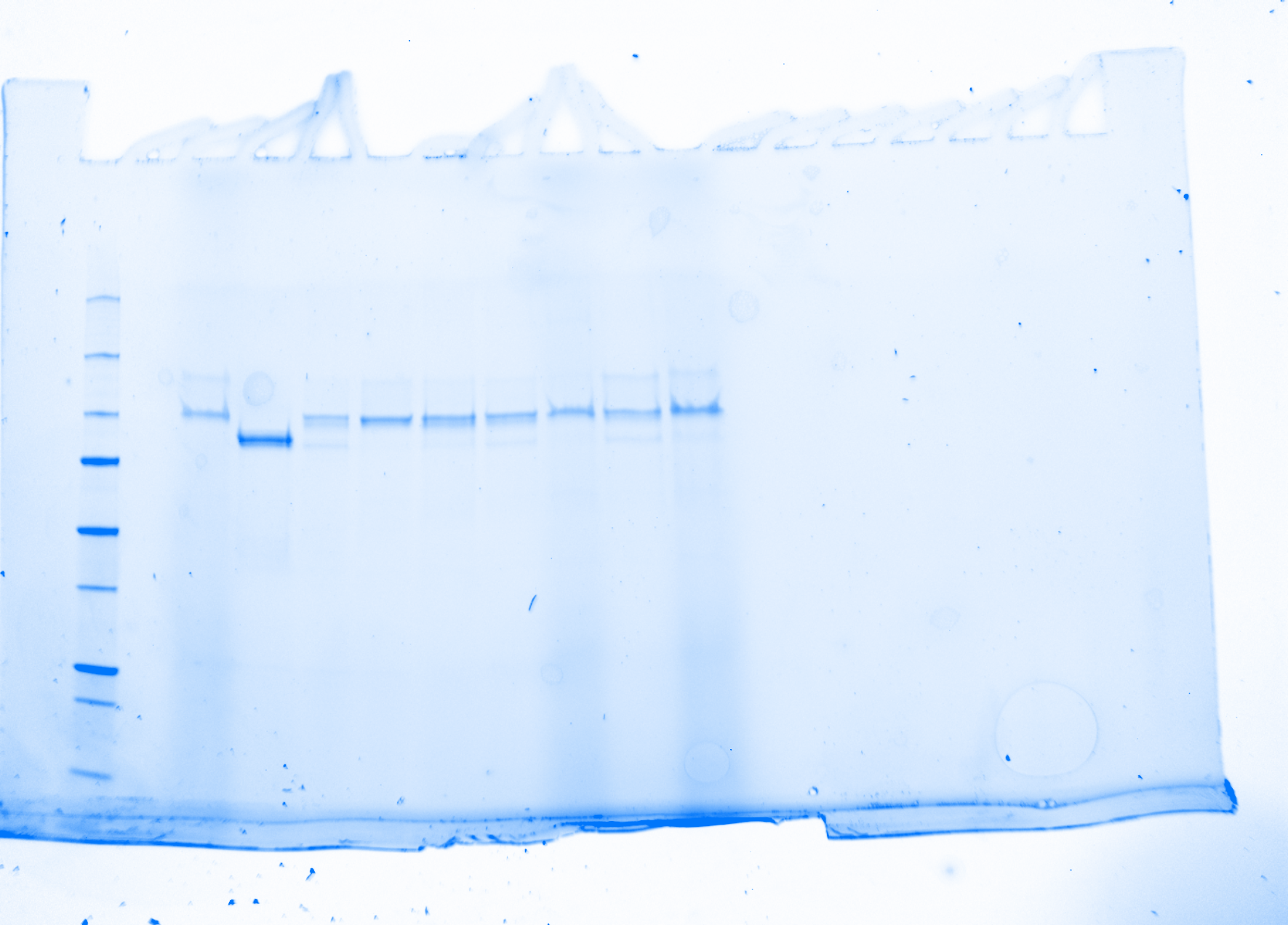

Supplement: Supplementary file 7 — Source Data for Figure 4 [file EMMM-15-e16775-s001.zip › Figure_4/Figure_4A.png]

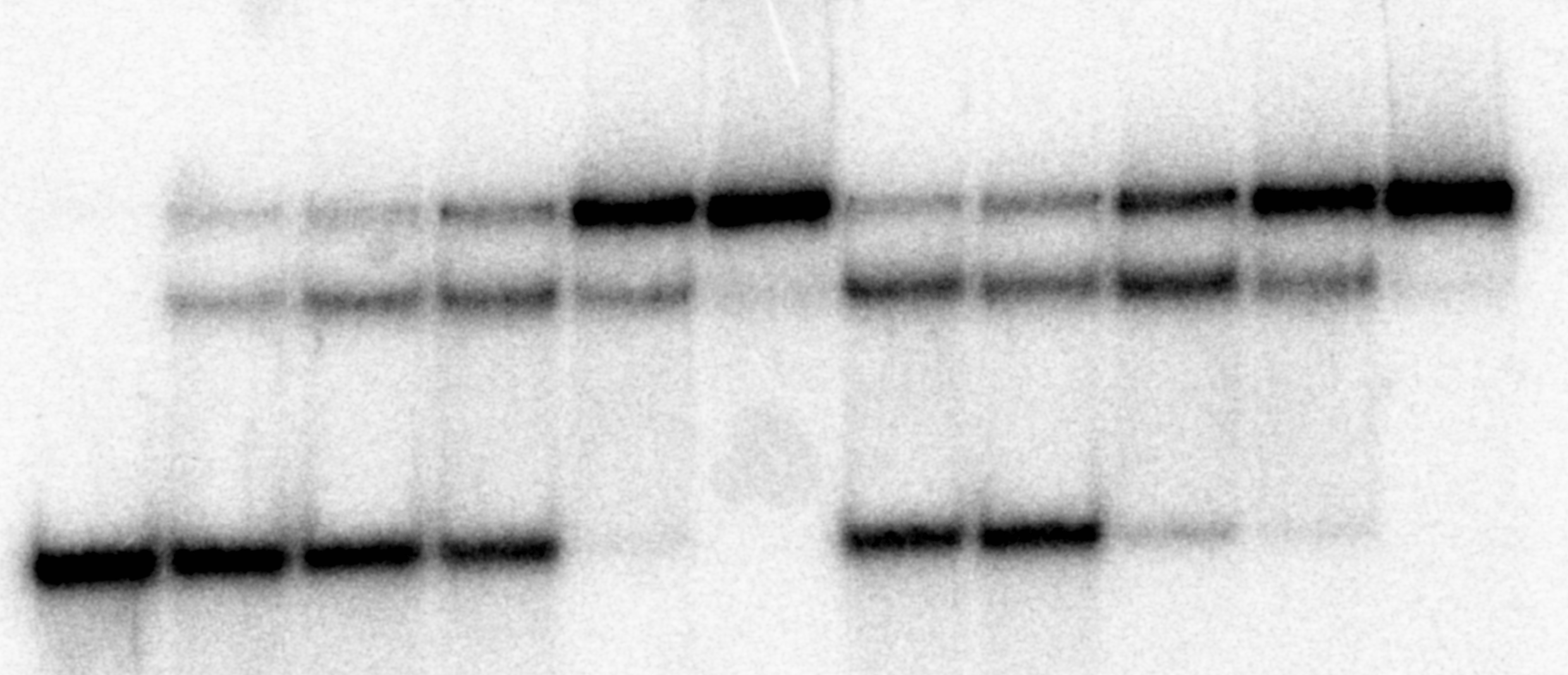

Supplement: Supplementary file 7 — Source Data for Figure 4 [file EMMM-15-e16775-s001.zip › Figure_4/Figure_4E.tiff]

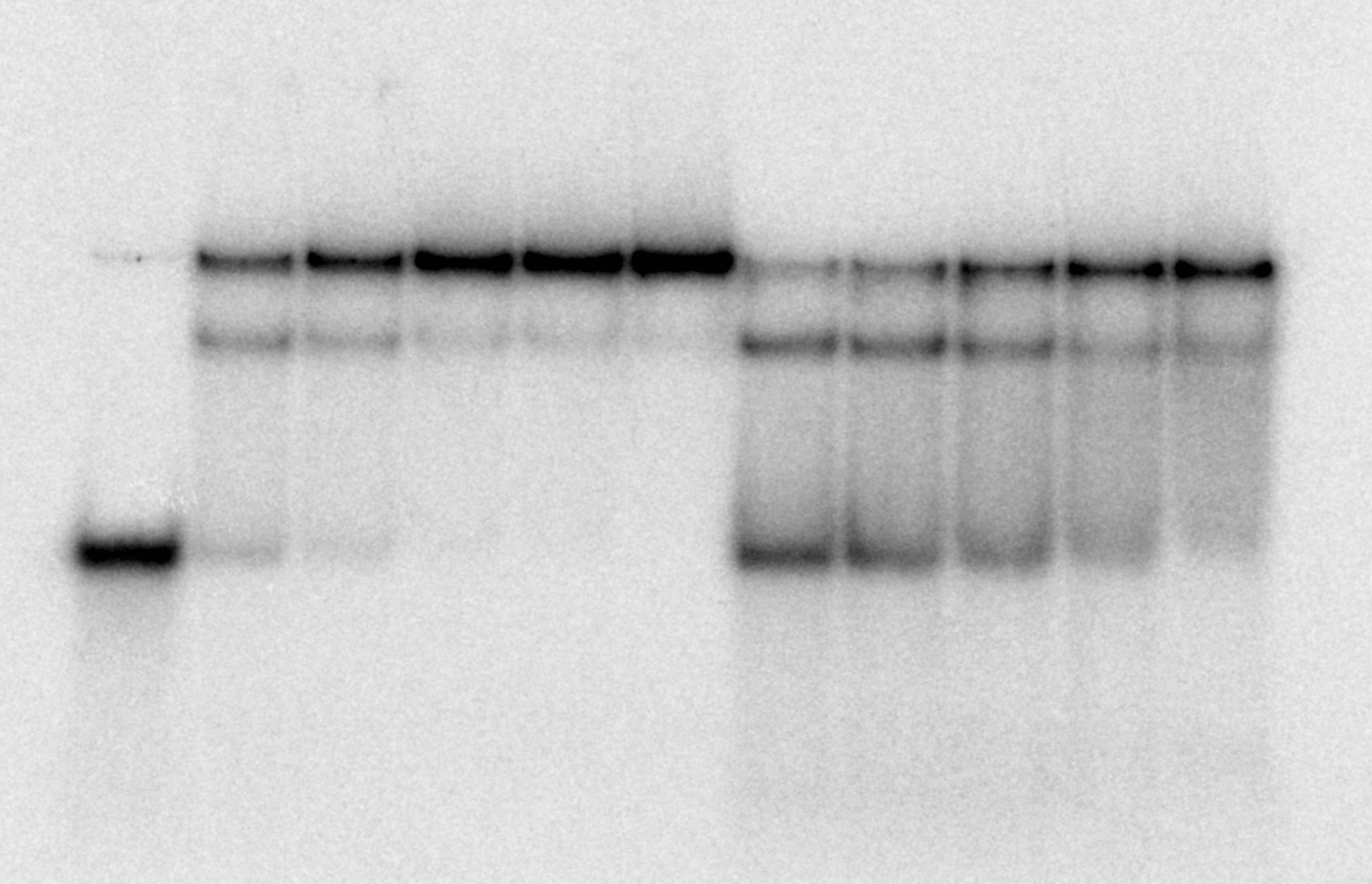

Supplement: Supplementary file 7 — Source Data for Figure 4 [file EMMM-15-e16775-s001.zip › Figure_4/Figure_4H.tiff]

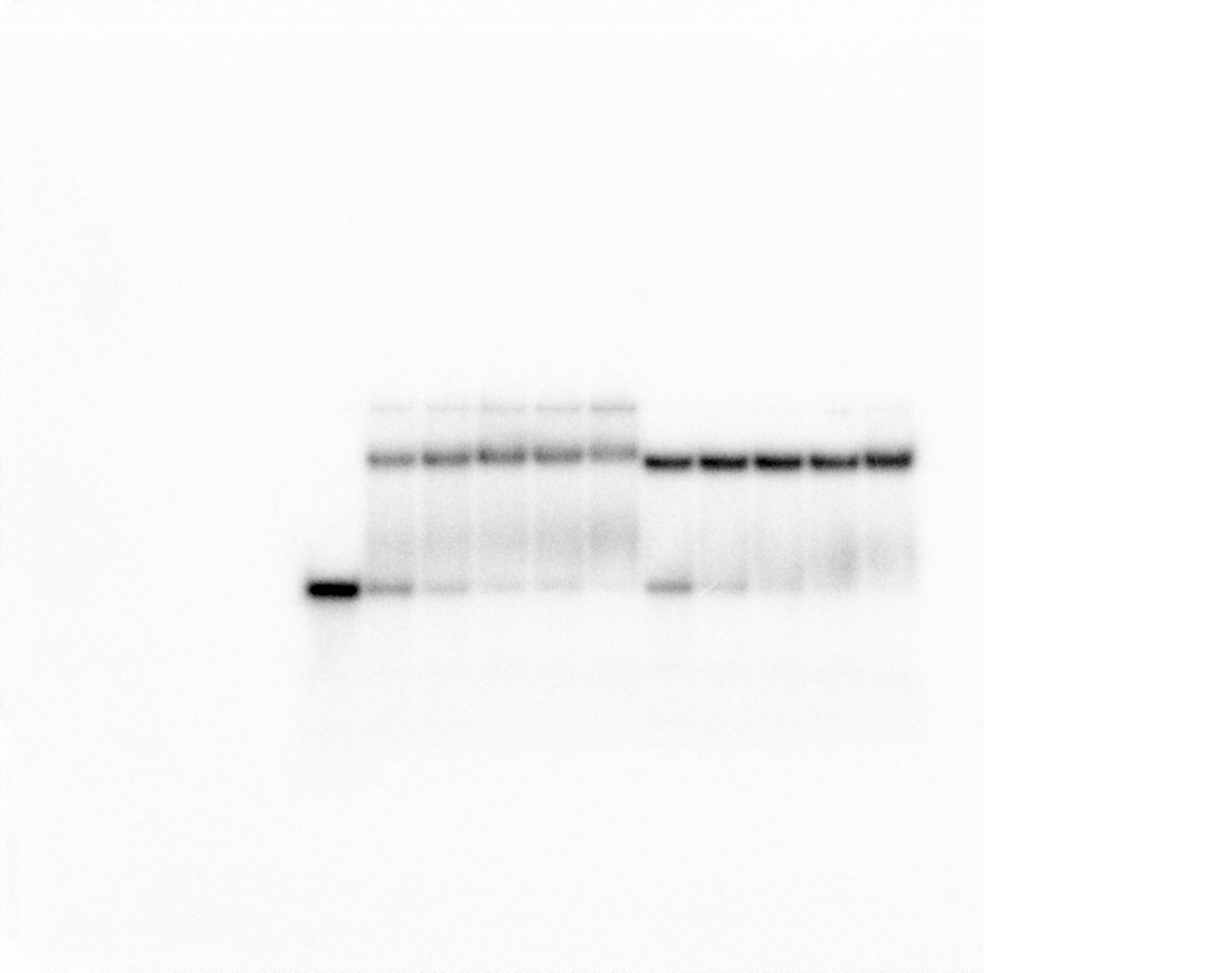

Supplement: Supplementary file 7 — Source Data for Figure 4 [file EMMM-15-e16775-s001.zip › Figure_4/Figure_4J.tif]

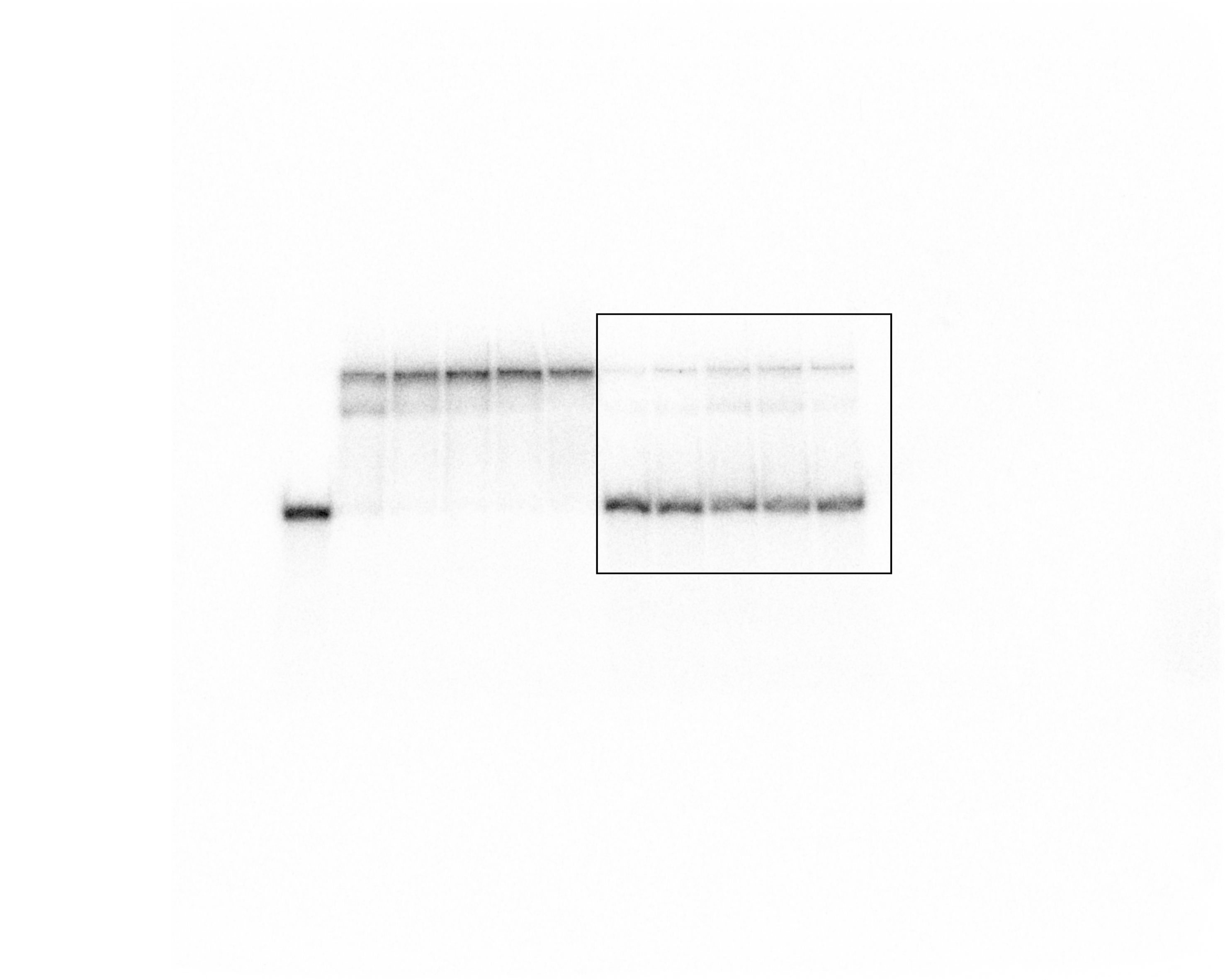

Supplement: Supplementary file 7 — Source Data for Figure 4 [file EMMM-15-e16775-s001.zip › Figure_4/Figure_4I.tif]

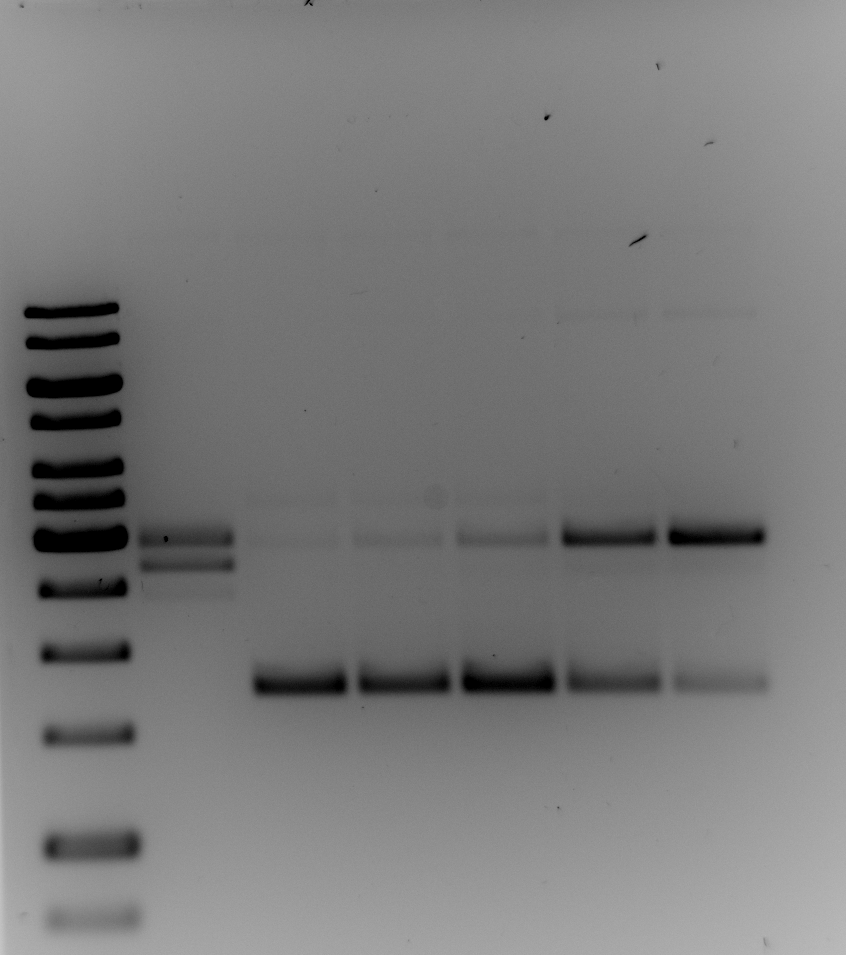

Supplement: Supplementary file 8 — Source Data for Figure 5 [file EMMM-15-e16775-s008.zip › Figure_5/Figure_5A.tif]

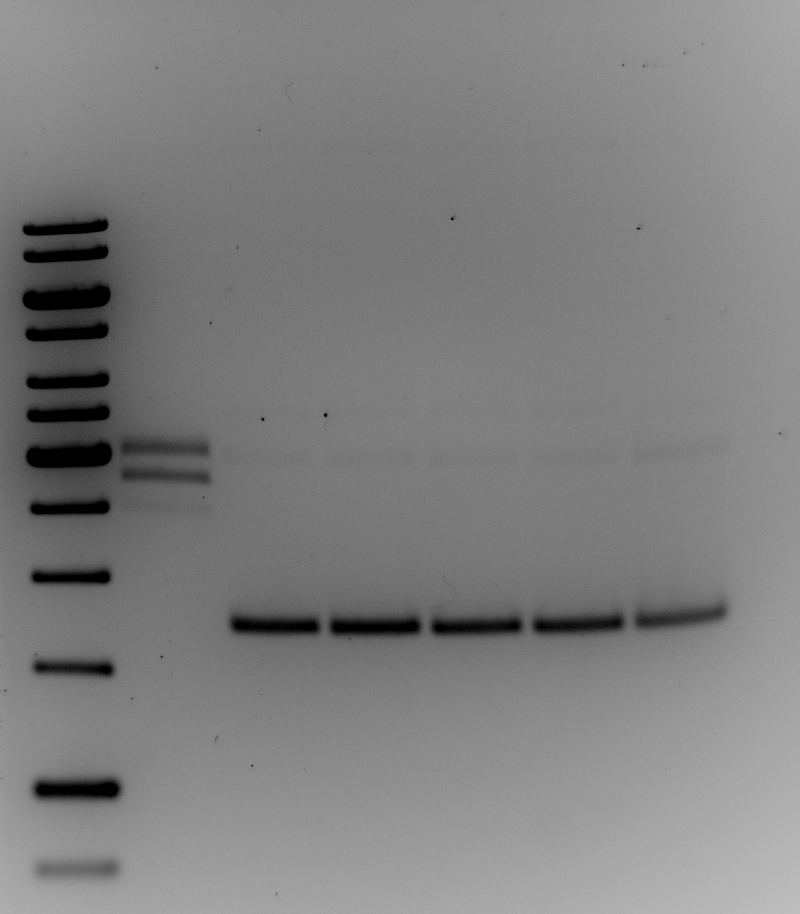

Supplement: Supplementary file 8 — Source Data for Figure 5 [file EMMM-15-e16775-s008.zip › Figure_5/Figure_5C.tif]

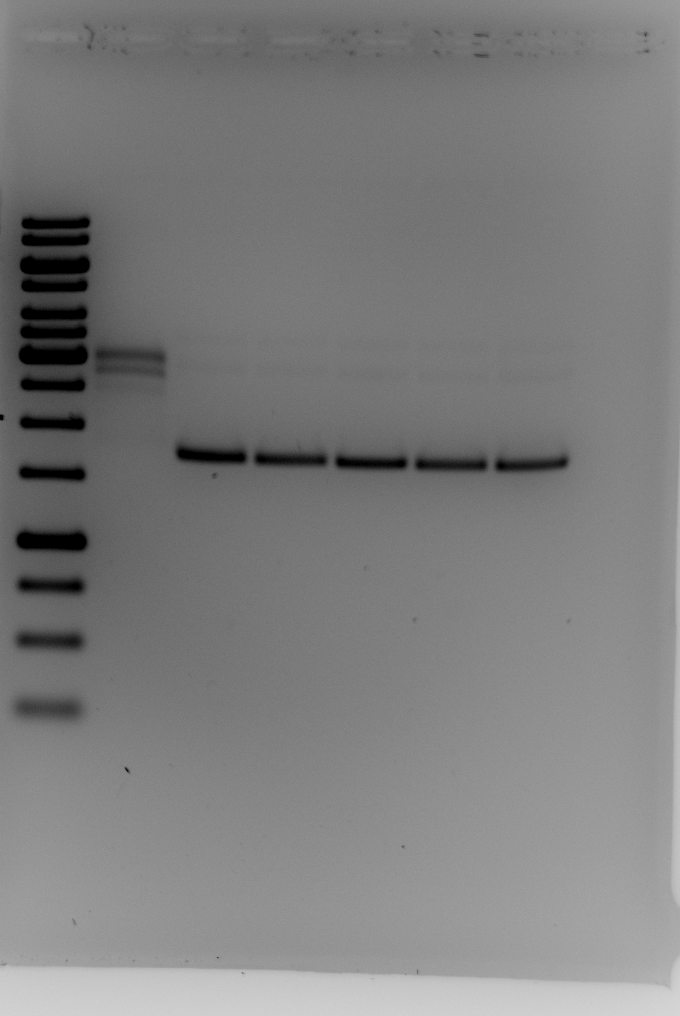

Supplement: Supplementary file 8 — Source Data for Figure 5 [file EMMM-15-e16775-s008.zip › Figure_5/Figure_5B.tif]

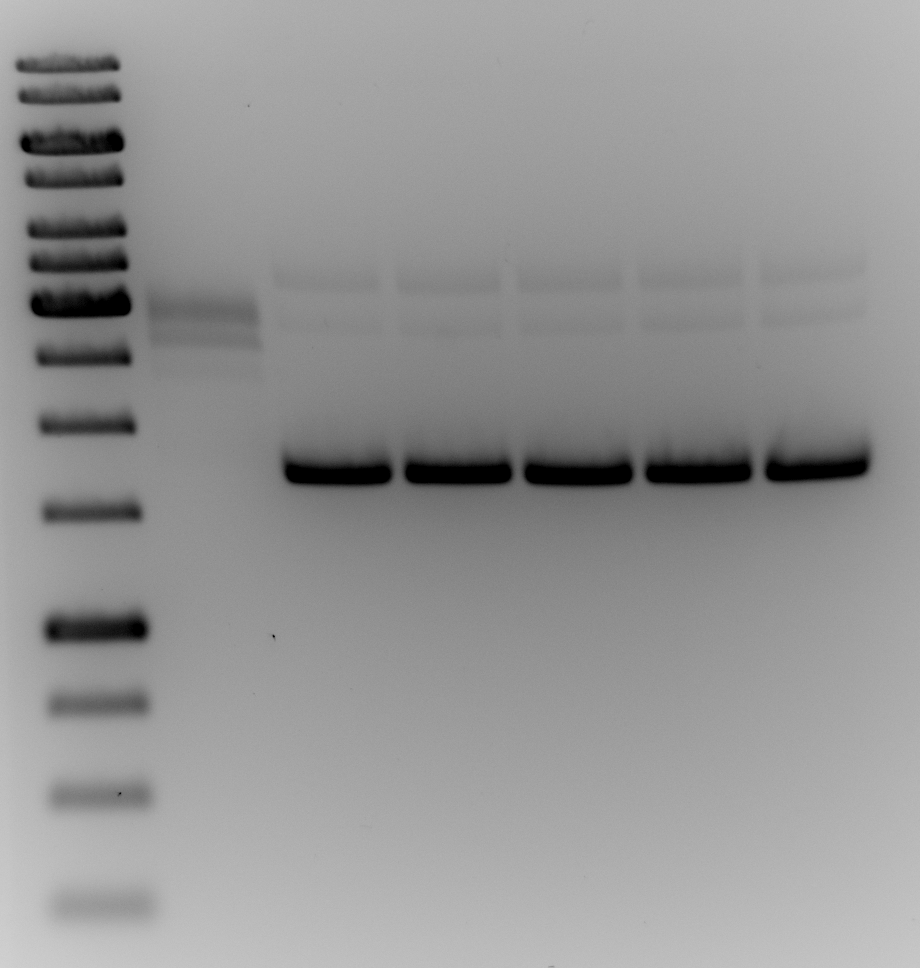

Supplement: Supplementary file 8 — Source Data for Figure 5 [file EMMM-15-e16775-s008.zip › Figure_5/Figure_5F.tif]

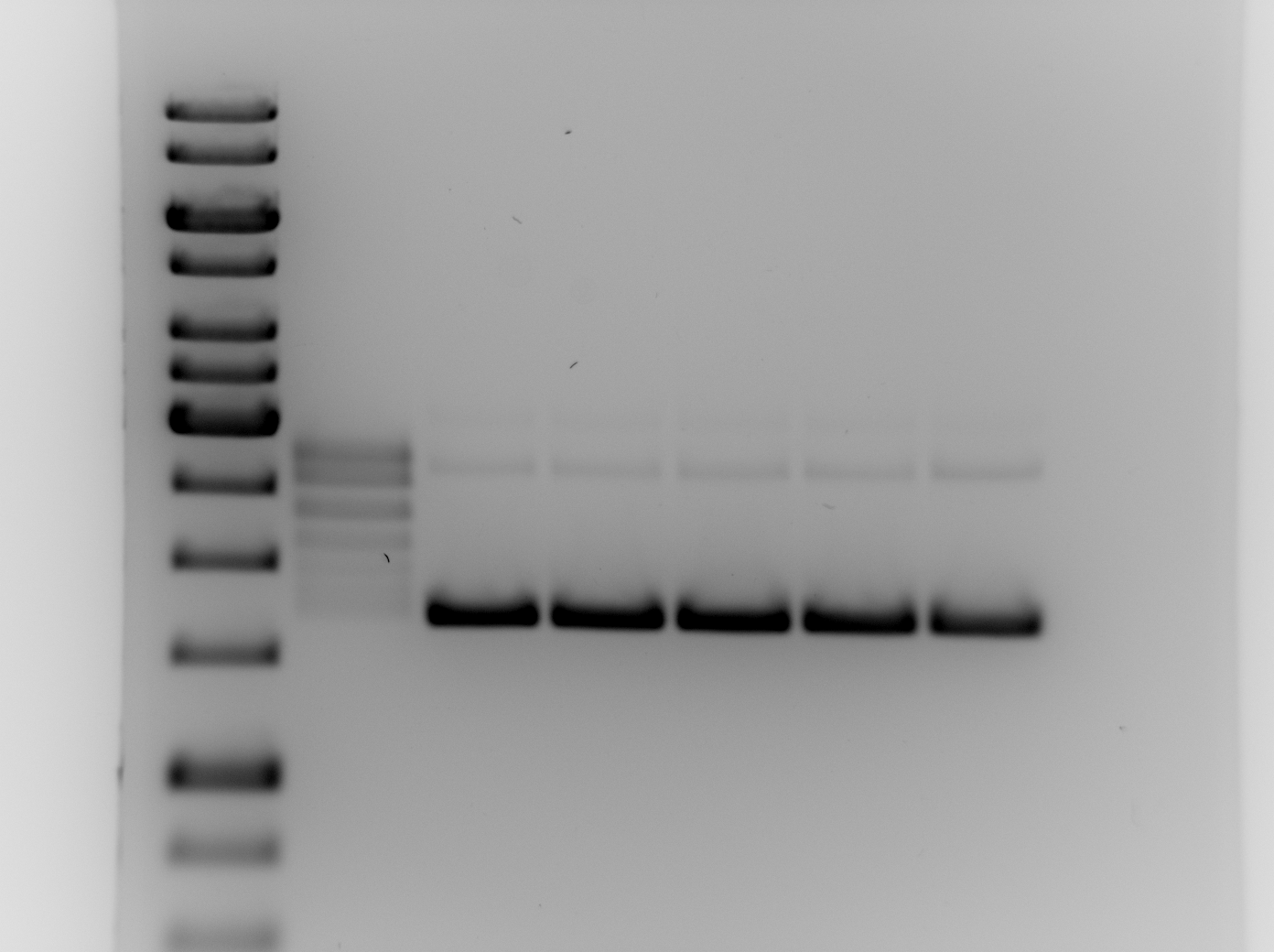

Supplement: Supplementary file 8 — Source Data for Figure 5 [file EMMM-15-e16775-s008.zip › Figure_5/Figure_5G.tif]

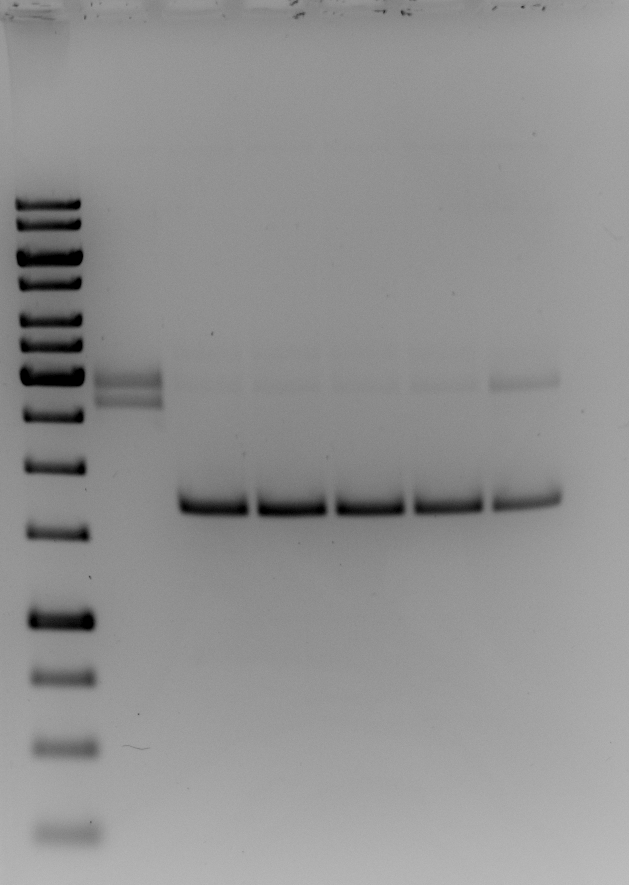

Supplement: Supplementary file 8 — Source Data for Figure 5 [file EMMM-15-e16775-s008.zip › Figure_5/Figure_5E.tif]

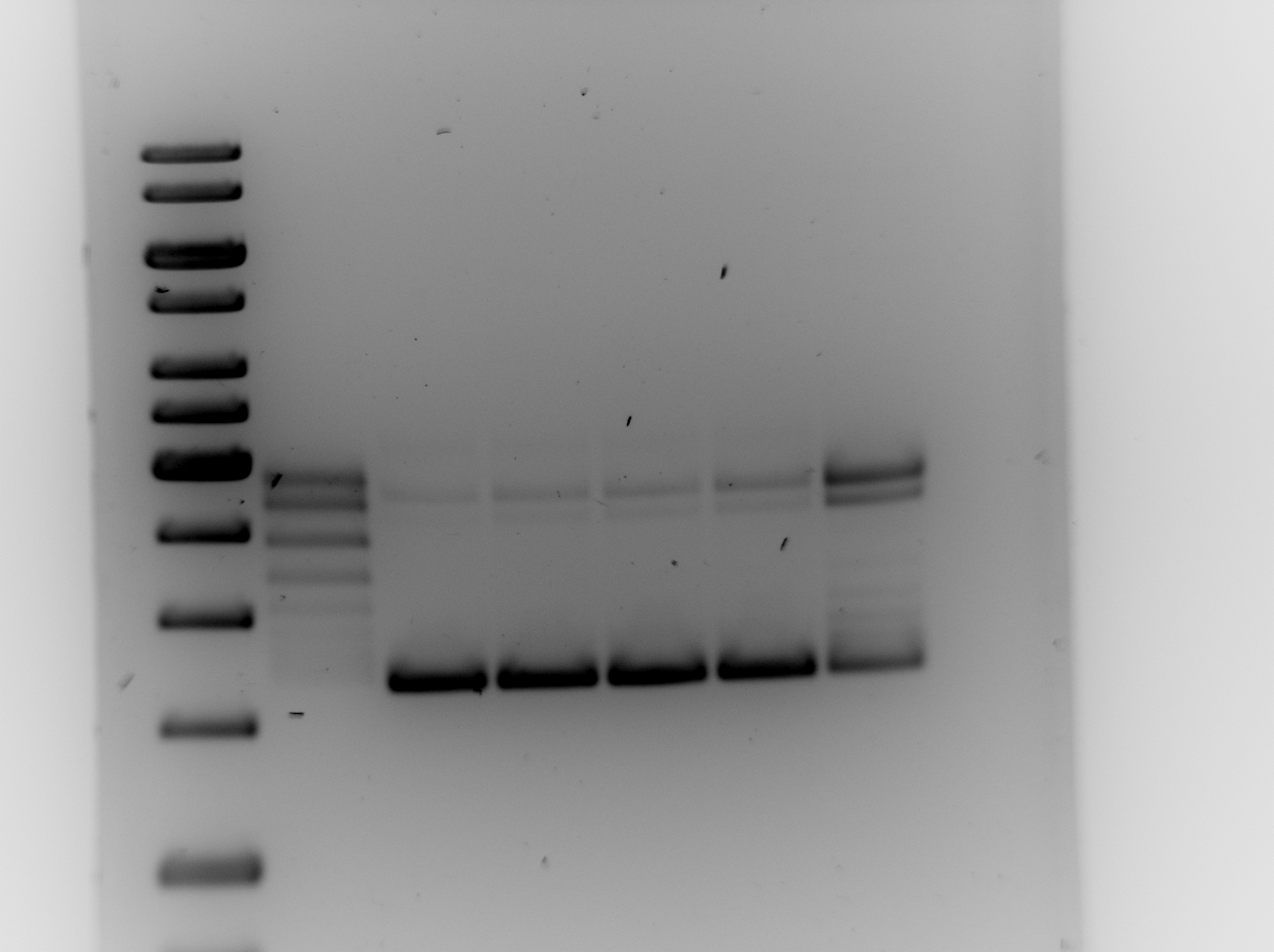

Supplement: Supplementary file 8 — Source Data for Figure 5 [file EMMM-15-e16775-s008.zip › Figure_5/Figure_5D.tif]

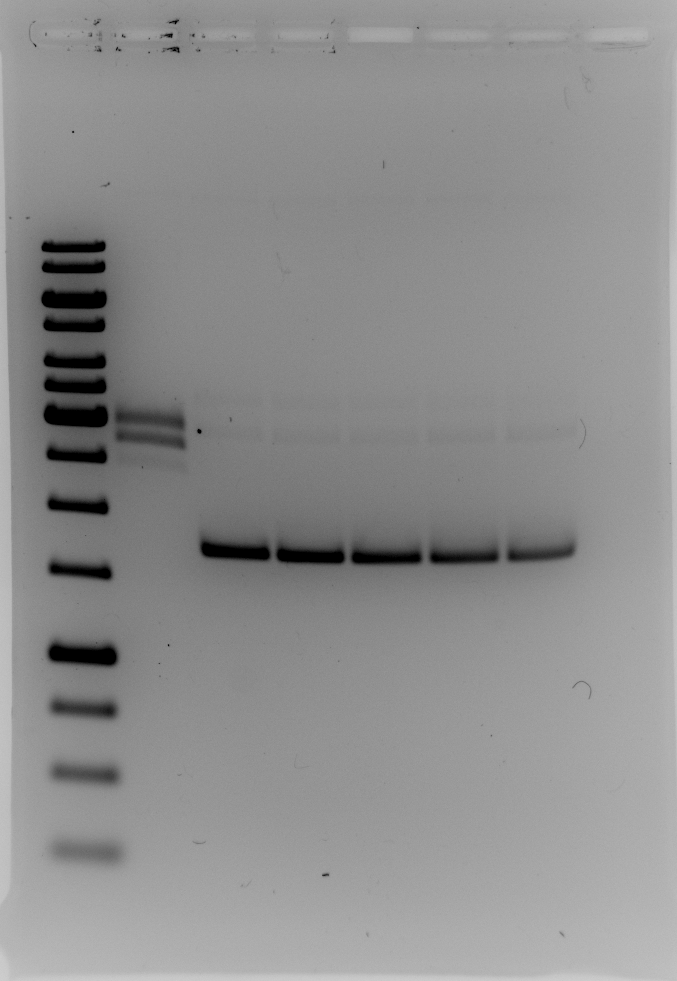

Supplement: Supplementary file 8 — Source Data for Figure 5 [file EMMM-15-e16775-s008.zip › Figure_5/Figure_5I.tif]

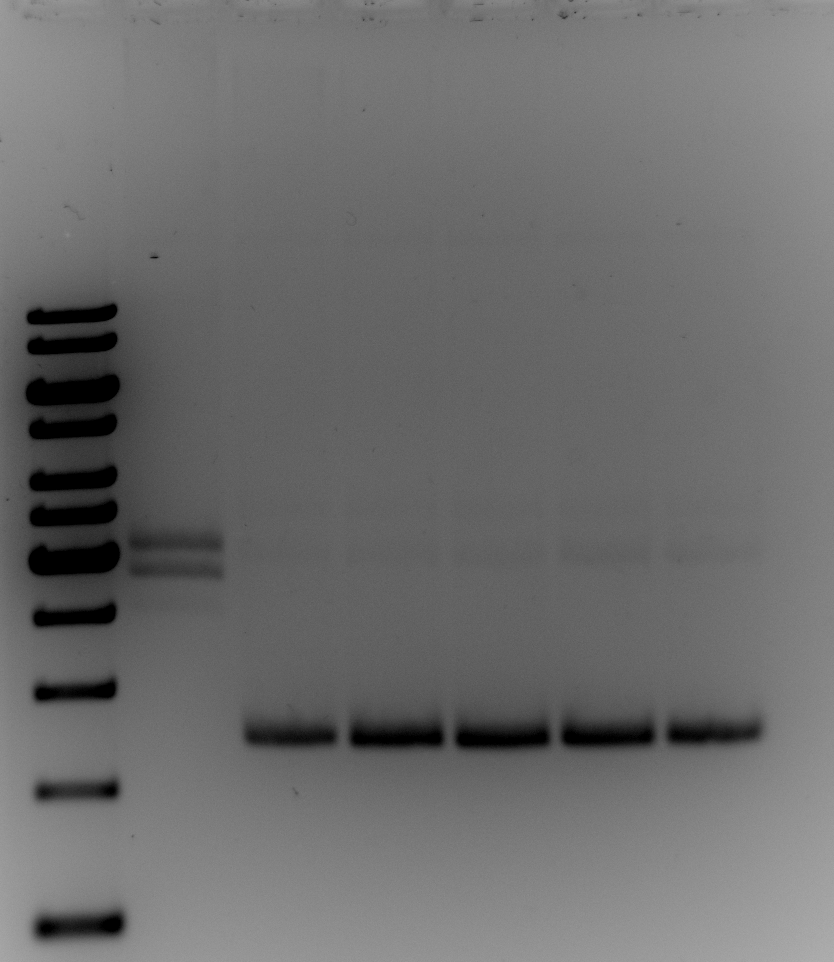

Supplement: Supplementary file 8 — Source Data for Figure 5 [file EMMM-15-e16775-s008.zip › Figure_5/Figure_5H.tif]

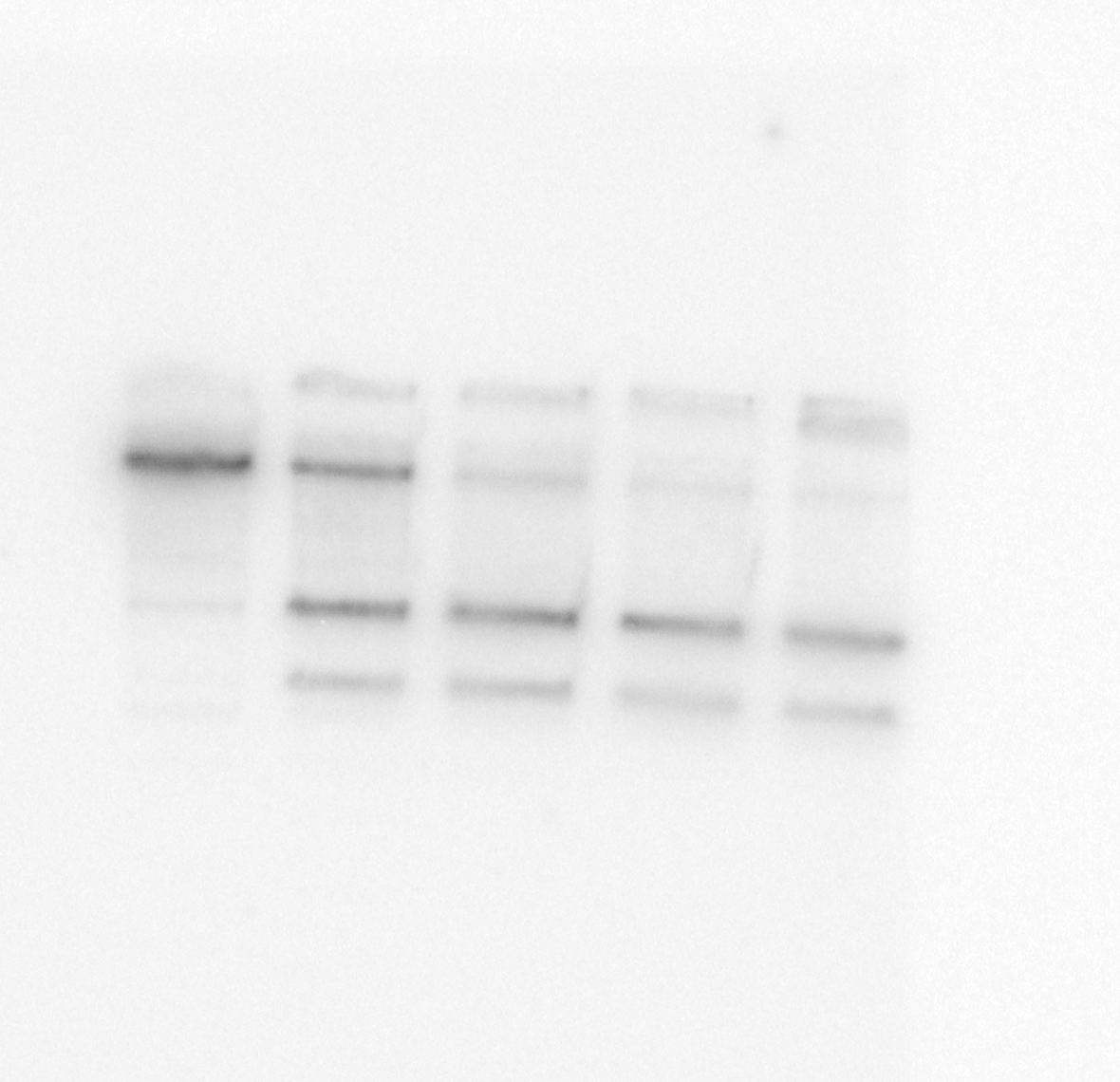

Supplement: Supplementary file 9 — Source Data for Figure 6 [file EMMM-15-e16775-s010.zip › Figure_6/Figure_6J.tif]

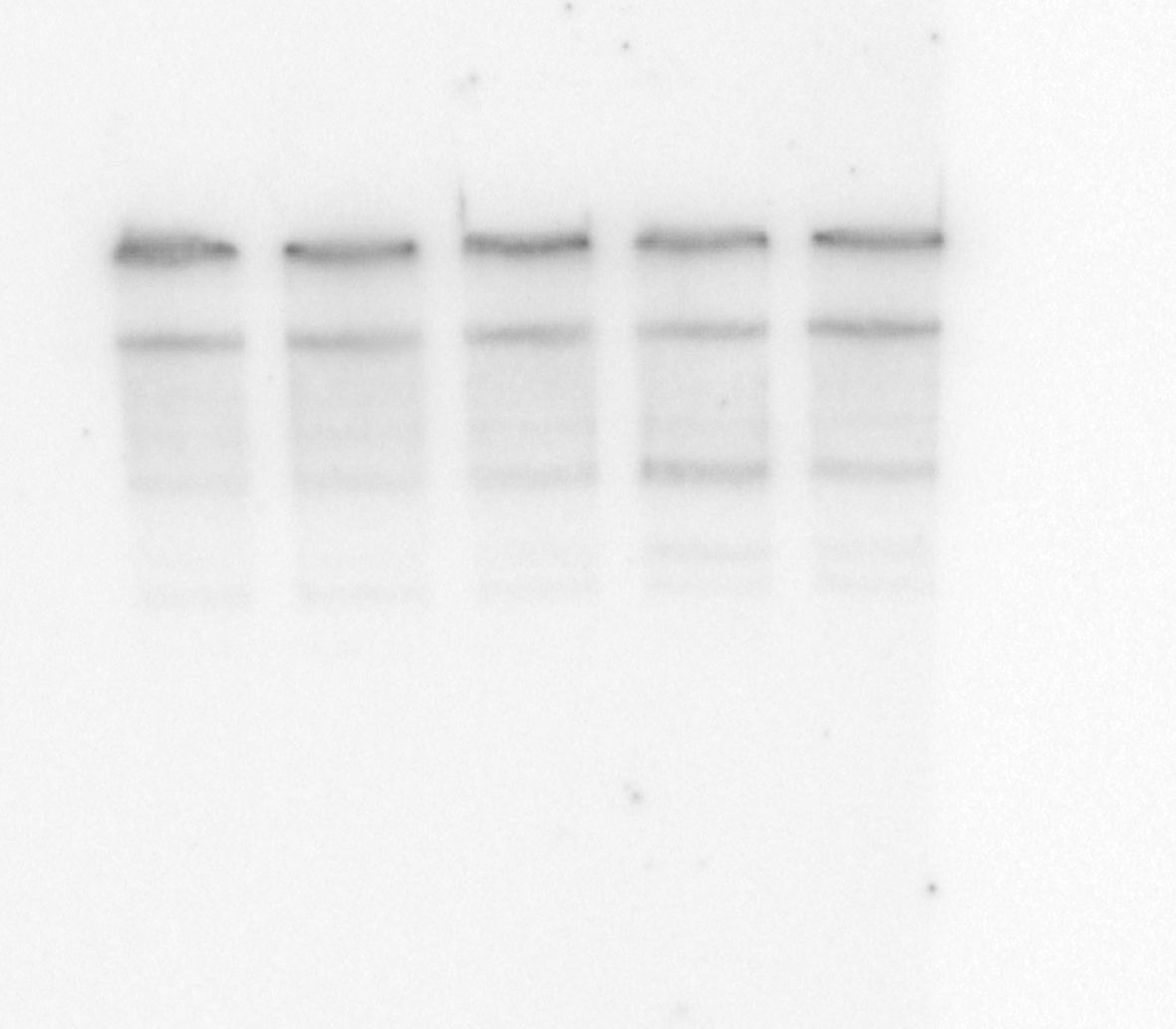

Supplement: Supplementary file 9 — Source Data for Figure 6 [file EMMM-15-e16775-s010.zip › Figure_6/Figure_6I.tif]

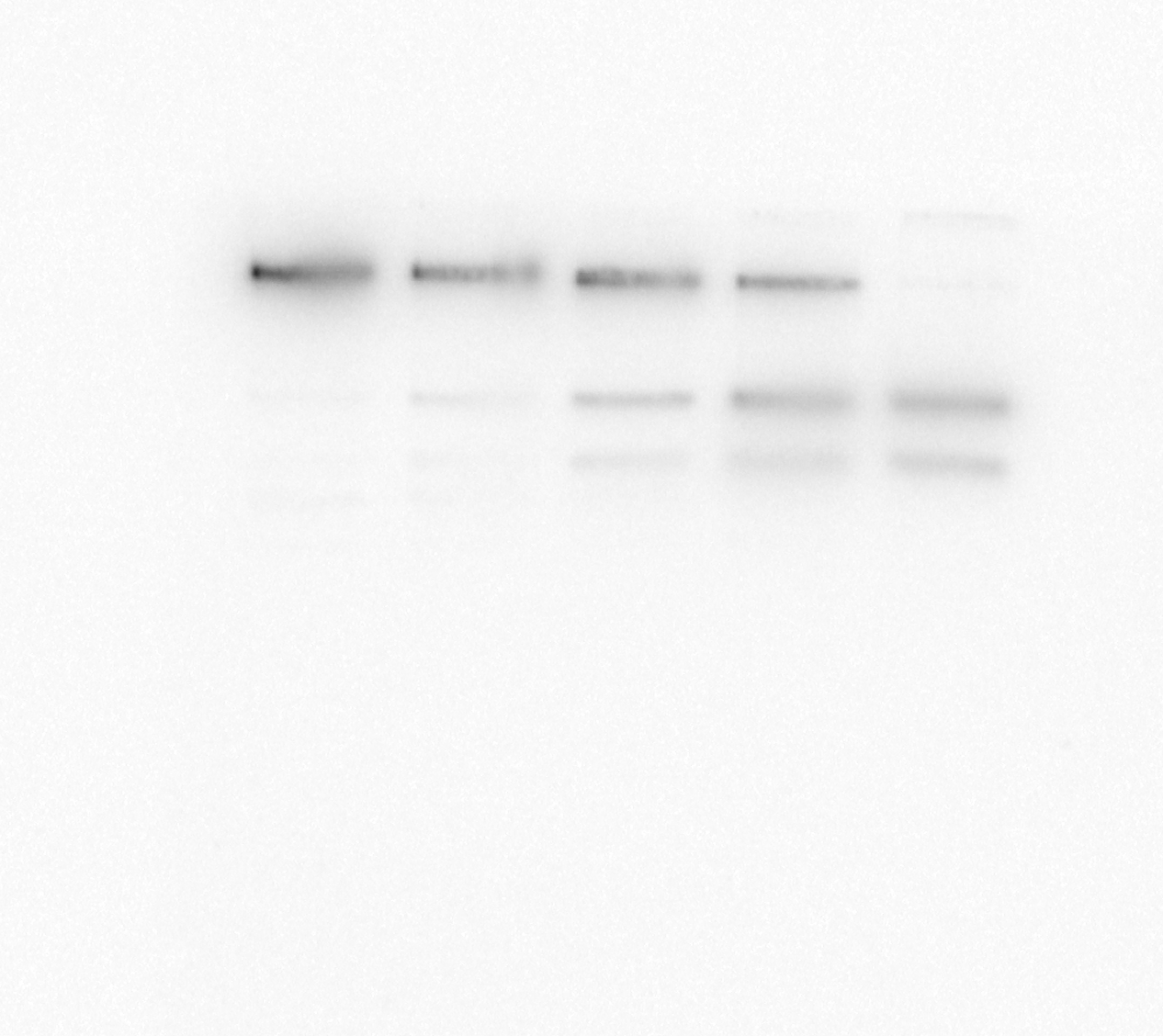

Supplement: Supplementary file 9 — Source Data for Figure 6 [file EMMM-15-e16775-s010.zip › Figure_6/Figure_6H.tif]

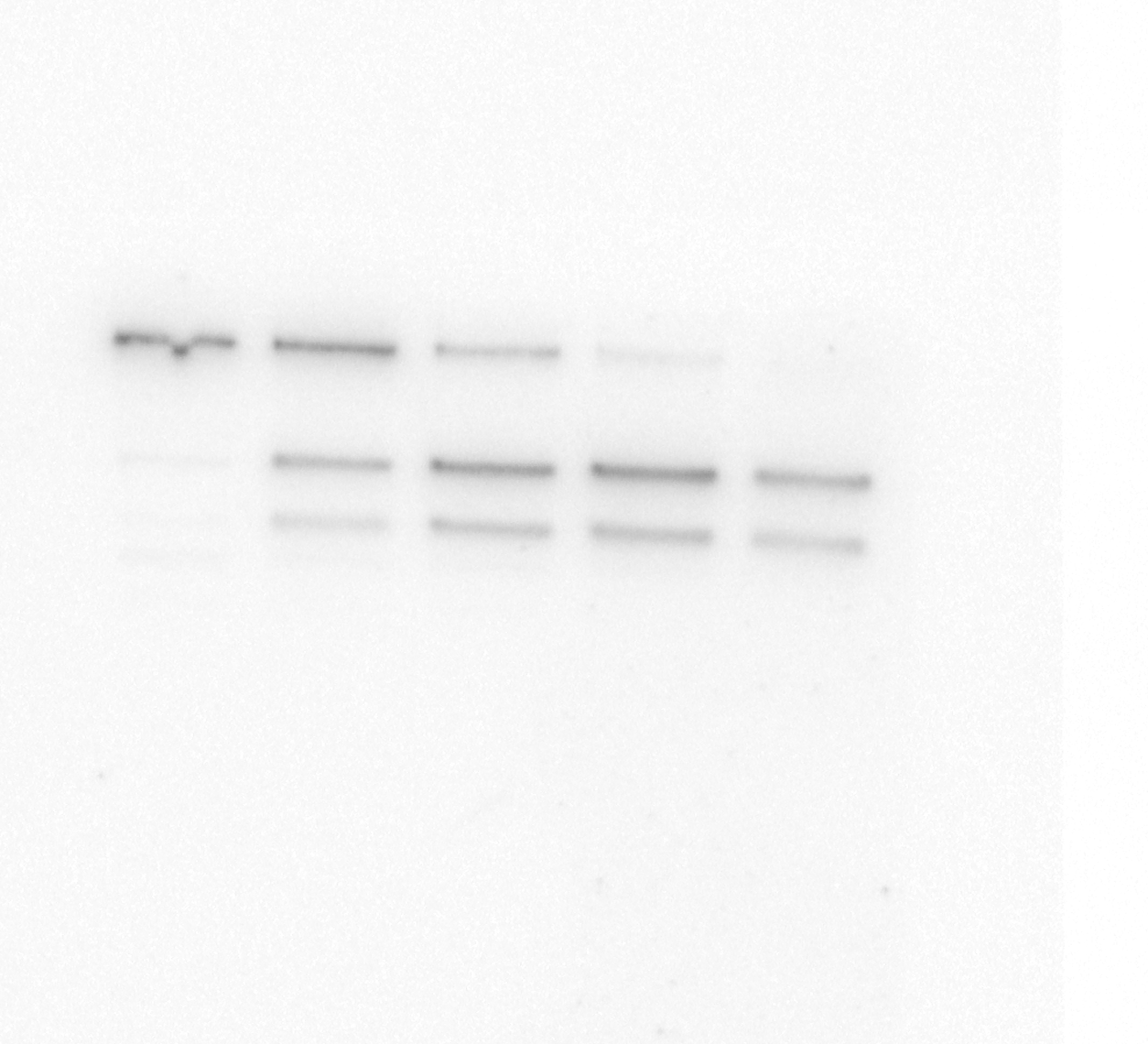

Supplement: Supplementary file 9 — Source Data for Figure 6 [file EMMM-15-e16775-s010.zip › Figure_6/Figure_6E.tif]

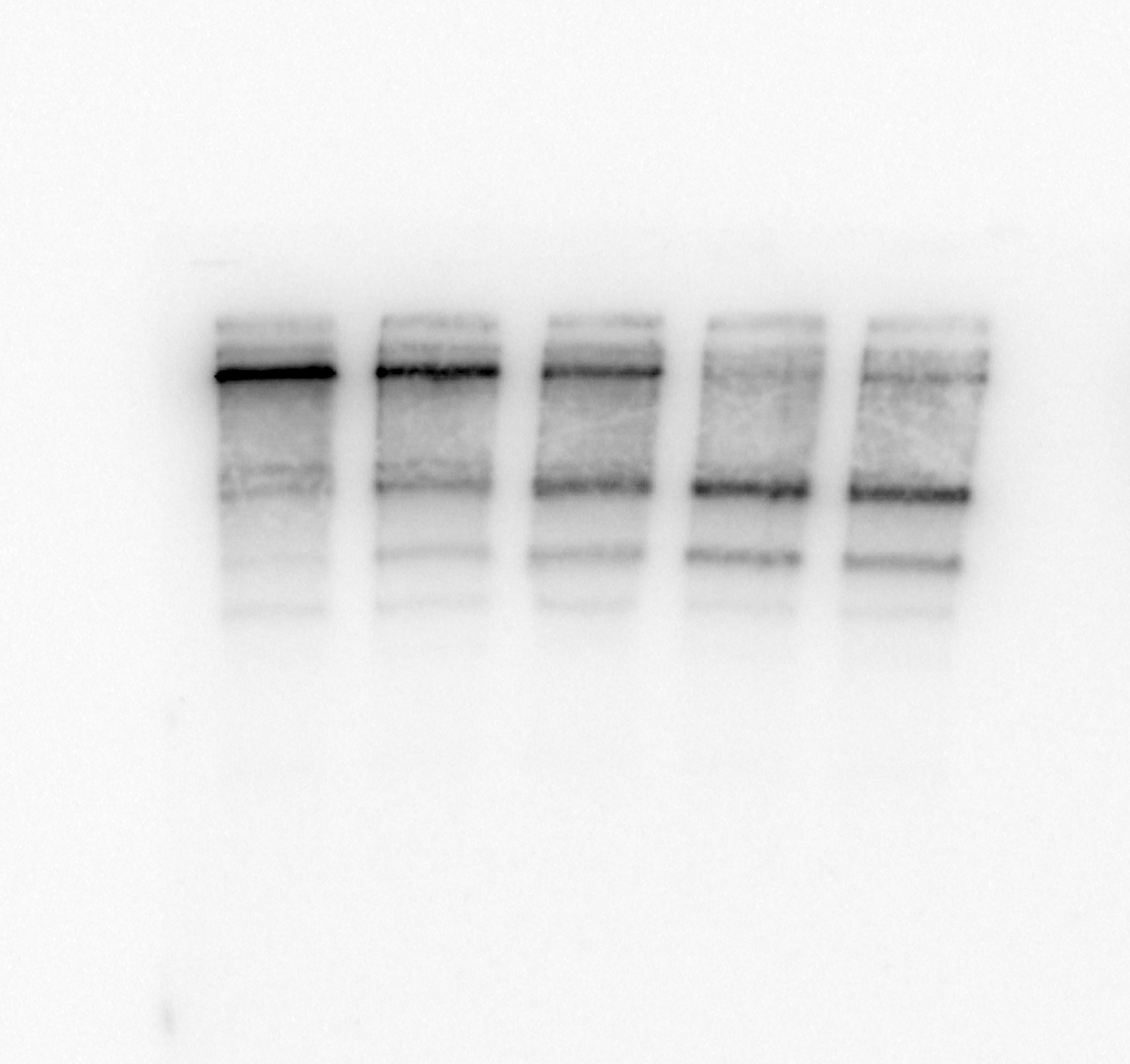

Supplement: Supplementary file 9 — Source Data for Figure 6 [file EMMM-15-e16775-s010.zip › Figure_6/Figure_6D.tif]

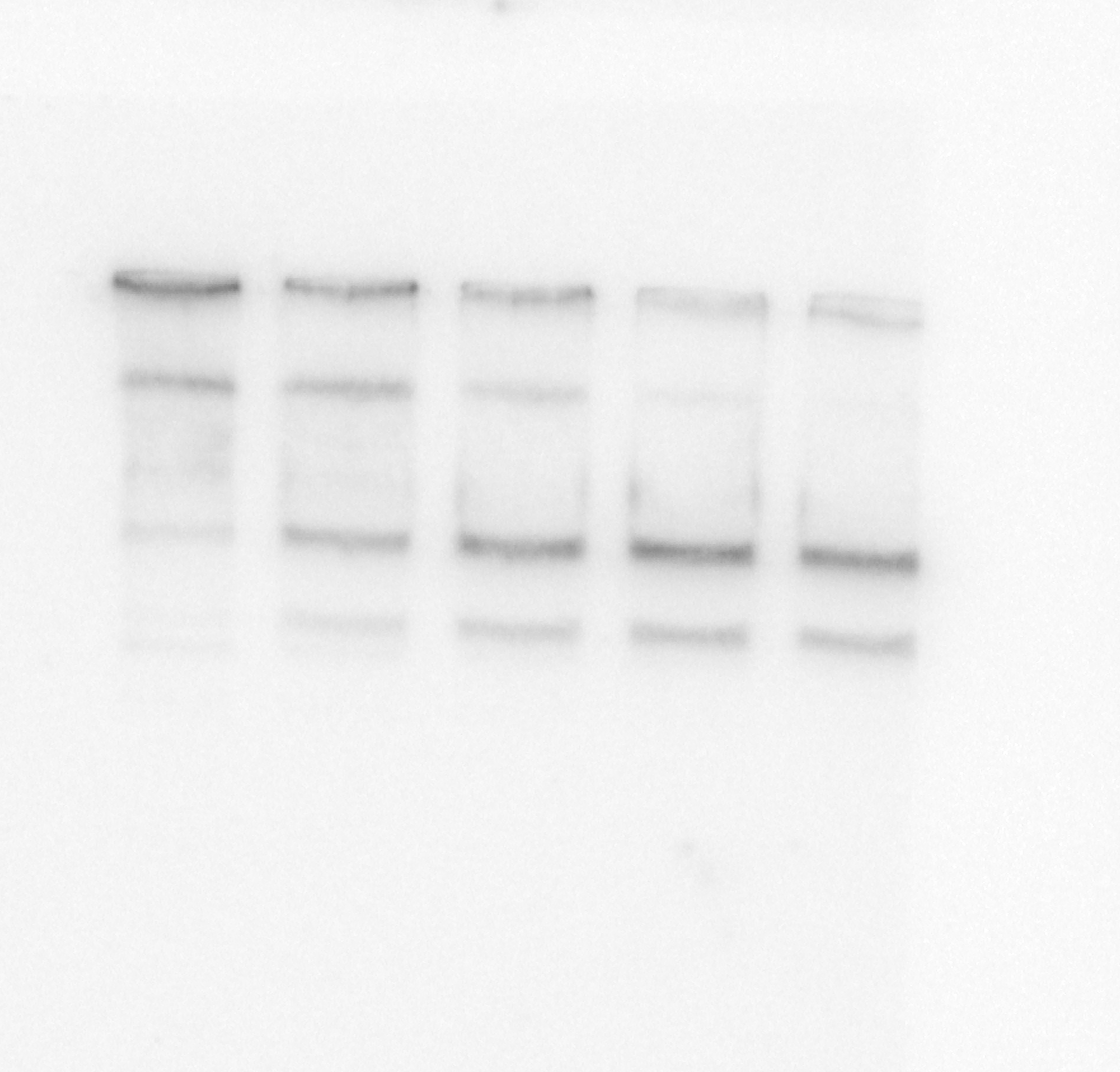

Supplement: Supplementary file 9 — Source Data for Figure 6 [file EMMM-15-e16775-s010.zip › Figure_6/Figure_6F.tif]

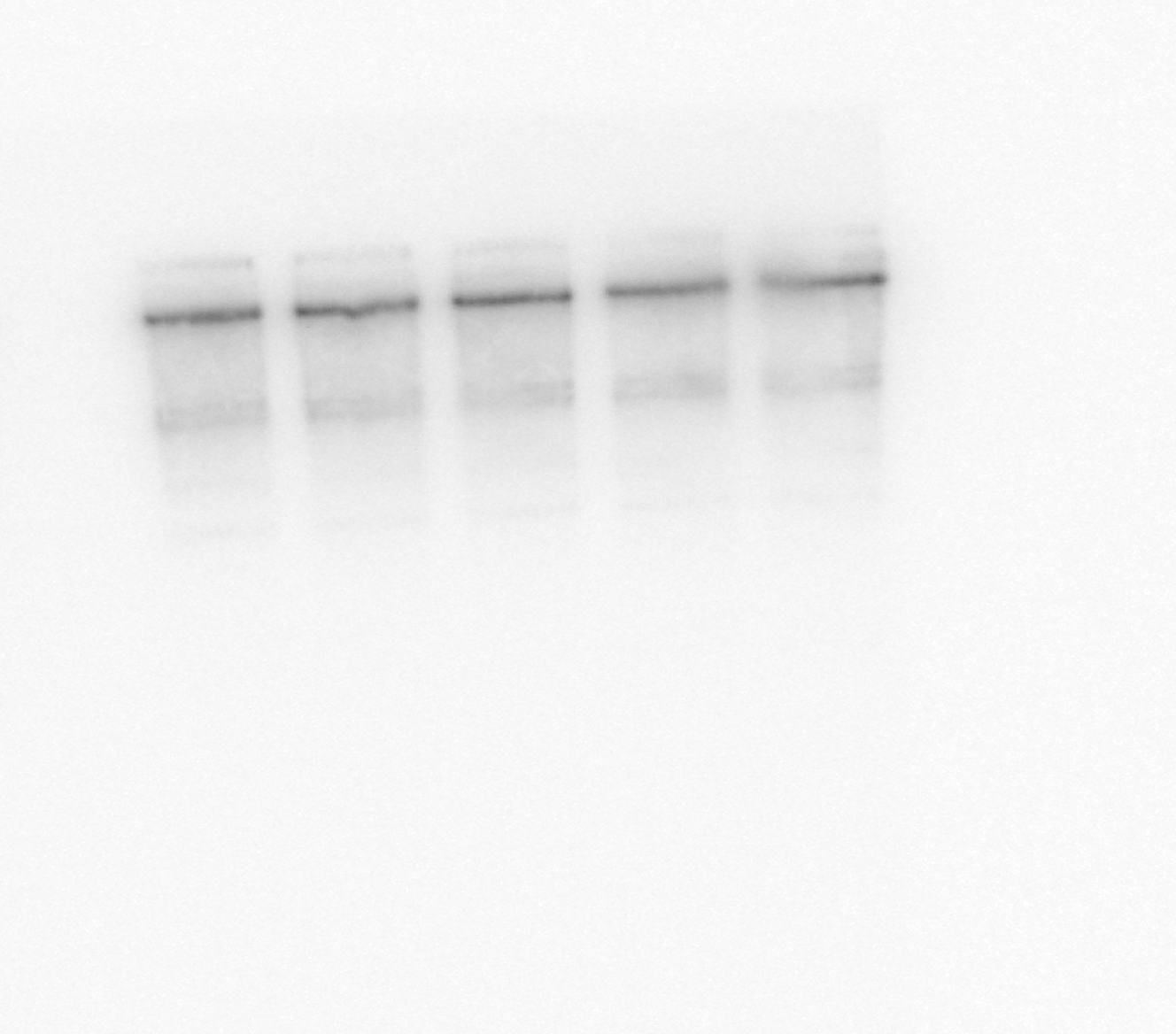

Supplement: Supplementary file 9 — Source Data for Figure 6 [file EMMM-15-e16775-s010.zip › Figure_6/Figure_6G.tif]

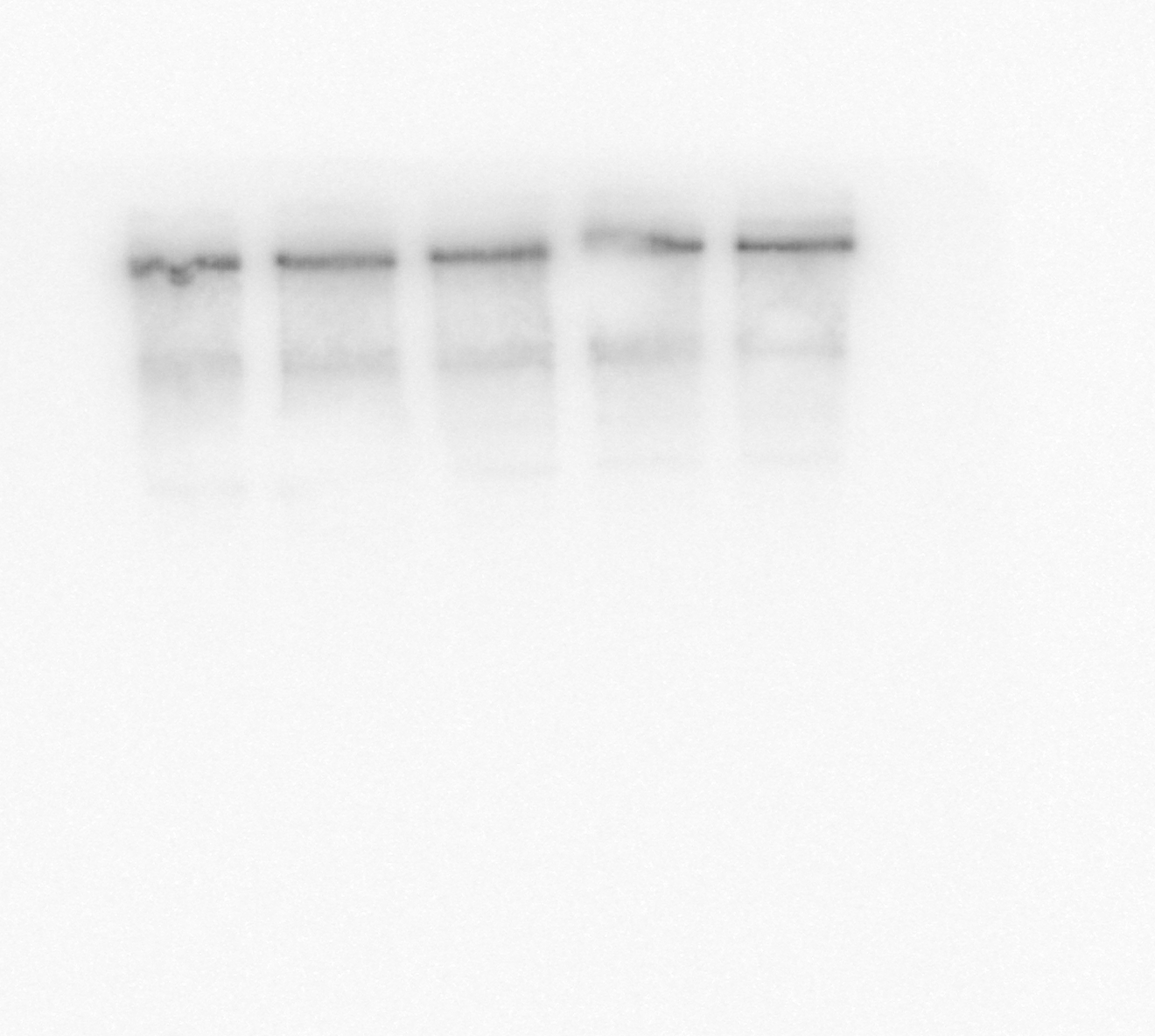

Supplement: Supplementary file 9 — Source Data for Figure 6 [file EMMM-15-e16775-s010.zip › Figure_6/Figure_6C.tif]

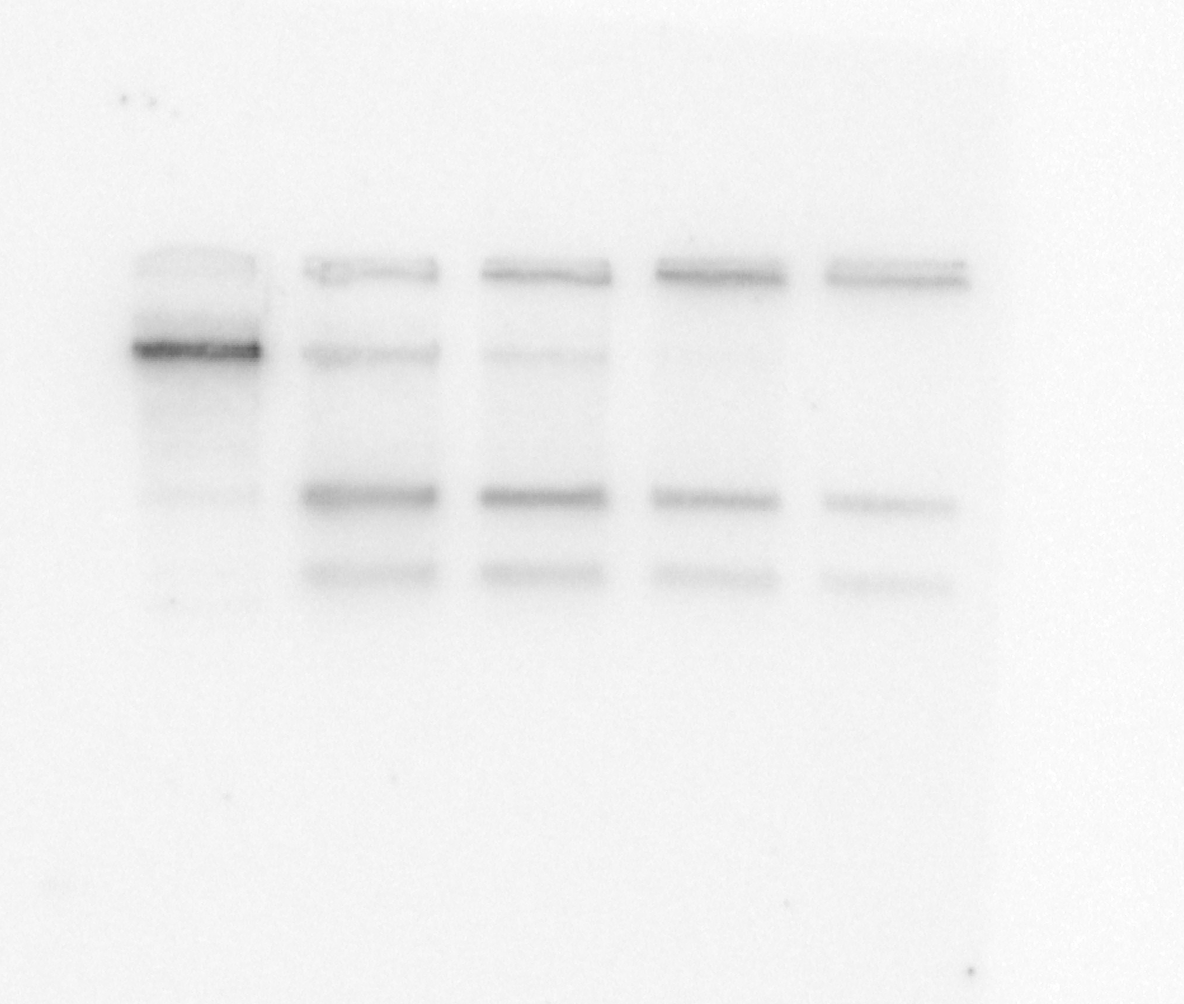

Supplement: Supplementary file 9 — Source Data for Figure 6 [file EMMM-15-e16775-s010.zip › Figure_6/Figure_6B.tif]
