# Supplementary material for: Pathological variants in TOP3A cause distinct disorders of mitochondrial and nuclear genome stability
Source: EMBO Mol Med. 2023 Apr 4;15(5):e16775. doi: 10.15252/emmm.202216775 (PMC10165364; doi:10.15252/emmm.202216775)
Supplement: Supplementary file 4 — PDF+ [file EMMM-15-e16775-s002.pdf]

SOURCE DATA TRANSPARENT PROCESS OPEN ACCESS

EMBO Molecular Medicine

Article

Pathological variants in *TOP3A* cause distinct disorders of mitochondrial and nuclear genome stability

Direnis Erdinc[1,†], Alejandro Rodríguez-Luis[2,3,†], Mahmoud R Fassad[2,4], Sarah Mackenzie[5], Christopher M Watson[6,7], Sebastian Valenzuela[1], Xie Xie[1], Katja E Menger[2,3], Kate Sergeant[8], Kate Craig[2,9], Sila Hopton[2,9], Gavin Falkous[2,9], Genomics England Research Consortium, Joanna Poulton[10], Hector Garcia-Moreno[11], Paola Giunti[11], Carlos A de Moura Aschoff[12], Jonas A Morales Saute[12,13,14], Amelia J Kirby[15], Camilo Toro[16], Lynne Wolfe[16], Danica Novacic[16], Lior Greenbaum[17,18,19], Aviva Eliyahu[17,19], Ortal Barel[20], Yair Anikster[19,21], Robert McFarland[2,4], Gráinne S Gorman[2,4], Andrew M Schaefer[2,9], Claes M Gustafsson[1,22], Robert W Taylor[2,4,9], Maria Falkenberg[1] & Thomas J Nicholls[2,3,*]

Abstract

Topoisomerase 3α (TOP3A) is an enzyme that removes torsional strain and interlinks between DNA molecules. TOP3A localises to both the nucleus and mitochondria, with the two isoforms playing specialised roles in DNA recombination and replication respectively. Pathogenic variants in *TOP3A* can cause a disorder similar to Bloom syndrome, which results from bi-allelic pathogenic variants in *BLM*, encoding a nuclear-binding partner of TOP3A. In this work, we describe 11 individuals from 9 families with an adult-onset mitochondrial disease resulting from bi-allelic *TOP3A* gene variants. The majority of patients have a consistent clinical phenotype characterised by bilateral ptosis, ophthalmoplegia, myopathy and axonal sensory-motor neuropathy. We present a comprehensive characterisation of the effect of *TOP3A* variants, from individuals with mitochondrial disease and Bloom-like syndrome, upon mtDNA maintenance and different aspects of enzyme function. Based on these results, we suggest a model whereby the overall severity of the TOP3A catalytic defect determines the clinical outcome, with milder variants causing adult-onset mitochondrial disease and more severe variants causing a Bloom-like syndrome with mitochondrial dysfunction in childhood.

Keywords Bloom syndrome; mitochondrial disease; mtDNA; TOP3A
Subject Categories Genetics, Gene Therapy & Genetic Disease; Organelles

1 Department of Medical Biochemistry and Cell Biology, University of Gothenburg, Gothenburg, Sweden
2 Wellcome Centre for Mitochondrial Research, Faculty of Medical Sciences, Newcastle University, Newcastle upon Tyne, UK
3 Biosciences Institute, Faculty of Medical Sciences, Newcastle University, Newcastle upon Tyne, UK
4 Translational and Clinical Research Institute, Faculty of Medical Sciences, Newcastle University, Newcastle upon Tyne, UK
5 The Newcastle Upon Tyne Hospitals NHS Foundation Trust, Newcastle upon Tyne, UK
6 North East and Yorkshire Genomic Laboratory Hub, Central Lab, St. James's University Hospital, Leeds, UK
7 Leeds Institute of Medical Research, University of Leeds, St. James's University Hospital, Leeds, UK
8 Oxford Genetics Laboratories, Oxford University Hospitals NHS Foundation Trust, Oxford, UK
9 NHS Highly Specialised Service for Rare Mitochondrial Disorders, Newcastle upon Tyne Hospitals NHS Foundation Trust, Newcastle upon Tyne, UK
10 Nuffield Department of Women's & Reproductive Health, The Women's Centre, University of Oxford, Oxford, UK
11 Department of Clinical and Movement Neurosciences, Ataxia Centre, UCL Queen Square Institute of Neurology, London, UK
12 Medical Genetics Service, Hospital de Clínicas de Porto Alegre (HCPA), Porto Alegre, Brazil
13 Department of Internal Medicine, Universidade Federal do Rio Grande do Sul, Porto Alegre, Brazil
14 Graduate Program in Medicine: Medical Sciences, Universidade Federal do Rio Grande do Sul, Porto Alegre, Brazil
15 Department of Pediatrics, Wake Forest School of Medicine, Winston-Salem, NC, USA
16 Undiagnosed Diseases Program, National Human Genome Research Institute, National Institutes of Health, Bethesda, MD, USA
17 The Danek Gertner Institute of Human Genetics, Sheba Medical Center, Tel Hashomer, Israel
18 The Joseph Sagol Neuroscience Center, Sheba Medical Center, Tel Hashomer, Israel
19 Sackler Faculty of Medicine, Tel Aviv University, Tel Aviv, Israel
20 Genomics Unit, The Center for Cancer Research, Sheba Medical Center, Tel Hashomer, Israel
21 Metabolic Disease Unit, Edmond and Lily Safra Children's Hospital, Sheba Medical Center, Tel Hashomer, Israel
22 Department of Clinical Chemistry, Sahlgrenska University Hospital, Gothenburg, Sweden
*Corresponding author. Tel: +44 01912086291; E-mail: thomas.nicholls@newcastle.ac.uk
[†]These authors contributed equally to this work

© 2023 The Authors. Published under the terms of the CC BY 4.0 license.

DOI 10.15252/emmm.202216775 | Received 19 August 2022 | Revised 10 March 2023 | Accepted 14 March 2023 | Published online 4 April 2023
EMBO Mol Med (2023) 15: e16775

Introduction

Mitochondrial diseases are a heterogeneous group of human disorders that can be caused by variants in either the mitochondrial genome (mtDNA) or the estimated 1,100 nuclear genes with protein products that play a role in mitochondrial function (Russell *et al*, 2020; Rath *et al*, 2021). All of the proteins required for the maintenance and expression of mtDNA are encoded in the nucleus and must be post-translationally imported into mitochondria in order to act upon mtDNA (Chacinska *et al*, 2009). Each human cell typically possesses several thousand copies of mtDNA (Filograna *et al*, 2021), which are replicated independently of the cell cycle in order to maintain a sufficient number of mtDNA copies per cell. Human mtDNA contains two canonical origins of DNA replication, termed OriH and OriL, and is replicated by an unusual asynchronous mechanism that results in a substantial delay between leading-strand synthesis from OriH and lagging-strand synthesis from OriL (Gustafsson *et al*, 2016). MtDNA is replicated by a dedicated protein machinery that includes the mtDNA polymerase POLG, the replicative helicase TWINKLE, the mitochondrial single-stranded DNA-binding protein mtSSB and the mitochondrial RNA polymerase POLRMT, which generates the primers for mtDNA replication (Gustafsson *et al*, 2016). Pathogenic variants in genes encoding mtDNA replication factors commonly manifest as an inability to maintain a sufficient number of copies of mtDNA per cell (termed mtDNA depletion) and/or the accumulation of deletions and point mutations in mtDNA. Clinically, these mtDNA maintenance disorders often exhibit a cluster of clinical features and ages of onset, from severe and progressive neurological disorders in childhood, such as Alpers syndrome and Leigh syndrome, to milder adult-onset disease such as progressive external ophthalmoplegia (PEO), depending on the severity and location of the variant (Gorman *et al*, 2016).

TOP3A is an enzyme that localises to both the nucleus and mitochondria. The *TOP3A* transcript contains two potential initiation codons, M1 and M26. Translation from the upstream start site generates a longer isoform bearing an N-terminal mitochondrial targeting sequence (MTS) that directs its import into mitochondria, while translation from the downstream initiation codon generates an N-terminally truncated protein that lacks the MTS and is directed for nuclear import (Wang *et al*, 2002; Wu *et al*, 2010; Tsai *et al*, 2016; Nicholls *et al*, 2018; Menger *et al*, 2022). Proteolytic cleavage of the MTS during mitochondrial import leads to mature mitochondrial and nuclear isoforms that are almost identical at the N-terminus (Wang *et al*, 2002). TOP3A is a topoisomerase; an enzyme that alters the topology of DNA by creating temporary breaks in the DNA backbone that can be used to remove topological stress or DNA interlinkages (Vos *et al*, 2011). Type I topoisomerases, such as TOP3A, break one strand of DNA thereby allowing the relaxation of negatively supercoiled DNA as well as the separation of hemicatenated molecules. In contrast, type II topoisomerases such as TOP2 create double-strand breaks, required for the catenation or decatenation of dsDNA molecules. In the nucleus, TOP3A forms a complex with the helicase BLM and the OB-fold proteins RMI1 and RMI2 to form the BTRR complex (Wu *et al*, 2000; Yin *et al*, 2005; Singh *et al*, 2008; Xu *et al*, 2008). This complex catalyses the processive removal of DNA interlinks from double Holliday junction containing recombination intermediates in a process termed dissolution (Raynard *et al*, 2006; Wu *et al*, 2006). Nuclear TOP3A also interacts with the DNA translocase PICH to induce positive DNA supercoiling, which is believed to facilitate the decatenation of sister chromatids by TOP2A during mitosis (Bizard *et al*, 2019). In contrast, the mitochondrial isoform of TOP3A appears to act independently of the BTRR complex, as there is no evidence that these nuclear-binding partners localise to mitochondria (Nicholls *et al*, 2018), and mitochondria also lack a system for homologous recombination (Hagstrom *et al*, 2014). Instead, the loss of mitochondrial TOP3A activity results in the accumulation of hemicatenated mitochondrial genomes, which are interlinked around the replication origin OriH (Nicholls *et al*, 2018). This indicates that the primary function of mitochondrial TOP3A is the separation of mtDNA replication products, with TOP3A acting to decatenate mtDNA either by the removal of precatenanes during replication and/or through hemicatenane resolution at replication completion (Nicholls *et al*, 2018; Hangas *et al*, 2022; Menger *et al*, 2022).

Loss-of-function variants in BLM, a binding partner of nuclear TOP3A, cause Bloom syndrome, a rare recessive disorder characterised by short stature, predisposition to cancer and a photosensitive (butterfly) rash, associated with an elevated rate of sister chromatid exchanges (Ellis *et al*, 1995). A number of individuals have recently been described with a Bloom-like disorder resulting from pathological variants in *TOP3A* (Martin *et al*, 2018; Jiang *et al*, 2021). Several of these patients also presented with cardiomyopathy, not typically associated with Bloom syndrome, and likely related to the additional mitochondrial role of TOP3A (Martin *et al*, 2018; Jiang *et al*, 2021). We previously reported a single patient with a complex adult-onset mitochondrial disorder characterised by PEO and prominent cerebellar features, but without evidence of Bloom syndrome, resulting from bi-allelic *TOP3A* gene variants (Nicholls *et al*, 2018); additional families have since been described (Llaurado *et al*, 2022; Primiano *et al*, 2022). Importantly, the genetic and biochemical factors that lead to these differing clinical phenotypes between TOP3A patients remain poorly understood. Here, in addition to the single case that we previously reported, we describe a further cohort of 10 individuals from 8 families with an adult-onset mitochondrial disease resulting from previously unreported bi-allelic variants in *TOP3A*. Using recombinant TOP3A variants with *in vitro* model substrates together with cellular rescue experiments, we characterise the molecular defects associated with mutated TOP3A, including variants found in patients with primary mitochondrial disease and a Bloom syndrome-like disorder. Our results provide insight into the molecular basis of disease in patients with distinct clinical syndromes resulting from variants in a single dual-targeted enzyme.

Results

Identification of patients with candidate pathogenic TOP3A variants

We identified 11 individuals from 9 families (Fig 1, and Tables 1 and 2; Appendix Supplementary Text) with clinical features strongly suggestive of a mitochondrial disorder and rare, damaging *TOP3A*

 © 2023 The Authors

variants (NM_004618.4) using a range of massively parallel sequencing strategies.

Seven of 11 patients first presented in the 5th or 6th decades of life. Four other patients (all women) presented in adolescence or their early 20s. Patients commonly manifested with bilateral ptosis and ophthalmoplegia (n = 9). The remaining two patients may have not yet manifested with an eye movement disorder due to their younger age (27 and 39 years old). Sensorineural hearing loss was present in seven patients including all four women who manifested with earlier-onset disease.

Myopathy was present in seven patients. Three affected individuals (Pa1, Pa3-1 and Pa5-1) manifested with proximal myopathy; two patients (Pa2 and Pa9) with mainly distal myopathy and muscle wasting and two patients (Pa6 and Pa8) with generalised muscle weakness. Affected individuals from all families manifested with mixed sensory motor axonal neuropathy except the two affected siblings of Family V. Cerebellar involvement, with or without evidence of sensory ataxia, was documented in seven patients.

Cardiac conduction defects necessitating pacemaker insertion occurred in five patients. Supraventricular tachycardia was reported in one patient (Pa3-2) who had left ventricular hypertrophy. MRI brain abnormalities were documented in six patients. Four patients shared features of cerebellar atrophy with evidence of brain stem and/or spinal cord atrophy. One patient (Pa4) showed bilateral high T2 signal in the basal ganglia and the brain stem. Another patient (Pa6) demonstrated extensive white matter changes consistent with small vessel ischaemic change. In a similar pattern to Bloom syndrome where malignancy manifests in early adulthood (Sugranes *et al*, 2022), one patient (Pa8) was diagnosed with cervical cancer in her early 30s.

All *TOP3A* variants identified were further confirmed by Sanger sequencing and, where parental and familial samples were available, segregation studies were performed, thus substantiating recessive inheritance (Fig 1A). To establish the phase of variants (the alleles from which variants are derived) in two of the families where familial samples were not available (Families V and VI), we performed low-coverage long-read whole-genome sequencing (Fig EV1). For Pa5-1 (*TOP3A* (NM_004618.5) variants c.298A>G and c.1723A>G), this yielded 11.42 Gb of sequence data comprising 8.76 million reads. While no single read spanned the 23 kb interval between the two target variants, an assembled haplotype could be created. Three reads (highlighted in brown, orange and blue in Fig EV1A) were linked through intervening single nucleotide polymorphisms identified from the short-read sequencing dataset (positions c.1282-21 and c.1468-11). Informative reads are variant supporting at position c.298 and reference matching at position c.1723, consistent with the interpretation that the target variants are arranged in *trans* (on different parental alleles). For the variants c.778C>T and c.1723A>G in Pa6, three MinION runs yielded 47.92 Gb of sequence data comprising 16.16 million reads. Again, no single read spanned the 17 kb interval between the target variants. However, an assembled haplotype could be constructed from two reads (coloured red and pink in Fig EV1F), which were linked by the absence of variant nucleotides at positions chr17:18195268 and chr17:18195287. The constructed haplotype was reference matching at position c.778 and variant supporting at position c.1723, consistent with the variants being arranged in *trans*.

Additional clinical details are provided in Table 2 and the Appendix Supplementary Text, and further details of the molecular variants are shown in Table 1. Two patients (Pa4 and Pa9) were homozygous for the p.Arg103Gln variant, while all other patients carry two heterozygous *TOP3A* variants. It is interesting to note that Pa5-1, Pa5-2 and Pa7 harbour the same p.Met100Val *TOP3A* variant that was identified in the first reported case (Pa1, Table 1; Nicholls *et al*, 2018). The p.Met575Val variant also appears to be recurrent and is shared by Pa2, Pa5-1 and 5-2, and Pa6.

Modelling of pathological TOP3A variants

With the exception of p.Met575Val and p.Arg558Trp, all identified missense variants are located within the Toprim (topoisomerase-primase) domain; a domain structure common to a number of nucleases and DNA repair proteins (Aravind *et al*, 1998) that contains the ssDNA-binding interface of TOP3A (Bocquet *et al*, 2014). The p.Met575Val and p.Arg558Trp variants are located in domain IV, to the side of the topoisomerase gate (Fig 1B). Sequence alignments of TOP3A homologues (Fig 1C) found that the sites of variants in TOP3A patients were conserved among mammals, with Leu37 and Met575 being more broadly conserved within metazoans. The p.Gln260* and p.Tyr300SerfsTer17 variants both result in truncation of the TOP3A protein before the essential active-site tyrosine residue (Tyr362) (Hanai *et al*, 1996), and so are assumed to represent loss-of-function variants and were not characterised further. The Ala176 locus, for which variants have been identified in Bloom syndrome (Martin *et al*, 2018) and which has been included in our study, is also located in the Toprim domain and is well conserved among metazoans. The truncating p.Ser810LeufsTer2 variant, also identified in patients with Bloom syndrome, removes almost 200 amino acids of the poorly characterised C-terminus of TOP3A, including predicted zinc finger motifs of unknown function.

Mapping of the affected loci onto the available crystal structure of TOP3A (PDB: 4CGY) indicated that variants in the Toprim domain were clustered close to the ssDNA-binding interface of the protein (Fig 1D) (Changela *et al*, 2007; Bocquet *et al*, 2014). For the residues in domain IV, the p.Arg558Trp variant is located close to the topoisomerase gate, while the p.Met575Val variant sits to the side of the gate, close to the C-terminus of the crystallised portion of TOP3A. Only the Arg558 residue is located in proximity to the binding interface with RMI1 (Fig EV2A), suggesting that the majority of variants should not affect this nuclear interaction.

The residues of interest were also analysed based on their intrastructural contacts (Fig 1E). The Leu37, Ala176 and Met575 residues are all coordinated in tight hydrophobic pockets, with variants likely to affect the integrity of these pockets. The p.Leu37Val variant is predicted to be unable to maintain interactions with a nearby helix (Fig 1E, panel i) which may impair stability or force a structural change to bridge the gap. For the p.Met575Val variant, the methionine side chain is normally coordinated deep into the hydrophobic pocket (Fig 1E, panel vii) with the valine side chain too short to form these interactions. Ala176 mainly interacts with Trp146 (Fig 1E, panel v) with a change to the bulkier valine causing direct clashes with Trp146, indicating that conformational adjustments are necessary to accommodate valine in the p.Ala176Val variant. Ala95, Met100 and Arg103 are all located on a hairpin-like region close to the ssDNA-binding groove (Changela *et al*, 2007; Bocquet

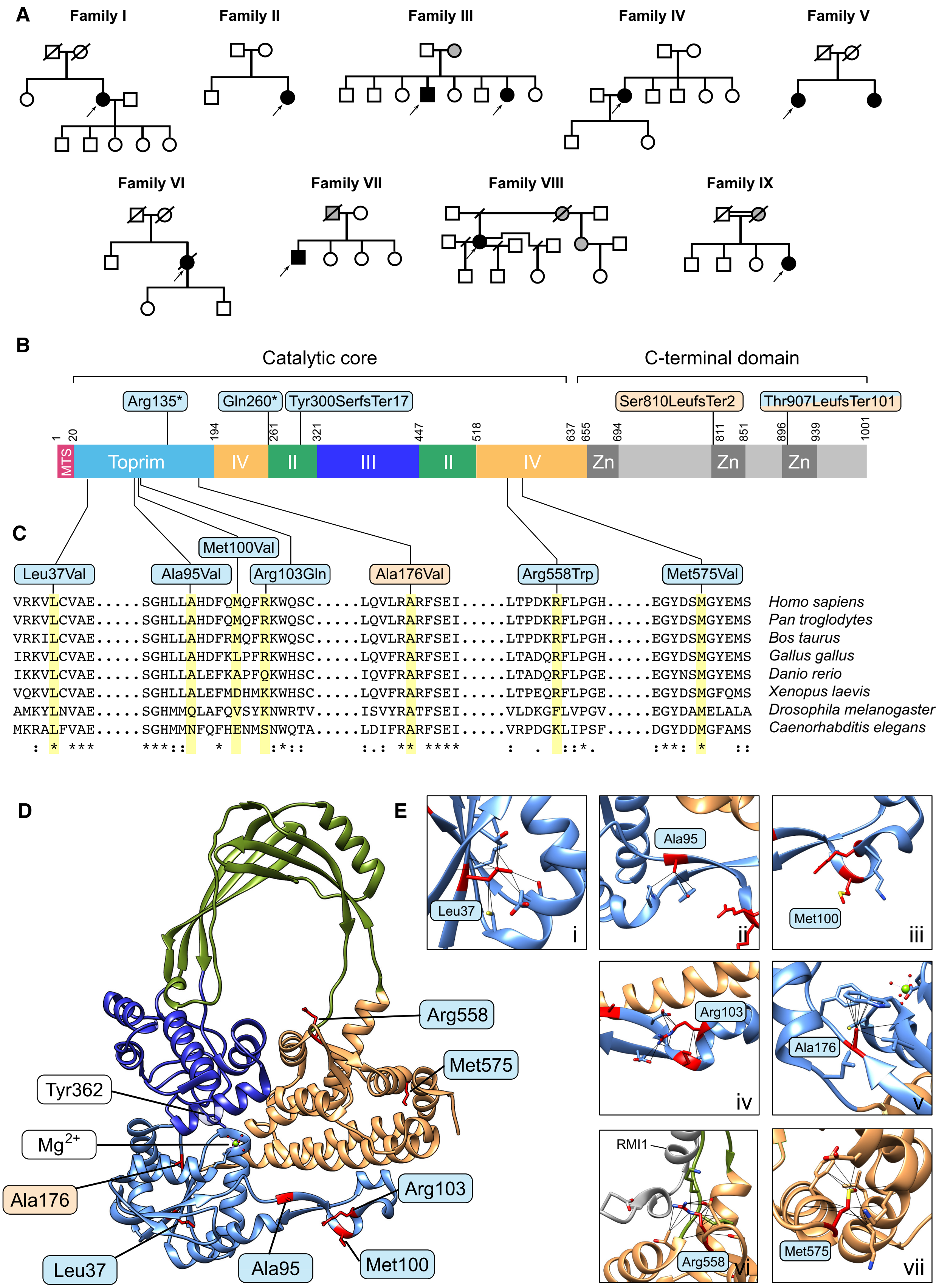

Figure 1.

© 2023 The Authors

Figure 1. Location and modelling of pathological TOP3A variants.

A Pedigrees and segregation of *TOP3A* variants in mitochondrial disease patients described in this study. Affected individuals are shown as filled shapes. Males are represented as squares, females are represented as circles and deceased individuals are indicated with diagonal lines. Arrows indicate the probands.

B Domain structure of the TOP3A protein. Domains are assigned according to Bocquet *et al* (2014). The locations of truncating variants in TOP3A are indicated above the diagram and missense variants are below the diagram. Variants found in mitochondrial disease patients are shown in blue, and variants found in Bloom syndrome that are included in this study are shown in orange.

C Sequence conservation of pathological TOP3A variants. Affected residues are highlighted in yellow, and sequence homology is indicated below the sequences.

D Location of pathological variants within the structure of TOP3A (PDB: 4CGY). Domains are coloured according to the assignments in (B). Affected residues are shown in red. The location of the catalytic tyrosine residue (Tyr362) and magnesium ion cofactor (Mg^{2+}) are also indicated. RMI1 is not shown.

E Enlarged images of affected residues as in (D). Interactions involving the side chains of these residues are depicted as black lines.

Table 1. TOP3A families in the study.

Patient ID/ Sex	Inheritance	cDNA[a]	Protein[a]	CADD[b]	Allele Frequency in gnomAD[c]	Reported previously
1/F	Recessive	c.298A>G;<br>c.403C>T	p.Met100Val;<br>p.Arg135*	20.4<br>N/A	0.00006432<br>0.00009901	Nicholls *et al* (2018)
2/F	Recessive	c.899_900delAT;<br>c.1723A>G	p.Tyr300Serfs*17;<br>p.Met575Val	N/A<br>23.6	0<br>0.00002122	VCV000636247.1<br>VCV000636248.1
3-1/M<br>3-2/F	Recessive	c.109C>G;<br>c.284C>T	p.Leu37Val;<br>p.Ala95Val	27.7<br>23.9	0<br>0	This study
4/F	Recessive	c.308G>A;<br>c.308G>A	p.Arg103Gln;<br>p.Arg103Gln	23.9<br>23.9	0.0001088<br>0.0001088	This study
5-1/F<br>5-2/F	Recessive	c.298A>G;<br>c.1723A>G	p.Met100Val;<br>p.Met575Val	20.4<br>23.6	0.00006432<br>0.00002122	This study
6/F	Recessive	c.778C>T;<br>c.1723A>G	p.Gln260*;<br>p.Met575Val	38<br>23.6	0<br>0.00002122	This study
7/M	Recessive	c.298A>G;<br>c.1672C>T	p.Met100Val;<br>p.Arg558Trp	20.4<br>25.3	0.00006432<br>0.00001194	This study
8/F	Recessive	c.2718del<br>c.240_240+2dup (intronic)	p.Thr907Leufs*101<br>N/A	N/A<br>14.3	0.000004373<br>0.000003978	VCV000560202.1<br>Martin *et al* (2018)
9/F	Recessive	c.308G>A;<br>c.308G>A	p.Arg103Gln;<br>p.Arg103Gln	23.9<br>23.9	0.0001088<br>0.0001088	This study

ND, Not determined.
[a]Variants described according to NM_004618.4 and GRCh37.
[b]CADD model GRCh37-v1.6.
[c]Heterozygous allele frequency taken from gnomAD v2.1.1; no homozygous occurrences of any variant.

et al, 2014). Alteration of Ala95 and Met100 do not cause obvious structural complications, although Met100Val has previously been found to impair TOP3A activity (Nicholls *et al*, 2018) (Fig 1E panels ii and iii). Arg103 interacts with multiple residues, forming two hydrogen bonds with the backbone oxygen of Phe98 (Fig 1E, panel iv) and potentially a salt bridge with Glu119. The Arg558 side chain forms multiple interactions with residues close to the topoisomerase gate (Fig 1E, panel vi), including a hydrogen bond with the backbone oxygen of Leu517 (~ 2.8 Å) and a salt bridge with Asp556 (~ 3.5 Å). The p.Arg558Trp variant likely destabilises this region due to loss of these interactions and clashes between the bulky tryptophan and surrounding residues. Furthermore, Arg558 is ~ 5.7 Å away from the Glu119 side chain of RMI1, and if the p.Arg558Trp variant induces structural complications in the region, interactions between TOP3A and RMI1 may be affected.

It should be noted that the truncated version of TOP3A used in the available crystal structure (Bocquet *et al*, 2014) omits approximately the C-terminal ~ 360 amino acids, containing a zinc finger domain, most likely due to high flexibility. It therefore cannot be determined whether this domain forms further relevant interactions. However, the zinc finger domain has been solved in structures of both *Escherichia coli* topoisomerase I (EcTOP1) and *Mycobacterium smegmatis* topoisomerase I (MsmTOP1). In these enzymes, the zinc finger domain binds to the intact strand of ssDNA in the strand passage reaction (T-strand), while the opposite strand (G-strand) is positioned at the active site for cleavage (Tan *et al*, 2015; Cao *et al*, 2020). The p.Met575Val variant is found close to the region, located at the junction between the catalytic core of TOP3A and the zinc finger domain (Fig 1E, panel vii), and may affect the communication between the N-terminal and C-terminal parts of TOP3A and how they interact with ssDNA.

The differences in clinical presentation between TOP3A patients led us to consider the potential presence of additional nuclear-modifying variants. Two major single nucleotide variants (SNPs)

Table 2. Summary of clinical phenotypes in TOP3A patients.

Family	I	II	III		IV	V		VI	VII	VIII	IX
**Patient**	**Pa1**	**Pa2**	**Pa3-1**	**Pa3-2**	**Pa4**	**Pa5-1**	**Pa5-2**	**Pa6**	**Pa7**	**Pa8**	**Pa9**
Sex	Female	Female	Male	Female	Female	Female	Female	Female	Male	Female	Female
Age at first presentation	43 years	Early 20s	Early 50s	Late 40s	16 years	59 years	59 years	52 years	54 years	21 years	15 years
Age at genetic diagnosis	67 years	39 years	64 years	59 years	27 years	72 years	66 years	Deceased at 72 years	71 years	44 years	41 years
TOP3A variants	p.Met100Val, p.Arg135*	p.Tyr300Serfs*17, p.Met575Val	p.Leu37Val, p.Ala95Val		p.Arg103Gln, p.Arg103Gln	p.Met100Val, p.Met575Val		p.Gln260*, p.Met575Val	p.Met100Val, p.Arg558Trp	p.Thr907Leufs*101, c.240_240 + 2dup	p.Arg103Gln, p.Arg103Gln
Ptosis & ophthalmoplegia	+	Ptosis only	+	+	–	+	+	+	+	+	+
Sensorineural hearing loss	+	+	+	–	+	–	–	+	NK	+	+
Myopathy	Proximal	Distal	Proximal	NK	–	Proximal	–	Generalised	–	Generalised	Distal
Axonal sensory motor Neuropathy	+	+	+	NK	+	–	–	+	+	+	+
Cerebellar Ataxia	+	+	+	+	–	–	–	+	+	–	+
Cardiac pacemaker/ ECG Abnormalities	PPM	PPM	NK	Supraventricular tachycardia	PPM	NAD	NAD	Episode of atrial flutter	NK	PPM	PPM
2D-Echocardiogram	Left ventricular dysfunction	Mitral valve prolapse	NK	Left ventricular hypertrophy	Normal	Normal	Normal	NK	NK	Moderately reduced LV systolic function, mildly dilated LV	Normal
MRI brain	Cerebellar atrophy with signal abnormalities in thalamus and brain stem	Cerebellar and moderate spinal cord atrophy	Cerebellar atrophy, T2 hyperintensities in globus pallidus, thalamus and brain stem	ND	Bilateral T2 hyperintensities in the globus pallidus, thalamus and brain stem.	NK	NK	Extensive white matter changes consistent with small vessel disease	Moderate cerebellar atrophy, with mild atrophy in the brainstem	NK	ND
Other morbidities	Pathogenic heterozygous *DSP* variant	—	Isolated seizures at age 56 years	Photosensitive epilepsy, myoclonus, dysphagia, dysarthria and premature menopause	—	Asthma, cholecystectomy, hypertension	Pancreatitis, cholecystectomy	—	Pigmentary retinopathy and Restless leg syndrome	Dysphagia requiring g-tube, dysarthria, cervical cancer	Premature menopause at age 28 years, Arachnoid cyst in the posterior cerebral fossa detected at age 17 years and seizures.
Family history	—	—	Mother was known to have epilepsy and deafness without ptosis or ophthalmoplegia		—	—		—	Father developed balance problems in early 80s.	Mother had pancreatic cancer. Maternal half-sister has cervical cancer in her 30s. Maternal grandmother had bowel cancer.	Mother had seizures until the age of 25, with complete remission. Maternal aunt had seizures.

NAD, No Abnormality Identified; ND, Not Done; NK, Not Known; PPM, Permanent Pacemaker.

have been reported for another mitochondrial topoisomerase, TOP1MT (Zhang *et al*, 2001); p.Val256Ile and p.Arg525Trp (Zhang *et al*, 2017). As these variants affect TOP1MT enzymatic activity (Zhang *et al*, 2017), and as TOP3A and TOP1MT appear to genetically interact (Nicholls *et al*, 2018; Menger *et al*, 2022), we assessed the presence of these variants in individuals within the TOP3A patient cohort where this information was available (Fig EV2B) but did not observe any clear correlation between the presence of TOP1MT SNPs and clinical severity.

OXPHOS deficiency and mtDNA instability in TOP3A patient muscle

Muscle biopsy samples were available from four patients, Pa2, Pa3-1, Pa4 and Pa6. Histopathological analyses using sequential COX-SDH histochemistry showed a variable but mosaic pattern of COX-deficient fibres (Fig 2A) with evidence of abnormal subsarcolemmal mitochondrial accumulation (ragged-red fibres). Quadruple immunofluorescence measurements of muscle biopsies from Pa3-1 and Pa6 additionally confirmed the presence of abundant fibres deficient in mitochondrial complex I (NDUFB8) and complex IV (COXI) proteins (Fig 2B). Given the role of TOP3A in mtDNA maintenance, we looked for the presence of mtDNA rearrangements using long-range PCR protocols, demonstrating the presence of multiple mtDNA deletions in all four available patient muscle samples (Fig 2C). To further characterise the mtDNA instability observed in TOP3A patient muscle, we used deep sequencing together with the MitoSAlt pipeline (Basu *et al*, 2020) to map mtDNA rearrangement breakpoints. Abundant major arc deletions, with a prominent breakpoint close to the termination-associated sequence (TAS) at the 3′ end of the mtDNA D-loop, were visible in muscle mtDNA from Pa3-1 and Pa5-1, comparable to deletion patterns reported previously in other mtDNA maintenance disorders (Zeviani *et al*, 1989; Persson *et al*, 2019). Analysis of muscle from Pa2 showed the presence of

 © 2023 The Authors

unusually small duplications in the mtDNA non-coding region (NCR) (Figs 2D and EV3A). A similar duplication has previously been detected in patients with mitochondrial myopathy (Brockington *et al*, 1993; Manfredi *et al*, 1995) as well as associated with certain mtDNA haplogroups (Torroni *et al*, 1994). As this region has also previously been mapped as the site of TOP3A activity (Nicholls *et al*, 2018), and is close to sites of mtDNA breakage upon overexpression of TOP3A (Hangas *et al*, 2022), the formation of this duplication may be promoted by aberrant TOP3A activity at this site. Other muscle samples showed the presence of abundant major arc mtDNA deletions (Fig 2E–G), comparable to those previously reported for Pa1 (Fig EV3B; Nicholls *et al*, 2018).

We additionally assessed structural alterations to patient mtDNA using Southern blotting. Because of the role of TOP3A in the separation of mtDNA, loss of TOP3A activity can lead to the accumulation of hemicatenated mtDNAs, which are highly stable and remain intact through restriction digestion and electrophoresis (Nicholls *et al*, 2018). We cleaved muscle DNA samples from four controls and four patients using BamHI (which cuts once in the mtDNA major arc) or PvuII (which cuts once in the mtDNA minor arc), separated products on an agarose gel and blotted for mtDNA. Samples from Pa2 and (particularly) Pa5-1 showed the presence of high-molecular-weight smears consistent with hemicatenated mtDNA replication products, as well as discrete bands that migrated either faster or slower than full-length mtDNA depending on the restriction enzyme used (Fig 3A and B). We further characterised these species from Pa2 and Pa5-1 by treatment with T7 endonuclease I (Fig EV3C), which cleaves hemicatenated mtDNA replication products (Nicholls *et al*, 2018). This treatment eliminated the high-molecular-weight smears, consistent with these representing branched hemicatenated molecules, but not the discrete bands, suggesting that these represent deletion-containing molecules that remove the minor arc PvuII restriction site (in samples from Pa2) or the major arc BamHI restriction site (in samples from Pa5-1).

Phenotypic rescue of mtDNA phenotypes by pathological TOP3A variants

In order to model the impact of pathological TOP3A variants upon mtDNA, we tested the capacity of protein variants to rescue mtDNA phenotypes associated with the loss of mitochondrial TOP3A activity. We generated U2OS cell lines that express siRNA-resistant versions of WT or pathological variant-containing TOP3A upon treatment with doxycycline. These cells were then depleted of endogenous TOP3A using siRNA while expressing the inducible TOP3A construct (Fig 3C). We were unable to detect the endogenous TOP3A using available antibodies in U2OS cells, but the depletion of TOP3A and the expression of the (non-tagged) Ser810* variant could be observed in parallel transfections of HeLa cells (Fig EV4A). The downregulation of TOP3A led to an approximately 75% loss of mtDNA copy number, which was rescued by the expression of WT TOP3A (Fig 3D). This copy number loss was also rescued by expression of the TOP3A variants Leu37Val, Ala95Val and Met100Val, while the expression of all other variants failed to rescue mtDNA copy number loss to different extents (Fig 3D). We additionally assessed the effects of TOP3A variants upon mtDNA structure and topology using Southern blotting. Depletion of TOP3A caused an accumulation of high-molecular-weight hemicatenated mtDNA that was retained in the well during electrophoresis, as described previously (Nicholls *et al*, 2018), which is eliminated by the expression of WT TOP3A (Fig 3E, lanes 2–3). High-molecular-weight mtDNA remained visible following expression of several pathological TOP3A variants, particularly Leu37Val, Arg558Trp and the two Bloom syndrome-associated variants Ala176Val and Ser810LeufsTer2 (expressed as Ser810*) (Fig 3E). Different topological forms of mtDNA can be distinguished by separation of uncut mtDNA on low-percentage agarose gels, with TOP3A depletion resulting in a loss of monomeric forms of mtDNA (Fig 3F, lane 4). This phenotype could again be rescued by expression of WT TOP3A (Fig 3F, lane 5) but only partially rescued by the expression of several pathological variants, including Arg103Gln, Arg558Trp, Met575Val and the Bloom syndrome-associated variants Ala176Val and Ser810* (Fig 3F, lanes 6–13).

DNA-binding activity of pathological TOP3A variants

During the catalytic cycle of TOP3A, the protein binds to a single-stranded (ssDNA) substrate, cleaves the DNA to create a break, then passes an intact DNA strand through this break and re-seals the broken strand (Bizard & Hickson, 2020). When the broken and passaged strands originate from a single negatively supercoiled DNA molecule, the result is relaxation of the substrate DNA. In contrast, if the two strands are from different ssDNA molecules, then the result is the linking (catenation) or unlinking (decatenation) of the molecules (Bizard & Hickson, 2020).

In order to characterise the effects of pathological variants upon different aspects of TOP3A function, we used Sf9 insect cells to express and purify wild-type (WT) TOP3A, as well as all missense variants found in mitochondrial disease patients reported here. We additionally expressed and purified the p.Ala176Val missense variant and a p.Ser810LeufsTer2 truncating variant (purified as p.Ser810*) found in patients with a Bloom syndrome-like disorder (Martin *et al*, 2018). The purity of the recombinant proteins was confirmed using SDS–PAGE (Fig 4A).

We first analysed the DNA-binding ability of TOP3A variants, by incubating purified TOP3A protein with a radiolabelled 80 nt ssDNA oligonucleotide in an electrophoretic mobility shift assay (EMSA). The incubation of labelled ssDNA with an increasing concentration of WT purified TOP3A resulted in a pronounced shift, confirming the DNA-binding capacity of the WT protein (Fig 4B). The two shifted bands observed reflect the capacity of two molecules of TOP3A to bind to a single 80 nt ssDNA oligo (Fig EV4B). We then assessed the DNA-binding capacity of all pathological variants of TOP3A. The p.Leu37Val (Fig 4C) and p.Met575Val (Fig 4I) variants of TOP3A, found in the cohort of mitochondrial disease patients, were unable to gel shift the labelled oligonucleotide, indicating a significant loss of DNA-binding activity (Fig 4K). In contrast, all other missense variants (Fig 4D–H), as well as the p.Ser810* truncating variant (Fig 4J), showed a comparable level of DNA-binding activity to the WT protein (Fig 4K).

Relaxation activity of pathological TOP3A variants

Type IA topoisomerases catalyse the relaxation of negatively supercoiled DNA via intramolecular strand passage reactions. These reactions are typically inefficient as TOP3A requires regions of ssDNA in order to bind and cleave, although some studies have used

© 2023 The Authors

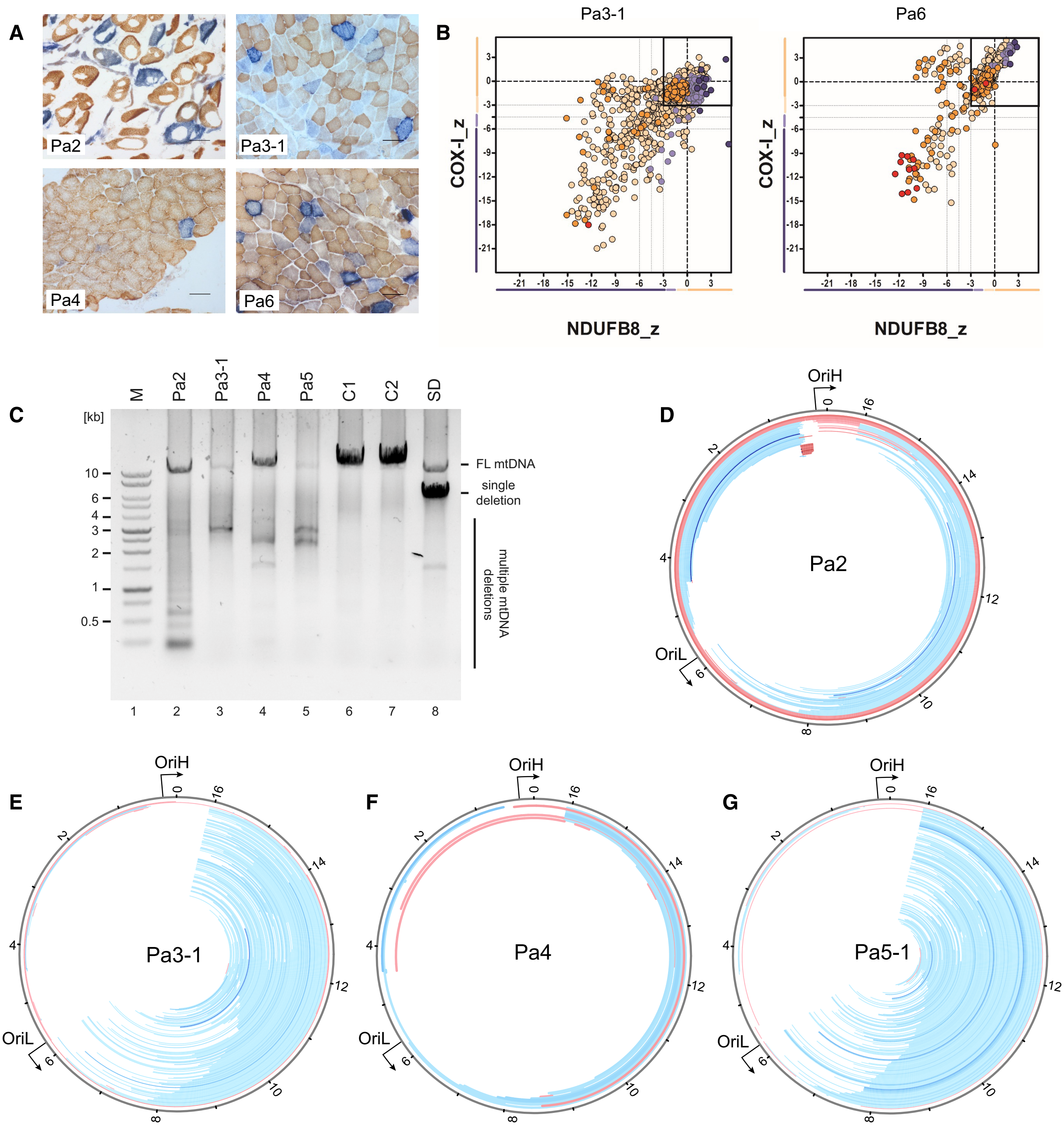

Figure 2. Pathological phenotypes in muscle of individuals with TOP3A-related mitochondrial disease.

A Sequential COX-SDH histochemical staining in muscle biopsies of Pa2, Pa3-1, Pa4 and Pa6 (as indicated), indicating a mosaic pattern of COX deficiency. Scale bar represents 100 μm.

B Quadruple immunofluorescence assay to detect deficiency in complex I (NDUFB8) and complex IV (COXI) in Pa3-1 (left) and Pa6 (right).

C Long-range PCR assay to detect mtDNA deletions in TOP3A patient muscle (lanes 2–5). Samples from unaffected individuals (lanes 6 and 7) and a single-deletion mitochondrial disease patient (lane 8) are used as negative and positive controls respectively. "M" indicates marker.

D–G Mapping of mtDNA rearrangements in TOP3A mitochondrial disease patients. Total DNA samples from Pa2 (D), Pa3-1 (E), Pa4 (F) and Pa5 (G) were analysed by whole-genome sequencing, and the data were processed using the MitoSAlt pipeline. Deleted regions are shown as blue bars and predicted duplicated regions as red bars. The intensity of the colour corresponds to the abundance of the rearrangement.

Source data are available online for this figure.

 © 2023 The Authors

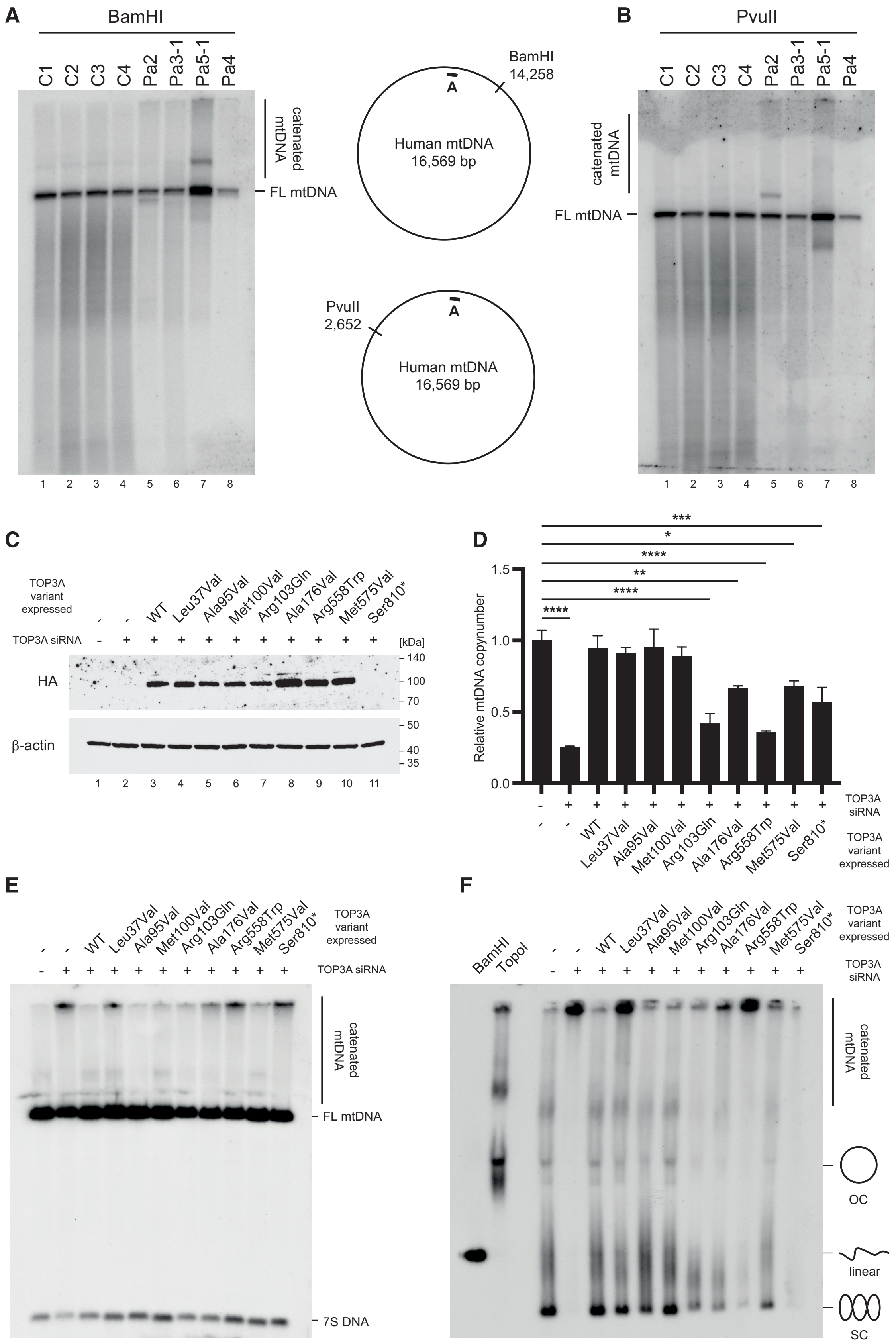
A
BamHI
C1 C2 C3 C4 Pa2 Pa3-1 Pa5-1 Pa4
catenated mtDNA
FL mtDNA
BamHI 14,258
Human mtDNA 16,569 bp
PvuII 2,652
Human mtDNA 16,569 bp
B
PvuII
C1 C2 C3 C4 Pa2 Pa3-1 Pa5-1 Pa4
catenated mtDNA
FL mtDNA
C
TOP3A variant expressed
WT Leu37Val Ala95Val Met100Val Arg103Gln Ala176Val Arg558Trp Met575Val Ser810*
TOP3A siRNA
[kDa]
HA
β-actin
D
Relative mtDNA copynumber
TOP3A siRNA
TOP3A variant expressed
E
TOP3A variant expressed
TOP3A siRNA
catenated mtDNA
FL mtDNA
7S DNA
F
BamHI TopoI
TOP3A variant expressed
TOP3A siRNA
catenated mtDNA
OC
linear
SC

Figure 3.

◀ **Figure 3. Effects of pathological TOP3A variants upon mtDNA structure and copy number.**

A, B Analysis of mtDNA structure in muscle from individuals with TOP3A-related mitochondrial disease using Southern blotting following restriction with BamHI (A) or PvuII (B). The black bar indicates the probe (nt. 16262-128).

C–F mtDNA phenotypes in U2OS Flp-In cells treated with TOP3A siRNA, rescued using variant-containing siRNA-resistant forms of TOP3A. (C) Western blot of TOP3A protein expression. Cells were untransfected (lane 1) or transfected with TOP3A siRNA (lanes 2–11), then induced to express WT TOP3A (lane 3) or pathological variants of TOP3A (lanes 4–11). Note that TOP3A Ser810* does not carry a C-terminal HA tag. β-actin is used as a loading control. (D) mtDNA copy number for TOP3A rescue cells as in (C) determined using qPCR. Error bars represent SEM, $n = 3$ (Ala95Val) or $n = 4$ (all other samples), where each data point represents the mean of three technical triplicates. Significance values are shown for one-way ANOVA compared to untreated wild-type cells, *$P < 0.05$, **$P < 0.01$, ***$P < 0.001$, ****$P < 0.0001$. (E) Southern blot of mtDNA structure from cells as in (C). The probe (black bar, nt. 16262-128) detects both full-length (FL) mtDNA and 7S DNA. The presence of hemicatenated mtDNAs is indicated. (F) Southern blot of uncut mtDNA to visualise different topological forms, from cells as in (C). Control DNA treated with BamHI (lane 1) or *E. coli* topoisomerase I (lane 2) acts as markers for linear and relaxed monomeric mtDNA respectively. Diagrams indicate the migration of different mtDNA forms.

Source data are available online for this figure.

conditions that promote an ssDNA conformation of the substrate such as bubble formation (Plank *et al*, 2005), elevated temperatures and low salt concentrations (Chen & Brill, 2007). We assessed the activity of TOP3A variants upon a negatively supercoiled pUC19 dsDNA plasmid substrate. The incubation of this substrate with increasing concentrations of purified TOP3A generated products that migrated similarly to those produced by the *E. coli* type IA topoisomerase TopoI (Fig 5A). In contrast, very little activity was observed using any of the pathological TOP3A variants analysed, with the exception of p.Met100Val, suggesting that the ability of TOP3A to processively catalyse strand passage reactions is impaired (Fig 5B–J). Covalently closed and relaxed dsDNAs migrate similarly on ethidium bromide (EtBr)-free agarose gels. The addition of EtBr to the gel allows these molecules to be distinguished by inducing supercoiling in covalently closed, but not nicked, molecules (Fig EV4C and D). The separation of WT TOP3A reaction products on EtBr-containing gels (Fig EV4E), indicated that a substantial proportion of these reaction products constituted nicked molecules. However, as these nicked products were not observed with other variants, we assume this to represent unligated products generated by the nicking/closing activity of the WT TOP3A protein (Cejka *et al*, 2012).

ssDNA decatenation activity of pathological TOP3A variants

We lastly investigated the ability of TOP3A variants to process single-stranded DNA catenanes and supercoiled DNA substrates. Because mitochondrial TOP3A plays a role in the separation of hemicatenated mtDNA replication products (Nicholls *et al*, 2018; Hangas *et al*, 2022), we sought to create substrates that would more closely mimic this *in vivo* activity. We used a two-holder strategy to create ssDNA catenanes composed of two interlocked circular ssDNAs (Li *et al*, 2018). To synthesise these substrates, two ssDNA oligonucleotides that share a short region of homology (R1 and R2) are held in place by holder strands (H1 and H2), while the free ends are brought together by short splint oligonucleotides (S1 and S2). The free ends are ligated together, and then the interlocked products are denatured and purified (Fig 6A). The products of this reaction have a linking number of 1, meaning that they can be resolved by a single catalytic cycle of TOP3A (Fig 6A) and so constitute the simplest substrate for assaying TOP3A decatenation activity. The identity of the purified synthesis products was confirmed through their resistance to degradation by the exonuclease ExoI and by their susceptibility to degradation by S1 nuclease and the restriction enzyme CviKI-1 (Fig EV5A and B). We then used these substrates to assay the decatenation activity of TOP3A. Decatenation reactions were performed in the absence of RMI1, as although RMI1 has been found to promote the decatenation activity of TOP3A (Yang *et al*, 2010; Bocquet *et al*, 2014), it does not localise to mitochondria (Nicholls *et al*, 2018; Rath *et al*, 2021), suggesting that RMI1 is not required for the mitochondrial activity of TOP3A. The incubation of ssDNA catenanes with increasing concentrations of purified WT TOP3A resulted in the release of individual circular ssDNAs (Fig 6B), indicating that the WT TOP3A protein can efficiently decatenate this substrate. Equivalent reactions using pathological variant-containing forms of TOP3A found that the p.Leu37Val (Fig 6C) and p.Met575Val (Fig 6I) variants found in the mitochondrial patient cohort, as well as the p.Ala176Val variant (Fig 6G) found in Bloom syndrome, showed a marked loss of decatenation activity. Other missense variants of TOP3A (p.Ala95Val, p.Met100Val, p.Arg103Gln and p.Arg558Trp; Fig 6D–F and H) showed a level of decatenation activity that was comparable to the WT protein (Fig 6K). The decatenation activity of the p.Ser810* truncating variant of TOP3A was also found to be comparable to the WT protein (Fig 6J–K), indicating that this C-terminal truncation of the protein does not affect the decatenation activity of this variant. This appears consistent with the finding that the catalytic core of TOP3A is sufficient for Holliday junction dissolution (Bocquet *et al*, 2014), and also with the notion that the pathology associated with this truncating variant is the result of the depletion of protein levels *in vivo* rather than because of a loss of activity (Martin *et al*, 2018). Given that three families carry two different segregating heterozygous missense TOP3A variants, we performed additional ssDNA decatenation assays using equimolar mixtures of the variants identified in these families. These combinations of variants displayed an intermediate level of activity relative to the single variants alone (Fig 6L and M) and confirms that patients with *TOP3A*-related mitochondrial disease in this cohort retain at least one allele that displays a detectable level of activity.

Discussion

Dual-localised DNA-interacting proteins such as TOP3A present a clinical and diagnostic challenge, as defects in enzymatic activity can potentially affect the maintenance and stability of either the nuclear genome or the mitochondrial genome to produce distinct clinical outcomes.

© 2023 The Authors

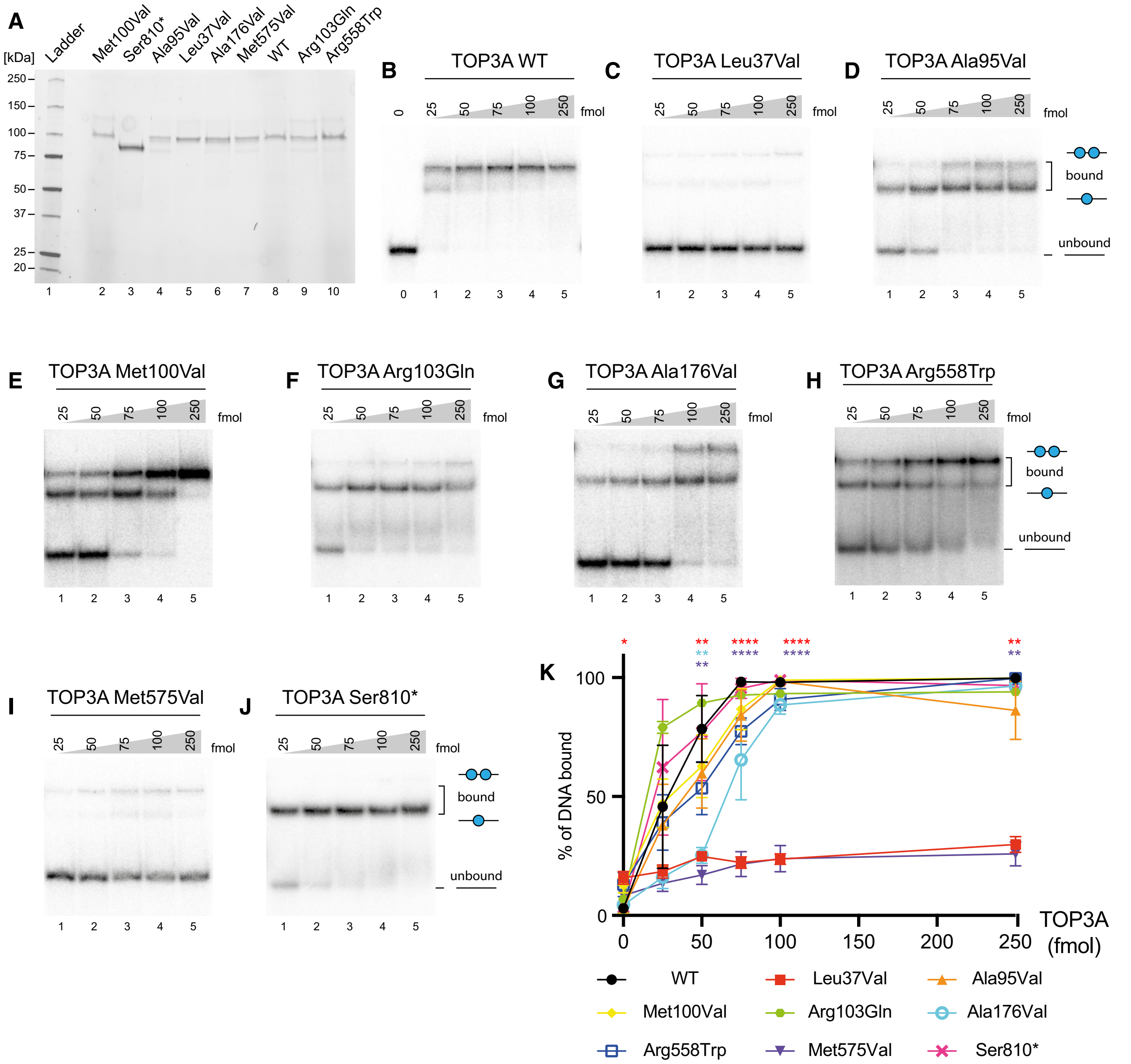

Figure 4. Purification of TOP3A variants and DNA-binding assays.

A Purification of TOP3A variants. Each variant (3 pmol) was separated on a 4–20% stain-free Criterion TGX SDS–PAGE gel (Bio-Rad) and imaged using a stain-free imager.

B–J Electrophoretic mobility shift assay (EMSA) to assess DNA-binding activity of TOP3A variants to a 5′ radiolabelled 80-nt-long ssDNA oligonucleotide.

K Quantification of DNA-binding activity as in (B–J), expressed as the proportion of bound DNA. Data represent mean values with at least three independent experiments per data point. Error bars represent SEM. Symbols represent significance values (one-way ANOVA) and are colour coded to the data points. *$P < 0.05$, **$P < 0.01$, ****$P < 0.0001$.

Source data are available online for this figure.

The majority of the core proteins of the mtDNA replication and expression machinery are dedicated mitochondrial proteins, with pathological variants resulting solely in mitochondrial disease phenotypes, including POLG (Van Goethem *et al*, 2001), POLG2 (Longley *et al*, 2006), TWINKLE (Spelbrink *et al*, 2001), MGME1 (Kornblum *et al*, 2013) and POLRMT (Olahova *et al*, 2021). However, variants have also been described in the dual-localised nucleases RNASEH1 (Reyes *et al*, 2015) and DNA2 (Ronchi *et al*, 2013) that result in mitochondrial disease phenotypes. The cohort of individuals with mitochondrial disease resulting from pathological

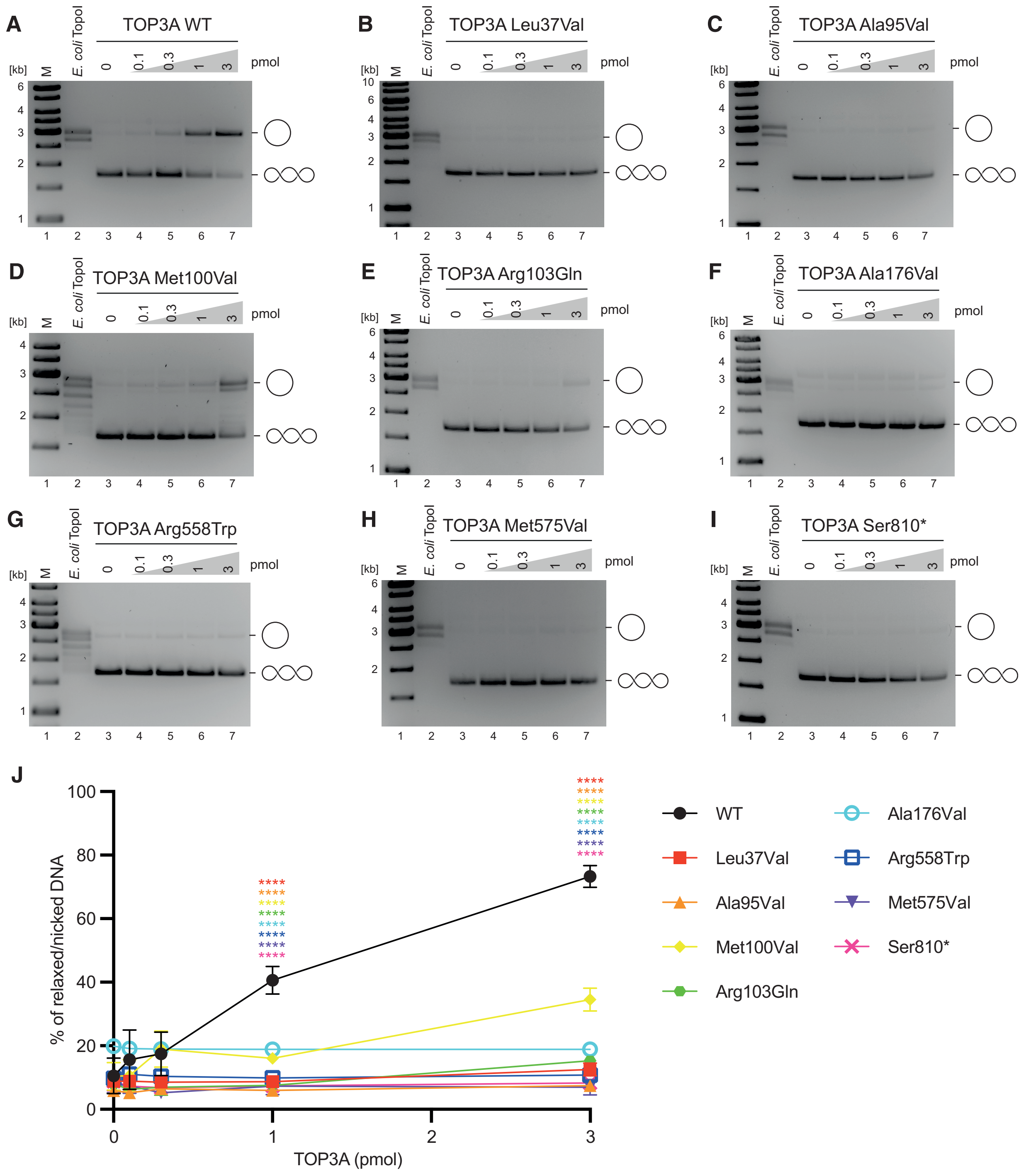

Figure 5. DNA relaxation activity of TOP3A variants.

A–I Relaxation of negatively supercoiled pUC19 substrate by recombinant TOP3A variants. "M" indicates marker.

J Quantification of DNA relaxation data as in (A–I). Data represent the percentage of substrate released from negatively supercoiled form, expressed as the mean of three independent experiments (except WT, for which $n = 4$). Error bars represent SEM. Symbols represent significance values (one-way ANOVA) and are colour coded to the data points. ****$P < 0.0001$.

Source data are available online for this figure.

 © 2023 The Authors

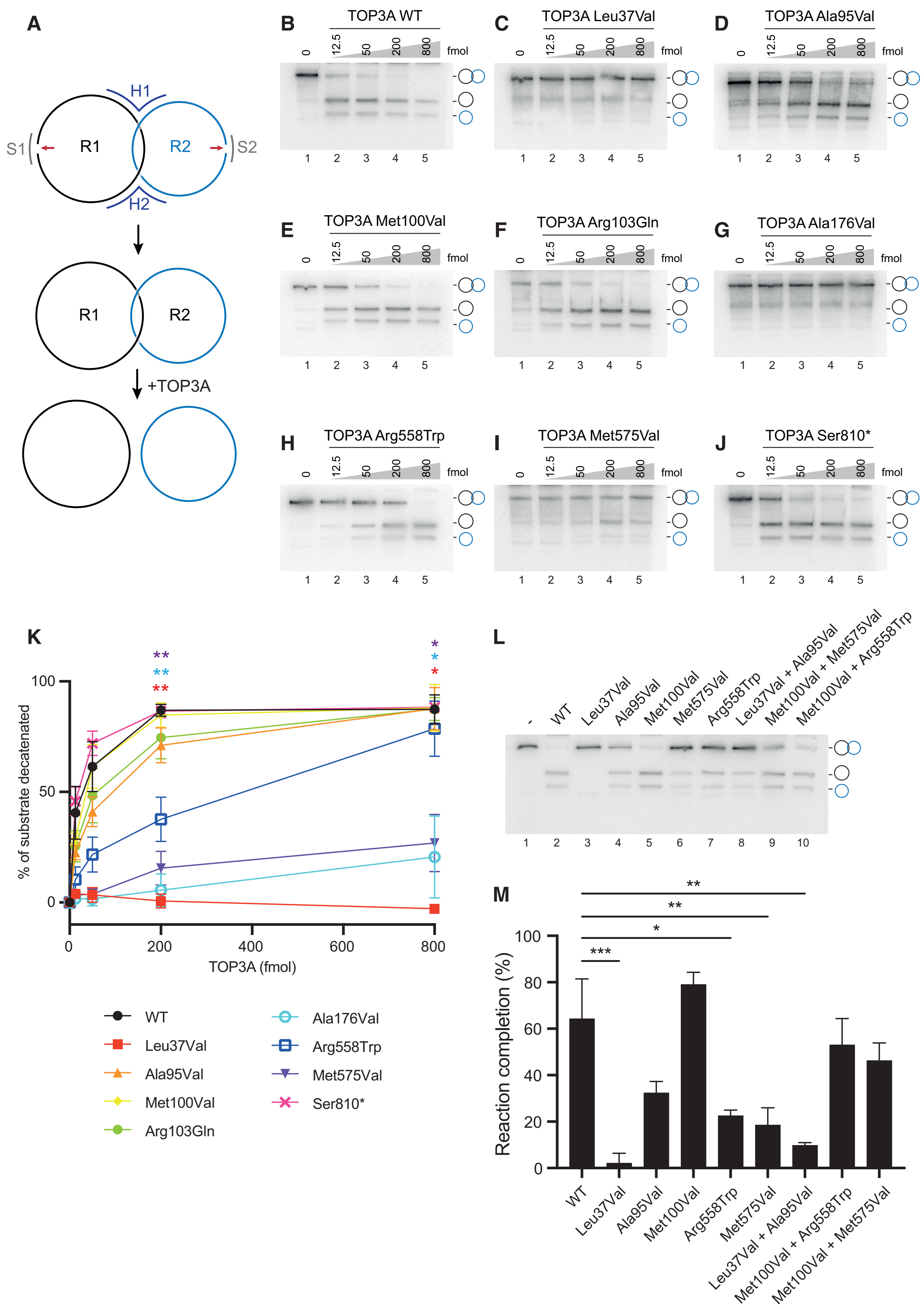
A
H1
S1
R1
R2
S2
H2
R1
R2
+TOP3A
B
TOP3A WT
C
TOP3A Leu37Val
D
TOP3A Ala95Val
E
TOP3A Met100Val
F
TOP3A Arg103Gln
G
TOP3A Ala176Val
H
TOP3A Arg558Trp
I
TOP3A Met575Val
J
TOP3A Ser810*
0 12.5 50 200 800 fmol
1 2 3 4 5
K
% of substrate decatenated
TOP3A (fmol)
WT
Leu37Val
Ala95Val
Met100Val
Arg103Gln
Ala176Val
Arg558Trp
Met575Val
Ser810*
L
-
WT
Leu37Val
Ala95Val
Met100Val
Met575Val
Arg558Trp
Leu37Val + Ala95Val
Met100Val + Met575Val
Met100Val + Arg558Trp
1 2 3 4 5 6 7 8 9 10
M
Reaction completion (%)
WT
Leu37Val
Ala95Val
Met100Val
Arg558Trp
Met575Val
Leu37Val + Ala95Val
Met100Val + Arg558Trp
Met100Val + Met575Val

Figure 6.

Figure 6. Assessment of decatenation activity of TOP3A variants.

A Schematic of substrate construction for ssDNA decatenation assays. The two ring oligos (R1 and R2) are held in an Lk1 conformation by holder oligos (R1 and R2) and ligated (red arrows) with the aid of splint oligos (S1 and S2), and then purified. Incubation with TOP3A decatenates this substrate into two independent circular ssDNA oligos.

B–J ssDNA decatenation assays using TOP3A variants, using substrates constructed as in (A).

K Quantification of ssDNA decatenation activity as in (B–J). Data represent the percentage of decatenated substrate expressed as mean values, ±SEM, from independent experiments, $n = 3$ (WT, p.Ala95Val, p.Met100Val and p.Arg558Trp), $n = 4$ (p.Ala176Val), $n = 5$ (p.Met575Val) and $n = 2$ (p.Leu37Val). Symbols represent significance values (one-way ANOVA) and are colour coded to the data points. *$P < 0.05$, **$P < 0.01$.

L ssDNA decatenation assays using combinations of compound heterozygous TOP3A variants. Reactions contained 50 fmol of protein (for single variants, lanes 2–7) or 25 fmol for each of two variants (lanes 8–10).

M Quantifications of ssDNA decatenation assays as in (L). Data represent the mean percentage of decatenated substrate from three independent experiments. Significance values are shown for one-way ANOVA compared to the WT protein. *$P < 0.05$, **$P < 0.01$, ***$P < 0.001$.

Source data are available online for this figure.

variants in *TOP3A* complements a previous report of variants in the same gene causing Bloom syndrome, primarily resulting from impaired function of the nuclear isoform of TOP3A (Martin *et al*, 2018) and expands our understanding of the pathophysiology of dual-targeted proteins.

Deep sequencing of mtDNA from patient muscle revealed abundant major arc mtDNA rearrangements with prominent breakpoints at the end of the NCR (Fig 2). This pattern of breakpoints is comparable to that observed with other autosomal disorders of mtDNA maintenance (Zeviani *et al*, 1989), and consistent with deletion formation via copy choice recombination (Persson *et al*, 2019). According to this model, replication stalling promotes the slippage of the template during mtDNA synthesis, leading to the generation of deletion-containing mtDNA molecules. The loss of mitochondrial TOP3A activity has been found to result in DNA replication stalling throughout the mtDNA, presumably resulting from the reduced efficiency of replicating through topologically constrained mtDNA molecules (Hangas *et al*, 2022; Menger *et al*, 2022) and so would be expected to promote deletion formation according to this model. In addition, skeletal muscle mtDNA from Pa2 showed an unusual pattern of small duplications close to the promoters and replication origin OriH within the NCR (Figs 2 and EV3), similar to a duplication previously seen in patients with mitochondrial myopathies (Torroni *et al*, 1994; Manfredi *et al*, 1995). This region has previously been mapped as the approximate site of TOP3A activity in cultured cells (Nicholls *et al*, 2018), suggesting that the formation of these duplications may be promoted by aberrant activity of this TOP3A variant within the NCR.

The *TOP3A* gene has been shown to be essential for embryonic development in mice (Li & Wang, 1998), suggesting that all combinations of pathological TOP3A variants found in disease must retain at least some residual activity. Patients with a Bloom syndrome-like disorder were predominantly found to carry truncating variants in TOP3A that resulted in markedly reduced protein levels, with only a single missense variant (p.Ala176Val) being identified (Martin *et al*, 2018). The p.Ala176Val variant was found *in trans* with a loss of function allele (Martin *et al*, 2018), and showed severely reduced or undetectable activity in our biochemical assays. Notably, the truncating p.Ser810* variant retained activity in our decatenation assays, consistent with previous observations that the C-terminal domain of TOP3A is not required for Holliday junction dissolution (Bocquet *et al*, 2014), and also with the suggestion that these disease phenotypes are the result of low protein levels *in vivo* rather than because of impaired protein activity (Martin *et al*, 2018). Both of these Bloom syndrome-associated variants also showed only a limited capacity to ameliorate the mtDNA copy number loss and structural phenotypes associated with TOP3A depletion in rescue experiments. In contrast, all variants identified in mitochondrial disease patients thus far have been either missense variants or loss-of-function alleles *in trans* with missense variants. *In vitro* decatenation assays using recombinant proteins found that different variants were associated with varying levels of catalytic impairment, with mutation of the most conserved residues (Leu37 and Met575, as well as Ala176) being associated with the greatest loss of enzymatic activity. We determined that different variants affect different aspects of the *in vitro* enzymatic activity, with p.Leu37Val and p.Met575Val impairing the DNA-binding activity of the enzyme, a prerequisite for its catalytic activity. In contrast, the p.Ala176Val variant associated with Bloom syndrome exhibited normal DNA-binding activity but was greatly impaired in its decatenation activity. Experiments using combinations of variants observed in patients with segregating heterozygous variants with mitochondrial disease highlight that each individual possesses at least one *TOP3A* allele retaining significant ssDNA decatenation activity, underscoring the importance of considering the combination of variants together. Interestingly some variants, particularly Leu37Val, were capable of significantly rescuing mtDNA copy number loss induced by siRNA depletion, despite possessing little activity *in vitro*. These differences may be accounted for by greater stability of the protein *in vivo*, or by differences in expression level between the endogenous and inducibly expressed proteins.

All pathological variants showed significantly impaired relaxation activity *in vitro* compared to the wild-type protein. However, we note that the type IB topoisomerase TOP1MT, which localises to human mitochondria (Zhang *et al*, 2001), is also capable of relieving supercoiling and could compensate for the lack of TOP3A activity *in vivo*. In contrast, TOP3A is the only mitochondrial topoisomerase that possesses ssDNA decatenation activity (Menger *et al*, 2022), highlighting the importance of maintaining this activity for mtDNA replication.

The variable clinical presentations and age of onset of individuals with similar genotypes potentially may also suggest the existence of additional, currently unidentified modifying variants.

Taken together, our results support a model in which *TOP3A* variants that cause a moderate loss of enzymatic activity result in adult-onset mitochondrial disease, whereas variants that cause a severe loss of enzymatic activity result in a Bloom syndrome-like disorder with mitochondrial phenotypes in childhood. The

 © 2023 The Authors

identification of so many new families strongly argues for the inclusion of *TOP3A* as a mitochondrial disease gene associated with adult mitochondrial disease characterised by a PEO phenotype and Mendelian inheritance (Llaurado *et al*, 2022; Primiano *et al*, 2022).

Materials and Methods

Molecular genetic diagnoses of subjects with bi-allelic TOP3A variants

Molecular genetic studies were largely focussed on whole-genome sequencing (WGS) of probands or family trios/quartets, with the exception of Family I and Family VIII where the probands were subjected to whole-exome sequencing (WES), identifying segregating *TOP3A* variants causing focal mitochondrial pathology in muscle and mtDNA instability (Nicholls *et al*, 2018).

For Family II, commercial WGS (Illumina HiSeqX) was undertaken on the family quartet (female proband, clinically unaffected father, mother and brother).

For Families III, V, VI and VII, WGS data were initially generated through the 100,000 Genomes Project (Turnbull *et al*, 2018; Investigators *et al*, 2021) and re-analysed using a panel of genes related to possible mitochondrial disorders, which included the *TOP3A* gene. For Family IV, exome sequencing was performed by Regeneron (NY, USA) using an IDT xGen capture kit (Integrated DNA Technologies, Iowa, USA) on a NovaSeq 6000 (Illumina, San Diego, CA, USA). Reads were aligned to the UCSC human reference assembly (hg19) with the BWA-MEM algorithm (BWA v.0.7.15). Variants were called using GATK variant caller version 3.8 as recommended by the Broad Institute (DePristo *et al*, 2011). Variant annotation was performed using KGGSEQ (Li *et al*, 2012). Further annotation and filtration steps were performed using various public databases, HGMD professional (The Human Gene Mutation Database) and internal databases by application of in-house custom scripts. For Family IX, commercial sequencing data were analysed using a panel of 333 genes related to mitochondrial function. Informed consent for diagnostic and research studies was obtained for all subjects in accordance with the Declaration of Helsinki protocols and approved by local institutional review boards. Individuals voluntarily offered their informed consent for publication of their clinical details and, as appropriate, the use of their donated tissues and cells for these studies.

Muscle histopathology and molecular genetics

Standard histological and histochemical analyses were performed on fresh-frozen sections (10 μm) of skeletal muscle biopsy according to established protocols (Old & Johnson, 1989). This included the assay of cytochrome c oxidase (COX) both individually and using a sequential COX/succinate dehydrogenase (SDH) assay. Additionally, mitochondrial OXPHOS function was assessed using a quadruple immunohistochemical assay of complex I (NDUFB8), complex IV (COXI) and porin (mitochondrial mass marker) immunoreactivity as previously reported (Rocha *et al*, 2015). Large-scale mtDNA rearrangements were investigated in patient muscle biopsies using validated long-range PCR protocols (Blakely *et al*, 2004).

Multiple sequence alignment

TOP3A protein sequences from *Homo sapiens* (NCBI Reference Sequence: NP_004609.1), *Pan troglodytes* (JAA39790.1), *Bos taurus* (NP_001071443.1), *Gallus gallus* (NP_001025807.1), *Danio rerio* (XP_6 88695.4), *Xenopus laevis* (NP_001085699.1), *Drosophila melanogaster* (NP_523602.2) and *Caenorhabditis elegans* (AAC13567.1) were aligned using Clustal Omega. Homology is indicated below the alignments using standard notation ("*"=full conservation, ":"=strongly similar properties, "."=weakly similar properties).

Structural analysis

The crystal structure of the TOP3A-RMI1 complex (PDB: 4CGY) was used as a reference model to map the locations of the identified patient mutations with UCSF Chimera (Pettersen *et al*, 2004). The affected residues were further analysed with the "Find Clashes/Contacts" and "FindHBond" tools in UCSF Chimera (Pettersen *et al*, 2004) to identify contacts (polar and non-polar interactions) and hydrogen bonds of the wild-type residues. Default settings were used for both tools.

Analysis of TOP3A variants using long-read sequencing

To determine the phase of variants c.298A>G and c.1723A>G in Pa5-1 and c.778C>T and c.1723A>G in Pa6, whole-genome libraries were prepared and run on a MinION long-read sequencer (Oxford Nanopore Technologies (ONT), Oxford, UK). Bulk genomic DNA was first processed, without shearing, in a combined end-repair and nickase treatment reaction. This comprised 3.5 μl of formalin-fixed paraffin-embedded (FFPE) DNA repair buffer (New England Biolabs [NEB]), 2 μl of FFPE DNA repair mix, 3.5 μl of Ultra™ II end prep reaction buffer (NEB), 3 μl of Ultra™ II end prep enzyme mix (NEB) and 1 μg of bulk genomic DNA made up to a total reaction volume of 60 μl. The reaction was incubated at 20°C for 5 min and then 65°C for 5 min before being purified using AMPure XP beads. Sequencing adapters from the LSK110 kit (ONT) were ligated to the prepared DNA in a reaction that comprised 5 μl of Adapter Mix F (AMX F; ONT), 25 μl of Ligation Buffer (LNB; ONT), 10 μl of Quick T4 Ligase (NEB) and 60 μl of eluted DNA. The reaction was incubated at room temperature for 10 min before a further AMPure clean-up was performed, and then the beads were washed twice, each time using 250 μl of Long Fragment Buffer (LFB; ONT). The sample was eluted in 15 μl of Elution Buffer (ONT). To prime an R9.4.1 flow cell, 800 μl of flow cell priming mix (30 μl of Flush Tether [FLT; ONT] added to a tube of Flush Buffer [FB]) was loaded through the priming port; after 5 min, the SpotON cover was opened and a further 200 μl of priming mix was added. The prepared sample (37.5 μl of Sequencing Buffer II (SBII; ONT), 25.5 μl of Load Beads (LBII; ONT) and 15 μl of DNA library) was loaded through the SpotON port and a 72-h sequencing run was initiated using MinKNOW software v.22.05.05 (ONT).

Offline base calling, to convert raw data from fast5 to FASTQ format, was performed with Guppy v.6.1.5 using the super-high-accuracy model (dna_r9.4.1_450bps_sup.cfg). Sequence reads were next aligned to an indexed human reference genome (build GRCh37) using minimap2 v.2.16 (https://lh3.github.io/minimap2/). Samtools v.1.9 (http://www.htslib.org/) was used to aid file

© 2023 The Authors

manipulations (SAM to BAM conversion, sorting reads by alignment coordinate and file indexing), and NanoStat v.1.1.2 (https://github.com/wdecoster/nanostat) was used to generate assay performance metrics. The Integrative Genome Viewer v.2.15.2 was used to visualise sequence reads.

Analysis of mtDNA deletions and duplications using next-generation sequencing

Total DNA was isolated from muscle using standard protocols. DNA was subjected to whole-genome sequencing on a NextSeq 500 (Illumina) platform. Analyses were performed following the previously described MitoSAlt pipeline (Basu *et al*, 2020). Reads were first mapped to the human genome (hg19 assembly for the nuclear genome and NCBI reference sequence: NC_012920.1 for the mitochondrial genome) using Bowtie2 (Langmead & Salzberg, 2012; parameters: very fast), then mitochondrial mapped reads and unmapped reads were realigned to the mitochondrial genome using LAST (Kielbasa *et al*, 2011; lastdb parameters: uNEAR; lastal parameters: Q1–e80). During post-alignment, deletions and duplications were identified, and gapped alignments, indicative of deletions and duplications, were clustered and visualised. Heteroplasmy levels were calculated by comparing the number of reads supporting the corresponding breakpoints to the total number of reads overlapping the breakpoints (including wild-type) after removal of PCR duplicates. For all analyses, cluster_threshold was set as 1, Breakthreshold as −2 and Heteroplasmy limit as 0.01.

Cloning

For protein purification, human TOP3A (amino acids 16–934, sequence codon optimised for expression in *Spodoptera frugiperda* cells and with a TEV-cleavable 6 × His-tag at the N-terminus) was cloned into the pBacPAK9 vector (Clontech). Constructs containing pathological variants of *TOP3A* were created using a QuickChange Lightning site-directed mutagenesis kit (Agilent Technologies) according to the manufacturer's instructions, and the creation of variants was confirmed using Sanger sequencing (Eurofins MWG Operon). For human cell transfection, constructs containing either wild-type or variant-containing TOP3A (amino acids 1–934 and with a C-terminal HA tag, except the Ser810* variant which was expressed as amino acids 1–810 without an epitope tag) were cloned into pcDNA5 FRT/TO.

Cell maintenance and transfection

Untransfected human U2OS cells carrying a stably integrated Flp recombination target (FRT) locus were cultured in Dulbecco's Modified Eagle Medium (DMEM, high glucose with GlutaMAX supplement, Gibco), supplemented with 10% foetal bovine serum (FBS), 100 U/ml penicillin, 100 μg/ml streptomycin, 15 μg/ml blasticidin and 100 μg/ml Zeocin. Following transfection, cells were maintained in DMEM supplemented with 10% FBS, 100 U/ml penicillin, 100 μg/ml streptomycin, 15 μg/ml blasticidin and 50 μg/ml hygromycin. HeLa cells were maintained in DMEM supplemented with 10% FBS. Both cell lines were gifts from Prof. R. Lightowlers, Newcastle University, and were routinely tested for mycoplasma contamination.

For stable transfection, approx. 250,000 cells were transfected with 2,250 ng of pOG44 and 250 ng of pcDNA5 FRT/TO containing the *TOP3A* cDNA (WT or pathological variant containing) using Lipofectamine 3000 (Invitrogen) in antibiotic-free medium according to the manufacturer's instructions. Cells were passaged to 9 cm cell culture dishes after 24 h, and selective medium were added after 48 h. After approx. 2 weeks of selection, colonies were transferred to 24-well plates using paper cloning disks in order to isolate clonal cell lines.

For siRNA transfection and rescue experiments, approx. 100,000 cells were transfected with 5 nM of Silencer Select TOP3A siRNA (Ambion, see Appendix Table S1) using Lipofectamine RNAiMAX in serum-free OptiMEM media (Thermo Fisher), and added to cells in antibiotic-free DMEM containing 5 ng/ml doxycycline to induce expression of TOP3A variants. Cells were re-transfected after 3 days using the same procedure and collected after a total of 6 days of transfection. For transient transfection of HeLa cells, cells were co-transfected with 2,500 ng of pcDNA5-containing TOP3A Ser810* during re-transfection with siRNA.

Southern blotting

Total DNA from muscle (300 ng) as above was restricted using 10 U of BamHI-HF or PvuII-HF (NEB) as indicated for 1 h at 37°C. For experiments including T7 endonuclease I, 250 ng of muscle DNA was restricted using 10 U of BamHI for 30 min at 37°C, then 1 U of T7 endonuclease I (NEB) was added and the reactions were incubated for a further 30 min at 37°C. Reactions were separated on 0.6% agarose gels for 4 h at 110 V. Gels were incubated in depurination buffer (0.25 M HCl) for 20 min, followed by denaturation buffer (0.5 M NaOH, 1.5 M NaCl) for 2 × 10 min and then neutralisation buffer (0.5 M tris–HCl (pH 7.4), 1.5 M NaCl) for a further 2 × 10 min. Gels were blotted onto nylon membrane (Hybond-N+, Amersham) overnight and then crosslinked using UV at 1200 mJ/cm^2. A probe (corresponding to mtDNA loci nt. 16262-128) was synthesised by radiolabelling a PCR product using a Prime-It II random primer labelling kit (Agilent); see Appendix Table S1 for primer sequences. Membranes were hybridised overnight at 60°C in hybridisation buffer (0.25 M phosphate buffer, 7% (w/v) SDS), then washed for 3 × 20 min with 1 × SSC containing 0.1% (w/v) SDS and imaged using a Typhoon FLA 9500 or exposed to X-ray film.

Western blotting

Cells were lysed in 50 mM Tris–HCl (pH 7.4), 150 mM NaCl, 1 mM EDTA, 1% (v/v) Triton X-100 and 1 × proteinase inhibitors for 30 min at 4°C, and equal protein amounts were separated on 4–20% Criterion TGX SDS–PAGE gels (Bio-Rad). Primary antibodies were incubated overnight at 4°C, and secondary antibodies were incubated for 1 h at room temperature. Membranes were developed using Pierce ECL or SuperSignal West Pico Plus (Thermo Fisher). Antibodies used were as follows: rabbit anti-HA (Sigma Aldrich H6908; RRID:AB_260070, 1:1,000), mouse anti-β-actin (Abcam ab6276; RRID:AB_2223210, 1:50,000), rabbit anti-TOP3A (Proteintech 14525-1-AP; RRID:AB_2205881, 1:1,000), rabbit anti-mouse HRP (Agilent P0260; RRID:AB_2636929, 1:3,000) and swine anti-rabbit HRP (Agilent P0217; RRID:AB_2728719, 1:3,000).

 © 2023 The Authors

Analysis of mtDNA topology

Cells were washed once with PBS and then resuspended in lysis buffer (75 mM NaCl, 50 mM EDTA, 20 mM HEPES (pH 7.8), 0.5% (w/v) SDS and 0.2 mg/ml proteinase K). DNA was isolated by sequential phenol and chloroform extractions and resuspended in TE (pH 8). DNA (2.5 μg) was separated on 0.4% agarose gels, without ethidium bromide, at 35 V for 22 h. As controls, 1 μg of DNA from wild-type U2OS Flp-In cells was treated with 10 U of BamHI-HF (NEB) or 5 U of *E. coli* Topoisomerase I (NEB) for 30 min at 37°C in 1 × CutSmart buffer (NEB), before being loaded on gels alongside uncut DNA as above. After electrophoresis, gels were prepared for Southern blotting as above for restricted samples.

Quantitative PCR

Total cellular DNA was isolated using a DNeasy Blood and Tissue kit (Qiagen) according to the manufacturer's instructions. The mtDNA target *MT-ND1* and nuclear target *B2M* were amplified as technical triplicates in 2 × Taqman universal PCR master mix (Applied Biosystems) on a StepOnePlus Real-Time PCR system using 50 ng of template DNA, 300 nM of each primer and 100 nM of probe. Oligo sequences are provided in Appendix Table S1. Cycling conditions were as follows: 50°C for 2 min, 95°C for 10 min, then 40 cycles of 95°C for 15 s and 60°C for 1 min. A standard curve was generated by amplifying both targets from a single plasmid, and used to determine *B2M* and *MT-ND1* levels. The mtDNA copy number was calculated as *MT-ND1/B2M* and normalised to the level of untransfected samples. Error bars are expressed as the standard error of the mean and converted to percentages according to the normalised *MT-ND1/B2M* ratio.

Protein purification

Human TOP3A was expressed in *Spodoptera frugiperda* Sf9 cells. Following expression, cells were harvested and lysis was performed in lysis buffer (20 mM Tris–HCl (pH 8), 500 mM NaCl, 10 mM β-mercaptoethanol and 1 × proteinase inhibitors). Samples were freeze-thawed once using liquid nitrogen to aid lysis. The lysate was cleared by centrifugation at 75,000 *g* for 45 min at 4°C using a Sorvall Surespin 630 rotor in a Thermo Scientific Sorvall WX 100 ultracentrifuge. The first purification step was performed using His-Select Nickel Affinity Gel (Sigma-Aldrich) which was equilibrated with Nickel Buffer (25 mM HEPES (pH 7), 400 mM NaCl, 10% (v/v) glycerol and 10 mM β-mercaptoethanol) containing 5 mM imidazole. The protein was washed with Nickel Buffer containing 10 mM imidazole, and eluted with Nickel Buffer containing 250 mM imidazole. The 6× His-tag-fusion protein was cleaved by dialysis in 1 l of Nickel Buffer with TEV protease at 4°C overnight. The protein was then reloaded onto His-Select Nickel Affinity Gel, and the flow through was collected. The flow through was further purified using HiTrap Heparin HP (GE Healthcare Life Sciences). The heparin column was equilibrated with Buffer A (25 mM HEPES (pH 7), 200 mM NaCl, 10% (v/v) glycerol, 1 mM DTT, 0.5 mM EDTA (pH 8) and 1 × proteinase inhibitors) and the protein was eluted as a linear gradient with Buffer B (25 mM HEPES (pH 7), 1.2 M NaCl, 10% (v/v) glycerol, 1 mM DTT, 0.5 mM EDTA (pH 8) and 1 × proteinase inhibitors). The TOP3A peak fractions were collected and further purified using a Superdex 200 16/600 gel filtration column (GE Healthcare Life Sciences) equilibrated with Buffer C (25 mM HEPES (pH 7), 400 mM NaCl, 10% (v/v) glycerol, 1 mM DTT, 0.5 mM EDTA (pH 8) and 1 × proteinase inhibitors). Finally, the purified human TOP3A protein samples were concentrated using HiTrap SP HP (GE Healthcare Life Sciences) equilibrated with Buffer A and eluted with Buffer B with a linear gradient.

Lk1 ssDNA catenane synthesis, extraction and validation

Lk1 ssDNA catenanes were synthesised according to the method of Li *et al* (2018). R1, R2, H1 and H2 oligos (10 pmol each) were mixed in 1 × T4 DNA Ligase (NEB) buffer (50 mM Tris–HCl, 10 mM $MgCl_2$, 1 mM ATP and 10 mM DTT, pH 7.5) in a total volume of 17 μl. The mixture was heated to 90°C for 3 min and cooled at 0.1°C/s to 25°C. S1 and S2 oligos (20 pmol each) were then added and the mixture was incubated at 25°C for 20 min. T4 DNA ligase (400 U) was then added and the mixture was incubated at 25°C for a further 2 h. Proteinase K was added to a concentration of 1 mg/ml and incubated at room temperature for 15 min. Reaction products were separated by denaturing PAGE (12% (v/v) acrylamide/bis (19:1), 7 M urea and 1 × TBE), and the gel was stained with 1 × SYBR gold. Bands corresponding to the Lk1 ssDNA catenanes were cut from the gel and soaked in 1 × TE at room temperature overnight. The tube was then briefly centrifuged and the supernatant was transferred to a new tube and stored at 4°C. The concentration of Lk1 ssDNA catenanes was calculated by separating products by PAGE alongside a standard curve and quantifying band intensities using ImageLab software (Bio-Rad Laboratories).

To validate the identity of the Lk1 ssDNA catenanes, 400 fmol of DNA catenanes were incubated with either 20 U of S1 nuclease (Promega), 100 U of exonuclease III (NEB), or 5 U of CviKI-1 (NEB) at 37°C for 30 min (S1 nuclease and exonuclease III) or 4 h (CviKI-1). Following these reactions, 1 μl of 20 mg/ml proteinase K was added and the reactions were incubated at room temperature for 20 min. Samples were separated by 12% PAGE and imaged as described above.

Decatenation reactions

The concentrations of purified TOP3A variants were measured using a Qubit protein assay (Thermo Fisher Scientific). Reactions were carried out in a total volume of 20 μl in reaction buffer (25 mM Tris–HCl pH 7.4, 5 mM $MgCl_2$, 50 mM NaCl, 1 mM DTT and 100 μg/ml BSA) at 37°C for 30 min. Proteins were diluted using dilution buffer (20 mM tris-Cl pH 8, 200 mM NaCl, 1 mM DTT, 10% (v/v) glycerol, 0.5 mM EDTA and 100 μg/ml BSA). In experiments involving two compound heterozygous variants, reactions contained 50 fmol of protein (for single variants) or 25 fmol for each of the two variants. The reaction products were then separated by denaturing PAGE as described above. Reaction products were electroblotted onto Hybond-N+ membranes (Cytiva) at 50 V in 1 × TBE for 1 h. Membranes were crosslinked by exposure to 1200 mJ/cm^2 of 254 nm UV. Membranes were hybridised overnight at 42°C with 5′ radioactively labelled DNA probes complementary to R1 or R2 (see Appendix Table S1 for probe sequences). For probe synthesis, 10 pmol of each of R1 probe and R2 probe were labelled using T4 polynucleotide kinase (NEB) and 2 μl of [γ-^{32}P] ATP (10 mCi/ml,

3,000 Ci/mmol, Hartmann Analytic). The following day, membranes were washed for 3 × 20 min with saline-sodium citrate (SSC) buffer containing 0.1% SDS and imaged using a Typhoon FLA 9500.

Electrophoretic mobility shift assay (EMSA)

A 40-mer or 80-mer ssDNA oligonucleotide (see Appendix Table S1 for oligo sequences) was labelled at the 5′ end with [γ-^{32}P] ATP using T4 PNK. EMSA reactions (20 µl) contained 10 fmol labelled ssDNA oligonucleotide, 25 mM HEPES (pH 7.5), 100 µg/ml BSA, 1 mM DTT, 10% (v/v) glycerol, 70 mM NaCl and increasing amounts (0, 25, 50, 75, 100 or 250 fmol) of recombinant TOP3A. Reactions were incubated at 37°C for 15 min, then transferred to ice. The samples were loaded onto 8% native polyacrylamide gels and separated at 150 V in 0.5 × TBE running buffer at 4°C for 50 min and visualised by autoradiography. Each experiment was performed at least three times.

DNA relaxation assays

Reaction mixtures (20 µl) contained supercoiled pUC19 (250 ng), 40 mM HEPES (pH 7.6), 1 mM $MgCl_2$ and 30% (v/v) glycerol. The proteins were diluted with dilution buffer (25 mM HEPES (pH 7.6), 100 mM NaCl, 10% (v/v) glycerol, 1 mM DTT and 100 µg/ml BSA). The supercoiled plasmid template was left untreated or treated with 100 fmol, 300 fmol, 1 pmol or 3 pmol TOP3A at 37°C for 45 min. The reactions were stopped by the addition of 1 µl of 20 mg/ml proteinase K, 2 µl of 5% (w/v) SDS and 2 µl of 50 mM EDTA (pH 8), and incubated at 37°C for 30 min. Lastly, 6 µl of 6 × DNA loading dye (Thermo Scientific) was added, and the samples were loaded onto a 0.8% agarose gel in TAE buffer without ethidium bromide, and run at 50 V for 3.5 h. The gels were then stained with 500 ng/ml ethidium bromide in water for 45 min and visualised under UV light. Each experiment was performed at least three times.

Quantification

For the quantification of EMSAs and relaxation assays, images were imported into ImageLab software (version 6.0.1 build 34, standard edition, Bio-Rad Laboratories). Lanes and bands were manually selected and the background was adjusted equally across all lanes of a single gel. For EMSA quantification, the intensity of the unbound DNA band was determined and expressed as a percentage of overall lane intensity. The inverse of unbound band intensity was taken as signal intensity for bound DNA. For relaxation assays, the relative band intensity of the supercoiled plasmid was determined and used to calculate the percentage of substrate released from supercoiled form. For decatenation assays, the proportion of substrate and decatenated product DNA was quantified using ImageJ. One-way ANOVA was performed independently for each topoisomerase concentration to determine statistically significant differences between treatments.

Statistical analysis

One-way ANOVA was used to identify differences between samples using GraphPad Prism 9. All statistical calculations used three independent experiments unless indicated otherwise in figure legends. For qPCR experiments, the value of each biological replicate represents the mean of three technical triplicate reactions. For experiments using patient-derived material, the samples were anonymised but not blinded. Data are presented as mean values ±SEM. Significance values used throughout the paper are as follows: *$P < 0.05$, **$P < 0.01$, ***$P < 0.001$ and ****$P < 0.0001$.

The paper explained

Problem
Mitochondrial diseases are a heterogeneous group of disorders that are among the most common form of inherited human metabolic disease. The range of symptoms, varying age of onset and different possible inheritance patterns of mitochondrial diseases present clinical and diagnostic challenges. Mitochondrial disease can be caused by variants in the nuclear genome or the mitochondrial genome, mtDNA, which is autonomously replicated by a nuclear-encoded protein machinery. Several proteins involved in mtDNA maintenance and repair localise to both the nucleus and mitochondria. One such protein is TOP3A, a topoisomerase that is involved in DNA replication termination and decatenation in mitochondria, and DNA recombination in the nucleus. Pathological variants in *TOP3A* have been found to cause symptoms associated with the nuclear function of TOP3A (Bloom syndrome) as well as mitochondrial dysfunction, but the pathophysiology associated with these different variants has not been established.

Results
We report 11 patients from nine families with an adult-onset mitochondrial disease associated with bi-allelic pathological variants in *TOP3A*. Common symptoms include ptosis, ophthalmoplegia, cardiac conduction defects, myopathy, sensorineural hearing loss, cerebellar ataxia and sensory axonal motor neuropathy. Pathological variants are associated with instability of the mitochondrial genome and affect different aspects of the enzyme function, including ssDNA binding, the relaxation of supercoiled DNA molecules and the ability to separate (decatenate) ssDNA substrates. Compound heterozygous variants that overall result in a milder impairment of TOP3A activity were associated with mitochondrial disease, whereas variants associated with Bloom syndrome with mitochondrial dysfunction caused a greater loss of enzymatic activity. Based on our data we propose that the degree of catalytic impairment determines whether *TOP3A* variants manifest as mitochondrial disease or in Bloom syndrome.

Impact
Our results expand the links between mitochondrial DNA maintenance and mitochondrial disease and provide insights into different diseases caused by different functions of a single mitochondrial and nuclear enzyme. These data may facilitate the identification of further cases of TOP3A-related mitochondrial disease and aid future investigations into the pathophysiology of dual-localised proteins.

Data availability

Due to consent agreement restrictions, patient WGS data could not be made freely available and are therefore not deposited in a public database.

Expanded View for this article is available online.

Acknowledgements
This research was supported by the Wellcome Centre for Mitochondrial Research (203105/Z/16/Z), the Mitochondrial Disease Patient Cohort (UK)

© 2023 The Authors

(G0800674), the UK National Institute for Health Research (NIHR) Biomedical Research Centre for Ageing and Age-Related Disease award to the Newcastle upon Tyne Hospitals National Health Service (NHS) Foundation Trust and the UK NHS Specialist Commissioners, which funds the Rare Mitochondrial Disorders of Adults and Children Diagnostic Service in Newcastle upon Tyne. TJN is the recipient of a Sir Henry Dale Fellowship jointly funded by the Wellcome Trust and the Royal Society (213464/Z/18/Z) and a Rosetrees and Stoneygate Trust Research Fellowship (M811). MF is funded by the Swedish Research Council, Swedish Cancer Foundation, the IngaBritt and Arne Lundberg Foundation, the Knut and Alice Wallenberg Foundation and the Västra Götalands Regionen (ALFGBG-965954). CMG is funded by the Västra Götalands Regionen (ALFGBG-966275). RM and RWT are funded by the Medical Research Council International Centre for Genomic Medicine in Neuromuscular Disease (MR/S005021/1) and the Lily Foundation. RWT receives additional support from the Medical Research Council (MR/W019027/1) and the Pathological Society. JP is funded by the Lily Foundation and the Angus Memorial Mitochondrial Fund. We gratefully acknowledge access to the data and findings generated by the 100,000 Genomes Project, managed by Genomics England Limited (a wholly owned company of the Department of Health and Social Care). The 100,000 Genomes Project is funded by the National Institute for Health Research and NHS England. The Wellcome Trust, Cancer Research UK and the Medical Research Council have also funded research infrastructure. The 100,000 Genomes Project uses data provided by patients and collected by the National Health Service as part of their care and support. The views expressed are those of the author(s) and not necessarily those of the NIHR, the NHS or the UK Department of Health and Social Care. The authors wish to thank Dr Yael Finezilber for her clinical assistance.

Author contributions

Direnis Erdinc: Conceptualization; formal analysis; investigation; visualization. **Alejandro Rodríguez-Luis:** Conceptualization; formal analysis; investigation; visualization. **Mahmoud Fassad:** Formal analysis; investigation; writing – original draft; writing – review and editing. **Sarah Mackenzie:** Investigation. **Christopher M Watson:** Formal analysis; investigation; visualization; methodology; writing – original draft; writing – review and editing. **Sebastian Valenzuela:** Software; formal analysis; investigation; visualization; methodology; writing – original draft; writing – review and editing. **Xie Xie:** Software; formal analysis; investigation; visualization; methodology. **Katja E Menger:** Formal analysis; investigation. **Kate Sergeant:** Investigation. **Kate Craig:** Investigation. **Sila Hopton:** Investigation. **Gavin Falkous:** Resources; funding acquisition; investigation. **Joanna Poulton:** Resources; funding acquisition; investigation. **Hector Garcia-Moreno:** Resources; investigation. **Paola Giunti:** Resources; investigation. **Carlos de Moura Aschoff:** Resources; investigation. **Jonas Saute:** Resources; investigation. **Amelia Kirby:** Resources; investigation. **Camilo Toro:** Resources; investigation. **Lynne Wolfe:** Resources; investigation. **Danica Novacic:** Resources; investigation. **Lior Greenbaum:** Resources; investigation. **Aviva Eliyahu:** Resources; formal analysis; investigation. **Ortal Barel:** Resources; formal analysis; investigation. **Yair Anikster:** Resources; funding acquisition; investigation. **Robert McFarland:** Resources; funding acquisition; investigation. **Grainne Gorman:** Resources; formal analysis; funding acquisition; investigation; writing – original draft. **Andrew Schaefer:** Resources; formal analysis; supervision; funding acquisition; investigation; writing – original draft. **Claes M Gustafsson:** Conceptualization; supervision; funding acquisition; writing – original draft; writing – review and editing. **Robert Taylor:** Conceptualization; supervision; funding acquisition; writing – original draft; writing – review and editing. **Maria Falkenberg:** Conceptualization; supervision; funding acquisition; investigation; writing – original draft; writing – review and editing. **Thomas J Nicholls:** Conceptualization; formal analysis; supervision; funding acquisition; investigation; visualization; methodology; writing – original draft; writing – review and editing.

Disclosure and competing interests statement

The authors declare that they have no competing interests.

For more information

- The Lily Foundation (https://www.thelilyfoundation.org.uk/)
- United Mitochondrial Disease Foundation (https://www.umdf.org/)
- Mito Foundation (https://www.mito.org.au/)
- Genomics England (https://www.genomicsengland.co.uk/)
- TOP3A in Online Mendelian Inheritance in Man (https://www.omim.org/entry/601243)

References

Aravind L, Leipe DD, Koonin EV (1998) Toprim – a conserved catalytic domain in type IA and II topoisomerases, DnaG-type primases, OLD family nucleases and RecR proteins. *Nucleic Acids Res* 26: 4205–4213

Basu S, Xie X, Uhler JP, Hedberg-Oldfors C, Milenkovic D, Baris OR, Kimoloi S, Matic S, Stewart JB, Larsson NG *et al* (2020) Accurate mapping of mitochondrial DNA deletions and duplications using deep sequencing. *PLoS Genet* 16: e1009242

Bizard AH, Hickson ID (2020) The many lives of type IA topoisomerases. *J Biol Chem* 295: 7138–7153

Bizard AH, Allemand JF, Hassenkam T, Paramasivam M, Sarlos K, Singh MI, Hickson ID (2019) PICH and TOP3A cooperate to induce positive DNA supercoiling. *Nat Struct Mol Biol* 26: 267–274

Blakely EL, He L, Taylor RW, Chinnery PF, Lightowlers RN, Schaefer AM, Turnbull DM (2004) Mitochondrial DNA deletion in "identical" twin brothers. *J Med Genet* 41: e19

Bocquet N, Bizard AH, Abdulrahman W, Larsen NB, Faty M, Cavadini S, Bunker RD, Kowalczykowski SC, Cejka P, Hickson ID *et al* (2014) Structural and mechanistic insight into Holliday-junction dissolution by topoisomerase IIIalpha and RMI1. *Nat Struct Mol Biol* 21: 261–268

Brockington M, Sweeney MG, Hammans SR, Morgan-Hughes JA, Harding AE (1993) A tandem duplication in the D-loop of human mitochondrial DNA is associated with deletions in mitochondrial myopathies. *Nat Genet* 4: 67–71

Cao N, Tan K, Zuo X, Annamalai T, Tse-Dinh YC (2020) Mechanistic insights from structure of mycobacterium smegmatis topoisomerase I with ssDNA bound to both N- and C-terminal domains. *Nucleic Acids Res* 48: 4448–4462

Cejka P, Plank JL, Dombrowski CC, Kowalczykowski SC (2012) Decatenation of DNA by the *S. cerevisiae* Sgs1-Top3-Rmi1 and RPA complex: a mechanism for disentangling chromosomes. *Mol Cell* 47: 886–896

Chacinska A, Koehler CM, Milenkovic D, Lithgow T, Pfanner N (2009) Importing mitochondrial proteins: machineries and mechanisms. *Cell* 138: 628–644

Changela A, DiGate RJ, Mondragon A (2007) Structural studies of *E. coli* topoisomerase III-DNA complexes reveal a novel type IA topoisomerase-DNA conformational intermediate. *J Mol Biol* 368: 105–118

Chen CF, Brill SJ (2007) Binding and activation of DNA topoisomerase III by the Rmi1 subunit. *J Biol Chem* 282: 28971–28979

DePristo MA, Banks E, Poplin R, Garimella KV, Maguire JR, Hartl C, Philippakis AA, del Angel G, Rivas MA, Hanna M *et al* (2011) A framework for variation

discovery and genotyping using next-generation DNA sequencing data. *Nat Genet* 43: 491–498

Ellis NA, Groden J, Ye TZ, Straughen J, Lennon DJ, Ciocci S, Proytcheva M, German J (1995) The Bloom's syndrome gene product is homologous to RecQ helicases. *Cell* 83: 655–666

Filograna R, Mennuni M, Alsina D, Larsson NG (2021) Mitochondrial DNA copy number in human disease: the more the better? *FEBS Lett* 595: 976–1002

Gorman GS, Chinnery PF, DiMauro S, Hirano M, Koga Y, McFarland R, Suomalainen A, Thorburn DR, Zeviani M, Turnbull DM (2016) Mitochondrial diseases. *Nat Rev Dis Primers* 2: 16080

Gustafsson CM, Falkenberg M, Larsson NG (2016) Maintenance and expression of mammalian mitochondrial DNA. *Annu Rev Biochem* 85: 133–160

Hagstrom E, Freyer C, Battersby BJ, Stewart JB, Larsson NG (2014) No recombination of mtDNA after heteroplasmy for 50 generations in the mouse maternal germline. *Nucleic Acids Res* 42: 1111–1116

Hanai R, Caron PR, Wang JC (1996) Human TOP3: a single-copy gene encoding DNA topoisomerase III. *Proc Natl Acad Sci U S A* 93: 3653–3657

Hangas A, Kekalainen NJ, Potter A, Michell C, Aho KJ, Rutanen C, Spelbrink JN, Pohjoismaki JL, Goffart S (2022) Top3alpha is the replicative topoisomerase in mitochondrial DNA replication. *Nucleic Acids Res* 50: 8733–8748

Investigators GPP, Smedley D, Smith KR, Martin A, Thomas EA, McDonagh EM, Cipriani V, Ellingford JM, Arno G, Tucci A *et al* (2021) 100,000 genomes pilot on rare-disease diagnosis in health care – preliminary report. *N Engl J Med* 385: 1868–1880

Jiang W, Jia N, Guo C, Wen J, Wu L, Ogi T, Zhang H (2021) Predominant cellular mitochondrial dysfunction in the TOP3A gene-caused bloom syndrome-like disorder. *Biochim Biophys Acta Mol Basis Dis* 1867: 166106

Kielbasa SM, Wan R, Sato K, Horton P, Frith MC (2011) Adaptive seeds tame genomic sequence comparison. *Genome Res* 21: 487–493

Kornblum C, Nicholls TJ, Haack TB, Scholer S, Peeva V, Danhauser K, Hallmann K, Zsurka G, Rorbach J, Iuso A *et al* (2013) Loss-of-function mutations in MGME1 impair mtDNA replication and cause multisystemic mitochondrial disease. *Nat Genet* 45: 214–219

Langmead B, Salzberg SL (2012) Fast gapped-read alignment with bowtie 2. *Nat Methods* 9: 357–359

Li W, Wang JC (1998) Mammalian DNA topoisomerase IIIalpha is essential in early embryogenesis. *Proc Natl Acad Sci U S A* 95: 1010–1013

Li MX, Gui HS, Kwan JS, Bao SY, Sham PC (2012) A comprehensive framework for prioritizing variants in exome sequencing studies of Mendelian diseases. *Nucleic Acids Res* 40: e53

Li Q, Li J, Cui Y, Liu S, An R, Liang X, Komiyama M (2018) Two-holder strategy for efficient and selective synthesis of Lk 1 ssDNA catenane. *Molecules* 23: 2270

Llaurado A, Rovira-Moreno E, Codina-Sola M, Martinez-Saez E, Salvado M, Sanchez-Tejerina D, Sotoca J, Lopez-Diego V, Restrepo-Vera JL, Garcia-Arumi E *et al* (2022) Chronic progressive external ophthalmoplegia plus syndrome due to homozygous missense variant in TOP3A gene. *Clin Genet* 103: 492–494

Longley MJ, Clark S, Yu Wai Man C, Hudson G, Durham SE, Taylor RW, Nightingale S, Turnbull DM, Copeland WC, Chinnery PF (2006) Mutant POLG2 disrupts DNA polymerase gamma subunits and causes progressive external ophthalmoplegia. *Am J Hum Genet* 78: 1026–1034

Manfredi G, Servidei S, Bonilla E, Shanske S, Schon EA, DiMauro S, Moraes CT (1995) High levels of mitochondrial DNA with an unstable 260-bp duplication in a patient with a mitochondrial myopathy. *Neurology* 45: 762–768

Martin CA, Sarlos K, Logan CV, Thakur RS, Parry DA, Bizard AH, Leitch A, Cleal L, Ali NS, Al-Owain MA *et al* (2018) Mutations in TOP3A cause a bloom syndrome-like disorder. *Am J Hum Genet* 103: 456

Menger KE, Chapman J, Diaz-Maldonado H, Khazeem MM, Deen D, Erdinc D, Casement JW, Di Leo V, Pyle A, Rodriguez-Luis A *et al* (2022) Two type I topoisomerases maintain DNA topology in human mitochondria. *Nucleic Acids Res* 50: 11154–11174

Nicholls TJ, Nadalutti CA, Motori E, Sommerville EW, Gorman GS, Basu S, Hoberg E, Turnbull DM, Chinnery PF, Larsson NG *et al* (2018) Topoisomerase 3alpha is required for Decatenation and segregation of human mtDNA. *Mol Cell* 69: e26

Olahova M, Peter B, Szilagyi Z, Diaz-Maldonado H, Singh M, Sommerville EW, Blakely EL, Collier JJ, Hoberg E, Stranecky V *et al* (2021) POLRMT mutations impair mitochondrial transcription causing neurological disease. *Nat Commun* 12: 1135

Old SL, Johnson MA (1989) Methods of microphotometric assay of succinate dehydrogenase and cytochrome c oxidase activities for use on human skeletal muscle. *Histochem J* 21: 545–555

Persson O, Muthukumar Y, Basu S, Jenninger L, Uhler JP, Berglund AK, McFarland R, Taylor RW, Gustafsson CM, Larsson E *et al* (2019) Copy-choice recombination during mitochondrial L-strand synthesis causes DNA deletions. *Nat Commun* 10: 759

Pettersen EF, Goddard TD, Huang CC, Couch GS, Greenblatt DM, Meng EC, Ferrin TE (2004) UCSF chimera? A visualization system for exploratory research and analysis. *J Comput Chem* 25: 1605–1612

Plank JL, Chu SH, Pohlhaus JR, Wilson-Sali T, Hsieh TS (2005) *Drosophila melanogaster* topoisomerase IIIalpha preferentially relaxes a positively or negatively supercoiled bubble substrate and is essential during development. *J Biol Chem* 280: 3564–3573

Primiano G, Torraco A, Verrigni D, Sabino A, Bertini E, Carrozzo R, Silvestri G, Servidei S (2022) Novel TOP3A variant associated with mitochondrial disease: expanding the clinical Spectrum of topoisomerase III alpha-related diseases. *Neurol Genet* 8: e200007

Rath S, Sharma R, Gupta R, Ast T, Chan C, Durham TJ, Goodman RP, Grabarek Z, Haas ME, Hung WHW *et al* (2021) MitoCarta3.0: an updated mitochondrial proteome now with sub-organelle localization and pathway annotations. *Nucleic Acids Res* 49: D1541–D1547

Raynard S, Bussen W, Sung P (2006) A double holliday junction dissolvasome comprising BLM, topoisomerase IIIalpha, and BLAP75. *J Biol Chem* 281: 13861–13864

Reyes A, Melchionda L, Nasca A, Carrara F, Lamantea E, Zanolini A, Lamperti C, Fang M, Zhang J, Ronchi D *et al* (2015) RNASEH1 mutations impair mtDNA replication and cause adult-onset mitochondrial encephalomyopathy. *Am J Hum Genet* 97: 186–193

Rocha MC, Grady JP, Grunewald A, Vincent A, Dobson PF, Taylor RW, Turnbull DM, Rygiel KA (2015) A novel immunofluorescent assay to investigate oxidative phosphorylation deficiency in mitochondrial myopathy: understanding mechanisms and improving diagnosis. *Sci Rep* 5: 15037

Ronchi D, Di Fonzo A, Lin W, Bordoni A, Liu C, Fassone E, Pagliarani S, Rizzuti M, Zheng L, Filosto M *et al* (2013) Mutations in DNA2 link progressive myopathy to mitochondrial DNA instability. *Am J Hum Genet* 92: 293–300

Russell OM, Gorman GS, Lightowlers RN, Turnbull DM (2020) Mitochondrial diseases: Hope for the future. *Cell* 181: 168–188

Singh TR, Ali AM, Busygina V, Raynard S, Fan Q, Du CH, Andreassen PR, Sung P, Meetei AR (2008) BLAP18/RMI2, a novel OB-fold-containing protein, is an essential component of the bloom helicase-double Holliday junction dissolvasome. *Genes Dev* 22: 2856–2868

 © 2023 The Authors

Spelbrink JN, Li FY, Tiranti V, Nikali K, Yuan QP, Tariq M, Wanrooij S, Garrido N, Comi G, Morandi L *et al* (2001) Human mitochondrial DNA deletions associated with mutations in the gene encoding Twinkle, a phage T7 gene 4-like protein localized in mitochondria. *Nat Genet* 28: 223 – 231

Sugranes TA, Flanagan M, Thomas C, Chang VY, Walsh M, Cunniff C (2022) Age of first cancer diagnosis and survival in bloom syndrome. *Genet Med* 24: 1476 – 1484

Tan K, Zhou Q, Cheng B, Zhang Z, Joachimiak A, Tse-Dinh YC (2015) Structural basis for suppression of hypernegative DNA supercoiling by *E. coli* topoisomerase I. *Nucleic Acids Res* 43: 11031 – 11046

Torroni A, Lott MT, Cabell MF, Chen YS, Lavergne L, Wallace DC (1994) mtDNA and the origin of Caucasians: identification of ancient Caucasian-specific haplogroups, one of which is prone to a recurrent somatic duplication in the D-loop region. *Am J Hum Genet* 55: 760 – 776

Tsai HZ, Lin RK, Hsieh TS (2016) *Drosophila* mitochondrial topoisomerase III alpha affects the aging process via maintenance of mitochondrial function and genome integrity. *J Biomed Sci* 23: 38

Turnbull C, Scott RH, Thomas E, Jones L, Murugaesu N, Pretty FB, Halai D, Baple E, Craig C, Hamblin A *et al* (2018) The 100 000 genomes project: bringing whole genome sequencing to the NHS. *BMJ* 361: k1687

Van Goethem G, Dermaut B, Lofgren A, Martin JJ, Van Broeckhoven C (2001) Mutation of POLG is associated with progressive external ophthalmoplegia characterized by mtDNA deletions. *Nat Genet* 28: 211 – 212

Vos SM, Tretter EM, Schmidt BH, Berger JM (2011) All tangled up: how cells direct, manage and exploit topoisomerase function. *Nat Rev Mol Cell Biol* 12: 827 – 841

Wang Y, Lyu YL, Wang JC (2002) Dual localization of human DNA topoisomerase IIIalpha to mitochondria and nucleus. *Proc Natl Acad Sci U S A* 99: 12114 – 12119

Wu L, Davies SL, North PS, Goulaouic H, Riou JF, Turley H, Gatter KC, Hickson ID (2000) The Bloom's syndrome gene product interacts with topoisomerase III. *J Biol Chem* 275: 9636 – 9644

Wu L, Bachrati CZ, Ou J, Xu C, Yin J, Chang M, Wang W, Li L, Brown GW, Hickson ID (2006) BLAP75/RMI1 promotes the BLM-dependent dissolution of homologous recombination intermediates. *Proc Natl Acad Sci U S A* 103: 4068 – 4073

Wu J, Feng L, Hsieh TS (2010) *Drosophila* topo IIIalpha is required for the maintenance of mitochondrial genome and male germ-line stem cells. *Proc Natl Acad Sci U S A* 107: 6228 – 6233

Xu D, Guo R, Sobeck A, Bachrati CZ, Yang J, Enomoto T, Brown GW, Hoatlin ME, Hickson ID, Wang W (2008) RMI, a new OB-fold complex essential for bloom syndrome protein to maintain genome stability. *Genes Dev* 22: 2843 – 2855

Yang J, Bachrati CZ, Ou J, Hickson ID, Brown GW (2010) Human topoisomerase IIIalpha is a single-stranded DNA decatenase that is stimulated by BLM and RMI1. *J Biol Chem* 285: 21426 – 21436

Yin J, Sobeck A, Xu C, Meetei AR, Hoatlin M, Li L, Wang W (2005) BLAP75, an essential component of Bloom's syndrome protein complexes that maintain genome integrity. *EMBO J* 24: 1465 – 1476

Zeviani M, Servidei S, Gellera C, Bertini E, DiMauro S, DiDonato S (1989) An autosomal dominant disorder with multiple deletions of mitochondrial DNA starting at the D-loop region. *Nature* 339: 309 – 311

Zhang H, Barcelo JM, Lee B, Kohlhagen G, Zimonjic DB, Popescu NC, Pommier Y (2001) Human mitochondrial topoisomerase I. *Proc Natl Acad Sci U S A* 98: 10608 – 10613

Zhang H, Seol Y, Agama K, Neuman KC, Pommier Y (2017) Distribution bias and biochemical characterization of TOP1MT single nucleotide variants. *Sci Rep* 7: 8614

License: This is an open access article under the terms of the Creative Commons Attribution License, which permits use, distribution and reproduction in any medium, provided the original work is properly cited.

Expanded View Figures

Figure EV1. Establishing the phase of TOP3A variants using long-read sequencing.

A–E Creation of an assembled haplotype at the *TOP3A* locus in Pa5-1. (A) Three reads highlighted in brown, orange and blue span the interval between target variants c.298A>G and c.1723A>G. (B) Read-level data for the haplotype assembled reads (highlighted brown and orange), which are reference nucleotide supporting at position c.1723. (C) Read-level data used to create the assembled haplotype through variant-supporting nucleotides at positions c.1282–21 and c.1468–11. (D) Read-level data for the haplotype assembled read (highlighted blue), which is variant supporting at position c.298. (E) A schematic illustration of the assembled haplotype, for target variants c.298A>G and c.1723A>G, which are consistent with a *trans* configuration (arranged on different parental alleles). Nomenclature provided according to transcript NM_004618.5.

F–J Creation of an assembled haplotype at the *TOP3A* locus in Pa6. (F) Two reads highlighted in red and pink span the interval between target variants c.778C>T and c.1723A>G. (G) Read-level data for the haplotype assembled read (highlighted red), which is variant nucleotide supporting at position c.1723. (H) Read-level data used to create the assembled haplotype from reference supporting nucleotides at positions chr17:18195268 and chr17:18195287 (red and pink highlighted reads). (I) Read-level data for the haplotype assembled read (highlighted pink), which is reference supporting at position c.778. (J) A schematic illustration of the assembled haplotype for target variants c.778C>T and c.1723A>G, which are consistent with a *trans* configuration (arranged on different parental alleles). Nomenclature provided according to transcript NM_004618.5.

 © 2023 The Authors

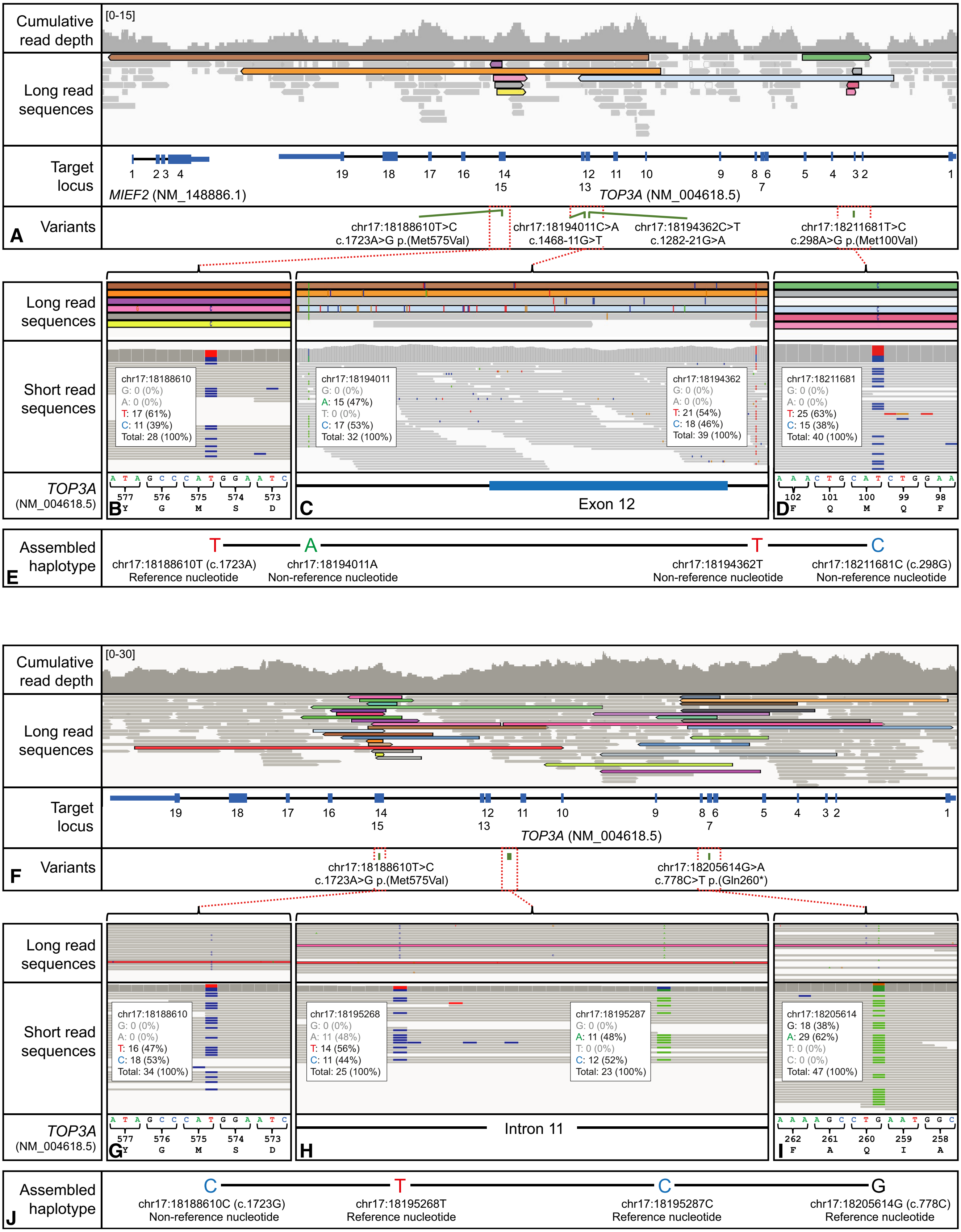
Cumulative read depth
[0-15]
Long read sequences
Target locus
1 2 3 4
19 18 17 16 14 15 12 13 11 10 9 8 6 7 5 4 3 2 1
MIEF2 (NM_148886.1)
TOP3A (NM_004618.5)
A
Variants
chr17:18188610T>C
c.1723A>G p.(Met575Val)
chr17:18194011C>A
c.1468-11G>T
chr17:18194362C>T
c.1282-21G>A
chr17:18211681T>C
c.298A>G p.(Met100Val)
Long read sequences
Short read sequences
chr17:18188610
G: 0 (0%)
A: 0 (0%)
T: 17 (61%)
C: 11 (39%)
Total: 28 (100%)
chr17:18194011
G: 0 (0%)
A: 15 (47%)
T: 0 (0%)
C: 17 (53%)
Total: 32 (100%)
chr17:18194362
G: 0 (0%)
A: 0 (0%)
T: 21 (54%)
C: 18 (46%)
Total: 39 (100%)
chr17:18211681
G: 0 (0%)
A: 0 (0%)
T: 25 (63%)
C: 15 (38%)
Total: 40 (100%)
TOP3A
(NM_004618.5)
577 576 575 574 573
B
C
Exon 12
102 101 100 99 98
D
E
Assembled haplotype
T A T C
chr17:18188610T (c.1723A)
Reference nucleotide
chr17:18194011A
Non-reference nucleotide
chr17:18194362T
Non-reference nucleotide
chr17:18211681C (c.298G)
Non-reference nucleotide
Cumulative read depth
[0-30]
Long read sequences
Target locus
19 18 17 16 14 15 12 13 11 10 9 8 6 7 5 4 3 2 1
TOP3A (NM_004618.5)
F
Variants
chr17:18188610T>C
c.1723A>G p.(Met575Val)
chr17:18205614G>A
c.778C>T p.(Gln260*)
Long read sequences
Short read sequences
chr17:18188610
G: 0 (0%)
A: 0 (0%)
T: 16 (47%)
C: 18 (53%)
Total: 34 (100%)
chr17:18195268
G: 0 (0%)
A: 11 (48%)
T: 14 (56%)
C: 11 (44%)
Total: 25 (100%)
chr17:18195287
G: 0 (0%)
A: 11 (48%)
T: 0 (0%)
C: 12 (52%)
Total: 23 (100%)
chr17:18205614
G: 18 (38%)
A: 29 (62%)
T: 0 (0%)
C: 0 (0%)
Total: 47 (100%)
TOP3A
(NM_004618.5)
577 576 575 574 573
G
H
Intron 11
262 261 260 259 258
I
J
Assembled haplotype
C T C G
chr17:18188610C (c.1723G)
Non-reference nucleotide
chr17:18195268T
Reference nucleotide
chr17:18195287C
Reference nucleotide
chr17:18205614G (c.778C)
Reference nucleotide

Figure EV1.

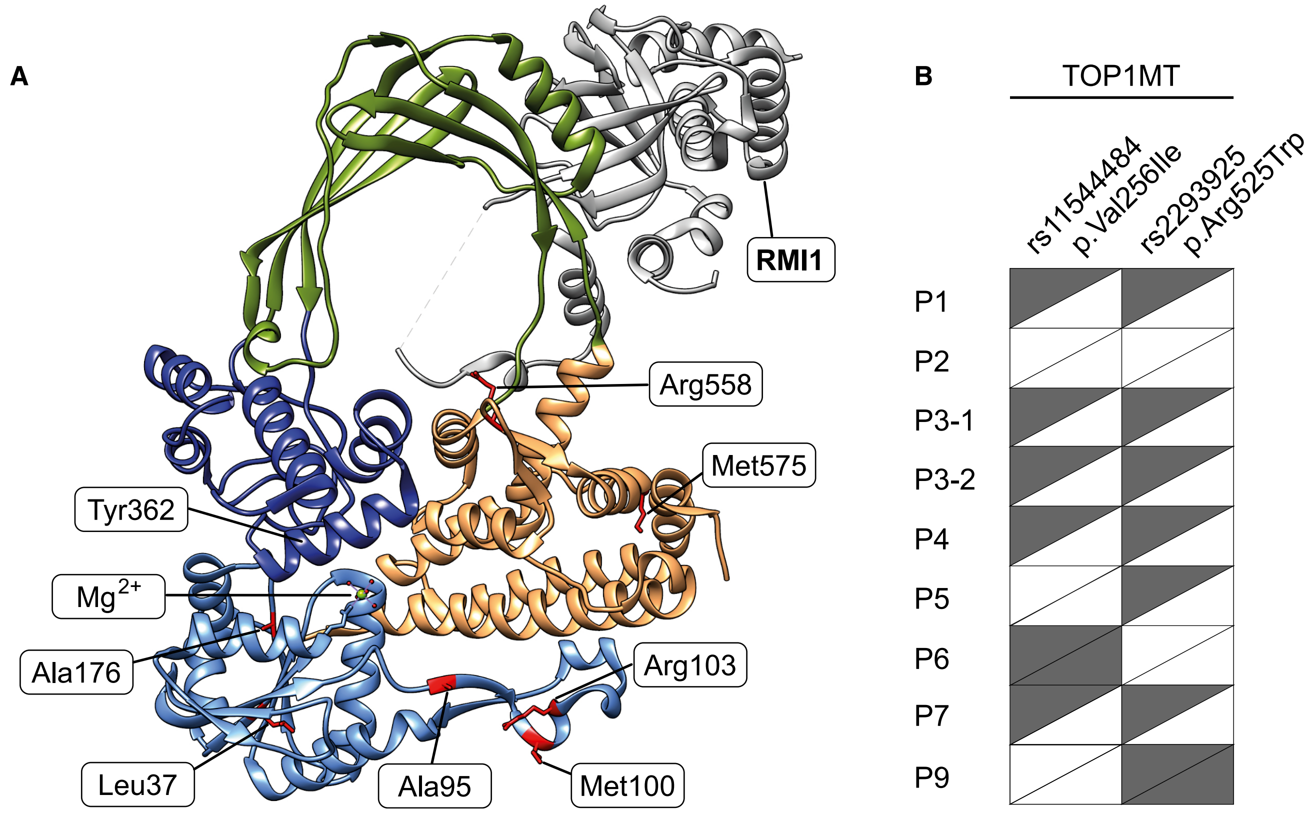

Figure EV2. Modelling of TOP3A variants and SNPs.

A The location of pathological variants within the crystal structure of TOP3A, including RMI1, a nuclear-binding partner of TOP3A (PDB: 4CGY). The affected residues are shown in red, and domains are coloured according to format shown in Fig 1B.

B The presence of previously identified SNPs in TOP1MT (rs11544484, p.Val256Ile; and rs2293925, p.Arg525Trp; Zhang *et al*, 2017) in TOP3A patients reported in this study for which the information is available. Half-filled rectangles indicate a heterozygous SNP, and filled rectangles indicate a homozygous SNP.

Source data are available online for this figure.

 © 2023 The Authors

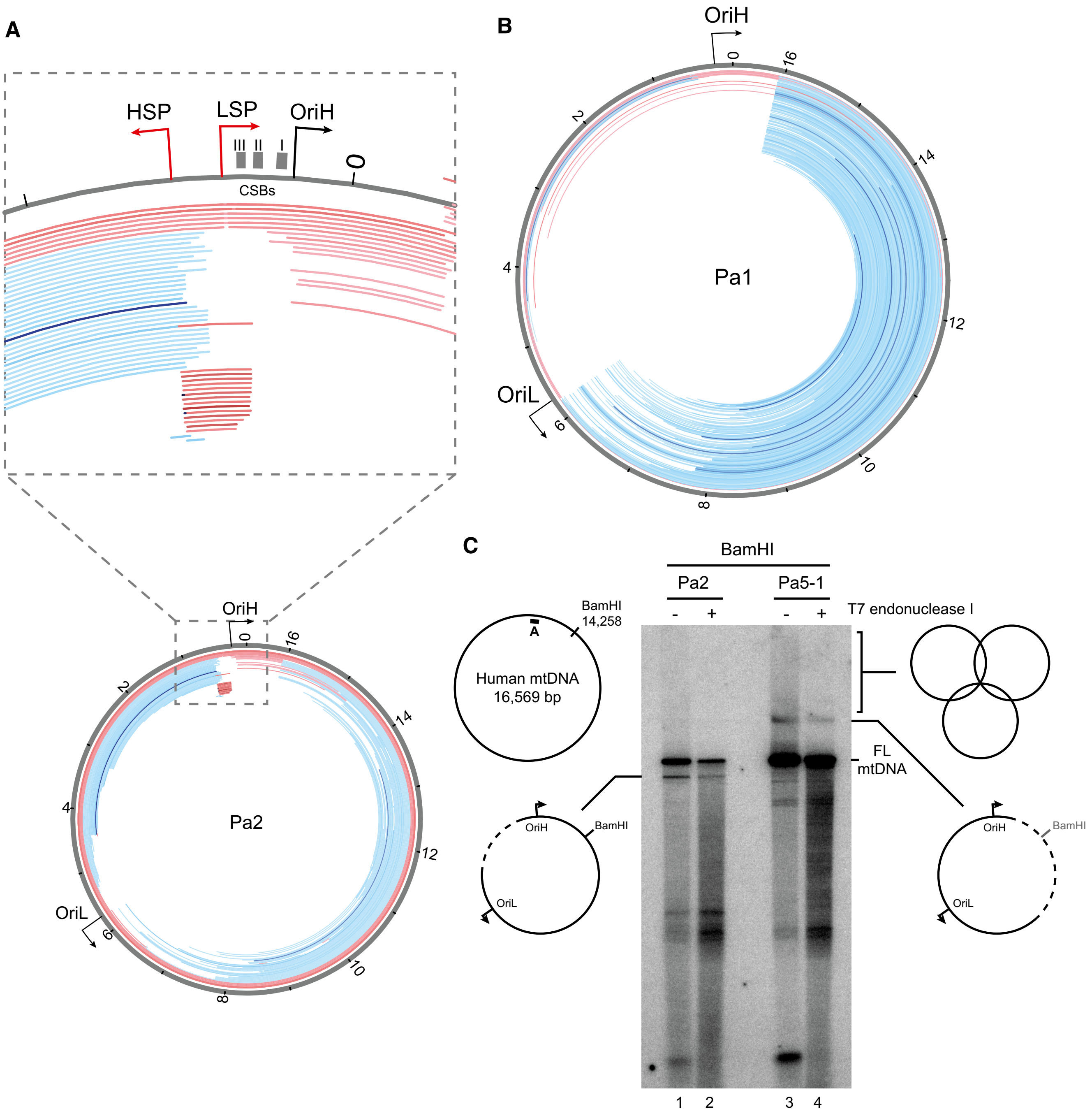

Figure EV3. mtDNA rearrangements in patient muscle.

A Enlargement of the NCR region in whole-genome sequence data of Pa2, indicating the locations of small mtDNA rearrangements. The locations of mtDNA *cis*-elements in this region are indicated (LSP, light-strand promoter; HSP, heavy-strand promoter; OriH, origin of heavy-strand replication; CSBs, conserved sequence blocks).

B Mitochondrial DNA rearrangements from Pa1 (Nicholls *et al*, 2018). Whole-genome sequencing data were analysed using the same version of the MitoSAlt pipeline as for newly reported individuals (Fig 2) to permit comparison of breakpoint mapping datasets. Deleted regions are shown as blue bars and predicted duplicated regions as red bars. The intensity of the colour corresponds to the abundance of the rearrangement.

C Analysis of mtDNA rearrangements in Pa2 and Pa5-1 using Southern blotting. Total muscle DNA (250 ng) was restricted with BamHI, and then left further untreated or incubated with 1 U of T7 endonuclease I, separated on agarose and Southern blotted using probe A (indicated with a black bar). Diagrams indicate the structures visible.

Source data are available online for this figure.

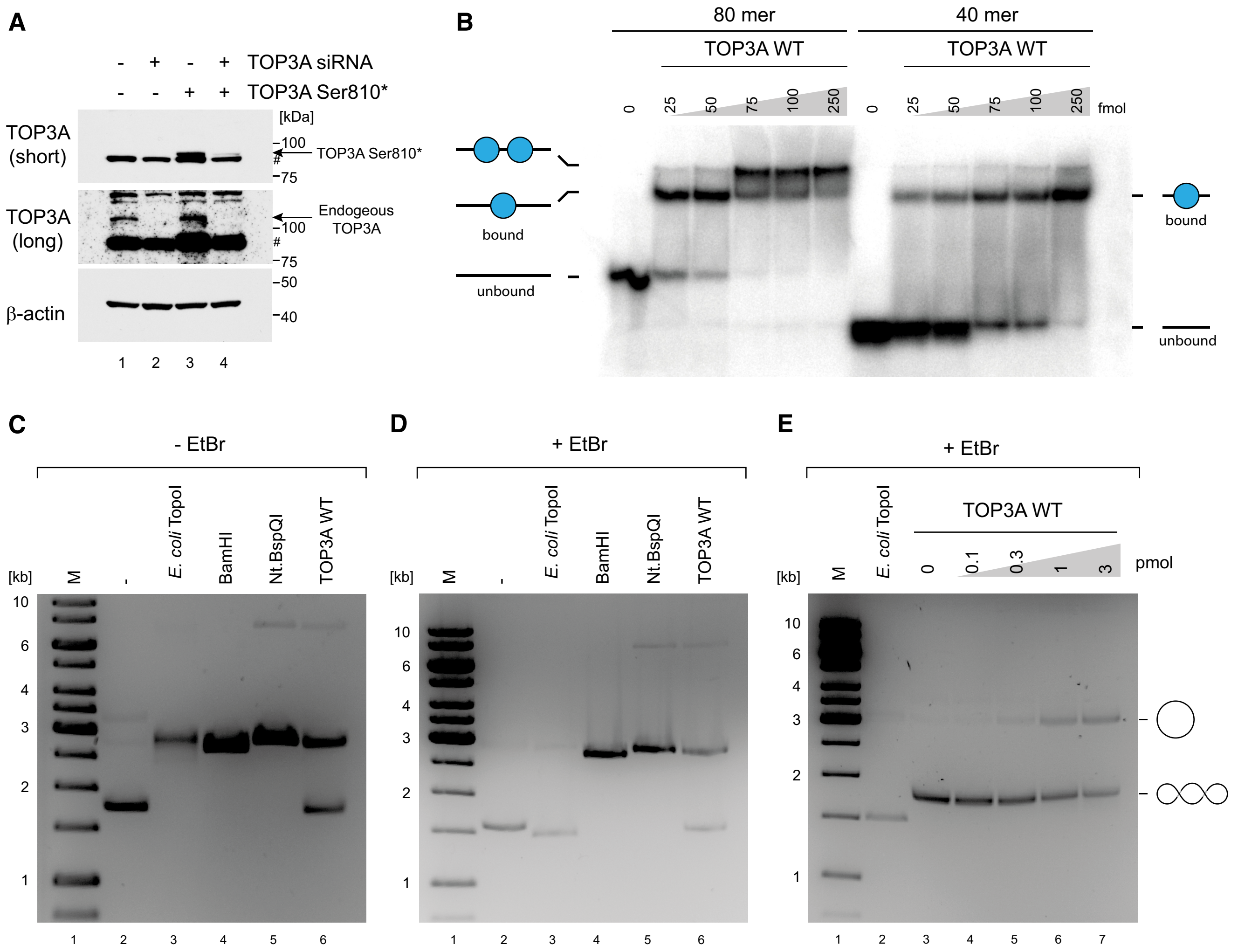

Figure EV4. Control reactions for EMSA and relaxation assays.

A Western blot of HeLa cells treated with TOP3A siRNA, with or without transient expression of TOP3A Ser810*. The band corresponding to TOP3A Ser810* migrates close to a non-specific band (indicated with #). β-actin is used as a loading control.

B Electrophoretic mobility shift assay (EMSA) using either 80-mer or 40-mer ssDNA substrates. Diagrams indicate singly and doubly shifted substrate.

C, D Controls for substrate migration in plasmid relaxation assays. Negatively supercoiled pUC19 plasmid DNA was either left untreated (lane 2) or treated with the indicated enzymes. *E. coli* TopoI-treated plasmid DNA (lane 3) shows the migration of covalently closed relaxed DNA, BamHI-treated DNA (lane 4) shows the migration of linearised plasmid and Nt.BspQI-treated DNA (lane 5) shows the migration of nicked circular plasmid. Reactions were separated either in the absence of ethidium bromide (EtBr) and post-stained for imaging (C) or run in the presence of EtBr (D). The migration of relaxed open, circular DNA and supercoiled DNA is indicated to the right of figure.

E Controls for TOP3A nicking activity. Negatively supercoiled pUC19 plasmid DNA was incubated with WT TOP3A protein as in Fig 5A, then separated on an agarose gel containing EtBr. The migration of nicked DNA and supercoiled DNA is indicated to the right of the figure. M" indicates marker.

Source data are available online for this figure.

 © 2023 The Authors

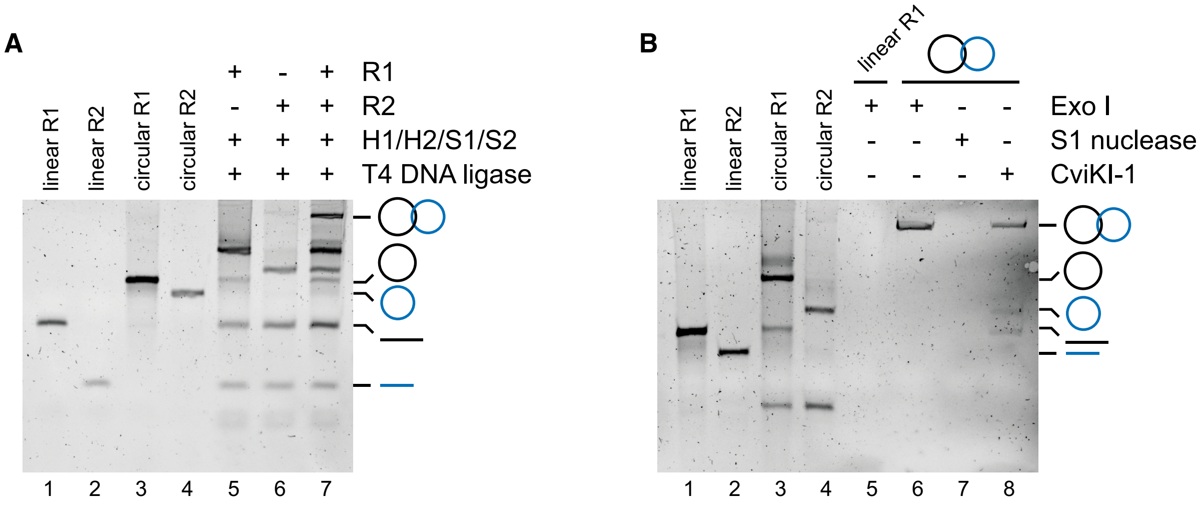

Figure EV5. ssDNA catenane substrate construction and validation.

A Synthesis of Lk1 ssDNA catenanes. Linear and circular R1 and R2 oligos are shown for size comparison (lanes 1–4). Products resulting from synthesis reactions omitting R2 (lane 5) or R1 (lane 6) are shown, as well as a complete synthesis reaction (lane 7). The migration of Lk1 ssDNA catenanes and circular and linear R1 and R2 oligos is indicated to the right of figure.

B Verification of Lk1 ssDNA catenanes. Linear and circular R1 and R2 oligos are shown for size comparison (lanes 1–4). The treatment of linear R1 oligo with ExoI (lane 5) is shown as a positive control for ExoI activity. Lk1 ssDNA catenanes were treated with ExoI (lane 6), S1 nuclease (lane 7) or CviKI-1 (lane 8). The migration of Lk1 ssDNA catenanes and circular and linear R1 and R2 oligos is indicated to the right of the figure.

Source data are available online for this figure.

Appendix for:

"Pathological variants in *TOP3A* cause distinct disorders of mitochondrial and nuclear genome stability"

Direnis Erdinc, Alejandro Rodriguez-Luis *et al.*

Contents: **Page**

Appendix Supplementary Text: Clinical Summaries for Families I-IX 2

Appendix Table S1: Oligonucleotides used 8

Appendix Supplementary Text - Clinical Summaries

Patient (Pa1)

Patient Pa1 is a 72 year-old female who was reported to have bilateral ptosis and complex ophthalmoplegia at the age of 43; gait ataxia and falls were noted from the age of 50. There was a history of bipolar disease and sensorineural hearing loss. Examination revealed bilateral ptosis, complex ophthalmoplegia, cerebellar ataxia, and retained reflexes. ECG showed sinus rhythm with ventricular bigeminy and left axis deviation (consistent with a left anterior hemi block). Echocardiogram was normal at the age of 50. Progression of disease was noted in all areas with the requirement for permanent pacemaker, and also the emergence of the significant proximal myopathy. An axonal sensory motor neuropathy was identified on nerve conduction studies (NCS), causing predominantly sensory symptoms. Left ventricular dysfunction was noted on echocardiogram and it improved with treatment. Subsequent to our previously published studies (Nicholls et al., 2018), a pathogenic *DSP* variant was identified which was felt to be consistent with the cardiac phenotype. Brain MRI revealed bilateral symmetrical changes within the thalami, red nuclei and long tracts within the midbrain, in addition to cerebellar atrophy. There was no other relevant family history.

Patient (Pa2)

Patient Pa2 is a 39 year-old female who developed problems with coordination and gait in her early twenties. She was found to have both cerebellar and sensory ataxia, with distal muscle weakness, foot drop and wasting. She has a bilateral ptosis but no ophthalmoplegia, hearing loss, nasal speech and dysphagia. She also showed dysautonomia with postural hypotension. NCS showed an axonal sensory motor neuropathy. Brain MRI showed cerebellar and spinal cord atrophy, with a lactate peak on brain MRS. Serum lactate 1.1mmol/l. CSF lactate 1.4mmol/l. Left bundle branch block developed on ECG at age 30, progressing to trifascicular block by age 32 requiring pacemaker insertion. Echocardiogram was normal apart from mitral valve prolapse. There was no other relevant family history, both parents and a sibling were clinically unaffected.

Patient (Pa3-1)

Patient Pa3-1 is a 64 year-old male who developed ptosis in his early 50’s, and was subsequently found to have a complex ophthalmoplegia. There was a past history of deafness and an isolated seizure at age 56. His younger sister (Patient 3-2; see below) was similarly affected. Examination revealed bilateral ptosis and advanced complex ophthalmoplegia. Mild bi-facial weakness was evident. There was evidence of a mild proximal myopathy and also features of a peripheral neuropathy with distal weakness, sensory loss and a positive Romberg’s test. The patient had an ataxic gait and was unable to heel toe walk. MRI brain scan revealed bilateral symmetric high signal within the brain stem, and the globus pallidus and antero-medial thalami. Calcification was noted in the dentate, caudate, lentiform and thalamic nuclei. Non-specific high signal lesions were noted within the white matter. Cerebellar atrophy was present. EMG/nerve conduction studies revealed an axonal sensory motor neuropathy and myopathic changes. An EEG showed marked photosensitivity. His mother was reported to have epilepsy and deafness, but no ophthalmoplegia, myopathy or neuropathy. No further details regarding the diagnosis of his mother’s epilepsy were available.

Patient (Pa3-2)

Patient Pa3-2, a 59 year-old female, is the clinically-affected sibling of Patient 3-1. She was diagnosed with photosensitive epilepsy and myoclonus, both of which were well controlled. She was noted to have bilateral ptosis in her late 40’s and subsequently found to have a complex ophthalmoplegia. Facial power was normal. She has dysarthria, dysphagia (requiring modified diet), mild right-sided hemiplegia (MRC 4/5) and cerebellar ataxia. She was unable to heel toe walk and had evidence of some proprioceptive issues. She was reported to have previously suffered a supraventricular tachycardia and left ventricular hypertrophy was detected on echocardiogram and cardiac MRI. She was documented to have had a premature menopause. Brain CT scan, following an RTA and orbital fracture, showed mild generalised atrophy consistent with age.

Patient (Pa4)

Patient Pa4 is a 27 year-old female, who first presented at the age of 16 with headache and encephalopathy. She has continued to suffer recurrent encephalopathic episodes, characterised by confusion and neurological deficits, some of which were associated

with pyrexia. After the first episode a progressive sensorineural hearing loss developed, unresponsive to steroids, and she currently uses hearing aids. No ptosis or ophthalmoplegia was noted and she had no clinical presentation of myopathy or ataxia. Although she returned to the usual cognitive baseline between episodes, she developed mild difficulties with executive functions. At the age of 24, she was hospitalised for a similar episode, and required rehabilitation for gait difficulties. During her first pregnancy (age 22) she suffered from preeclampsia. MRI brain scans revealed bilateral and symmetrical T2 hyperintensities in the globus pallidus, thalamus and brain stem (predominantly in the midbrain). Upon laboratory testing, CSF analysis and viral studies were unremarkable. Extended autoantibody screens were normal. Electroencephalogram (EEG) during these episodes demonstrated diffuse generalized slowing, and treatment with antiepileptic drugs was initiated**.** At the age of 25, she developed presyncope and complete atrioventricular block was observed. Echocardiogram revealed normal cardiac function. Cardiac MRI was not available. Cardiac biopsy was performed, but results were non-specific. Nerve conduction study (NCS) revealed an axonal sensory motor neuropathy without myopathic features on electromyography. There was no other relevant family history demonstrated amongst her unrelated parents or four siblings.

Patient (Pa5-1)

Patient Pa5-1 is a 72 year-old female who reported eyelid ptosis and the emergence of diplopia at the age of 59. She was found to have bilateral ptosis and a complex ophthalmoplegia. There was evidence of a subtle proximal myopathy, but no neuropathic features. She has a past medical history of asthma, cholecystectomy and hypertension. Her younger sister (Patient 5-2; see below) was similarly affected. Her mother and father were both clinically unaffected but passed away at the ages of 75 and 56 respectively. She does not have children. An ECG and echocardiogram at the age of 71 were both normal.

Patient (Pa5-2)

Patient Pa5-2, a 66 year-old female, is the clinically-affected sibling of Patient 5-1. She originally sought opinion for diplopia at the age of 59 and was diagnosed with bilateral ptosis and a complex ophthalmoplegia. There were no symptoms or signs of either myopathy or neuropathy. Cardiac investigations for unexplained collapses were

unremarkable (including echocardiogram and an implantable loop recorder). She had previously suffered pancreatitis and subsequent gall bladder surgery. There was no other relevant family history aside from her clinically-affected sister.

Patient (Pa6)

Patient Pa6 is a 52 year-old female who complained of weakness and wasting bilaterally in the hands. She was referred to the Neurology department where she was found to have bilateral ptosis and complex ophthalmoplegia, bilateral sensorineural hearing loss (reported to date back several decades), proximal weakness and evidence for a sensory motor peripheral neuropathy, with distal wasting, areflexia and reduced sensation to the mid shin. Nerve conduction studies confirmed an axonal sensory motor neuropathy. There was no relevant family history; her unrelated parents, her brother and two children are clinically normal. An ECG showed inverted T-waves in the lateral chest leads and some anterior Q-waves. An MRI brain scan showed extensive white matter changes. These were consistent with small vessel ischemic change. It was noted that there were limited vascular risk factors.

Patient (Pa7)

Patient Pa7 is a 59-years-old male with a 5-years history of progressive imbalance. His symptoms were more noticeable in environments without adequate illumination, and he subsequently developed dysarthria, changes in the volume of his voice and dysphagia. His father had developed balance problems in his early 80s, but none of his relatives had received a diagnosis of ataxia. Physical examination showed ophthalmoparesis in the up-gaze and lateral gaze, with preserved down-gaze, and slowness of saccades. Motor examination was normal, apart from areflexia in both Achilles tendons. Vibration and proprioception were impaired in both lower limbs distally, and pin-prick test revealed hypoesthesia in the feet. He showed mild dysmetria in the four limbs and broad-based gait. The Neuro-Ophthalmological examination showed normal visual acuity and colour vision, and ptosis with frontalis overaction. Fundoscopy revealed a pigmentary retinopathy, with preserved retinal nerve fibre layer.

Over the years, he experienced slow progression of his ataxia (aged 62, his SARA score was 13/40) and oculomotor signs (aged 71, he presented with total ophthalmoplegia with horizontal diplopia). Paraesthesia in both lower limbs, urinary

symptoms (nocturia, increased frequency and urgency) and restless leg syndrome (RLS) were also reported in his last follow-up. He has received treatment with Co-enzyme Q10 (200 mg, tds), with improvement in his fatigue levels, and for his RLS symptoms.

Brain MRI showed moderate cerebellar atrophy, with mild atrophy in the brainstem (midbrain and pons). EMG/NCS revealed a length-dependent axonal sensorimotor polyneuropathy. Neuropsychological assessment identified mild cognitive under-functioning, implicating anterior and bitemporal regions. Muscle biopsy showed SDH-positive/COX-negative fibres and mild changes secondary to denervation. Genetic testing was negative for FRDA, SCA1, SCA2, SCA3, SCA6, SCA7, POLG, PEO1, GFPT1, CHRNE. Sequencing of the whole mtDNA in muscle was normal, and no large scale mtDNA rearrangements were observed. There was no evidence of mitochondrial respiratory chain enzyme deficiency in the muscle biopsy.

Patient (Pa8)

Patient Pa8 is a 42 year old female with myopathy, dysphagia, dysarthria, sensorineural hearing loss, cardiac conduction defects, chronic progressive external ophthalmoplegia, and ptosis. She initially presented at age 21 with syncope and was found to have cardiac conduction defects requiring pacemaker placement. She was then diagnosed with sensorineural hearing loss about 10 years later, and shortly after that she began to notice muscle weakness and swallowing difficulties. EMG/NCV at that time revealed moderate sensory axonal polyneuropathy. Muscle biopsy revealed ragged-red fibres consistent with mitochondrial disease. She requires g-tube feedings due to silent aspiration identified on swallow study. Her muscle weakness has worsened over time, and she now has respiratory muscle involvement with decreased FVC (60%) as well as cardiomyopathy with ejection fraction (EF) of 30-35%.

Patient (Pa9)

Patient Pa9 is a 41-year-old female, born to consanguineous parents, who at the age of 15 developed focal onset seizures presenting with right arm and language abnormalities and no postictal state, accompanied by worsening of academic performance. At the age of 17, a posterior fossa arachnoid cyst was diagnosed. She required placement of a cardiac pacemaker at the age of 21, due to syncope and

arrhythmia and ever since she was seizure free. At the age of 26, she developed distal weakness, atrophy of the distal limbs' musculature and sensory abnormalities. She had early menopause at the age of 28. Posterior fossa arachnoid cyst was drained surgically at the age of 33, with no improvement in the neurological condition that continued to progress. At the age of 38, sensorineural hearing loss was identified. She was evaluated in our service at age 40, presenting a steppage gait with the necessity of unilateral support. In addition, she presented with mild dysmetria and dysarthria. Bilateral eyelid ptosis, ophthalmoparesis with alteration of the vestibulo-ocular reflex were also identified. Vibration, tactile and pain sensory modalities were compromised in distal lower limbs, and she presented global areflexia. Nerve conduction studies depicted an axonal sensory and motor polyneuropathy. Tonal audiometry revealed bilateral neurosensory hearing loss. Brain CT scan only showed the posterior fossa arachnoid cyst and brain MRI was not performed due to the cardiac pacemaker placement.

Appendix Table S1 *- Oligonucleotides used in this study*

'p' indicates 5' phosphorylation, regions in bold denote homology.

Name	Sequence (5'-3')
R1	p-GACTAGAGCAGATATAATACGACT**CACTATAGGG**ATACAATAGGCAGCTGGACGTGTACCAAGTTAGCAGTCATG
R2	p-ATGTCTGG*TTCGTCTCACGACTCATCACG****CCCTATAGTG****GA*TCAGCACACATCATATCACAGC
H1	GTACACGTCCAGCTGCCTATTGTAT CGTGATGAGTCGTGAGACGAA
H2	GTGCTGATCAGTCGTATTA
S1	CTAGTCCATGAC
S1	AGACATGCTGTG
R1 Probe	TCTGCTCTAGTCCATGACTGCTAAC
R2 Probe	CGAACCAGACATGCTGTGATATGAT
Probe A Forward	CTCACCCACTAGGATACCAAC
Probe A Reverse	GATACTGCGACATAGGGTGC
EMSA substrate (80 mer)	ACCACATTAACAACATAAAACCCTCATTCACACGAGAAAACACCCTCATGTTCATACACCTATCCCCCATTCTCCTCCTA
EMSA substrate (40 mer)	TGTTATTATTATGTCCTACAAGCATTAATTAATTAACACA
TOP3A siRNA s14310	CGGCUUGCCUAGUUCUCUAtt
