# Supplementary material for: Pathological variants in TOP3A cause distinct disorders of mitochondrial and nuclear genome stability
Source: EMBO Mol Med. 2023 Apr 4;15(5):e16775. doi: 10.15252/emmm.202216775 (PMC10165364; doi:10.15252/emmm.202216775)
Supplement: Supplementary file 10 [file EMMM-15-e16775-s003.pdf]

EMBO Molecular Medicine

Pathological variants in TOP3A cause distinct disorders of mitochondrial and nuclear genome stability

Direnis Erdinc, Alejandro Rodríguez-Luis, Mahmoud Fassad, Sarah Mackenzie, Christopher Watson, Sebastian Valenzuela, Xie Xie, Katja Menger, Kate Sergeant, Kate Craig, Sila Hopton, Gavin Falkous, Joanna Poulton, Hector Garcia-Moreno, Paola Giunti, Carlos de Moura Aschoff, Jonas Saute, Amelia Kirby, Camilo Toro, Lynne Wolfe, Danica Novacic, Lior Greenbaum, Aviva Eliyahu, Ortal Barel, Yair Anikster, Robert McFarland, Grainne Gorman, Andrew Schaefer, Claes Gustafsson, Robert Taylor, Maria Falkenberg, and Thomas Nicholls
DOI: 10.15252/emmm.202216775

Corresponding author: Thomas Nicholls (thomas.nicholls@newcastle.ac.uk)

Review Timeline:

Submission Date:	19th Aug 22
Editorial Decision:	23rd Sep 22
Revision Received:	31st Jan 23
Editorial Decision:	28th Feb 23
Revision Received:	10th Mar 23
Accepted:	14th Mar 23

Editor: Zeljko Durdevic

Transaction Report:

(Note: With the exception of the correction of typographical or spelling errors that could be a source of ambiguity, letters and reports are not edited. Depending on transfer agreements, referee reports obtained elsewhere may or may not be included in this compilation. Referee reports are anonymous unless the Referee chooses to sign their reports.)

23rd Sep 2022

Dear Dr. Nicholls,

Thank you for the submission of your manuscript to EMBO Molecular Medicine. We have now received feedback from the three reviewers who agreed to evaluate your manuscript. As you will see from the reports, all three referees recognize potential interest of the study but also raise serious concerns, particularly regarding limited understanding of the disease mechanism, that should be addressed in a major revision.

Further consideration of a revision that addresses reviewers' concerns in full will entail a second round of review. EMBO Molecular Medicine encourages a single round of revision only and therefore, acceptance or rejection of the manuscript will depend on the completeness of your responses included in the next, final version of the manuscript. For this reason, and to save you from any frustrations in the end, I would strongly advise against returning an incomplete revision.

We would welcome the submission of a revised version within three months for further consideration. Please let us know if you require longer to complete the revision.

Please use this link to login to the manuscript system and submit your revision: https://embomolmed.msubmit.net/cgi-bin/main.plex

I look forward to receiving your revised manuscript.

Yours sincerely,

Zeljko Durdevic

Zeljko Durdevic
Editor
EMBO Molecular Medicine

.

***** Reviewer's comments *****

Referee #1 (Remarks for Author):

This manuscript from Erdinc et al. titled 'Pathological variants in TOP3A cause distinct disorders of mitochondrial and nuclear genome stability' is a novel study that identifies TOP3A variants causing mitochondrial disease. This finding is interesting, as previous pathogenic variants in TOP3A have been primarily only recognized as causing a disease similar to Bloom Syndrome, which is caused by pathogenic variants in the TOP3A binding partner BLM. As BLM is only found in the nucleus and does not appear to have a mitochondrial function, Bloom Syndrome is largely considered to be caused by impaired maintenance of the nuclear genome, rather than the mtDNA. Similarly, the Bloom-like syndrome caused by TOP3A variants is also considered to be caused by impaired maintenance of the nuclear genome.

The authors describe a cohort of eleven individuals from 9 families with a variety of pathogenic variants in TOP3A and convincingly show that mtDNA maintenance is impaired. A subset of patient muscle samples was analyzed for pathological phenotypes including histochemical staining, mitochondrial Oxphos complex activity, mtDNA deletions/rearrangements, and mtDNA structure. The shared patient phenotypes among this group provides strong support that this new disorder is caused by TOP3A variants, which is further supported by the biochemical studies that comprise the bulk of the manuscript. Functionally, they used in vitro assays to look at DNA binding, DNA relaxation, and decatenation.

A key question addressed in this manuscript is why TOP3A dysfunction can lead to two distinct phenotypes, namely a Bloom-like syndrome, or the newly described mitochondrial disease. In this regard their in vitro biochemical studies also examined TOP3A variants previously linked to Bloom-like syndrome. My take on their data is that the Bloom-like syndrome is caused when a patient has a combination of alleles that leads to severely reduced TOP3A enzymatic activity, while the novel mitochondrial disease is caused by a combination of alleles that leads to moderate loss of TOP3A activity. Overall, the findings in this manuscript are novel, and the data provided are strong. I do have a few comments and suggestions listed below.

My biggest issue is that I felt a bit misled by the abstract, as I was expecting the impairment of biochemical functions to differ between the variants causing Bloom vs Mito disease (e.g., DNA binding vs DNA relaxation). Upon re-reading the abstract, it does not say this, but it also doesn't clearly state the main finding, which is that the severity of loss of enzymatic activity that determines the patient phenotype. I think adding a sentence to the abstract that discusses the severity of enzymatic loss as being the basis for the patient phenotype would avoid having the reader infer what is meant by 'defects in nuclear and mitochondrial functions'.

My understanding of how TOP3A ends up in the mitochondria or the nucleus (from what is written in the introduction) is that this is due to inefficient targeting to the mitochondria, rather than due to splice isoforms or alternative translation start sites, as are sometimes observed for dual-localized nuclear/mito proteins. In this case, if the protein sequences are exactly the same, are the nuclear and mitochondrial versions of the protein truly distinct isoforms?

On line 346, could the authors expand on what they mean by 'phase was established', as some readers may not be familiar with the term.

Line 349-350 states "all patients carry two (likely compound) het TOP3A variants", but there are also two patients who are homozygous for Arg103Gln. As such, the text needs to be corrected to accurately reflect that there are patients with homozygous variants. Further, could the authors comment further on what they mean by 'likely heterozygous'. I assume that they mean they cannot confirm the heterozygosity based solely on their existing data, which is fine. However, they should simply mention this as a potential limitation.

Regarding the available crystal structure, I gather this does not include the zinc finger domain. It might be worth briefly commenting on what is missing from the structure, and why the structure is incomplete (if this is known).

Looking at the potential overlap with the common TOP1MT variants was an interesting idea, but I wonder if there are any common TOP3A variants that might also be relevant?

Regarding the analysis of catenated mtDNA products (Fig 2, lines 445-447), it may be worth explaining how you get unresolved catenated mtDNA products when the mtDNA is linearized with a restriction enzyme, as this may not be immediately evident.

For Figure 3 (DNA binding assay), there are two shifted bands. What do the two different shifted bands represent, and are both considered as bound with respect to the quantification for Fig 3K? Does TOP3A form a dimer, and if so would this impact the mechanism of disease? Would it be worth combining multiple variants in the in vitro biochemical assays to see how variants may interact? Could mutant variants decrease WT protein activity in a dominant negative way?

In the discussion, the authors may want to speculate on some of the potential disease modifying effects, especially why the female patients seem to present earlier than male patients.

Referee #2 (Comments on Novelty/Model System for Author):

Mutation discovery follows the standards in the field and is perhaps unexciting routine work for most medical genetics laboratories. The effect of the mutations have been only characterised in vitro, which, while adequate, brings only limited information about the disease mechanisms.

Referee #2 (Remarks for Author):

The manuscript by Erdinc et al. describes a number of new pathological variants in TOP3A, resulting in mtDNA rearrangements, COX dysfunction and mitochondrial diseases with varying representation and age of onset. The findings are highly interesting, and the experimental work well conducted, although characterising the mutant phenotypes only in vitro brings rather limited information about the disease mechanisms. Introduction and discussion could be expanded but the overall presentation of the manuscript is well structured and almost flawless English.

More specifically,

Introduction:
L86: Maybe worth mentioning that the mitochondrial targeting sequence is translated from an alternative start-codon?
L99: There seems to be a recent publication linking TOP3A to mtDNA replication progression as well.
https://doi.org/10.1093/nar/gkac660

L100: TOP3A is a Type I topoisomerase, so it cannot separate two interlocked double-stranded mtDNAs, which is basically meant by decatenation, also by definition, catenanes do not have a "junction" (L101). It seems that the authors are talking about hemicatenanes (mentioned only once in the discussion L503), which are quite different. It is mandatory that mtDNA replication, hemicatenane formation, resolution and their relevance to the experiments are properly introduced.

Methods
L200 Please provide sequence coordinates for the probe.

Results:
L428: Figure 3C should be 2C.
Mark the replication origins to the mtDNA maps on Fig. 2D-G. Many of the breakpoints around 16k seem oddly precise. This should be discussed. Overexpression experiments on TOP3A seem to result in specific breakage of mtDNA around the non-coding region (https://doi.org/10.1093/nar/gkac660). Can there be a connection between a sequence specific break point and the observed rearrangements?
Fig 2H and I: As the DNA has been digested, there should not be any catenated DNA left. What are the sizes of "non-16.6 kb" fragments in P2 and P5 samples?
L441 "interlinked mtDNAs". Related to comment for L100, so these are not catenanes, but I assume hemicatenanes are meant?
L447 "unresolved catenated mtDNA replication products". Catenanes should fall apart when the DNA is linearized. The size of the additional bands in P2 and P5 sample is different depending on the used restriction enzyme, indicating that the molecule is a partial duplication or then some non-linear species with a specific branch point region or a surprisingly stabile hemicatenane, which is different between the patients. This is an interesting observation and should be pursued further. To me, the fact that the

two patients have different additional bands strongly argues against simple "unresolved replication products". I suggest digesting the sample with different restriction enzymes yielding smaller fragment around the expected duplicated region to map it more carefully. However, judging from the sequencing results of the same sample, it can well be a junctional molecule of some type. For the scope of the work, it would be sufficient to differentiate between mtDNA sequence rearrangement (partial duplication) and persistent molecular structure, using e.g. two-dimensional gels.
Figure 4: I am astonished that the disease mutations are all basically catalytically inactive in relaxing DNA, which basically requires only nicking action. As the patients are compound heterozygous, does the combination rescue any activity? For example, would you expect the performance of 1:1 mixture of Met100Val+Met575Val to be better than one mutant alone?
L558: Do the authors suggest that the TOP3A mutations cause replication stalling, which results in mtDNA rearrangements? Please elaborate and discuss in more detail the proposed disease mechanism.
L567: Not only the patients survive, but the mutations result in rather late onset disease symptoms (the youngest also at their teens, Table 2). Considering how poorly the disease variants perform in the in vitro assays, I think this invites more discussion. It might be also good to point out that many mtDNA maintenance diseases show tissue specificity and varying age of onset depending on the mutations.
L587: Interestingly all of the mutants are really poor in relaxing supercoils and Met575Val seems also very impaired in decatenation, although it is associated with the late onset disease. Based on the results, the de-hemicatenation seems more function important than unwinding supercoils? Can you discuss more the functional importance of the different activities of TOP3A based on your findings?

Referee #3 (Remarks for Author):

This manuscript reported eleven individuals from nine families with adult-onset mitochondrial disease caused by compound heterozygous mutations in TOP3A gene. Through sequential COX-SDH histochemistry and quadruple immunofluorescence, the presence of mitochondrial myopathy was confirmed in patients with TOP3A mutations. Structural analysis of the mtDNA revealed the mtDNA instability in TOP3A patient muscle. Various methods were used to investigate the function of the TOP3A variants, confirming that pathological TOP3A variants cause a decrease in its activity of DNA binding, relaxation capacity, and decatenation. Overall, the paper confirms that TOP3A is a causative gene for adult mitochondrial diseases and try to provide a better understanding on TOP3A.

The clinical presentation, modelling, and muscle mitochondrial characterization from patients are quite good. I am worried about model proposed based on the biochemical characterization of TOP3A variants.

1. The content of abstract is not consistent with the title. The tile is "Pathological variants in TOP3A cause distinct disorders of mitochondrial and nuclear genome stability". However, the core results of biochemical characterization of TOP3A variants were not summarized in the abstract.
2. The model proposed by this article from the results was not quite reasonable. Take the DNA binding activity as an example, leu37Val and Met575Val variants of TOP3A from mitochondrial disease patients had significant loss of DNA binding activity, while Ala176Val and Ser810* of TOP3A variants from Bloom-like disease patients had comparable level of DNA binding activity to the WT protein. The model is that moderate loss of enzyme activity results in mitochondrial disease in adults and the severe loss of enzyme activity results in Bloom syndrome-like disorder. The model is the opposite of the results.

Thank you for taking the time to consider our manuscript. In addition to the specific points raised by the reviewers below, we have made the following changes:

(i) One new author, Dr Christopher Watson (University of Leeds) has been added to the paper on the basis of new work to establish the phase of compound heterozygous variants using long-read sequencing. All authors agree to this change to authorship. This data is described in our response to Reviewer 1 and is presented in Figure EV1.

(ii) In Figure 1A, the pedigrees for Family V and Family VI were mislabelled. We apologise for this oversight on our part, and this has now been corrected.

(iii) In Figure 6, a small alteration has been made to the quantification method in order to normalise the amount of product to the no-enzyme control, which we feel makes it easier to visualise the differences between variants. The underlying data and the conclusions of the experiment remain unchanged, and the method is described in the Materials and Methods section.

(iv) The figures have been reformatted to conform to the *EMBO Molecular Medicine* style, and now consists of 6 main figures and 5 Extended View figures. Figure 2 from the original submission has been divided into two following the inclusion of additional data, and references to figures in the main text have been updated.

***** Reviewer's comments *****

Referee #1 (Remarks for Author):

This manuscript from Erdinc et al. titled 'Pathological variants in TOP3A cause distinct disorders of mitochondrial and nuclear genome stability' is a novel study that identifies TOP3A variants causing mitochondrial disease. This finding is interesting, as previous pathogenic variants in TOP3A have been primarily only recognized as causing a disease similar to Bloom Syndrome, which is caused by pathogenic variants in the TOP3A binding partner BLM. As BLM is only found in the nucleus and does not appear to have a mitochondrial function, Bloom Syndrome is largely considered to be caused by impaired maintenance of the nuclear genome, rather than the mtDNA. Similarly, the Bloom-like syndrome caused by TOP3A variants is also considered to be caused by impaired maintenance of the nuclear genome.

The authors describe a cohort of eleven individuals from 9 families with a variety of pathogenic variants in TOP3A and convincingly show that mtDNA maintenance is impaired. A subset of patient muscle samples was analyzed for pathological phenotypes including histochemical staining, mitochondrial Oxphos complex activity, mtDNA deletions/rearrangements, and mtDNA structure. The shared patient phenotypes among this group provides strong support that this new disorder is caused by TOP3A variants, which is further supported by the biochemical studies that comprise the bulk of the manuscript. Functionally, they used in vitro assays to look at DNA binding, DNA relaxation, and decatenation.

A key question addressed in this manuscript is why TOP3A dysfunction can lead to two distinct phenotypes, namely a Bloom-like syndrome, or the newly described mitochondrial disease. In this regard their in vitro biochemical studies also examined TOP3A variants previously linked to Bloom-like syndrome. My take on their data is that the Bloom-like syndrome is caused when a patient has a combination of alleles that leads to severely reduced TOP3A enzymatic activity, while the novel mitochondrial disease is caused by a combination of alleles that leads to moderate loss of TOP3A activity. Overall, the findings in

this manuscript are novel, and the data provided are strong. I do have a few comments and suggestions listed below.

My biggest issue is that I felt a bit misled by the abstract, as I was expecting the impairment of biochemical functions to differ between the variants causing Bloom vs Mito disease (e.g., DNA binding vs DNA relaxation). Upon re-reading the abstract, it does not say this, but it also doesn't clearly state the main finding, which is that the severity of loss of enzymatic activity that determines the patient phenotype. I think adding a sentence to the abstract that discusses the severity of enzymatic loss as being the basis for the patient phenotype would avoid having the reader infer what is meant by 'defects in nuclear and mitochondrial functions'.

We agree that the abstract could be improved, and so we have rewritten the second half of the abstract to more clearly describe how the observed catalytic defect links to the clinical presentation. This now reads:

"We present a comprehensive characterisation of the effect of TOP3A variants, from individuals with mitochondrial disease and a Bloom-like syndrome, upon mtDNA maintenance and upon different aspects of enzyme function. Based on these results we suggest a model whereby the overall severity of the TOP3A catalytic defect determines the clinical outcome, with milder variants causing adult-onset mitochondrial disease and more severe variants causing a Bloom-like syndrome with mitochondrial dysfunction in childhood."

We have also expanded the Discussion to more clearly describe how the overall level of TOP3A catalytic activity is affected in patients carrying two compound heterozygous *TOP3A* variants, and how this determines the patient phenotype.

My understanding of how TOP3A ends up in the mitochondria or the nucleus (from what is written in the introduction) is that this is due to inefficient targeting to the mitochondria, rather than due to splice isoforms or alternative translation start sites, as are sometimes observed for dual-localized nuclear/mito proteins. In this case, if the protein sequences are exactly the same, are the nuclear and mitochondrial versions of the protein truly distinct isoforms?

It is assumed that the mitochondrial and nuclear isoforms of TOP3A are generated by alternative translation initiation, whereby translation of the full-length protein (from M1) leads to mitochondrial localisation, and the generation of an N-terminally truncated protein (from M26) leads to nuclear localisation (Wang *et al*. (2002) **99**(19): 12114-9; Nicholls *et al*. (2018) Mol Cell **69**(1): 9-23.
We have also recently published a study of the subcellular localisation of topoisomerases with a view to understanding their contributions to mtDNA maintenance and expression (Menger *et al*. (2022). *Nucleic Acids Res*; **50**(19): 11154-11174), which supports that the two isoforms are translated from these sites. The original characterisation of TOP3A targeting suggested that the MTS is cleaved at approximately position 21-22, which would result in the mitochondrial isoform of TOP3A being a few residues longer than the nuclear isoform at the N-terminus. We realise that this section of the Introduction could have been written more clearly, and so we have rewritten these sentences to help clarify the issue of subcellular TOP3A targeting. This section now reads:

"The TOP3A transcript contains two potential initiation codons, M1 and M26. Translation from the upstream start site generates a longer isoform bearing an N-terminal mitochondrial targeting sequence (MTS) that directs its import into mitochondria, while translation from the downstream initiation codon generates an N-terminally truncated protein that lacks the MTS and is directed for nuclear import (Menger et al., 2022; Nicholls et al., 2018; Tsai et al., 2016; Wang et al., 2002; Wu et al., 2010). Proteolytic cleavage of the MTS during

mitochondrial import leads to mature mitochondrial and nuclear isoforms that are almost identical at the N-terminus (Wang et al., 2002)."

On line 346, could the authors expand on what they mean by 'phase was established', as some readers may not be familiar with the term.

We have now briefly defined phase in this section as follows:

"To establish the phase of variants (the alleles from which variants are derived) in Families V and VI, . . ."

Line 349-350 states "all patients carry two (likely compound) het TOP3A variants", but there are also two patients who are homozygous for Arg103Gln. As such, the text needs to be corrected to accurately reflect that there are patients with homozygous variants. Further, could the authors comment further on what they mean by 'likely heterozygous'. I assume that they mean they cannot confirm the heterozygosity based solely on their existing data, which is fine. However, they should simply mention this as a potential limitation.

We have modified the text in this section to take account of patients carrying homozygous variants. This now reads:

*"Two patients (Pa4 and Pa9) were homozygous for the p.Arg103Gln variant, while all other patients carry two heterozygous TOP3A variants. It is interesting to note that Pa5-1, Pa5-2 and Pa7 harbour the same p.Met100Val TOP3A variant reported in the first reported case (Pa1, **Table 1**; (Nicholls et al., 2018)). The p.Met575Val variant also appears to be recurrent, and is shared by Pa2, Pa5-1 and 5-2, and Pa6."*

The Results text has also been modified to remove the reference to 'likely heterozygous' to avoid ambiguity, and to state that heterozygosity has been confirmed where appropriate samples were available.

In order to confirm the phase of variants for further patients for whom familial samples were not available, in the revised version of the manuscript we have used long-read sequencing to analyse the *TOP3A* variants of interest in two patients, Pa5-1 and Pa6. From this data, together with SNP data from the short-read sequencing datasets, the haplotypes could be reconstructed and indicate that the two variants (p.Met100Val + p.Met575Val for Family V, and p.Gln260* + p.Met575Val for Family VI) are arranged *in trans* for both families. These data are now presented as Figure EV1 in the revised manuscript, together with explanatory text in the Results and an accompanying Methods section.

Regarding the available crystal structure, I gather this does not include the zinc finger domain. It might be worth briefly commenting on what is missing from the structure, and why the structure is incomplete (if this is known).

The available crystal structure indeed lacks the C-terminal 360 amino acids, which includes a predicted zinc finger domain. Although none of the variants that we have studied are within this region, we cannot exclude that there are relevant interactions between these variants and the C-terminal domain. Crystal structures are available from bacterial TOP3 homologues that include the C-terminal zinc finger motifs, and some insight can be gained from these structures. We have expanded this section of the Results to take account of this. The revised text now reads:

"It should be noted that the truncated version of TOP3A used in the available crystal structure (Bocquet et al., 2014) omits approximately the C-terminal ~360 amino acids, containing a finger domain, most likely due to high flexibility. It therefore cannot be

*determined whether this domain forms further relevant interactions. However, the zinc finger domain has been solved in structures of both Escherichia coli topoisomerase I (EcTOP1) and Mycobacterium smegmatis topoisomerase I (MsmTOP1). In these enzymes the zinc finger domain binds to the intact strand of ssDNA in the strand passage reaction (T-strand), while the opposite strand (G-strand) is positioned at the active site for cleavage (Cao et al., 2020; Tan et al., 2015). The p.Met575Val variant is found close to the region, located at the junction between the catalytic core of TOP3A and the zinc finger domain (**Figure 1E**, panel vii), and may affect the communication between the N-terminal and C-terminal parts of TOP3A and how they interact with ssDNA."*

Looking at the potential overlap with the common TOP1MT variants was an interesting idea, but I wonder if there are any common TOP3A variants that might also be relevant?

Thank you for raising this. The pathological *TOP3A* variants that we describe are unlikely to be the only changes in this gene observed following high-throughput sequencing approaches, although variants that are common or very likely benign would have been bioinformatically filtered out of the final dataset for clinical review. What we are able to say is that we did not identify additional TOP3A variants that were likely to impact TOP3A enzyme activity and that any functional validation of common variants is very much beyond the scope of this manuscript. Our data suggest that there are not 'modifier' variants within the same gene.

Regarding the analysis of catenated mtDNA products (Fig 2, lines 445-447), it may be worth explaining how you get unresolved catenated mtDNA products when the mtDNA is linearized with a restriction enzyme, as this may not be immediately evident.

Additional detail has been added to this section of the Results to help to explain the structures observed using Southern blotting. This section now reads:

"Because of the role of TOP3A in the separation of mtDNA, loss of TOP3A activity can lead to the accumulation of hemicatenated mtDNAs, which are highly stable and remain intact through restriction digestion and electrophoresis (Nicholls et al., 2018)."

We have also carried out further characterisation of hemicatenated mtDNAs from patient muscle samples, which suggest that both hemicatenated and deletion-containing mtDNAs are visible on Southern blots. These experiments are described in detail in our response to Reviewer 2.

For Figure 3 (DNA binding assay), there are two shifted bands. What do the two different shifted bands represent, and are both considered as bound with respect to the quantification for Fig 3K?

We appreciate that this point is not explained clearly enough. In the EMSAs, the 80 nt ssDNA oligo that is used for these experiments is sufficiently long that two molecules of TOP3A can bind to a single molecule of ssDNA. This leads to a lower gel-shifted band being visible at lower protein concentrations and a second, higher gel-shifted product also being visible at higher protein concentrations.
In order to demonstrate this, we have carried out an additional EMSA using WT TOP3A together with either a 40 nt or 80 nt ssDNA oligo, side-by-side (Figure EV4B in the revised version of the manuscript). This experiment shows that a single shifted band is visible when the 40 nt oligo is used, whereas two shifted bands are visible when the longer oligo is used. For the quantifications of the EMSAs both shifted bands are quantified as bound, and so the results are not affected by the length of the oligo. The diagrams have also been updated in Figure 4.

Does TOP3A form a dimer, and if so would this impact the mechanism of disease? Would it be worth combining multiple variants in the in vitro biochemical assays to see how variants may interact? Could mutant variants decrease WT protein activity in a dominant negative way?

As a type IA topoisomerase, TOP3A acts as a monomer (aside from forming a heteromeric nuclear complex with RMI1, RMI2 and BLM), and given the recessive inheritance patterns of the variants in our patient cohort we would not necessarily expect the different variants to interact. However, we had not established this experimentally and we therefore carried out additional *in vitro* decatenation assays to investigate possible interactions between variants. An analysis of the relative decatenation activities of pathological TOP3A variants, either alone or in the combinations observed in patients that carry two compound heterozygous missense variants (Families III, V and VII) found that combinations of two variants showed an intermediate level of decatenation activity relative to the two individual variants alone (Figure 6L+M in the revised version of the manuscript). Based on these data we do not believe there to be any indication that the variants interact. These data also highlight that all combinations of variants that are found in mitochondrial disease possess one allele that retains a significant level of catalytic activity. This is in contrast to, for example, the p.Ala176Val variant found in Bloom syndrome that is observed *in trans* with a nonsense allele (Martin *et al*. (2018). *Am J Hum Genet*; **103**(2): 221-231), and is consistent with our hypothesis that the disease phenotype is determined by the combined severity of the two compound variants upon the catalytic activity of the protein. Descriptions of these experiments have also been added to the Results and Discussion sections.

In the discussion, the authors may want to speculate on some of the potential disease modifying effects, especially why the female patients seem to present earlier than male patients.

Thank you for highlighting this, although we're not sure that we have sufficient data to comment fully and include a comment in the revised manuscript. The patient cohort is too small to properly ascertain if this is a gender-related phenomenon, with the milder/later presentations more likely to reflect the severity of the TOP3A genetic defect, and its consequences in clinically relevant tissues (i.e. multiple mtDNA deletions leading to focal COX deficiency). We would note that for patients III-1 and III-2, a brother and sister, there is no noticeable difference in either the clinical phenotype or age at presentation, suggesting that sex is not playing a significant role in this pedigree at least.

Referee #2 (Comments on Novelty/Model System for Author):

Mutation discovery follows the standards in the field and is perhaps unexciting routine work for most medical genetics laboratories. The effect of the mutations have been only characterised in vitro, which, while adequate, brings only limited information about the disease mechanisms.

In the revised version of the manuscript we have carried out a number of new experiments aimed at expanding our understanding of the disease mechanisms involved, including the use of *in vivo* rescue experiments in a cell model, and experiments mixing proteins to understand how compound heterozygous variants may interact. We feel that these new experiments provide a greater insight into the pathophysiology of *TOP3A* variants. These experiments are described in our specific responses to the points raised below.

Referee #2 (Remarks for Author):

The manuscript by Erdinc et al. describes a number of new pathological variants in TOP3A, resulting in mtDNA rearrangements, COX dysfunction and mitochondrial diseases with

varying representation and age of onset. The findings are highly interesting, and the experimental work well conducted, although characterising the mutant phenotypes only in vitro brings rather limited information about the disease mechanisms. Introduction and discussion could be expanded but the overall presentation of the manuscript is well structured and almost flawless English.

More specifically,

Introduction:
L86: Maybe worth mentioning that the mitochondrial targeting sequence is translated from an alternative start-codon?

We agree that the description of TOP3A subcellular targeting could be improved, and we have added additional detail to the Introduction (see also our response to Reviewer 1, point 2). The updated text reads:

"The TOP3A transcript contains two potential initiation codons, M1 and M26. Translation from the upstream start site generates a longer isoform bearing an N-terminal mitochondrial targeting sequence (MTS) that directs its import into mitochondria, while translation from the downstream initiation codon generates an N-terminally truncated protein that lacks the MTS and is directed for nuclear import (Menger et al., 2022; Nicholls et al., 2018; Tsai et al., 2016; Wang et al., 2002; Wu et al., 2010). Proteolytic cleavage of the MTS during mitochondrial import leads to mature mitochondrial and nuclear isoforms that are almost identical at the N-terminus (Wang et al., 2002)."

L99: There seems to be a recent publication linking TOP3A to mtDNA replication progression as well. https://doi.org/10.1093/nar/gkac660

This recent paper is now referenced in the Introduction in relation to the molecular role of TOP3A, and in the Results in relation to the formation of mtDNA rearrangements.

L100: TOP3A is a Type I topoisomerase, so it cannot separate two interlocked double-stranded mtDNAs, which is basically meant by decatenation, also by definition, catenanes do not have a "junction" (L101). It seems that the authors are talking about hemicatenanes (mentioned only once in the discussion L503), which are quite different. It is mandatory that mtDNA replication, hemicatenane formation, resolution and their relevance to the experiments are properly introduced.

We have made a number of small revisions to the text aimed at improving the accuracy of the language regarding the substrates and mechanism of TOP3A.
Because the location and exact structure of the hemicatenanes formed during mtDNA replication termination in the absence of TOP3A remain quite poorly characterised, we have found it difficult to write in very specific terms about the nature and structure of this termination intermediate. However, we agree that the current text could be improved and that we could provide greater background on mtDNA replication and the role of TOP3A. We have added additional details to the Introduction in several places to better introduce mtDNA replication, replication origins, and the mode of action of TOP3A. We have also removed references to 'catenanes' when referring to mtDNA replication products. For *in vitro* experiments, we now refer to the synthesised substrates as 'ssDNA catenanes', and the mechanism of TOP3A as 'ssDNA decatenation activity' which we feel is appropriate and consistent with previous uses of the term in the literature.

Methods
L200 Please provide sequence coordinates for the probe.

The probe loci have been added as suggested to the Methods and also to the Figure Legends.

Results:
L428: Figure 3C should be 2C.

We are grateful to the reviewer to noticing this mistake, which has now been corrected.

Mark the replication origins to the mtDNA maps on Fig. 2D-G. Many of the breakpoints around 16k seem oddly precise. This should be discussed. Overexpression experiments on TOP3A seem to result in specific breakage of mtDNA around the non-coding region (https://doi.org/10.1093/nar/gkac660). Can there be a connection between a sequence specific break point and the observed rearrangements?

We have now marked the replication origins onto the mtDNA plots in Figure 2 and Figure EV2.
The specific breakpoint that is prominent in the plots for Pa3-1 and Pa5-1 is located around nt.16,070 at the termination associated sequence (TAS) at the 3' end of the mtDNA D-loop. This breakpoint is frequently observed in cases of adult-onset mitochondrial disease related to variants in mtDNA replication factors (e.g. Zeviani *et al*. (1989). *Nature*; **339**(6222): 309-311; Persson *et al*. (2019). *Nat Commun*; **10**(1): 759), as well as following artificially induced breaks in mtDNA (e.g. Bacman *et al*. (2009). *Nucleic Acids Res*; **37**(13): 4218-26).
The exact mechanism of deletion formation at this site has not been resolved, although may relate to this site being particularly accessible for rearrangements owing to the triple-stranded structure of the D-loop. As this breakpoint is seen in other mtDNA replication disorders we do not believe the locus to be specifically related to TOP3A, but rather indicative of impaired mtDNA replication more broadly. We have added a specific description of this breakpoint to the Results section.
Of the other observed rearrangements, the small duplications that are visible in mtDNA plots from Pa2 are of interest as they are positioned very close to the location of hemicatenane formation following TOP3A depletion (Nicholls *et al*. (2018). *Mol Cell*; **69**(1): 9-23) and to mtDNA breakage following overexpression of TOP3A (Hangas *et al*. (2022). *Nucleic Acids Res*; **50**(15): 8733-8748). However, we are hesitant to specifically ascribe this rearrangement to the site of TOP3A activity as it has also been observed before in a small number of patients not known to have impaired TOP3A activity. This is currently mentioned in the Results (lines 449-458) and in the Discussion (lines 578-584).

Fig 2H and I: As the DNA has been digested, there should not be any catenated DNA left. What are the sizes of "non-16.6 kb" fragments in P2 and P5 samples?

The hemicatenanes formed when TOP3A activity is impaired are very stable, and will remain bound upon restriction digestion and electrophoresis. These appear as high molecular weight smears on Southern blots, and we have used this property in the past for mapping the site of TOP3A activity. However, it can be difficult to distinguish hemicatenated mtDNAs from mtDNA rearrangements, and we have carried out control experiments to better characterise these products. These are described in our response to the point below beginning "L447".

L441 "interlinked mtDNAs". Related to comment for L100, so these are not catenanes, but I assume hemicatenanes are meant?

We agree, and this has been changed to 'hemicatenated'.

L447 "unresolved catenated mtDNA replication products". Catenanes should fall apart when the DNA is linearized. The size of the additional bands in P2 and P5 sample is different

depending on the used restriction enzyme, indicating that the molecule is a partial duplication or then some non-linear species with a specific branch point region or a surprisingly stabile hemicatenane, which is different between the patients. This is an interesting observation and should be pursued further. To me, the fact that the two patients have different additional bands strongly argues against simple "unresolved replication products". I suggest digesting the sample with different restriction enzymes yielding smaller fragment around the expected duplicated region to map it more carefully. However, judging from the sequencing results of the same sample, it can well be a junctional molecule of some type. For the scope of the work, it would be sufficient to differentiate between mtDNA sequence rearrangement (partial duplication) and persistent molecular structure, using e.g. two-dimensional gels.

We have carried out additional experiments in order to better characterise the species visible on these blots. As mentioned above, the hemicatenanes that are formed when TOP3A activity is impaired in cultured cells are stable and can remain bound upon restriction digestion and electrophoresis. We believe that the smears of high molecular weight products that can especially be seen in muscle mtDNA from Pa5-1 represent such unresolved hemicatenanes. However, we believe that the additional discrete bands that the reviewer notes represent deletion-containing molecules. In muscle DNA from Pa5-1, because the majority of deletions occur in the major arc, and many have similar breakpoints, these deletion-containing mtDNAs run as smaller molecules when cleaved in the minor arc with PvuII. However, because most of these deletions remove the BamHI restriction site in the major arc at nt. 14,258, these deletion-containing molecules remain uncut and circular, and run above the linear mtDNA. Conversely, muscle mtDNA from Pa2 contains a high proportion of minor arc deletions, suggesting that the faster-migrating band in BamHI-treated samples represents cleaved deletion-containing molecules, and the slower-migrating band in the PvuII-treated samples represents uncut circular deletion-containing molecules.
The quantity and quality of patient muscle DNA available unfortunately precludes analysis using 2D gels. However, we have treated muscle DNA samples from Pa2 and Pa5-1 with T7 endonuclease I, which cuts branched DNA molecules (including the hemicatenanes formed upon loss of TOP3A activity) and visualised the products using Southern blotting (Figure EV2C in the revised version of the manuscript). This treatment eliminates the high molecular weight smears, consistent with these representing hemicatenated mtDNAs, but leaves the discrete bands largely intact, consistent with these representing deletion-containing mtDNAs.
This section in the revised version of the manuscript now reads:

*"Samples from Pa2 and (particularly) Pa5-1 showed the presence of high molecular weight smears consistent with hemicatenated mtDNA replication products, as well as discrete bands that migrated either faster or slower than full-length mtDNA depending upon the restriction enzyme used (**Figure 3A-B**). We further characterised these species from Pa2 and Pa5-1 by treatment with T7 endonuclease I (**Figure EV3C**), which cleaves hemicatenaned mtDNA replication products (Nicholls et al., 2018). This treatment eliminated the high molecular weight smears, consistent with these representing branched hemicatetenated molecules, but not the discrete bands, suggesting that these represent deletion-containing molecules that remove the minor arc PvuII restriction site (in samples from Pa2) or the major arc BamHI restriction site (in samples from Pa5-1)."*

Figure 4: I am astonished that the disease mutations are all basically catalytically inactive in relaxing DNA, which basically requires only nicking action. As the patients are compound heterozygous, does the combination rescue any activity? For example, would you expect the performance of 1:1 mixture of Met100Val+Met575Val to be better than one mutant alone?

We agree that the apparent lack of activity of most of the patient variants of TOP3A in the relaxation assays appears surprising, however, we believe that this can be accounted for by

the mechanism of action of the protein, and by functional redundancy in the removal of supercoiling *in vivo*.

The ssDNA catenane substrates that we have constructed to monitor decatenation activity by TOP3A (Figure 6) require only a single round of the catalytic cycle of the enzyme in order to decatenate, whereas the relaxation reactions require multiple rounds of strand-passage to be carried out in a processive manner. In our experiments, this would indicate that the processivity of the enzyme is strongly affected by the observed variants, but that these variants retain the limited capacity to undergo single rounds of their catalytic cycle in order to separate the Lk1 ssDNA catenanes used in Figure 6. The fact that TOP3A is inefficient at relaxing negatively supercoiled DNA may reflect its predominant cellular function in decatenation rather than DNA relaxation. Please note that the relaxation activity of TOP3A could also be compensated for by the type IB topoisomerase TOP1MT, which can also remove negative supercoiling *in vivo*. This would mean that the relaxation activity (but not the decatenation activity) of TOP3A is largely dispensable (see our response to the point beginning "L587" below).

We have added text to the Results and Discussion to specifically address this point.

However, we also fully agree that being able to better measure the relaxation activity of these variants would make it easier to quantitatively assess the impact of pathological variants.

In response to the reviewer's comments, and in order to try to better measure the activity of TOP3A in these assays, we have carried out further experiments:

(i) Combinations of variants. In order to determine whether there are interactions between compound heterozygous variants we have performed decatenation assays using 1:1 mixtures of variants. We chose to use decatenation assays for these experiments because we are most easily able to quantitatively assay enzyme activity using this method. We find that the mixtures of compound heterozygous variants have an intermediate level of activity compared to the individual variants alone (Figure 6L-M in the revised version of the manuscript). Based on these experiments we do not believe there is any evidence that the variants interact, consistent with their recessive inheritance. A description of these experiments has been added to the Results section, and text has been added to the Discussion to highlight the importance of considering the combined impact of both heterozygous variants.

(ii) Temperature. Previous studies of *Drosophila* TOP3 have found the enzyme to have increased relaxation activity at elevated temperatures (Chen, CF and Brill, SJ (2007). *J Biol Chem*; **282**(39): 28971-28979). We have assayed the relaxation activity of WT human TOP3A at a range of temperatures but did not see a significant increase in relaxation activity. We have chosen not to include these negative results in the manuscript, but they are shown as Figure 1A-B below for the reviewer to see.

(iii) Nicking-closing activity. As our relaxation reactions using WT TOP3A generate a significant fraction of nicked substrate, we sought to allow the protein to complete to ligation of these open complexes, which may simplify the analysis of relaxation activity. The addition of a high concentration of salt for a short period at the end of relaxation reactions has previously been shown to facilitate re-ligation for type IA topoisomerases (Cejka *et al*. (2012). *Mol Cell*; **47**(6): 886-96). However, we did not find that this step made an appreciable difference to these reactions and so we have chosen not to include them in the manuscript. These experiments are shown below as Figure 1C-F.

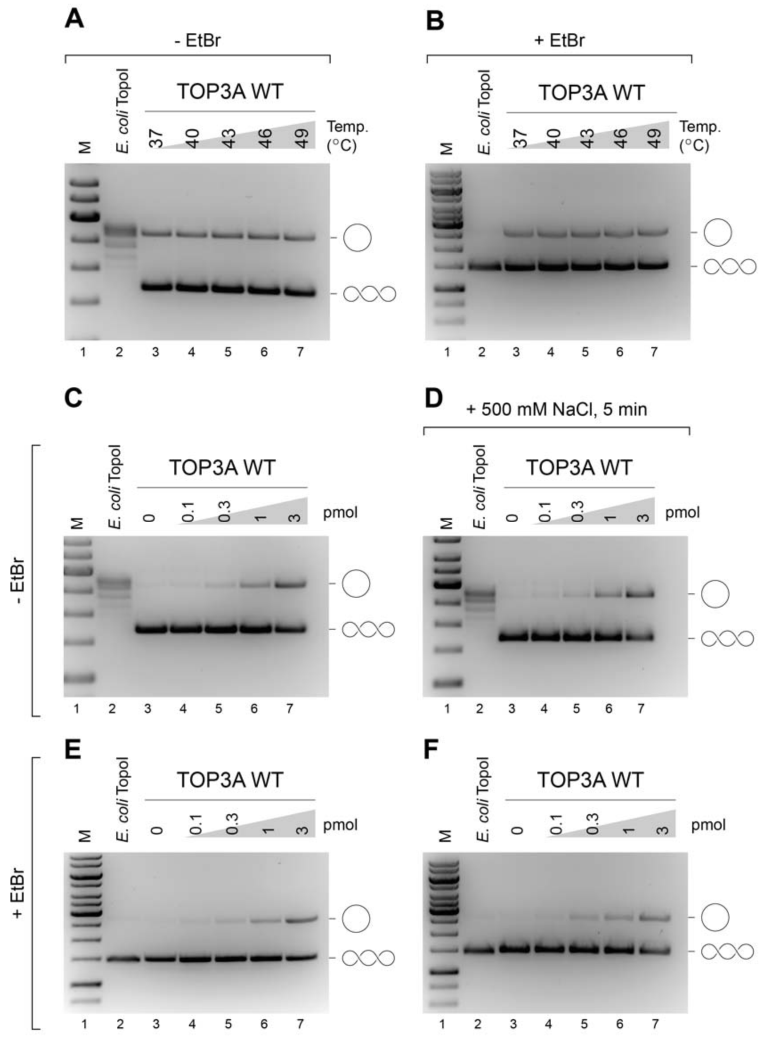

Figure 1 - Additional TOP3A relaxation reactions. (A-B) *TOP3A relaxation reactions using elevated temperatures. Supercoiled pUC19 plasmid (250 ng) was incubated with 1 pmol of WT TOP3A at the indicated temperature for 30 min. Reaction products were separated on 0.8% agarose TAE gels, either without ethidium bromide before post-staining* ***(A)*** *or in a gel containing ethidium bromide* ***(B)****. 'M' indicates marker.* ***(C-F)*** *Promotion of TOP3A re-ligation of nicked templates. Supercoiled pUC19 plasmid (250 ng) was incubated with the indicated quantity of WT TOP3A at 37℃ for 30 min. Samples were either further untreated* ***(C+E)*** *or incubated for a further 5 min with 50 mM NaCl before being deproteinised with SDS and proteinase K* ***(D+F)****. Reactions were separated on 0.8% agarose TAE gels, either without* ***(C+D)*** *or with ethidium bromide* ***(E+F)****.*

L558: Do the authors suggest that the TOP3A mutations cause replication stalling, which results in mtDNA rearrangements? Please elaborate and discuss in more detail the proposed disease mechanism.

Yes. We have previously found that the loss of mitochondrial TOP3A activity in cultured cells causes DNA replication stalling throughout the mtDNA, which we assume to result from the difficulty of replicating topologically-constrained hemicatenated templates. Given the observed mtDNA instability in patient muscle, we believe that pathological *TOP3A* variants will cause a similar impairment of mtDNA replication *in vivo*. The fact that the pattern of mtDNA rearrangements in our *TOP3A* patient is broadly similar to that observed in other autosomal disorders of mtDNA replication suggests that there is a common mechanism for

deletion formation in these disorders. We have added the following text to the Discussion to better describe this model:

"This pattern of breakpoints is comparable to that observed with other autosomal disorders of mtDNA maintenance (Zeviani et al., 1989), and consistent with deletion formation via copy-choice recombination (Persson et al., 2019). According to this model, replication stalling promotes the slippage of the template during mtDNA synthesis, leading to the generation of deletion-containing mtDNA molecules. The loss of mitochondrial TOP3A activity has been found to result in DNA replication stalling throughout the mtDNA, presumably resulting from the reduced efficiency of replicating through topologically-constrained mtDNA molecules (Hangas et al., 2022; Menger et al., 2022) and so would be expected to promote deletion formation according to this model."

Furthermore, as we recognise the limitations of using only *in vitro* assays to characterise the impact of pathological TOP3A variants upon enzyme function, we have additionally carried out cellular rescue experiments using these variants. We created cell lines that inducibly express siRNA-resistant forms of either wild-type TOP3A or individual pathological variants. We then depleted cells of endogenous TOP3A using siRNA and assessed the ability of TOP3A variants to rescue the loss of mtDNA copy number and changes to mtDNA topology (Figure 3C-F in the revised version of the manuscript). The inability of many of these variants to rescue changes to mtDNA topology and copy number supports the concept that defective mtDNA replication and topology contributes to the pathophysiology of *TOP3A* variants.
We have added descriptions of these new experiments to the Results and Discussion, together with an evaluation of the relative strengths and imitations of the different approaches that we have used to assay the effect of pathological TOP3A variants upon enzyme function.

L567: Not only the patients survive, but the mutations result in rather late onset disease symptoms (the youngest also at their teens, Table 2). Considering how poorly the disease variants perform in the in vitro assays, I think this invites more discussion. It might be also good to point out that many mtDNA maintenance diseases show tissue specificity and varying age of onset depending on the mutations.

We agree that many variants show little *in vitro* activity in the context of the disease phenotypes. However, we also note that it is not uncommon for pathological variants in core mtDNA replication and expression factors to be compatible with life despite showing little or no detectable activity *in vitro* (e.g. Olahova *et al*. (2021). *Nat Commun*; **12**: 1135, Chan *et al*. (2005). *J Biol Chem*; **280**(36): 31341-46).
After carrying out cellular rescue experiments (described in our response to the previous point) we have found that some pathological variants, particularly Leu37Val, perform better at rescuing mtDNA phenotypes in cells than their level of activity *in vitro* would have suggested. While this may indicate that this variant is (for example) more stable *in vivo*, and that its activity is underestimated by the *in vitro* assays, we are hesitant to draw strong conclusions because of the difficulty in directly comparing the activity of the endogenous protein to that of an overexpressed hypomorphic variant.

The results of these experiments are now described in the Results section in the revised manuscript. We have also added text to the Discussion. This text now reads:

"Interestingly some variants, particularly Leu37Val, were capable of significantly rescuing mtDNA copy number loss induced by siRNA depletion, despite possessing little activity in vitro. These differences may be accounted for by a greater stability of the protein in vivo, or by differences in expression level between the endogenous and inducibly expressed proteins."

We have amended the Introduction to better describe the tissue specificity and age of onset of mitochondrial disorders, which now reads:

"Clinically, these mtDNA maintenance disorders often exhibit a cluster of clinical features and ages of onset, from severe and progressive neurological disorders in childhood such as Alpers syndrome and Leigh syndrome, to milder adult-onset disease such as progressive external ophthalmoplegia (PEO), depending upon the severity and location of the variant (Gorman et al., 2016)."

L587: Interestingly all of the mutants are really poor in relaxing supercoils and Met575Val seems also very impaired in decatenation, although it is associated with the late onset disease. Based on the results, the de-hemicatenation seems more function important than unwinding supercoils? Can you discuss more the functional importance of the different activities of TOP3A based on your findings?

This is an interesting question. As the reviewer notes, the activity of the p.Met575Val variant is significantly impaired *in vitro*, despite being found *in trans* with loss-of-function alleles in adult-onset disease (Families II and VI).
The type IB topoisomerase TOP1MT also localises to human mitochondria (Zhang H *et al*. (2001). *PNAS*; **98**(19): 10608-13), and so is presumably capable of providing functional redundancy with TOP3A in the relaxation of negative supercoiling. Our recent work has indicated that TOP3A and TOP1MT are the only topoisomerases that localise to mitochondria in human cells (Menger K *et al*. (2022). *Nucleic Acids Res*; **50**(19): 11154-74). This would mean that, while TOP1MT would be able to provide relaxation activity when TOP3A activity is impaired, TOP3A would be the only mitochondrial topoisomerase with the capacity to separate hemicatenated mtDNA replication products. This highlights the importance of maintaining TOP3A ssDNA decatenation activity for mtDNA replication. We have added the following text to the manuscript to discuss this point further:

"All pathological variants showed significantly impaired relaxation activity in vitro compared to the wild-type protein. However, we note that the type IB topoisomerase TOP1MT, which localises to human mitochondria (Zhang et al., 2001), is also capable of relieving supercoiling and could compensate for the lack of TOP3A activity in vivo. In contrast, TOP3A is the only mitochondrial topoisomerase that possesses ssDNA decatenation activity (Menger et al., 2022), highlighting the importance of maintaining this activity for mtDNA replication."

Referee #3 (Remarks for Author):

This manuscript reported eleven individuals from nine families with adult-onset mitochondrial disease caused by compound heterozygous mutations in TOP3A gene. Through sequential COX-SDH histochemistry and quadruple immunofluorescence, the presence of mitochondrial myopathy was confirmed in patients with TOP3A mutations. Structural analysis of the mtDNA revealed the mtDNA instability in TOP3A patient muscle. Various methods were used to investigate the function of the TOP3A variants, confirming that pathological TOP3A variants cause a decrease in its activity of DNA binding, relaxation capacity, and decatenation. Overall, the paper confirms that TOP3A is a causative gene for adult mitochondrial diseases and try to provide a better understanding on TOP3A.

The clinical presentation, modelling, and muscle mitochondrial characterization from patients are quite good. I am worried about model proposed based on the biochemical characterization of TOP3A variants.

1. The content of abstract is not consistent with the title. The tile is "Pathological variants in

TOP3A cause distinct disorders of mitochondrial and nuclear genome stability". However, the core results of biochemical characterization of TOP3A variants were not summarized in the abstract.

In response to the comments of the Reviewers we have significantly revised the abstract, which now places a greater emphasis on the characterisation of TOP3A variants and the proposed model for how different variants lead to differing clinical phenotypes. We hope that the reviewer will agree that the abstract has been significantly improved by these changes.

2. The model proposed by this article from the results was not quite reasonable. Take the DNA binding activity as an example, leu37Val and Met575Val variants of TOP3A from mitochondrial disease patients had significant loss of DNA binding activity, while Ala176Val and Ser810* of TOP3A variants from Bloom-like disease patients had comparable level of DNA binding activity to the WT protein. The model is that moderate loss of enzyme activity results in mitochondrial disease in adults and the severe loss of enzyme activity results in Bloom syndrome-like disorder. The model is the opposite of the results.

We appreciate that, in the original manuscript, the model for how different *TOP3A* variants result in different disease phenotypes was not presented clearly enough. In the revised version of the manuscript, we have performed a number of additional experiments intended to expand our understanding of the disease mechanism, and made a number of alterations to the text in order to better describe the model. These changes have addressed the following main issues:

(i) Testing for interactions between compound heterozygous variants.
Our model proposes that the disease phenotype depends upon the degree of loss of TOP3A catalytic activity, but we had not assessed the activity level of combinations of variants found in compound heterozygous patients. In the revised version of the manuscript we have carried out ssDNA decatenation assays using equimolar mixtures of these variants, which are presented as Figure 6L+M in the new manuscript. These data highlight that TOP3A variants that show very limited activity and are found in mitochondrial disease are typically found *in trans* with a variant that retains a significant level of activity. For instance, while both the Arg558Trp and Met575Val variants possess little activity *in vitro*, both are found in trans with the Met100Val, which retains a significant level of activity. The combination of both variants together results in an intermediate level of activity. In contrast, the Bloom syndrome-associated p.Ala176Val variant, which also possesses very little *in vitro* activity, is found *in trans* with a loss-of-function truncating variant, suggesting that the overall TOP3A activity level will be lower. We have also added text to the Results and Discussion sections to describe these results, and to highlight the importance of considering variants in the combinations that they are observed in patients.

(ii) Rescue experiments in a cultured cell model of TOP3A deficiency.
We recognise that the *in vitro* characterisation of pathological TOP3A variants, while allowing a detailed dissection of different aspects of enzyme function, may not necessarily correspond faithfully to the *in vivo* enzymatic defects. We have therefore carried out additional rescue experiments using a cellular siRNA model of TOP3A deficiency. We created U2OS cell lines stably and inducibly expressing siRNA-resistant forms of TOP3A, either in wild-type form or carrying one of the pathological variants that we have studied in this manuscript. We then depleted cells of TOP3A using siRNA, and induced the expression of individual TOP3A variants in order to assess the ability of these variants to rescue the mtDNA phenotypes associated with TOP3A deficiency. These experiments are Figure 3C-F in the revised version of the manuscript. Treatment with TOP3A siRNA results in an approximately 75% loss of mtDNA copy number (determined using qPCR), which can be rescued by expression of WT TOP3A or by some TOP3A variants (Leu37Val, Ala95Val and Met100Val), or either not rescued or partially rescued by the expression of other variants.

We additionally assess the degree of mtDNA hemicatenation and mtDNA topology using Southern blotting, and find that the loss of TOP3A causes the loss of monomeric mtDNA forms. This can again be rescued by expression of WT TOP3A and some, but not all, pathological TOP3A variants. The ability of variants to rescue mtDNA phenotypes in this cell model broadly corresponds to the observed level of activity *in vitro*, although we note that some variants perform better in cellular experiments than *in vitro*, notably the Leu37Val variant. We have added descriptions of these experiments to the Results, Discussion, and Methods sections, as well as a critical evaluation of the relative strengths and weaknesses of these different approaches to determining enzymatic defects and their impact upon mtDNA structure and replication.

(iii) Improving descriptions of how different *in vitro* assays relate to different aspects of the protein function.
TOP3A must first bind to its DNA substrate, cleave it, undergo strand passage, and then reseal the DNA break. DNA binding is therefore necessary but not sufficient for catalytic activity, and a variant could be capable of binding, but not cleaving, a DNA substrate. This appears to be the case with the p.Ala176Val variant associated with Bloom syndrome, which exhibits a wild-type level of DNA binding activity but very little ssDNA decatenation or relaxation activity. This phenotype is also observed with the catalytically-inactive Y362F protein, which results from mutation of the catalytic tyrosine residue. In the case of the p.Ser810* variant, although this variant possesses ssDNA decatenation activity in our assays, it was undetectable in cells from Bloom syndrome patients (Martin *et al*. (2018). *Am J Hum Genet*; **103**(2): 221-231). It was proposed that the low level of TOP3A activity in these cells is therefore the result of protein instability rather than a direct effect upon the catalytic activity of the protein. Our finding that this variant is inefficient at rescuing mtDNA copy number depletion and mtDNA hemicatenation resulting from depletion of TOP3A in cells (Figure 3) is also consistent with this model.
We appreciate that these points were not made clearly enough in the manuscript, and we have made several changes to the text in order to clarify the results of different assays.
The following text has also been added to the Discussion:

"We find that different variants affect different aspects of the enzymatic activity, with p.Leu37Val and p.Met575Val impairing the DNA binding activity of the enzyme, which is a prerequisite for its catalytic activity. In contrast, the p.Ala176Val variant associated with Bloom syndrome exhibits a wild-type level of DNA binding activity but is greatly impaired in its decatenation activity."

28th Feb 2023

Dear Dr. Nicholls,

Thank you for the submission of your revised manuscript to EMBO Molecular Medicine. I am pleased to inform you that we will be able to accept your manuscript pending the following final amendments:

1) Please address the referee #2 minor points.
2) Authors: E-mail correspondence to Maria Falkenberg could not be delivered. Please update her e-mail addresses and make sure to enter correct e-mail addresses for all authors in our submission system.
3) In the main manuscript file, please do the following:
- Correct/answer the track changes suggested by our data editors by working from the attached document.
- Add up to 5 keywords.
- Add callouts for Figure 4D-H, J and 6D-H, J-K.
- In M&M, please include statement that in addition to WMA Declaration of Helsinki the experiments also conformed the Department of Health and Human Services Belmont Report.
- In M&M, provide the antibody dilutions that were used for each antibody.
- In M&M, add statistical paragraph that should reflect all information that you have filled in the Authors Checklist, especially regarding randomization, blinding, replication etc.
- Author contributions: Please remove it from the manuscript and specify author contributions in our submission system. CRediT has replaced the traditional author contributions section because it offers a systematic machine-readable author contributions format that allows for more effective research assessment. You are encouraged to use the free text boxes beneath each contributing author's name to add specific details on the author's contribution. More information is available in our guide to authors:
https://www.embopress.org/page/journal/17574684/authorguide#authorshipguidelines
- In data availability section please remove information about source data and data generated in other studies. Genomics England 100,000 Genomes Project should be cited at an appropriate place in M&M. Data availability section should contain information only for the raw data generated in this study and deposited in public databases. Since we have here a case of patient data, the journal encourages authors to provide access to genotype and clinical data with as few restrictions as possible while respecting ethical obligations to the patients and relevant medical and legal issues. If compatible with the individual consent agreement used in the study, such data should be deposited in one of the major public access-controlled repositories. If there are, however, consent agreement restrictions or any other ethical obligations preventing publication of patient data these should be declared in the "Data availability" section. For example: Due to the consent agreement restrictions, patient WGS data could not be made freely available and are therefore not deposited in a public database.

If you deposit patient WGS raw data, please use the following format to report the accession number of your data:

The datasets produced in this study are available in the following databases:
[data type]: [full name of the resource] [accession number/identifier] ([doi or URL or identifiers.org/DATABASE:ACCESSION])

Please check "Author Guidelines" for more information.
https://www.embopress.org/page/journal/17574684/authorguide#availabilityofpublishedmaterial
4) Appendix: Please add page numbers, also in the table of content.
5) Synopsis: Every published paper now includes a 'Synopsis' to further enhance discoverability. Synopses are displayed on the journal webpage and are freely accessible to all readers. They include separate synopsis image and synopsis text.
- Synopsis image: Please adjust the visual abstract and upload it as a high-resolution jpeg file 550 px-wide x (250-400)-px high.
- Synopsis text: Please provide a short standfirst (maximum of 300 characters, including space) as well as 2-5 one sentence bullet points that summarise the paper as a .doc file. Please write the bullet points to summarise the key NEW findings. They should be designed to be complementary to the abstract - i.e. not repeat the same text. We encourage inclusion of key acronyms and quantitative information (maximum of 30 words / bullet point). Please use the passive voice.
- Please check your synopsis text and image before submission with your revised manuscript. Please be aware that in the proof stage minor corrections only are allowed (e.g., typos).
6) For more information: This space should be used to list relevant web links for further consultation by our readers. Could you identify some relevant ones and provide such information as well? Some examples are patient associations, relevant databases, OMIM/proteins/genes links, author's websites, etc...
7) As part of the EMBO Publications transparent editorial process initiative (see our Editorial at http://embomolmed.embopress.org/content/2/9/329), EMBO Molecular Medicine will publish online a Review Process File (RPF) to accompany accepted manuscripts. This file will be published in conjunction with your paper and will include the anonymous referee reports, your point-by-point response and all pertinent correspondence relating to the manuscript. Let us know whether you agree with the publication of the RPF and as here, if you want to remove or not any figures from it prior to publication. Please note that the Authors checklist will be published at the end of the RPF.
8) Please provide a point-by-point letter INCLUDING my comments as well as the reviewer's reports and your detailed

responses (as Word file).

I look forward to reading a new revised version of your manuscript as soon as possible.

Yours sincerely,

Zeljko Durdevic

Zeljko Durdevic
Editor
EMBO Molecular Medicine

***** Reviewer's comments *****

Referee #2 (Comments on Novelty/Model System for Author):

The presented manuscript is a detailed study of disease-associated mutations in TOP3A, utilising state-of-the-art sequencing methods for mutation identification and proper combination of in vitro and in vivo experimental approaches to interrogate the pathological consequences of these mutations. I've marked the medical impact as medium, as while the results help to understand and diagnose TOP3A diseases, these are nevertheless rare disorders and there are no presented treatment options to influence their clinical outcome.

Referee #2 (Remarks for Author):

The revised manuscript by Erdinc et al. has developed to a nice and coherent story. The additional experiment performed as requested and overall, the data are well presented. I believe that this is a valuable contribution to the field, giving interesting insight into the physiological roles of human mitochondrial topoisomerases and their pathologies. The presented manuscript is therefore well suited to be published in EMBO Molecular Medicine. I have only few minor suggestions:

L109: Consider adding a bit of detail for the later story, for example: "TOP3A is a _type I_ topoisomerase; an enzyme that alters the topology of DNA by creating temporary _single-strand_ breaks that [...] (Voss et al., 2011). _In contrast type II topoisomerases, such as TOP2, catalyze double-strand breaks, required for passage of DNA strands during (de)catenation." Apologies for missing this in the first review round.

L191-L210: The MinION sequencing of such samples has been quite brave, and as noted the result difficult to achieve. Despite the improvements in the basecalling, nanopore sequencing is still very prone to missing or miss-calling nucleotides. This might be an issue, especially with such a poor coverage over the region of interest. Easier option would have been to sequence one of the parents, but this was probably not possible? While this analysis was not suggested by me, I am more convinced by the data presented later in the paper that these cases represent compound heterozygosity. Cis arrangement of the variants would have also meant that the disease is dominant and a gain-of-function phenotype for the combination is difficult to imagine based on the data on individual mutations presented in this paper.

L349 "while expressing the TOP3A cDNA" > consider rewording to "while expressing the _inducible TOP3A construct_". The long sentence starting from L347 could be modified so that the inducibly/inducible is not repeated.

L355-7: A very nice result.

L496: Is "copy choice recombination" completely excluded as a mechanism of double-strand break repair in mitochondria? Replication stalling does induce double-strand breaks in other systems and, as pointed out in the introduction, TOP3A expression can cause mtDNA breakage at the non-coding region.In nucleus (and with prokaryote DNA), replication-mediated recombination is an important double-strand break repair mechanism, which is independent of recombinases, such as Rad51 (aka Rad51-independent, break-induced replication).

Overall, great work.

Referee #3 (Remarks for Author):

Is suitable for publication.

***** Reviewer's comments *****

Referee #2 (Comments on Novelty/Model System for Author):

The presented manuscript is a detailed study of disease-associated mutations in TOP3A, utilising state-of-the-art sequencing methods for mutation identification and proper combination of in vitro and in vivo experimental approaches to interrogate the pathological consequences of these mutations. I've marked the medical impact as medium, as while the results help to understand and diagnose TOP3A diseases, these are nevertheless rare disorders and there are no presented treatment options to influence their clinical outcome.

Referee #2 (Remarks for Author):

The revised manuscript by Erdinc et al. has developed to a nice and coherent story. The additional experiment performed as requested and overall, the data are well presented. I believe that this is a valuable contribution to the field, giving interesting insight into the physiological roles of human mitochondrial topoisomerases and their pathologies. The presented manuscript is therefore well suited to be published in EMBO Molecular Medicine. I have only few minor suggestions:

L109: Consider adding a bit of detail for the later story, for example: "TOP3A is a _type I_ topoisomerase; an enzyme that alters the topology of DNA by creating temporary _single-strand_ breaks that [...] (Voss et al., 2011). _In contrast type II topoisomerases, such as TOP2, catalyze double-strand breaks, required for passage of DNA strands during (de)catenation." Apologies for missing this in the first review round.

The following text has been added to this point in the Introduction:

"*Type I topoisomerases, such as TOP3A, break one strand of DNA thereby allowing the relaxation of negatively supercoiled DNA as well as the separation of hemicatenated molecules. In contrast, type II topoisomerases such as TOP2 create double-strand breaks, required for the catenation or decatenation of dsDNA molecules.*"

L191-L210: The MinION sequencing of such samples has been quite brave, and as noted the result difficult to achieve. Despite the improvements in the basecalling, nanopore sequencing is still very prone to missing or miss-calling nucleotides. This might be an issue, especially with such a poor coverage over the region of interest. Easier option would have been to sequence one of the parents, but this was probably not possible? While this analysis was not suggested by me, I am more convinced by the data presented later in the paper that these cases represent compound heterozygosity. Cis arrangement of the variants would have also meant that the disease is dominant and a gain-of-function phenotype for the combination is difficult to imagine based on the data on individual mutations presented in this paper.

To establish phase, we agree that the straightforward approach is the sequencing of parental samples, and this has been carried out where possible. In cases that we have used MinION sequencing, however, such samples were not available. We also agree that the accuracy of nanopore sequencing is lower than that of most short-read chemistries. Nevertheless, as our scenario required us to identify reference/non-reference bases at loci for which the variant genotypes had been previously established, we anticipated that the cumulative read-depth would be lower than is typically required for variant discovery. While increased read depths are always welcomed, the observed read sequences are consistent with the variants being arranged in *trans*. As the reviewer indicates, the *cis* arrangement of our reported variants is also unlikely given the presented functional data.

L349 "while expressing the TOP3A cDNA" > consider rewording to "while expressing the

inducible TOP3A construct". The long sentence starting from L347 could be modified so that the inducibly/inducible is not repeated.

This sentence has been changed as suggested.

L355-7: A very nice result.

L496: Is "copy choice recombination" completely excluded as a mechanism of double-strand break repair in mitochondria? Replication stalling does induce double-strand breaks in other systems and, as pointed out in the introduction, TOP3A expression can cause mtDNA breakage at the non-coding region.In nucleus (and with prokaryote DNA), replication-mediated recombination is an important double-strand break repair mechanism, which is independent of recombinases, such as Rad51 (aka Rad51-independent, break-induced replication).

High levels of replication stalling may be expected to lead to the accumulation of broken mtDNA molecules, although previous data has suggested that linear mtDNA molecules are rapidly degraded rather than being used for DSB repair. We have referenced the copy-choice recombination model in relation to deletion formation as the pattern of deletions in TOP3A patient muscle appears consistent with this. We agree that the small duplications seen in Pa2 may relate to TOP3A activity in the NCR, although as these species are only detected in one patient we would be hesitant to draw any strong conclusions from this.
Assuming that break-induced replication would still require resection activity, it is not clear which enzyme present in the mitochondrial matrix would provide this activity. Such a mechanism seems formally possible although would require further work to provide evidence for it, and we feel would sit significantly outside of the scope of the current work.

Overall, great work.

Referee #3 (Remarks for Author):

Is suitable for publication.

2nd Revision - Editorial Decision 14th Mar 2023

We are pleased to inform you that your manuscript is accepted for publication and is now being sent to our publisher to be included in the next available issue of EMBO Molecular Medicine.

EMBO Press Author Checklist

Corresponding Author Name: Thomas J. Nicholls	
Journal Submitted to: EMBO Molecular Medicine	
Manuscript Number: EMM-2022-16775	

USEFUL LINKS FOR COMPLETING THIS FORM
The EMBO Journal - Author Guidelines
EMBO Reports - Author Guidelines
Molecular Systems Biology - Author Guidelines
EMBO Molecular Medicine - Author Guidelines

Reporting Checklist for Life Science Articles (updated January 2022)
This checklist is adapted from Materials Design Analysis Reporting (MDAR) Checklist for Authors. MDAR establishes a minimum set of requirements in transparent reporting in the life sciences (see Statement of Task: 10.31222/osf.io/9sm4x). Please follow the journal's guidelines in preparing your manuscript.
Please note that a copy of this checklist will be published alongside your article.

Abridged guidelines for figures
1. Data
The data shown in figures should satisfy the following conditions:
- the data were obtained and processed according to the field's best practice and are presented to reflect the results of the experiments in an accurate and unbiased manner.
- ideally, figure panels should include only measurements that are directly comparable to each other and obtained with the same assay.
- plots include clearly labeled error bars for independent experiments and sample sizes. Unless justified, error bars should not be shown for technical replicates.
- if n<5, the individual data points from each experiment should be plotted. Any statistical test employed should be justified.
- Source Data should be included to report the data underlying figures according to the guidelines set out in the authorship guidelines on Data Presentation.

2. Captions
Each figure caption should contain the following information, for each panel where they are relevant:
- a specification of the experimental system investigated (eg cell line, species name).
- the assay(s) and method(s) used to carry out the reported observations and measurements.
- an explicit mention of the biological and chemical entity(ies) that are being measured.
- an explicit mention of the biological and chemical entity(ies) that are altered/varied/perturbed in a controlled manner.
- the exact sample size (n) for each experimental group/condition, given as a number, not a range;
- a description of the sample collection allowing the reader to understand whether the samples represent technical or biological replicates (including how many animals, litters, cultures, etc.).
- a statement of how many times the experiment shown was independently replicated in the laboratory.
- definitions of statistical methods and measures:
  - common tests, such as t-test (please specify whether paired vs. unpaired), simple χ2 tests, Wilcoxon and Mann-Whitney tests, can be unambiguously identified by name only, but more complex techniques should be described in the methods section;
  - are tests one-sided or two-sided?
  - are there adjustments for multiple comparisons?
  - exact statistical test results, e.g., P values = x but not P values < x;
  - definition of 'center values' as median or average;
  - definition of error bars as s.d. or s.e.m.

Please complete ALL of the questions below.
Select "Not Applicable" only when the requested information is not relevant for your study.

Materials

Newly Created Materials	Information included in the manuscript?	In which section is the information available? (Reagents and Tools Table, Materials and Methods, Figures, Data Availability Section)
New materials and reagents need to be available; do any restrictions apply?	Not Applicable	

Antibodies	Information included in the manuscript?	In which section is the information available? (Reagents and Tools Table, Materials and Methods, Figures, Data Availability Section)
For **antibodies** provide the following information:<br>- Commercial antibodies: RRID (if possible) or supplier name, catalogue number and or/clone number<br>- Non-commercial: RRID or citation	Yes	Materials and Methods

DNA and RNA sequences	Information included in the manuscript?	In which section is the information available? (Reagents and Tools Table, Materials and Methods, Figures, Data Availability Section)
**Short novel DNA or RNA including primers, probes**: provide the sequences.	Yes	Appendix Table S1

Cell materials	Information included in the manuscript?	In which section is the information available? (Reagents and Tools Table, Materials and Methods, Figures, Data Availability Section)
**Cell lines:** Provide species information, strain. Provide accession number in repository **OR** supplier name, catalog number, clone number, and/**OR** RRID.	Yes	Materials and Methods
**Primary cultures:** Provide species, strain, sex of origin, genetic modification status.	Not Applicable	
Report if the cell lines were recently **authenticated** (e.g., by STR profiling) and tested for mycoplasma contamination.	Yes	Materials and Methods

Experimental animals	Information included in the manuscript?	In which section is the information available? (Reagents and Tools Table, Materials and Methods, Figures, Data Availability Section)
**Laboratory animals or Model organisms:** Provide species, strain, sex, age, genetic modification status. Provide accession number in repository **OR** supplier name, catalog number, clone number, **OR** RRID.	Not Applicable	
**Animal observed in or captured from the field:** Provide species, sex, and age where possible.	Not Applicable	
Please detail **housing and husbandry conditions**.	Not Applicable	

Plants and microbes	Information included in the manuscript?	In which section is the information available? (Reagents and Tools Table, Materials and Methods, Figures, Data Availability Section)
**Plants:** provide species and strain, ecotype and cultivar where relevant, unique accession number if available, and source (including location for collected wild specimens).	Not Applicable	
**Microbes:** provide species and strain, unique accession number if available, and source.	Not Applicable	

Human research participants	Information included in the manuscript?	In which section is the information available? (Reagents and Tools Table, Materials and Methods, Figures, Data Availability Section)
If collected and within the bounds of privacy constraints report on age, sex and gender or ethnicity for all study participants.	Yes	Results and Appendix Supplementary Text

Core facilities	Information included in the manuscript?	In which section is the information available? (Reagents and Tools Table, Materials and Methods, Figures, Data Availability Section)
If your work benefited from core facilities, was their service mentioned in the acknowledgments section?	Not Applicable	

Design

Study protocol	Information included in the manuscript?	In which section is the information available? (Reagents and Tools Table, Materials and Methods, Figures, Data Availability Section)
If study protocol has been **pre-registered, provide DOI in the manuscript**. For clinical trials, provide the trial registration number **OR** cite DOI.	Not Applicable	
Report the **clinical trial registration number** (at ClinicalTrials.gov or equivalent), where applicable.	Not Applicable	

Laboratory protocol	Information included in the manuscript?	In which section is the information available? (Reagents and Tools Table, Materials and Methods, Figures, Data Availability Section)
Provide DOI OR other citation details if **external detailed step-by-step protocols** are available.	Not Applicable	

Experimental study design and statistics	Information included in the manuscript?	In which section is the information available? (Reagents and Tools Table, Materials and Methods, Figures, Data Availability Section)
Include a statement about **sample size** estimate even if no statistical methods were used.	Yes	Materials and Methods and Figure Legends
Were any steps taken to minimize the effects of subjective bias when allocating animals/samples to treatment (e.g. **randomization procedure**)? If yes, have they been described?	Not Applicable	
Include a statement about **blinding** even if no blinding was done.	Not Applicable	
Describe **inclusion/exclusion criteria** if samples or animals were excluded from the analysis. Were the criteria pre-established?<br><br>If sample or data points were omitted from analysis, report if this was due to attrition or intentional exclusion and provide justification.	Not Applicable	
For every figure, are **statistical tests** justified as appropriate? Do the data meet the assumptions of the tests (e.g., normal distribution)? Describe any methods used to assess it. Is there an estimate of variation within each group of data? Is the variance similar between the groups that are being statistically compared?	Yes	One-way ANOVA is used for multiple pairwise comparisons. Variance is similar between samples and is assumed to be normally distributed.

Sample definition and in-laboratory replication	Information included in the manuscript?	In which section is the information available? (Reagents and Tools Table, Materials and Methods, Figures, Data Availability Section)
In the figure legends: state number of times the experiment was **replicated** in laboratory.	Yes	Figure Legends
In the figure legends: define whether data describe **technical or biological replicates**.	Yes	Figure Legends

Ethics

Ethics	Information included in the manuscript?	In which section is the information available? (Reagents and Tools Table, Materials and Methods, Figures, Data Availability Section)
Studies involving **human participants**: State details of **authority granting ethics approval** (IRB or equivalent committee(s), provide reference number for approval.	Yes	Materials and Methods
Studies involving **human participants**: Include a statement confirming that **informed consent** was obtained from all subjects and that the experiments conformed to the principles set out in the WMA Declaration of Helsinki and the Department of Health and Human Services Belmont Report.	Yes	Materials and Methods
Studies involving **human participants:** For publication of **patient photos**, include a statement confirming that consent to publish was obtained.	Not Applicable	
Studies involving experimental **animals**: State details of **authority granting ethics approva**l (IRB or equivalent committee(s), provide reference number for approval. Include a statement of compliance with ethical regulations.	Not Applicable	
Studies involving **specimen and field samples:** State if relevant **permits** obtained, provide details of authority approving study; if none were required, explain why.	Not Applicable	

Dual Use Research of Concern (DURC)	Information included in the manuscript?	In which section is the information available? (Reagents and Tools Table, Materials and Methods, Figures, Data Availability Section)
Could your study fall under dual use research restrictions? Please check biosecurity documents and list of **select agents and toxins** (CDC): https://www.selectagents.gov/sat/list.htm	Not Applicable	
If you used a select agent, is the security level of the lab appropriate and reported in the manuscript?	Not Applicable	
If a study is subject to dual use research of concern regulations, is the name of the **authority granting approval and reference number** for the regulatory approval provided in the manuscript?	Not Applicable	

Reporting

The MDAR framework recommends adoption of discipline-specific guidelines, established and endorsed through community initiatives. Journals have their own policy about requiring specific guidelines and recommendations to complement MDAR.

Adherence to community standards	Information included in the manuscript?	In which section is the information available? (Reagents and Tools Table, Materials and Methods, Figures, Data Availability Section)
State if relevant guidelines or checklists (e.g., **ICMJE, MIBBI, ARRIVE, PRISMA**) have been followed or provided.	Not Applicable	
For **tumor marker prognostic studies,** we recommend that you follow the **REMARK** reporting guidelines (see link list at top right). See author guidelines, under 'Reporting Guidelines'. Please confirm you have followed these guidelines.	Not Applicable	
For **phase II and III randomized controlled trials**, please refer to the **CONSORT** flow diagram (see link list at top right) and submit the CONSORT checklist (see link list at top right) with your submission. See author guidelines, under 'Reporting Guidelines'. Please confirm you have submitted this list.	Not Applicable	

Data Availability

Data availability	Information included in the manuscript?	In which section is the information available? (Reagents and Tools Table, Materials and Methods, Figures, Data Availability Section)
Have **primary datasets** been deposited according to the journal's guidelines (see 'Data Deposition' section) and the respective accession numbers provided in the Data Availability Section?	Not Applicable	
Were **human clinical and genomic datasets** deposited in a public access-controlled repository in accordance to ethical obligations to the patients and to the applicable consent agreement?	Not Applicable	
Are **computational models** that are central and integral to a study available without restrictions in a machine-readable form? Were the relevant accession numbers or links provided?	Not Applicable	
If publicly available data were reused, provide the respective **data citations in the reference list**.	Not Applicable	
